# Response-adapted omission of radiotherapy and comparison of consolidation chemotherapy in children and adolescents with intermediate-stage and advanced-stage classical Hodgkin lymphoma (EuroNet-PHL-C1): a titration study with an open-label, embedded, multinational, non-inferiority, randomised controlled trial

**DOI:** 10.1016/S1470-2045(21)00470-8

**Published:** 2022-01

**Authors:** Christine Mauz-Körholz, Judith Landman-Parker, Walentyna Balwierz, Roland A Ammann, Richard A Anderson, Andische Attarbaschi, Jörg M Bartelt, Auke Beishuizen, Sabah Boudjemaa, Michaela Cepelova, Alexander Claviez, Stephen Daw, Karin Dieckmann, Ana Fernández-Teijeiro, Alexander Fosså, Stefan Gattenlöhner, Thomas Georgi, Lisa L Hjalgrim, Andrea Hraskova, Jonas Karlén, Regine Kluge, Lars Kurch, Thiery Leblanc, Georg Mann, Francoise Montravers, Jean Pears, Tanja Pelz, Vladan Rajić, Alan D Ramsay, Dietrich Stoevesandt, Anne Uyttebroeck, Dirk Vordermark, Dieter Körholz, Dirk Hasenclever, William Hamish Wallace

**Affiliations:** a Department of Paediatric Oncology, Justus-Liebig- University Giessen, Giessen, Germany; b Medical Faculty, Martin Luther University Halle-Wittenberg, Halle, Germany; c Department of Radiation Oncology, Martin Luther University Halle-Wittenberg, Halle, Germany; d Department of Paediatric Haematology-Oncology, Sorbonne Université and APHP-SIRIC CURAMUS Hôpital a Trousseau, Paris, France; e Department of Paediatric Oncology and Haematology, Institute of Paediatrics, Jagiellonian University Medical College, Krakow, Poland; f Paediatric Haematology and Oncology, Department of Paediatrics, Inselspital, Bern University Hospital, University of Bern, Bern Switzerland; g MRC Centre for Reproductive Health, Queens Medical Research Institute, University of Edinburgh, Edinburgh, UK; h Department of Paediatric Haematology and Oncology, Medical University of Vienna, Vienna, Austria; i St Anna Children's Hospital, Medical University of Vienna, Vienna, Austria; j Department of Radiology, University Hospital Halle, Halle, Germany; k Princess Máxima Centre for Paediatric Oncology, Utrecht and Erasmus, Sophia Children's Hospital, Rotterdam, The Netherlands; l Department of Pathology, Armand Trousseau Hospital, Paris, France; m Department of Paediatric Haematology and Oncology, University Hospital Motol, Prague, Czech Republic; n Department of Paediatrics, University of Schleswig-Holstein, Kiel, Germany; o Children and Young People's Cancer Service, University College Hospital London, London, UK; p Department of Cellular Pathology, University College Hospital London, London, UK; q Strahlentherapie AKH Wien Medizinische, Universitätsklinik Wien, Vienna, Austria; r Hospital Universitario Virgen Macarena, Universidad de Sevilla, Sevilla, Spain; s Oslo Universitetssykehus, Radiumhospitalet, Oslo, Norway; t Institute of Pathology, Justus-Liebig-University Giessen, Giessen, Germany; u Department of Nuclear Medicine, University of Leipzig, Leipzig, Germany; v Institute for Medical Informatics, Statistics and Epidemiology, University of Leipzig, Leipzig, Germany; w Department of Paediatrics and Adolescent Medicine, The Juliane Marie Centre, University Hospital Rigshospitalet, Copenhagen, Denmark; x Department of Paediatric Haematology and Oncology, National Institute of Children's Disease and Comenius University, Bratislava, Slovakia; y Department of Paediatric Oncology at Astrid Lindgrens Children's Hospital, Karolinska University Hospital, Stockholm, Sweden; z Service d'Hématologie Pédiatrique, Hôpital Robert-Debré, Paris, France; aa Department of Nuclear Medicine, Tenon Hospital, APHP and Sorbonne Université, Paris, France; ab Our Lady's Hospital for Children's Health, Dublin, Ireland; ac Clinical Department of Paediatric Haematology, Oncology, and Stem Cell Transplantation, University Medical Centre Ljubljana and University Children's Hospital, Ljubljana, Slovenia; ad Paediatric Haematology and Oncology, Department of Oncology, University Hospitals Leuven, KU Leuven, Leuven, Belgium; ae Department of Paediatric Haematology and Oncology, Royal Hospital for Children and Young People and University of Edinburgh, Edinburgh, UK

## Abstract

**Background:**

Children and adolescents with intermediate-stage and advanced-stage classical Hodgkin lymphoma achieve an event-free survival at 5 years of about 90% after treatment with vincristine, etoposide, prednisone, and doxorubicin (OEPA) followed by cyclophosphamide, vincristine, prednisone, and procarbazine (COPP) and radiotherapy, but long-term treatment effects affect survival and quality of life. We aimed to investigate whether radiotherapy can be omitted in patients with morphological and metabolic adequate response to OEPA and whether modified consolidation chemotherapy reduces gonadotoxicity.

**Methods:**

Our study was designed as a titration study with an open-label, embedded, multinational, non-inferiority, randomised controlled trial, and was carried out at 186 hospital sites across 16 European countries. Children and adolescents with newly diagnosed intermediate-stage (treatment group 2) and advanced-stage (treatment group 3) classical Hodgkin lymphoma who were younger than 18 years and stratified according to risk using Ann Arbor disease stages IIAE, IIB, IIBE, IIIA, IIIAE, IIIB, IIIBE, and all stages IV (A, B, AE, and BE) were included in the study. Patients with early disease (treatment group 1) were excluded from this analysis. All patients were treated with two cycles of OEPA (1·5 mg/m^2^ vincristine taken intravenously capped at 2 mg, on days 1, 8, and 15; 125 mg/m^2^ etoposide taken intravenously on days 1–5; 60 mg/m^2^ prednisone taken orally on days 1–15; and 40 mg/m^2^ doxorubicin taken intravenously on days 1 and 15). Patients were randomly assigned to two (treatment group 2) or four (treatment group 3) cycles of COPP (500 mg/m^2^ cyclophosphamide taken intravenously on days 1 and 8; 1·5 mg/m^2^ vincristine taken intravenously capped at 2 mg, on days 1 and 8; 40 mg/m^2^ prednisone taken orally on days 1 to 15; and 100 mg/m^2^ procarbazine taken orally on days 1 to 15) or COPDAC, which was identical to COPP except that 250 mg/m^2^ dacarbazine administered intravenously on days 1 to 3 replaced procarbazine. The method of randomisation (1:1) was minimisation with stochastic component and was centrally stratified by treatment group, country, trial sites, and sex. The primary endpoint was event-free survival, defined as time from treatment start until the first of the following events: death from any cause, progression or relapse of classical Hodgkin lymphoma, or occurrence of secondary malignancy. The primary objectives were maintaining 90% event-free survival at 5 years in patients with adequate response to OEPA treated without radiotherapy and to exclude a decrease of 8% in event-free survival at 5 years in the embedded COPDAC versus COPP randomisation to show non-inferiority of COPDAC. Efficacy analyses are reported per protocol and safety in the intention-to-treat population. The trial is registered with ClinicalTrials.gov (trial number NCT00433459) and EUDRACT (trial number 2006-000995-33), and is closed to recruitment.

**Findings:**

Between Jan 31, 2007, and Jan 30, 2013, 2102 patients were recruited. 737 (35%) of the 2102 recruited patients were in treatment group 1 (early-stage disease) and were not included in our analysis. 1365 (65%) of the 2102 patients were in treatment group 2 (intermediate-stage disease; n=455) and treatment group 3 (advanced-stage disease; n=910). Of these 1365, 1287 (94%) patients (435 [34%] of 1287 in treatment group 2 and 852 [66%] of 1287 in treatment group 3) were included in the titration trial per-protocol analysis. 937 (69%) of 1365 patients were randomly assigned to COPP (n=471) or COPDAC (n=466) in the embedded trial. Median follow-up was 66·5 months (IQR 62·7–71·7). Of 1287 patients in the per-protocol group, 514 (40%) had an adequate response to treatment and were not treated with radiotherapy (215 [49%] of 435 in treatment group 2 and 299 [35%] of 852 in treatment group 3). 773 (60%) of 1287 patients with inadequate response were scheduled for radiotherapy (220 [51%] of 435 in the treatment group 2 and 553 [65%] of 852 in treatment group 3. In patients who responded adequately, event-free survival rates at 5 years were 90·1% (95% CI 87·5–92·7). event-free survival rates at 5 years in 892 patients who were randomly assigned to treatment and analysed per protocol were 89·9% (95% CI 87·1–92·8) for COPP (n=444) versus 86·1% (82·9–89·4) for COPDAC (n=448). The COPDAC minus COPP difference in event-free survival at 5 years was −3·7% (−8·0 to 0·6). The most common grade 3–4 adverse events (intention-to-treat population) were decreased haemoglobin (205 [15%] of 1365 patients during OEPA *vs* 37 [7%] of 528 treated with COPP *vs* 20 [2%] of 819 treated with COPDAC), decreased white blood cells (815 [60%] *vs* 231 [44%] *vs* 84 [10%]), and decreased neutrophils (1160 [85%] *vs* 223 [42%] *vs* 174 [21%]). One patient in treatment group 2 died of sepsis after the first cycle of OEPA; no other treatment-related deaths occurred.

**Interpretation:**

Our results show that radiotherapy can be omitted in patients who adequately respond to treatment, when consolidated with COPP or COPDAC. COPDAC might be less effective, but is substantially less gonadotoxic than COPP. A high proportion of patients could therefore be spared radiotherapy, eventually reducing the late effects of treatment. With more refined criteria for response assessment, the number of patients who receive radiotherapy will be further decreased.

**Funding:**

Deutsche Krebshilfe, Elternverein für Krebs-und leukämiekranke Kinder Gießen, Kinderkrebsstiftung Mainz, Tour der Hoffnung, Menschen für Kinder, Programme Hospitalier de Recherche Clinique, and Cancer Research UK.

## Introduction

Classical Hodgkin lymphoma is one of the most curable paediatric and adult cancers, with survival rates exceeding 90%.^1^, ^2^, ^3^, ^4^, ^5^, ^6^ However, survivors are at high risk of secondary cancers, infertility, and cardiovascular disease after chemoradiotherapy.^7^, ^8^, ^9^

The current challenge is to tailor therapy to avoid overtreatment or undertreatment.^10^ Patient subgroups, which differ in disease stage and consequently in their treatment assignment, should have the same high cure rate, with as minimal treatment as possible.

Building on a series of treatment optimisation trials since 1978,^2^ the European Network for Paediatric Hodgkin Lymphoma (EuroNet-PHL) adopted a common comprehensive treatment strategy on the basis of a combined-modality scheme for early-stage (treatment group 1), intermediate-stage (treatment group 2), and advanced-stage (treatment group 3) disease. As part of this treatment strategy, after two intensive induction cycles (with vincristine, etoposide, prednisone, and doxorubicin [OEPA]), treatment group 2 and treatment group 3 received two or four consolidation cycles (cyclophosphamide, vincristine, prednisone, and procarbazine [COPP]) and all patients received involved-field radiotherapy to all initially involved tumour sites. event-free survival at 5 years was in the order of 90%.^2^, ^11^ However, treatment with procarbazine increases the risk of infertility in male patients and premature ovarian insufficiency in female patients.^12^, ^13^, ^14^, ^15^

**Research in context**

**Evidence before this study**

Survivors of childhood and adolescent classical Hodgkin lymphoma are at high risk for secondary cancers, infertility, and cardiovascular disease later in their life after successful chemoradiotherapy combination treatment. The challenge is to sustain high cure rates while reducing long-term side-effects by reducing radiation exposure and avoiding gonadotoxic chemotherapy. We searched MEDLINE between Jan 1, 2005, and Dec 1, 2020, using the terms “Hodgkin lymphoma”, “radiotherapy-induced late effects”, “interim PET”, “procarbazine chemotherapy”, and “fertility”. PET after two or three cycles of chemotherapy is used for response-adapted therapy in patients with classical Hodgkin lymphoma. Procarbazine-free treatment in boys was equally effective as standard treatment with vincristine, prednisone, procarbazine, and doxorubicin (OPPA) followed by cyclophosphamide, vincristine, prednisone, and procarbazine (COPP) in girls. However, convincing data in the literature on the long-term effect of procarbazine in girls and women are scarce.

**Added value of this study**

With the results of our large multinational randomised trial (EuroNet-PHL-C1) in patients younger than 18 years of age with classical Hodgkin lymphoma, we showed that radiotherapy can safely be avoided in intermediate and advanced stage patients who have an adequate response to an intensified induction with vincristine, etoposide, prednisone, and doxorubicin chemotherapy. EFS or overall survival were not compromised when patients received either COPP or cyclophosphamide, vincristine, prednisone, and dacarbazine (COPDAC) consolidation. The results of the randomised embedded non-inferiority study showed that in consolidation treatment, dacarbazine (COPDAC) might be less effective compared to procarbazine (COPP), but is substantially less gonadotoxic in both boys and girls.

**Implications of all the available evidence**

The omission of radiotherapy in adequately responding patients is likely to reduce late-effects of treatment like second malignancies and has become the current treatment approach for young patients with classical Hodgkin lymphoma in the EuroNet-PHL consortium. The replacement of procarbazine with dacarbazine for consolidation is less gonadotoxic in both sexes, therefore COPDAC is now considered the standard consolidation chemotherapy for intermediate and advanced stage patients within the EuroNet-PHL group. The hope is that with this approach radiotherapy-induced second cancers and procarbazine-induced gonadotoxicity in young Hodgkin lymphoma survivors can be avoided. The results of this trial might change the current treatment for classical Hodgkin lymphoma in children and adolescents throughout the EuroNet-PHL consortium and in other international paediatric Hodgkin study groups.

The main objectives of the first EuroNet-PHL prospective trial for classical Hodgkin lymphoma in young people (EuroNet-PHL-C1), which we report here, were twofold. First, to examine whether in patients with adequate PET and morphological response to two cycles of OEPA, radiotherapy could be safely omitted while maintaining the 90% event-free survival target rate at 5 years. Second, to replace procarbazine with the potentially less gonadotoxic drug dacarbazine. Therefore, patients in treatment group 2 and treatment group 3 were randomly assigned to either cyclophosphamide, vincristine, prednisone, and dacarbazine (COPDAC) or COPP with the aim to show non-inferiority and decreased gonadotoxicity. Here, we report only the results of patients with intermediate-stage (treatment group 2) and advanced-stage (treatment group 3) disease. An important secondary objective was to investigate whether patients with inadequate response to OEPA who were scheduled to receive standard radiotherapy after consolidation chemotherapy would still meet the 90% 5-year event-free survival target with COPP or COPDAC and radiotherapy.

## Methods

### Study design and participants

Our study was designed as a titration study with an open-label, embedded, multinational, non-inferiority, randomised controlled trial, and was done at 186 hospital sites across 16 European countries (appendix p 5).

Within a titration study, the objective of treatment tailoring requires looking at patient subgroups, which might be overtreated or undertreated with a given strategy. However, stochastic fluctuations and multiplicity issues notoriously complicate subgroup analyses. Estimating treatment by subgroup interactions generally requires large sample sizes. The treatment titration trial (TTT) design developed for EuroNet-PHL-C1 relies on a peculiarity of paediatric cancer studies, whereby a currently ongoing trial of the respective national study group is often considered the standard of care in that country. The patient populations of the participating trial sites nearly coincide with the trial target population. Therefore, estimating absolute outcome measures is meaningful. TTTs focus on absolute outcome rates, by contrast to randomised controlled trials that assess causal effects estimating treatment differences. TTTs ask whether a given treatment strategy is good enough in defined subgroups of patients relative to a target outcome. This approach is not new; rather the TTT design only explains what series of paediatric study groups trials have implicitly done in the past.^2^, ^11^ Statistically, we replaced estimating differences by estimating absolute-outcome rates, thus increasing power in assessing subgroups.

In the titration study of EuroNet-PHL-C1, we aimed for a titration target rate of 90% event-free survival at 5 years. Treatment reduction can be justified even if it leads to a minor decrease in efficacy, as long as outcome in the respective subgroups remains consistent with the target outcome rate, while toxicity is reduced appreciably. Therefore, the primary objective of the titration study was to analyse whether the event-free survival rates estimates at 5 years in patients with adequate response after two cycles of OEPA treatment without radiotherapy is consistent with a target event-free survival rate of 90% in treatment group 2 and treatment group 3. For the titration study, results of all relevant subgroups defined by treatment group (treatment group 2 or treatment group 3), early-response result (adequate response or inadequate response), or consolidation chemotherapy (COPP or COPDAC) have been analysed, with the primary focus on patients with adequate response, in whom standard radiotherapy was omitted. A secondary objective was to investigate whether the event-free survival rate at 5 years in patients with inadequate response after two cycles of OEPA who received standard involved-field radiotherapy after consolidation chemotherapy is consistent with a target event-free survival rate of 90%.

We also embedded a randomised substudy into the EuroNet-PHL-C1 titration study. The primary objective was to show non-inferiority of COPDAC versus COPP by excluding a decrease of 8% in event-free survival at 5 years. Omission of radiotherapy and randomised consolidation treatement were considered unrelated, that is to say the randomised study was designed to compare consolidation chemotherapies, irrespective of radiotherapy assignment. The randomised study was not additionally powered for any subgroup analysis within the randomised controlled trial. However, subgroup analysis by treatment group was planned. The secondary objective was to investigate whether substitution of dacarbazine for procarbazine in patients in treatment group 2 and treatment group 3 decreased the rate of infertility in male patients and premature menopause in female patients.

Previously untreated patients younger than 18 years with classical Hodgkin lymphoma were included and stratified according to risk using Ann Arbor disease stages IIAE, IIB, IIBE, IIIA, IIIAE, IIIB, IIIBE, and all stages IV (A, B, AE, and BE). Reference pathology was mandatory, and involved the centralised review of all local histopathology specimens (at the patient level) by a second expert, to make sure that the disease diagnosis was uniform and did not comprise other entities of lymphoma, or worse, other non-lymphomatic tumours. Exclusion criteria included the following: pretreatment of Hodgkin lymphoma differing from that of the study protocol (except recommended prephase therapy of a large mediastinal tumour); known hypersensitivity or contraindication to study drugs; diagnosis of lymphocyte-predominant Hodgkin lymphoma; previous chemotherapy or radiotherapy; other (simultaneous) malignancies; pregnancy, lactation, or both; female patients who were sexually active and refused the use of effective contraception (oral contraception, intrauterine devices, a barrier method of contraception in conjunction with spermicidal gel, or surgical sterilisation); current or recent (within 30 days of the start of trial treatment) treatment with another investigational drug or participation in another investigational trial; severe concomitant diseases (eg, immune deficiency syndrome); or known HIV positivity.

Staging was done through clinical assessment, diagnostic imaging, and ^18^F-FDG-PET. Diagnostic imaging included intravenous contrast-enhanced, cross-sectional imaging from the skull base to the symphysis. Investigation of the neck, abdomen, and pelvis was performed either by CT or MRI, whereas a chest CT was mandatory. Patients were assigned to treatment groups according to the reference staging, defined by real-time central board review or local staging (in France only). Patients with Ann Arbor disease stages IA or IB and IIA were assigned to treatment group I, stages IAE and IBE, IIAE, IIB, or IIIA to treatment group 2, and stages IIBE, IIIAE and IIIBE, IIIB, or IVA and IVB to treatment group 3.^11^ Patients in treatment group 2 and treatment group 3 were eligible for the randomised substudy reported here (appendix p 2).

All patients or their guardians provided written informed consent. Local and central ethical boards in each country approved the study, and the study was done in accordance with Good Clinical Practice and the Declaration of Helsinki.

### Randomisation and masking

Patients were randomly assigned (1:1) to COPP and COPDAC within treatment groups, countries, trial sites, and sex by minimisation with a stochastic component. Randomisation was performed before early-response assessment (ERA) to avoid treatment delay (appendix p 2); no platform or website was used for randomisation, and it was instead done manually by the Centre for Clinical Trials (Leipzig, Germany). This study was open label. Only the Central Review Board assessing the ERA was masked to the randomisation result.

### Procedures

All patients received two cycles of OEPA (1·5 mg/m^2^ vincristine taken intravenously capped at 2 mg, on days 1, 8, and 15; 125 mg/m^2^ etoposide taken intravenously on days 1–5; 60 mg/m^2^ prednisone taken orally on days 1–15; and 40 mg/m^2^ doxorubicin taken intravenously on days 1 and 15). Patients in treatment group 2 received two cycles of consolidation therapy and patients in treatment group 3 received four cycles of consolidation therapy, with either COPP (500 mg/m^2^ cyclophosphamide taken intravenously on days 1 and 8; 1·5 mg/m^2^ vincristine taken intravenously capped at 2 mg, on days 1 and 8; 40 mg/m^2^ prednisone taken orally on days 1 to 15; and 100 mg/m^2^ procarbazine taken orally on days 1 to 15) or COPDAC. COPDAC was identical to COPP except that procarbazine was replaced with dacarbazine at 250 mg per m^2^ administered intravenously on days 1 to 3 (appendix p 2). The duration of each cycle was 28 days. The subsequent chemotherapy cycle started on day 29 of each cycle when the following criteria were fulfilled: satisfactory general condition; white blood cell count higher than 2000 cells per mm^3^; absolute neutrophil count higher than 500 cells per mm^3^; platelets higher than 80 000 per mm^3^; and no contraindication to any of the prescribed drugs.

In cases where patients had an expected treatment delay of more than 1 week due to febrile neutropenia, contacting the regional study chairperson was recommended, given that treatment delay can affect efficacy. Before the start of each chemotherapy cycle, differential blood count and alanine aminotransferase, aspartate aminotransferase, gamma glutamyl transferase, bilirubin, and creatinine were measured. Further diagnostic measures (such as electrocardiograms and lung-function assessments) were carried out according to the individual circumstances of the patient. Additionally, blood counts were measured at least twice during every cycle, especially when the haematotoxic effects of chemotherapy were highest. The patient's general condition, assessed with the Lansky and Karnofsky performance index, was documented before therapy and regularly during therapy. Chemotherapy-related toxicity was categorised according to common toxicity criteria (version 2.0).^16^

Adverse events were documented and graded concomitantly to each chemotherapy cycle using a prespecified list of expected adverse events and by describing unexpected adverse events. Long-term sequelae were documented on follow-up case report forms. The primary endpoint was not centrally reviewed.

The premature termination of trial therapy in an individual patient was considered for the following reasons: adverse events or serious adverse events; no response to therapy according to protocol definition; excessive toxicity; at the discretion of the investigator; severe protocol violation; non-compliance of the patient; and administrative problems. In addition, the trial therapy could be terminated by request of the patient, in case of withdrawal of consent, or in case of loss to follow-up. Additional criteria for a patient to be removed from the study are described in the appendix (p 3).

The decision to recommend radiotherapy on completion of chemotherapy was established at ERA on day 29–31 of the second cycle of OEPA. All ERA PET, CT, and MRI scans of all patients in all participating countries were immediately sent to the Central Review Board for real-time review (a necessary step to move on with consolidation treatment without delay), except for France, who had their own regional assessment for decisions on further stratification of their patients. All decision makers (ie, the central and regional review boards) were masked to the randomisation of the patients. ERA was based on morphological tumour-volume response and PET response.^11^ Partial remission corresponded to at least 50% tumour-volume reduction in any involved nodal site. We used four gradual visual categories to assess ERA PET response (category 1, no FDG uptake and activity; category 2, slight FDG uptake and activity; category 3, focal FDG uptake and activity; and category 4, strong FDG uptake and activity). The cutoff for PET positivity was set between the first two and the last two visual categories and corresponds approximately to considering a visual Deauville score of 3 or higher as positive.^17^ Adequate response at ERA was defined as partial remission or greater and PET negativity. Radiotherapy was omitted in patients who responded adequately to consolidation chemotherapy. Patients with an inadequate response received radiotherapy at a dose of 19·8 Gy (usually 11 fractions of 1·8 Gy per day) at all initially involved tumour sites. Residual masses of more than 100 mL at ERA and sites that responded slowly to treatment (<75% volume reduction at ERA in lesions with minimal residual volume of >5 mL) received an additional boost of 10 Gy.

Fertility-related parameters in patients who had been through puberty were assessed by endocrine analysis at diagnosis and at least 12 months after start of treatment. Gonadal function in patients who had been through puberty was assessed by measurement of serum reproductive hormones, and semen analyses in male individuals according to recently assessed standards. Semen analysis was classified as either normal or abnormal if sperm concentration, morphology, or motility were outside reference ranges.^18^ In male individuals, serum follicle-stimulating hormone (FSH) concentrations higher than 10 IU per litre with or without inhibin-B concentrations of less than 100 ng per litre were considered to indicate spermatogenic damage and probable azoospermia,^19^ supported by direct semen analysis.^18^ Inhibin B was also measured as an index of spermatogenesis.^20^ In female individuals, FSH concentrations higher than 25 IU per litre were used to indicate premature ovarian insufficiency.^21^ In case of several assessments, the latest sample in male individuals and the highest FSH value in female individuals was analysed. FSH and inhibin were measured locally by standard laboratory tests.

### Outcomes

The primary endpoint was event-free survival, defined as time from treatment start until the first of the following events: death from any cause, progression or relapse of classical Hodgkin lymphoma, or occurrence of secondary malignancy. Secondary endpoints in all patients were overall survival, defined as time from start of treatment to death from any cause, progression-free survival, defined as time from treatment start until the first of the following events: death from any cause; progression of classical Hodgkin lymphoma, or relapse of classical Hodgkin lymphoma; evidence of male infertily score (FSH measurements, semen analysis if available, and inhibin B); female sexual functioning score (FSH measurements and rate of premature ovarian insufficiency, defined as FSH >25 IU/L); and long-term data.

### Statistical analysis

This pragmatic trial aimed to include all patients available within an enrolment period of 6 years in the TTT to maximise power in the relevant subgroups, with at least 1216 patients required for treatment group 2 and treatment group 3. We estimated event-free survival at 5 years in the subgroups implied by the design with two-sided 95% CI. Subgroup outcome was considered consistent with the target rate if the CI included or was higher than 90%. The main results of the TTT were compiled in a forest plot. The primary objective was to confirm that the 5-year event-free survival was consistent with a 90% target rate in patients with adequate response, in whom standard radiotherapy was omitted. The secondary objective was to confirm that the 5-year event-free survival was still consistent with the 90% target rate in patients with inadequate response.

For the randomised substudy, 700–1100 patients were expected to be eligible. To claim non-inferiority of COPDAC versus COPP, we aimed to exclude a difference of 8% from the two-sided 95% CI for the event-free survival rate at 5 years. Assuming an event-free survival rate of 90% with COPP, the expected sample size would need to provide 80% power to show non-inferiority, as long as COPDAC was less than 2% inferior to COPP. Annual interim analyses were scheduled after the third year to monitor for unexpected superiority using a two-sided α of 0·1%. Subgroup analyses by treatment group and ERA response (with adequate response occurring when radiotherapy was omitted and inadequate response occurring with standard radiotherapy) were implicit by the study design. Only events within a follow-up of 72 months were considered.

We did both an intention-to-treat (ITT) and a per-protocol analysis. The per-protocol set excluded patients who progressed or died before the start of consolidation chemotherapy, and patients who did not receive the assigned treatment or without treatment assignment at ERA. These protocol deviations included patients who received no radiotherapy but had an inadequate response at ERA, patients who received radiotherapy but had an adequate response at ERA, and patients who switched chemotherapy groups after randomisation. Patients with disease progression or serious toxicity during consolidation chemotherapy were not excluded. Here, we report the results of the per-protocol analysis because the study aimed to establish non-inferiority. Data obtained from the ITT analysis are shown in the appendix (pp 24–29).

Toxicity profiles of the OEPA, COPP, and COPDAC regimens are described using regimen-specific safety populations consisting of all ITT patients who received the respective regimens. Analyses were done using R version 4.03.

Estimates of 5-year rates with two-sided 95% CIs were obtained with the Kaplan-Meier product-limit estimator for all time-to-event endpoints (event-free survival, overall survival, and progression-free survival). For the titration study, results of all relevant subgroups defined by treatment group (treatment group 2 or treatment group 3), early-response result (adequate response or inadequate response), or consolidation chemotherapy (COPP or COPDAC) have been analysed, with the primary focus being on patients with adequate response, in whom standard radiotherapy was omitted. Subgroup outcome was considered consistent with the target rate if the 95% CI included or was higher than 90%. The main results of the TTT were compiled in a forest plot for event-free survival.

FSH (male and female) and data on inhibin B after COPP or COPDAC consolidation chemotherapy were presented by plotting empirical cumulative-distribution curves and compared using Mann-Whitney U tests with a two-sided 5% significance threshold. Results of the semen analysis were compared using Fisher's exact test.

The trial was registered ClinicalTrials.gov (trial number NCT00433459) and EUDRACT (trial number 2006-000995-33).

### Role of the funding source

The funders of the study had no role in study design, data collection, data analysis, data interpretation, or writing of the report.

## Results

**Figure 1:**
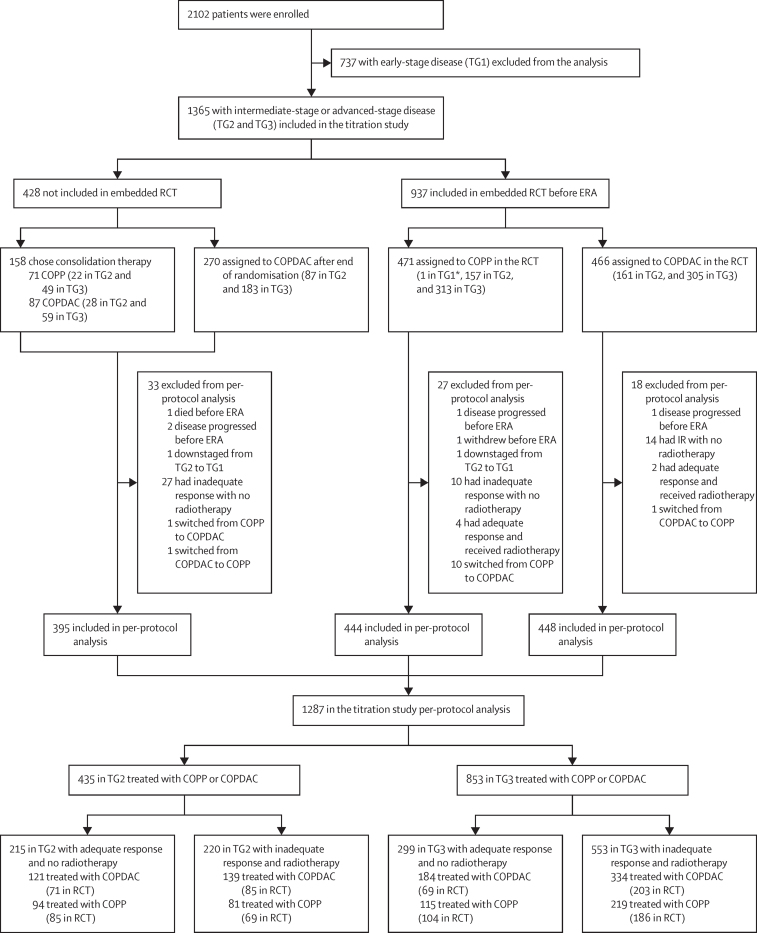
Trial profile of the titration study with embedded randomisation COPP=cyclophosphamide, vincristine, prednisone, and procarbazine. ERA=early response assessment. ITT=intention to treat. RCT=randomised controlled trial. TG1=treatment group 1. TG2=treatment group 2. TG3=treatment group 3. *One patient initially assigned to TG2 was downstaged and treated as being in TG1 according to the local investigator's discretion. COPDAC=cyclophosphamide, vincristine, prednisone, and dacarbazine.

Between Jan 31, 2007, until Jan 30, 2013, 2102 patients were enrolled. 737 (35%) of the 2102 recruited patients were in treatment group 1 (early-stage disease), and therefore are not included in our analysis and will be reported separately. 1365 (65%) of the 2102 patients were in treatment group 2 (intermediate-stage disease; n=455) and treatment group 3 (advanced-stage disease; n=910). Of these 1365 patients, 1287 (94%) patients (435 [34%] were in treatment group 2 and 852 [66%] of 1287 were in treatment group 3) were included in the titration trial per-protocol analysis. 514 (40%) of 1287 patients with adequate response received no radiotherapy (215 [49%] of 435 in treatment group 2 and 299 [35%] of 852 in treatment group 3). 773 (60%) of 1287 patients with inadequate response were scheduled for radiotherapy (220 [51%] of 435 in the treatment group 2 and 553 [65%] of 852 in treatment group 3; figure 1), and all but one patient had a positive PET at ERA. Only one patient had an inadequate morphological response and was PET negative.

158 (12%) of 1365 patients refused random assignment to treatment and chose either COPP (n=71) or COPDAC (n=87). Randomisation was stopped at 937 patients randomised between COPP (n=471) or COPDAC (n=466) consolidation because of a perceived change in the risk–benefit assessment, with emerging evidence from interim analyses of this trial that COPP and COPDAC are similarly efficacious, but COPDAC was less likely to impair male fertility. Thereafter, 270 patients were assigned to COPDAC. The per-protocol set of the embedded randomised cohort consisted of 444 patients treated with COPP and 448 patients treated with COPDAC after exclusion of 45 patients with protocol deviations (figure 1). Patient characteristics of 1287 patients in the per-protocol group of the titration study and 892 patients in the per-protocol group of the randomised trial are described in table 1. The adequate-response rate was lower in COPDAC (189 [43%] of 444) than in COPP (160 [36%] of 448), although ERA was masked to the randomisation result. The characteristics of patients who were in the per-protocol group and who chose COPP or COPDAC, along with the characteristics of patients who were assigned to COPDAC after stopping the randomisation, are described the appendix (p 14).

**Table 1:** Demographic data of patients in the per-protocol group in the titration study and the embedded, randomised cohort

	**Patients in the titration study (n=1287)**	**Patients in the embedded, randomised cohort (n=892)**	**Patients randomly assigned to COPP (n=444)**	**Patients randomly assigned to COPDAC (n=448)**
**Age, years**				
≥13 years	934 (73%)	652 (73%)	317 (71%)	335 (75%)
<13 years	353 (27%)	240 (27%)	127 (29%)	113 (25%)
Median (IQR)	14·8 (12·8–16·2)	14·8 (12·8–16·3)	14·7 (12·6–16·1)	15·0 (12·9–16·3)
**Sex**				
Male	717 (56%)	494 (55%)	244 (55%)	250 (56%)
Female	570 (44%)	398 (45%)	200 (45%)	198 (44%)
Combined stage*				
2A	3 (<1%)	1 (<1%)	1 (<1%)	0
2AE	93 (7%)	66 (7%)	30 (7%)	36 (8%)
2B	194 (15%)	140 (16%)	73 (16%)	67 (15%)
2BE	114 (9%)	85 (10%)	49 (11%)	36 (8%)
3A	157 (12%)	112 (13%)	56 (13%)	56 (13%)
3AE	30 (2%)	20 (2%)	8 (2%)	12 (3%)
3B	148 (11%)	97 (11%)	51 (11%)	46 (10%)
3BE	54 (4%)	41 (5%)	20 (5%)	21 (5%)
4A	167 (13%)	112 (13%)	49 (11%)	63 (14%)
4AE	44 (3%)	26 (3%)	17 (4%)	9 (2%)
4B	189 (15%)	130 (15%)	61 (14%)	69 (15%)
4BE	94 (7%)	62 (7%)	29 (7%)	33 (7%)
**Ann Arbor stage**				
2	404 (31%)	292 (33%)	153 (34%)	139 (31%)
3	389 (30%)	270 (30%)	135 (30%)	135 (30%)
4	494 (38%)	330 (37%)	156 (35%)	174 (39%)
**B symptoms**				
No	494 (38%)	337 (38%)	161 (36%)	176 (39%)
Yes	793 (62%)	555 (62%)	283 (64%)	272 (61%)
**Bulky disease**†				
No	635 (49%)	429 (48%)	222 (50%)	207 (46%)
Yes	602 (47%)	422 (47%)	201 (45%)	221 (49%)
**Response group**				
Adequate response	514 (40%)	349 (39%)	189 (43%)	160 (36%)
Inadequate response	773 (60%)	543 (61%)	255 (57%)	288 (64%)
**Treatment group and ERA response**				
TG 2 adequate response	215 (17%)	156 (17%)	85 (19%)	71 (16%)
TG 2 inadequate response	220 (17%)	154 (17%)	69 (16%)	85 (19%)
TG 3 adequate response	299 (23%)	193 (22%)	104 (23%)	89 (20%)
TG 3 inadequate response	553 (43%)	389 (44%)	186 (42%)	203 (45%)

Data are n (%). COPP=cyclophosphamide, vincristine, prednisone, and procarbazine. COPDAC=cyclophosphamide, vincristine, prednisone, and dacarbazine. ERA=early-response assessment. TG2=treatment group 2. TG3=treatment group 3.

* Combined stage consists of the Ann Arbor stage combined with B symptoms (ie, unexplained fever >38.5°C, weight loss of 10% during the past 6 months, and drenching night sweats) and E stage (ie, lymphoma with contiguous invasion of neighbouring organs or tissue).

† Bulky disease is defined as a contiguous tumour volume of at least 200 mL.

Follow-up duration was 5 years for 80% of all patients. Median follow-up was 66·5 months (IQR 62·7–71·7). Within 72 months of the start of the trial, 153 event-free survival events occurred in the per-protocol set (38 in treatment group 2 and 115 in treatment group 3), with recurrent classical Hodgkin lymphoma in 143 patients (35 in treatment group 2 and 108 in treatment group 3). In ten patients, secondary malignancies as first events occurred 6·9–61·9 months after start of treatment with the following diagnoses: four thyroid cancers; one Langerhans-cell histiocytosis; two B-cell lymphomas; two cases of acute myeloid leukaemia; and one alveolar rhabdomyosarcoma (appendix p 37). 22 patients in the per-protocol group died (six in treatment group 2 and 16 in treatment group 3), 21 of whom died after recurrent disease, and one patient in treatment group 3 died after secondary malignancy as a first event.

**Figure 2:**
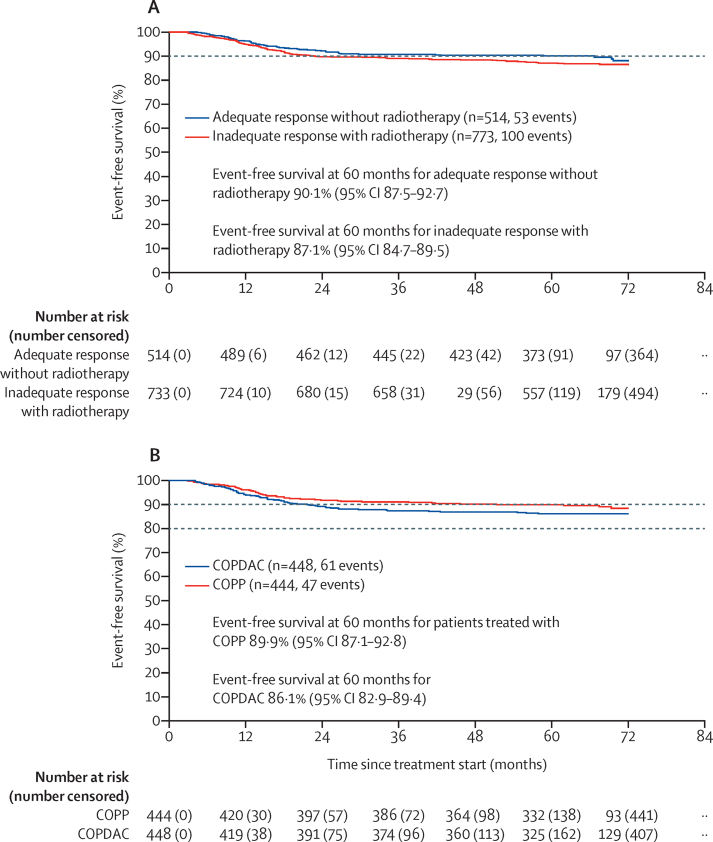
Event-free survival of patients in the per-protocol group in the titration trial and the embedded randomised trial (A) Event-free survival of the patients in the per-protocol group in the titration trial, for patients with adequate response without radiotherapy (blue) and patients with inadequate response scheduled for radiotherapy (red). (B) Event-free survival of the patients in the per-protocol group in the embedded randomisation trial who were treated with COPP (red) and COPDAC (blue). COPP=cyclophosphamide, vincristine, prednisone, and procarbazine. COPDAC=cyclophosphamide, vincristine, prednisone, and dacarbazine.

**Figure 3:**
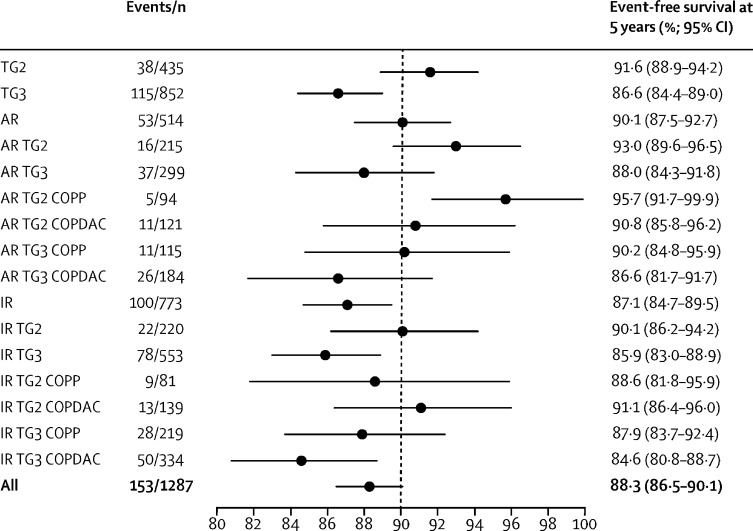
Forest plot of point estimates of event-free survival for relevant subgroups in the titration study (per-protocol population) Point estimates and 95% CIs for relevant subgroups in the titration study are shown. AR=adequate response. COPP=cyclophosphamide, vincristine, prednisone, and procarbazine. COPDAC=cyclophosphamide, vincristine, prednisone, and dacarbazine. IR=inadequate response. TG2=treatment group 2. TG3=treatment group 3.

Event-free survival rates at 5 years in patients with adequate response and omission of radiotherapy were consistent with the target rate of 90%. event-free survival rate at 5 years (primary endpoint) in all patients who responded adequately was 90·1% (95% CI 87·5–92·7; figure 2A). Overall survival and progression-free survival rates at 5 years are shown in the appendix (pp 30–33). We obtained similar event-free survival and progression-free survival rates at 5 years in the ITT analysis (appendix pp 24–26). Subgroup analyses showed event-free survival rates at 5 years of 93·0% (89·6–96·5) in people with adequate response in treatment group 2 and 88·0% (84·3–91·8) in those with adequate response in treatment group 3, 92·7% (89·2–96·3) in those with adequate response who received COPP, and 88·3% (84·7–92·0) in those with adequate response who received COPDAC. In addition, in patients with adequate response in treatment group 2 or treatment group 3 subgroups treated with COPP or COPDAC, Event-free survival rates at 5 years were within the 90% target rate (figure 3).

The 773 patients with inadequate response were scheduled for standard radiotherapy; 208 (27%) of 773 patients received boost irradiation, 45 (22%) of 208 patients were in treatment group 2 and 163 (78%) of 208 patients were in treatment group 3. Thus 45 (20%) of 220 patients in treatment group 2 and 163 (29%) of 553 in treatment group 3 received boost radiotherapy. The event-free survival rate at 5 years in the inadequate-response group was 87·1% (95% CI 84·7–89·5), lower than the 90% target (figure 2A). Overall survival results in these patients are shown in the appendix (p 30). We obtained similar event-free survival rates at 5 years in the ITT analysis (appendix p 24). Subgroup analyses showed event-free survival rates at 5 years of 90·1% (95% CI 86·2–94·2) in people with inadequate response in treatment group 2 and 85·9% (83·0–88·9) in those with inadequate response in treatment group 3, 88·1% (84·4–91·9) in those with inadequate response who received COPP, and 86·5% (83·4–92·0) in those with adequate response who received COPDAC.

The event-free survival rate at 5 years in 892 patients who were randomly assigned to COPP or COPDAC treatment and analysed per protocol was 89·9% (95% CI 87·1–92·8) in the 444 patients who received COPP versus 86·1% (82·9–89·4) in the 448 patients who received COPDAC (figure 2B). We obtained similar event-free survival rates at 5 years in the ITT analysis (appendix p 27). The COPDAC minus COPP difference in 5-year event-free survival; rate was −3·7% (95% CI–8·0 to 0·6) in the per-protocol analysis, and −3·1% (−7·5 to 1·3) in the ITT analysis. 5-year overall survival rates are shown in the appendix (p 29 and 31).

In an unplanned subgroup analysis, in patients with inadequate response, the 5 year event-free survival rate was 88·3% (95% CI 84·4–92·4) with COPP and 87·9% (84·2–91·8) with COPDAC (appendix p 34). Thus, the treatment difference was −0·4% (−5·9 to 5·1). However, in patients with adequate response, the 5-year event-free survival rate was 91·9% (95% CI 88·1–95·9) with COPP and 82·9% (77·2–89·0) with COPDAC (appendix p 35). The treatment difference was −9·1% (−16·1 to −2·0). There was no difference in overall survival between COPP and COPDAC, with or without radiotherapy (data not shown).

**Figure 4:**
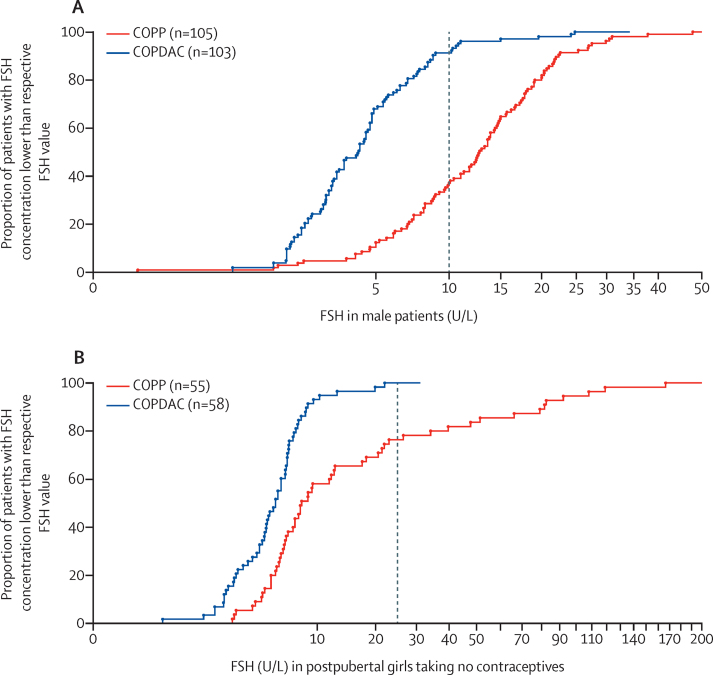
FSH serum concentrations in male and female patients after COPP and COPDAC treatment FSH serum concentrations were obtained at least 12 months after start of treatment from male (A) and female (B) patients randomly assigned to either COPP or COPDAC consolidation treatment. Data are depicted as an empirical cumulative-distribution function; the dotted vertical lines indicate cutoff values beyond which gonadal damage is suspected. FSH=follicle-stimulating hormone.

Pretreatment semen analyses were done in 127 (19%) of 717 male patients in the per-protocol group with intermediate and advanced disease stages. Of these 127, 44 (35%) were normospermic, 60 (47%) had an abnormal analysis, and 23 (18%) were azoospermic. Azoospermia at time of diagnosis was detected in five (13%) of 39 patients in treatment group 2 and in 18 (20%) of 88 of patients in treatment group 3 (p=0·44). After a median of 40 months follow-up, 19 (83%) of 23 patients had azoospermia after COPP, but none of the 22 patients who received COPDAC had azoospermia (p<0·0001). There was no suggestion of a procarbazine dose effect: nine (90%) of ten patients exposed to two cycles of COPP had azoospermia compared with ten (77%) of 13 patients exposed to four cycles. FSH was elevated (higher than 10 U/L) in 69 (66%) of 105 boys who were randomly assigned to COPP (median 13·6 U/L, IQR 8·6–19·7) compared with nine (9%) of 103 patients who were randomly assigned to COPDAC (median 4·2 U/L, 2·6–6·4; p<0·0001; figure 4A). Inhibin B was reduced after treatment with COPP compared with COPDAC (p<0·0001; appendix p 17).

Serum FSH in female patients who had gone through puberty and who were not taking hormonal contraceptives was measured in the per-protocol group at presentation in 132 (23%) of 570 female patients and at least 12 months after start of treatment in 156 (27%) of 570 female patients. At presentation, only one girl in treatment group 3 (with disease-stage 4B) had FSH concentrations higher than 25 IU/L. However, during follow-up (median 29·4 months after start of treatment; IQR 21·1–45·3) FSH was significantly increased in 55 female individuals randomly assigned to COPP (median 8·1 U/L, 5·7–22·5) compared with 58 female individuals randomly assigned to COPDAC (median 5·5 U/L, 3·4–6·7; p<0·0001; figure 4B), 15 female patients had FSH concentrations higher than 25 U/L during follow-up (median 18·1 months after start of treatment, IQR 15·5–34·8). All of these female patients had been exposed to four cycles of COPP, and only three of them had received radiotherapy to a field that included their ovaries.

More than 90% of all chemotherapy cycles (OEPA, COPP, and COPDAC) were administered with more than 90% of the prescribed drug doses (appendix p 15). The duration of the consolidation cycles were 28 days in more than 75% of cycles and treatment delay was not more than 1 week in more than 90% of cycles (appendix p 23). In seven patients (one in treatment group 2 and six in treatment group 3), study treatment could not be continued, and alternative drugs were used (appendix p 16). The majority of adverse events occurred as haematological toxicity (table 2). The most common grade 3–4 adverse events were decreased haemoglobin (205 [15%] of 1365 patients during OEPA *vs* 37 [7%] of 528 treated with COPP *vs* 20 [2%] of 819 treated with COPDAC), decreased white blood cells (815 [60%] *vs* 231 [44%] *vs* 84 [10%]), and decreased neutrophils (1160 [85%] *vs* 223 [42%] *vs* 174 [21%]). One patient died after treatment-related sepsis during OEPA, and there were a further 24 non-treatment related deaths. Other rare, severe adverse reactions of CTCAE grades 3 and 4 are described in the appendix pp (17–22).

**Table 2:** Adverse events during OEPA, COPP, and COPDAC cycles in the ITT patient cohort

	**OEPA (n=1365)**				**COPP (n=528)**				**COPDAC (n=819)**			
	Grade 1	Grade 2	Grade 3	Grade 4	Grade 1	Grade 2	Grade 3	Grade 4	Grade 1	Grade 2	Grade 3	Grade 4
Decreased haemoglobin	337 (25%)	639 (47%)	187 (14%)	18 (1%)	178 (34%)	162 (31%)	33 (6%)	4 (<1%)	296 (36%)	215 (26%)	18 (2%)	2 (<1%)
Decreased white blood cells	131 (10%)	332 (24%)	463 (34%)	352 (26%)	79 (15%)	166 (31%)	153 (29%)	78 (15%)	225 (27%)	259 (32%)	73 (9%)	11 (1%)
Decreased neutrophils	39 (3%)	76 (6%)	220 (17%)	940 (71%)	82 (16%)	133 (26%)	128 (25%)	95 (18%)	160 (20%)	198 (25%)	130 (16%)	44 (5%)
Decreased platelets	119 (9%)	63 (5%)	51 (4%)	8 (<1%)	58 (11%)	20 (4%)	12 (2%)	1 (<1%)	37 (4%)	18 (2%)	15 (2%)	0
Creatinine increase	67 (5%)	6 (<1%)	0	0	33 (6%)	0	1 (<1%)	0	59 (7%)	4 (<1%)	0	0
Bilirubin increase	59 (4%)	23 (2%)	2 (<1%)	0	36 (7%)	2 (<1%)	0	0	39 (5%)	5 (<1%)	0	0
Liver enzymes increase	577 (43%)	235 (18%)	77 (6%)	6 (<1%)	245 (47%)	74 (14%)	21 (4%)	0	369 (47%)	101 (13%)	23 (3%)	0
Fever	270 (20%)	88 (7%)	12 (<1%)	5 (<1%)	75 (14%)	25 (5%)	3 (<1%)	0	88 (11%)	31 (4%)	2 (<1%)	0
Infection	207 (15%)	191 (14%)	77 (6%)	9 (<1%)	76 (14%)	61 (12%)	21 (4%)	1 (<1%)	134 (16%)	51 (6%)	19 (2%)	1 (<1%)
Stomatitis and pharyngitis	308 (23%)	188 (14%)	42 (3%)	4 (<1%)	79 (15%)	19 (4%)	1 (<1%)	2 (<1%)	104 (13%)	20 (2%)	0	0
Vomiting	258 (19%)	218 (16%)	15 (1%)	4 (<1%)	65 (12%)	44 (8%)	6 (1%)	0	89 (11%)	45 (5%)	1 (<1%)	0
Diarrhoea	172 (13%)	83 (6%)	14 (1%)	11 (<1%)	29 (5%)	7 (1%)	3 (<1%)	1 (<1%)	47 (6%)	21 (3%)	1 (<1%)	0
Constipation	287 (21%)	156 (11%)	30 (2%)	2 (<1%)	72 (14%)	30 (6%)	5 (<1%)	0	108 (13%)	35 (4%)	0	1 (<1%)
Neuropathy (sensory)	244 (18%)	69 (5%)	25 (2%)	1 (<1%)	100 (19%)	37 (7%)	15 (3%)	2 (<1%)	121 (15%)	49 (6%)	24 (3%)	0
Neuropathy (motor activity)	152 (11%)	63 (5%)	28 (2%)	1 (<1%)	55 (10%)	36 (7%)	21 (4%)	3 (<1%)	85 (10%)	45 (5%)	27 (3%)	2 (<1%)

One patient in treatment group 2 died of sepsis after the first course of OEPA. OEPA=vincristine, etoposide, prednisone, and doxorubicin. COPP=cyclophosphamide, vincristine, prednisone, and procarbazine. COPDAC=cyclophosphamide, vincristine, prednisone, and dacarbazine. ITT=intention to treat.

## Discussion

EuroNet-PHL-C1 was a large multinational trial done in 16 European countries. The participating national paediatric classical Hodgkin lymphoma consortia agreed on a comprehensive treatment strategy for all stages of the disease. Here we presented the results of patients younger than 18 years with intermediate-stage and advanced-stage classical Hodgkin lymphoma. We have shown that radiotherapy can safely be avoided in patients with intermediate-stage and advanced-stage disease who have an adequate response to OEPA induction without compromising 5-year event-free survival or overall survival when treated with either COPP or COPDAC consolidation. In a randomised, embedded, non-inferiority study for patients with intermediate-stage and advanced-stage disease, we have shown that dacarbazine can replace procarbazine with reduced gonadal toxicity and similar event-free survival in patients who had radiotherapy. All cases of observed gonadal damage in both sexes were in those exposed to COPP, with none observed in those receiving COPDAC (ie, all boys with azoospermia after treatment and all girls with FSH concentrations >25 IU/L after treatment, consistent with premature ovarian insufficiency, were exposed to COPP).

Our first objective was to investigate whether radiotherapy can be safely omitted in patients with adequate response to intensive OEPA induction chemotherapy. We implemented PET-guided omission of radiotherapy in adequate responders without randomising against standard radiotherapy, to gain sufficient power for the titration questions. This concept is fully in line with the treatment-titration strategy of our consortium, in which reduction of serious adverse events is traded off with minor reductions in treatment efficacy.^22^, ^11^ In previous studies, we observed an event-free survival at 5 years of around 90% with OEPA–COPP and radiotherapy in all patients. Therefore, for our titration design, we used a target rate of 90% event-free survival at 5 years with less treatment burden in patients who were adequately responding.

With the treatment-titration design, we have shown that radiotherapy can be avoided in about 40% of patients while keeping event-free survival at 5 years consistent with a target rate of 90%. Adult classical Hodgkin lymphoma studies have similarly used PET-guided treatment adaptation successfully for omitting radiotherapy^23^, ^24^ or reducing chemotherapy burden^25^ in patients who responded adequately, defined as a PET response lower than a Deauville 3 score.

Without randomisation, we cannot exclude a minor loss in efficacy when radiotherapy is omitted. However, event-free survival remains in the range of 90% while avoiding large-field radiotherapy with its associated potential long-term sequelae.

Without randomisation, the prognostic value of FDG-PET cannot be established. However, event-free survival curves of patients with and without radiotherapy are nearly superimposable, indicating that the prognostic value of interim FDG-PET is low. Adequate response was defined cautiously, requiring a Deauville score lower than 3 when the study was designed in 2005. Today, a Deauville score lower than 4 would be considered to indicate complete metabolic remission.^26^

The second objective was to eliminate procarbazine from consolidation chemotherapy, to reconfirm previous trial results of the consortium.^2^, ^11^, ^27^ Procarbazine has been used extensively in the treatment of classical Hodgkin lymphoma for induction and consolidation with great efficacy,^1^, ^3^, ^23^ but has been repeatedly shown to impair spermatogenesis and to cause premature ovarian insufficiency.^12^, ^13^, ^15^ At the time EuroNet-PHL-C1 was designed, COPDAC had shown promising early results in the preceding gender-stratified study GPOH-HD-2002.^11^ However, in that trial, all patients with intermediate-stage and advanced-stage disease received radiotherapy. Here, we investigated in an embedded randomised trial whether COPDAC is not inferior compared with COPP. The results show that non-inferiority is marginal in the per-protocol set, with a predefined tolerance limit (−8% in event-free survival at 5 years) just on the lower boundary of the two-sided 95% CI for the treatment difference in the per-protocol population. The point estimate for the difference in event-free survival at 5 years is −3·7% for COPDAC compared with COPP. This result was surprising because of the excellent results of COPDAC in the GPOH-HD-2002 trial,^11^ in which all patients received radiotherapy. Therefore, we did an unplanned subgroup analysis for patients with and without radiotherapy. The event-free survival curves of COPP and COPDAC in patients who had radiotherapy are superimposable. However, in the subgroup of patients who did not undergo radiotherapy, COPDAC appears inferior to COPP with a point estimate for the difference in event-free survival at 5 years of −9%. This finding might indicate that COPDAC is less effective than COPP in patients who have not had radiotherapy. However, subgroup analyses are typically underpowered, and their results are prone to chance fluctuation.

The results of all patients randomly assigned to treatment showed a difference of −3·7% for COPDAC when compared with COPP. This result is in line with those of the titration trial that includes 428 patients who were not randomly assigned to treatment.

The clinically important question of whether COPDAC without radiotherapy can replace COPP without radiotherapy in patients who respond adequately was already answered by the titration study.

The Paediatric Oncology Group^5^ and Childrens' Oncology Group trials^6^ have also previously shown excellent outcomes in patients at intermediate and high risk without the use of procarbazine. A study in patients at intermediate risk^6^ and one in patients at high risk^28^ showed that a procarbazine-free regimen can be used to achieve similar outcomes with or without reduced need for radiotherapy on the basis of early response to therapy. In adult consortia, either less toxic but less effective adriamycin, bleomycine, vincristine, and dacarbazine (ABVD) or more toxic but more effective bleomycine, etoposide, adriamycine, cyclophosphamide, vincristine, procarbazine, and prednisone (BEACOPP) has been used.^29^ The results of the present study and other paediatric-focused regimens could therefore bridge the gap between ABVD and the intensive-escalated BEACOPP regimens. These paediatric regimens should be explored in other age groups.

Our study provides strong evidence for the benefit of removing procarbazine on reproductive function. Procarbazine had a clear detrimental effect on FSH, both in male and female patients, and all cases of observed gonadal damage in both sexes were in those exposed to COPP, with none in those receiving COPDAC. Although the fertility data presented here are limited, fertility data after treatment of young people with cancer and classical Hodgkin lymphoma in particular are notoriously sparse and incomplete. Young men who have been treated for cancer are generally reluctant to produce semen for analysis. The reasons for this reluctance include that oncologists do not often discuss fertility with their patients but concentrate on disease control. Discussion can be embarrassing and difficult to bring up and there is often a reluctance to go to a fertility centre and masturbate to produce a specimen. Many young people take a long time to come to terms with their fertility prognosis after cancer treatment and providing semen, usually within a hospital environment, can be quite threatening. The hormonal data presented, including inhibin B concentrations in men, indicate robust power to support a clear interpretation in both male and female individuals.

A secondary objective was to investigate whether event-free survival at 5 years is consistent with a target rate of 90% in patients with inadequate response to OEPA who received radiotherapy. The effects of advanced disease stage, inadequate response to induction chemotherapy, and treatment with slightly less efficacious, but non-gonadotoxic COPDAC consolidation chemotherapy on event-free survival are small and appear to be additive.

OEPA induction followed by COPDAC consolidation chemotherapy, with radiotherapy only for patients with a Deauville score higher than 3, has been taken forward as the standard treatment in the subsequent EuroNet-PHL-C2 trial.^30^ The aim of the research is to clarify whether intensified COPDAC without radiotherapy can achieve improved event-free survival rates higher than 90%. In EuroNet-PHL-C2, the comparator to COPDAC is a procarbazine-free intensified consolidation chemotherapy, which might be both more efficacious and non-gonadotoxic. We have shown that radiotherapy can safely be avoided in patients with intermediate-stage and advanced-stage disease who have an adequate response to intensified OEPA induction without compromising event-free or overall survival when treated with either COPP or COPDAC consolidation. Furthermore, in a randomised embedded non-inferiority study for patients with intermediate-stage and advanced-stage disease, we have shown that dacarbazine can replace procarbazine with reduced gonadal toxicity and comparable event-free survival.

## Data sharing

The study protocol is available in the appendix (p 38). Individual participant data that underlie the results reported in this Article (text, tables, figures, and appendices) will be shared after deidentification to researchers who provide a methodologically sound and ethically approved proposal. Proposals can be submitted up to at least 36 months after Article publication. Proposals should be directed to dirk.hasenclever@imise.uni-leipzig.de; to gain access, data requestors will need to sign a data-access agreement.

## Declaration of interests

We declare no competing interests.

## References

[1] Schellong G, Bramswig J, Ludwig R (1986). Combined treatment strategy in over 200 children with Hodgkin's disease: graduated chemotherapy, involved field irradiation with low dosage and selective splenectomy. A report of the cooperative therapy study DAL-HD-82. Klin Padiatr.

[2] Schellong G, Potter R, Bramswig J (1999). High cure rates and reduced long-term toxicity in pediatric Hodgkin's disease: the German-Austrian multicenter trial DAL-HD-90. The German-Austrian Pediatric Hodgkin's Disease Study Group. J Clin Oncol.

[3] Weiner MA, Leventhal B, Brecher ML (1997). Randomized study of intensive MOPP-ABVD with or without low-dose total-nodal radiation therapy in the treatment of stages IIB, IIIA2, IIIB, and IV Hodgkin's disease in pediatric patients: a Pediatric Oncology Group study. J Clin Oncol.

[4] Donaldson SS, Hudson MM, Lamborn KR (2002). VAMP and low-dose, involved-field radiation for children and adolescents with favorable, early-stage Hodgkin's disease: results of a prospective clinical trial. J Clin Oncol.

[5] Schwartz CL, Constine LS, Villaluna D (2009). A risk-adapted, response-based approach using ABVE-PC for children and adolescents with intermediate- and high-risk Hodgkin lymphoma: the results of P9425. Blood.

[6] Friedman DL, Chen L, Wolden S (2014). Dose-intensive response-based chemotherapy and radiation therapy for children and adolescents with newly diagnosed intermediate-risk hodgkin lymphoma: a report from the Children's Oncology Group Study AHOD0031. J Clin Oncol.

[7] Bhatia S, Robison LL, Oberlin O (1996). Breast cancer and other second neoplasms after childhood Hodgkin's disease. N Engl J Med.

[8] Schellong G, Riepenhausen M, Ehlert K (2014). Breast cancer in young women after treatment for Hodgkin's disease during childhood or adolescence: an observational study with up to 33-year follow-up. Dtsch Arztebl Int.

[9] van Nimwegen FA, Ntentas G, Darby SC (2017). Risk of heart failure in survivors of Hodgkin lymphoma: effects of cardiac exposure to radiation and anthracyclines. Blood.

[10] Hasenclever D (2002). The disappearance of prognostic factors in Hodgkin's disease. Ann Oncol.

[11] Mauz-Korholz C, Hasenclever D, Dorffel W (2010). Procarbazine-free OEPA-COPDAC chemotherapy in boys and standard OPPA-COPP in girls have comparable effectiveness in pediatric Hodgkin's lymphoma: the GPOH-HD-2002 study. J Clin Oncol.

[12] Brämswig JH, Heimes U, Heiermann E, Schlegel W, Nieschlag E, Schellong G (1990). The effects of different cumulative doses of chemotherapy on testicular function. Results in 75 patients treated for Hodgkin's disease during childhood or adolescence. Cancer.

[13] Hassel JU, Brämswig JH, Schlegel W, Schellong G (1991). Testicular function after OPA/COMP chemotherapy without procarbazine in boys with Hodgkin's disease. Results in 25 patients of the DAL-HD-85 study. Klin Padiatr.

[14] Sieniawski M, Reineke T, Josting A (2008). Assessment of male fertility in patients with Hodgkin's lymphoma treated in the German Hodgkin Study Group (GHSG) clinical trials. Ann Oncol.

[15] Behringer K, Mueller H, Goergen H (2013). Gonadal function and fertility in survivors after Hodgkin lymphoma treatment within the German Hodgkin Study Group HD13 to HD15 trials. J Clin Oncol.

[16] Trotti A, Byhardt R, Stetz J (2000). Common toxicity criteria: version 2.0. An improved reference for grading the acute effects of cancer treatment: impact on radiotherapy. Int J Radiat Oncol Biol Phys.

[17] Cheson BD, Fisher RI, Barrington SF (2014). Recommendations for initial evaluation, staging, and response assessment of Hodgkin and non-Hodgkin lymphoma: the Lugano classification. J Clin Oncol.

[18] WHO (2010).

[19] Kelsey TW, McConville L, Edgar AB (2017). Follicle stimulating hormone is an accurate predictor of azoospermia in childhood cancer survivors. PLoS One.

[20] Kelsey TW, Miles A, Mitchell RT (2016). A normative model of serum inhibin B in young males. PLoS One.

[21] Webber L, Davies M, Anderson R (2016). ESHRE guideline: management of women with premature ovarian insufficiency. Hum Reprod.

[22] Schellong GM (1989). The German cooperative therapy studies. An approach to minimize treatment modalities and invasive staging procedures. Cancer Treat Res.

[23] Engert A, Haverkamp H, Kobe C (2012). Reduced-intensity chemotherapy and PET-guided radiotherapy in patients with advanced stage Hodgkin's lymphoma (HD15 trial): a randomised, open-label, phase 3 non-inferiority trial. Lancet.

[24] Radford J, Illidge T, Counsell N (2015). Results of a trial of PET-directed therapy for early-stage Hodgkin's lymphoma. N Engl J Med.

[25] Borchmann P, Goergen H, Kobe C (2018). PET-guided treatment in patients with advanced-stage Hodgkin's lymphoma (HD18): final results of an open-label, international, randomised phase 3 trial by the German Hodgkin Study Group. Lancet.

[26] Barrington SF, Kluge R (2017). FDG PET for therapy monitoring in Hodgkin and non-Hodgkin lymphomas. Eur J Nucl Med Mol Imaging.

[27] Mauz-Körholz C, Hasenclever D, Holzendorf V (2014). Feasibility of VECOPA: a dose-intensive chemotherapy regimen for children and adolescents with intermediate and advanced stage Hodgkin's lymphoma: results of the GPOH-HD-2002/VECOPA pilot trial. Leuk Lymphoma.

[28] Kelly KM, Cole PD, Pei Q (2019). Response-adapted therapy for the treatment of children with newly diagnosed high risk Hodgkin lymphoma (AHOD0831): a report from the Children's Oncology Group. Br J Haematol.

[29] Viviani S, Zinzani PL, Rambaldi A (2011). ABVD versus BEACOPP for Hodgkin's lymphoma when high-dose salvage is planned. N Engl J Med.

[30] http://Clinicaltrials.gov.

