# Correction to *Lancet Oncol* 2022; 23: 125–37

**DOI:** 10.1016/S1470-2045(22)00029-8

**Published:** 2022-02

*Mauz-Körholz C, Landman-Parker J, Balwierzet W, et al. Response-adapted omission of radiotherapy and comparison of consolidation chemotherapy in children and adolescents with intermediate-stage and advanced-stage classical Hodgkin lymphoma (EuroNet-PHL-C1): a titration study with an open-label, embedded, multinational, non-inferiority, randomised controlled trial.* Lancet Oncol *2022; **23:** 125–37*—In this Article, in the Procedures section of the Methods, “Radiotherapy was omitted in patients who responded adequately to consolidation chemotherapy” should read “Radiotherapy was omitted in patients who responded adequately to induction chemotherapy with two cycles of OEPA”. This correction has been made to the online version as of Jan 31, 2022.

