# Supplementary material for: Response-adapted omission of radiotherapy and comparison of consolidation chemotherapy in children and adolescents with intermediate-stage and advanced-stage classical Hodgkin lymphoma (EuroNet-PHL-C1): a titration study with an open-label, embedded, multinational, non-inferiority, randomised controlled trial
Source: Lancet Oncol. 2022 Jan;23(1):125–37. doi: 10.1016/S1470-2045(21)00470-8 (PMC8716340; doi:10.1016/S1470-2045(21)00470-8)
Supplement: Supplementary appendix [file mmc1.pdf]

THE LANCET
Oncology

Supplementary appendix

This appendix formed part of the original submission and has been peer reviewed. We post it as supplied by the authors.

Supplement to: Mauz-Körholz C, Landman-Parker J, Balwierz W, et al. Response-adapted omission of radiotherapy and comparison of consolidation chemotherapy in children and adolescents with intermediate-stage and advanced-stage classical Hodgkin lymphoma (EuroNet-PHL-C1): a titration study with an open-label, embedded, multinational, non-inferiority, randomised controlled trial. *Lancet Oncol* 2021; published online Dec 9. https://doi.org/10.1016/S1470-2045(21)00470-8.

Supplementary material

Response-adapted omission of radiotherapy and comparison of consolidation chemotherapy in children and adolescents with intermediate-stage and advanced-stage classical Hodgkin lymphoma (EuroNet-PHL-C1): a titration study with an open-label, embedded, multinational, non-inferiority, randomised controlled trial

Christine Mauz-Körholz, Judith Landman-Parker*, Walentyna Balwierz*, Roland A Ammann, Richard A Anderson, Andische Attarbaschi, Jörg M Bartelt, Auke Beishuizen, Sabah Boudjemaa, Michaela Cepelova, Alexander Claviez, Stephen Daw, Karin Dieckmann, Ana Fernández-Teijeiro, Alexander Fosså, Stefan Gattenlöhner, Thomas Georgi, Lisa L Hjalgrim, Andrea Hraskova, Jonas Karlén, Regine Kluge, Lars Kurch, Thiery Leblanc, Georg Mann, Francoise Montravers, Jean Pears, Tanja Pelz, Vladan Rajić, Alan D Ramsay, Dietrich Stoevesandt, Anne Uyttebroeck, Dirk Vordermark, Dieter Körholz†, Dirk Hasenclever†, William Hamish Wallace†*

Joint first* and last† authors contributed equally

Wallace WH, hamish.wallace@ed.ac.uk, corresponding author

Körholz D, International coordinating chairperson

(Funded by Deutsche Krebshilfe e.V. and others; ClinicalTrials.gov number NCT00433459). Funding in France PHRC-K n°07235-P070105. Funding in UK CRUK.

1. Study design overview chart

Figure S1: Study Design

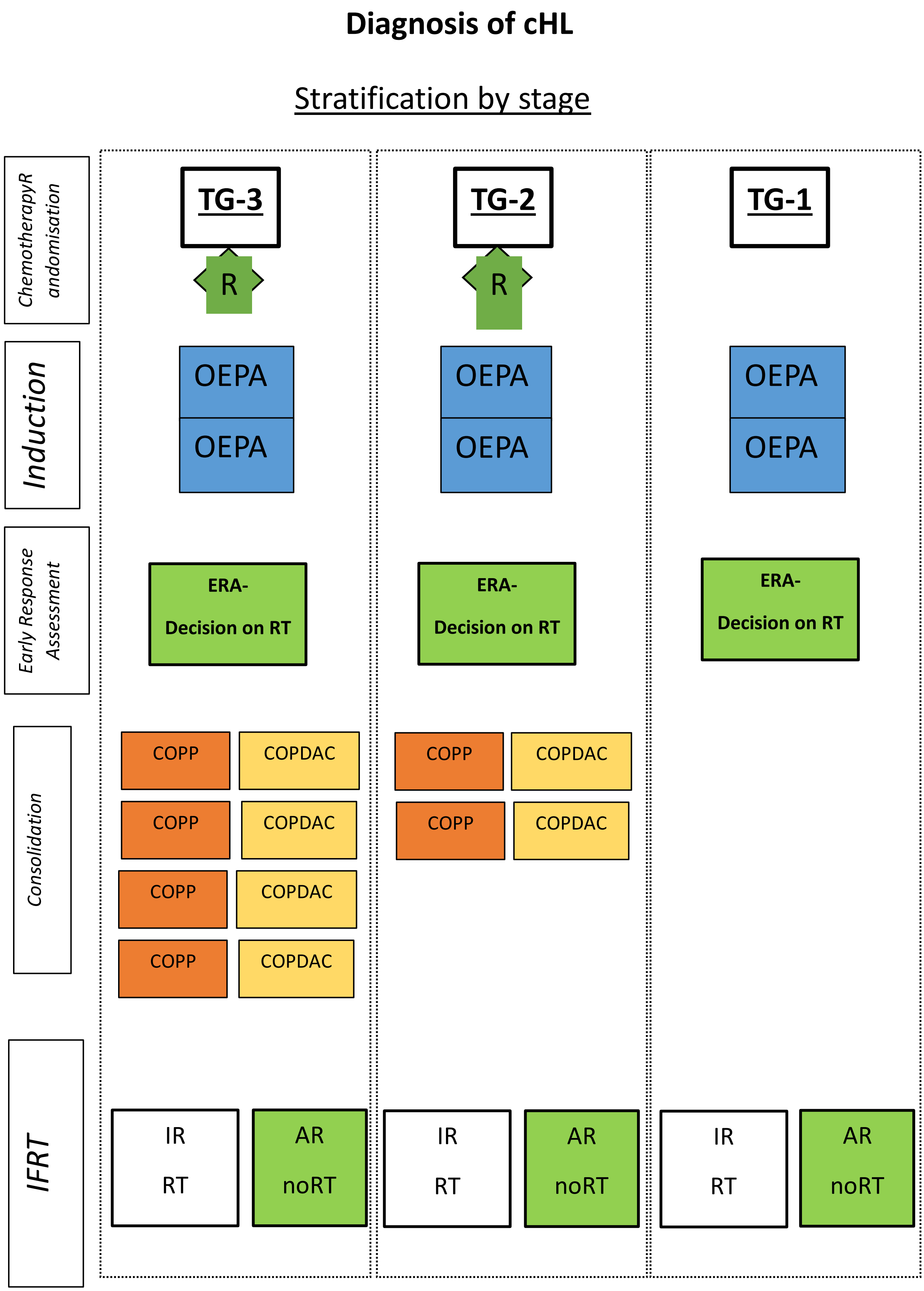

2. Selection of trial subjects for EuroNet-PHL-C1

2.1 Inclusion criteria
Trial sites register patients at their corresponding study office if the following requirements are met:
• diagnosis of classic Hodgkin's lymphoma
• patient aged under 18 years at time of diagnosis
• written informed consent of the patient and/or the patient's parents or guardian according to national laws
Country-specifically very young patients may be excluded to comply with national laws or formal insurance requirements.

2.2 Exclusion Criteria
Patients with one of the following circumstances are excluded:
• pre-treatment of Hodgkin's lymphoma differing from study protocol (except recommended pre-phase therapy of a large mediastinal tumour)
• known hypersensitivity or contraindication to study drugs
• diagnosis of lymphocyte predominant Hodgkin's lymphoma
• prior chemotherapy or radiotherapy
• other (simultaneous) malignancies
• pregnancy and / or lactation
• females who are sexually active refusing to use effective contraception (oral contraception, intrauterine devices, barrier method of contraception in conjunction with spermicidal jelly or surgical sterile)
• Current or recent (within 30 days prior to start of trial treatment) treatment with another investigational drug or participation in another investigational trial
• severe concomitant diseases (e.g. immune deficiency syndrome)
• known HIV positivity

2.3 Subsequent exclusion of patients
Patients are excluded from the study **after registration** if:
• documents or material ascertained before study inclusion show that an exclusion criterion was fulfilled or an inclusion criterion was not met
• the patient and/or the patient's parents or guardian withdraw(s) his/her/their consent to further study participation
If the diagnosis of Hodgkin's lymphoma is not confirmed by the reference pathologist the patient is still documented in the scope of the study. Patient's physician decides then together with the patient/parents or guardian on the further therapy.
The study chairpersons decide on the exclusion together with the biometrician of the study. A trial site may request the subsequent exclusion of a patient by sending a written detailed account of the reasons to the study office.
Subsequent exclusion of a patient differs from an individual therapy withdrawal. In the latter case the treatment of the patient according to protocol is terminated, but follow-up and documentation (data collection in the CRF) is continued according to protocol and the patient appears in all relevant analyses.

Further specifications
The rules in the study protocol needed further specification.

We excluded all patients in whom reference pathology positively confirmed that the patient did not have classic Hodgkin lymphoma. It proved unfeasible to document such patients within the study, in particular when the change in diagnosis was detected before or early during treatment.

We did not exclude patients in whom no reference pathology was performed or in whom reference pathology was inconclusive, because general reference pathology confirmed classic Hodgkin lymphoma in a very high proportion of cases.
We also excluded patients who withdrew consent before start of treatment. These cases are completely uninformative.
We excluded patients for formal reasons if no valid informed consent was obtained.

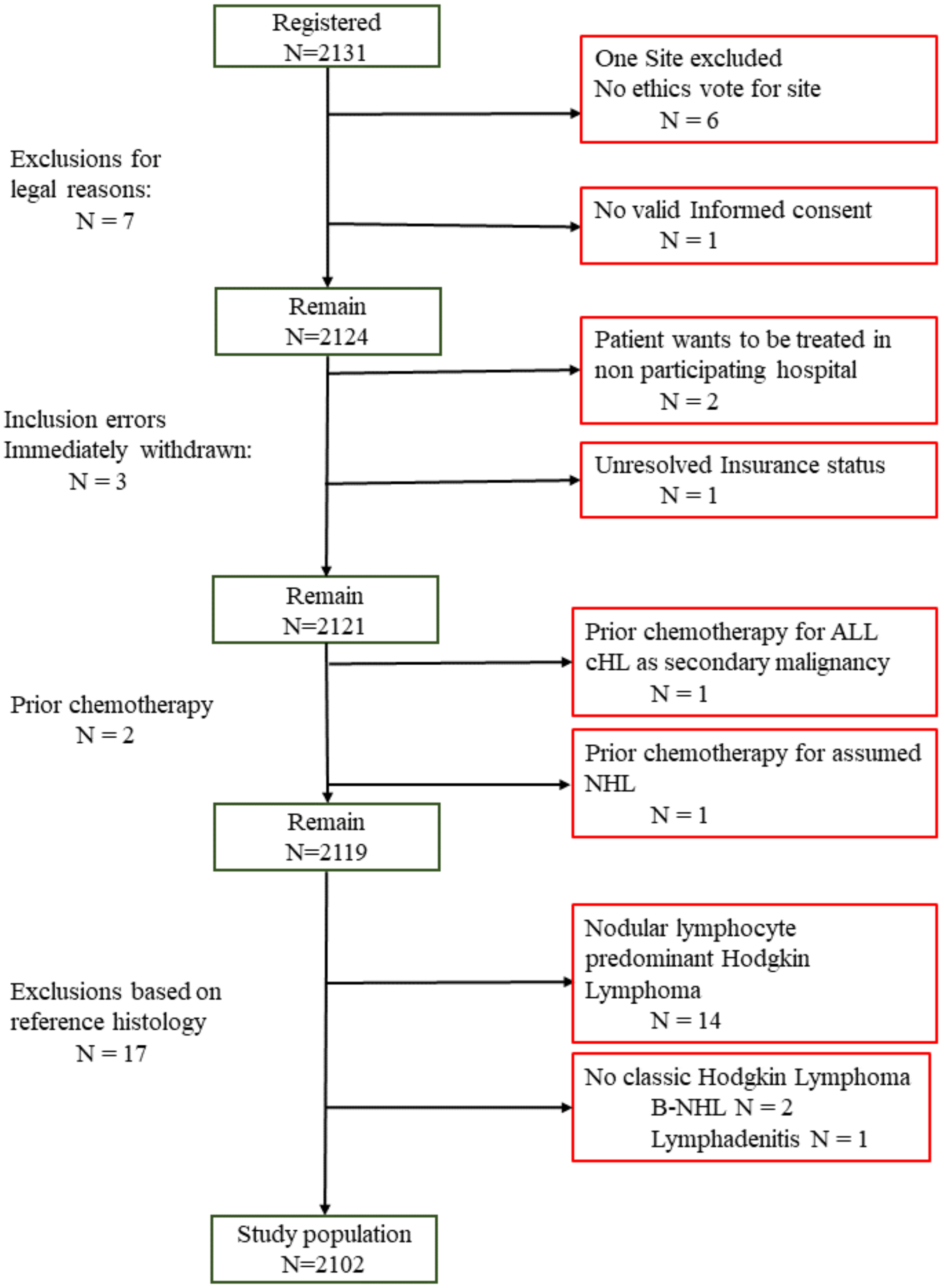

Figure S2: Disposition of all registered patients to achieve the overall study population

Already registered patients were excluded from the study population, if exclusion criteria were present or became apparent during the course of the study or if inclusion criteria were subsequently violated. Six randomised patients were among the exclusions: 3 in the COPP and 3 in the COPDAC arm. Five patients were retrospectively excluded, since the treating rial site was not accredited by their local IRB. One patient was retrospectively diagnosed as having mature B non-Hodgkin lymphoma.

3. Trial sites

Table S1. List of 180 Trial sites and respective principal investigators that contributed the N= 1365 patients reported in the TG2+3 publication

Trial site	Country	# Patients TG23	Name of	principal investigator
UCLH London	GB	41	Stephen	Daw
Klinik und Poliklinik für Kinder- und Jugendmedizin Münster	DE	31	Heribert	Jürgens
Our Lady's Children's Hospital. Dublin	IE	30	Anne	O'Meara
Faculty Hospital Motol Praha	CZ	28	Michaela Cepelova	
Institut d'hématologie et d'oncologie pédiatrique - IHOP, Lyon	FR	25	Mathias	Schell
CHU de Bordeaux - Hopital Pellegrin	FR	24	Yves	Perel
Institut Gustave Roussy, Villejuif	FR	23	Odile	Oberlin
Universitäts-Kinderklinik Essen	DE	20	Bernhard	Kremens
Royal Marsden Hospital, Sutton	GB	20	Mary	Taj
Hopital Armand Trousseau, Paris	FR	20	Judith	Landman-Parker
Yorkshire Regional Centre for Paediatric Oncology, Leeds General Infirmary, Leeds Teaching Hospitals (NHS Trust)	GB	19	Sue	Picton
Bristol Royal Hospital for Children	GB	19	Stephen	Lowis
Klinikum Augsburg, I. Klinik für Kinder und Jugendliche	DE	17	Astrid	Gnekow
CHU de Poitiers	FR	17	Frédéric	Millot
CHRU de Lille Hopital Jeanne de Flandre	FR	16	Anne	Lambilliotte
CHU de Toulouse - Hopital des Enfants	FR	16	Alain	Robert

Rikshospitalet HF Oslo	NO	16	Alexander	Fosså
Institut Curie, Paris	FR	15	Hélène	Pacquement
Universitäts-Kinderklinik Düsseldorf	DE	14	Gisela Maria	Janßen
Universitäts-Kinderklinik III Frankfurt/Main	DE	16	Thomas	Klingebiel
Kinderklinik Oldenburg gGmbH	DE	14	Hermann	Müller
Universitätsklinik für Kinder- und Jugendmedizin Heidelberg	DE	14	Wolfgang	Behnisch
Kinderklinik und Poliklinik im Dr. med. von Haunerschen Kinderspital München	DE	14	Irene	Schmid
Universitätskinderklinik zu Köln	DE	14	Frank	Berthold
Hopital d'Enfants La Timone, Marseille	FR	14	Gérard	Michel
CHU de Strasbourg - Hopital de Hautepierre	FR	14	Patrick	Lutz
Wroclaw Medical University Wroclaw	PL	14	Radoslaw	Chaber
Hopital Robert Debré, Paris	FR	13	André	Baruchel
Kinderspital Zürich	CH	14	Eva	Bergsträsser
Klinikum Stuttgart Olgahospital	DE	13	Stefan	Bielack
CHU de Rennes - Hopital Sud	FR	13	Virginie	Gandemer
St. Anna Kinderspital Wien	AT	12	Andishe	Attarbaschi
Charité - Campus Virchow-Klinikum Berlin	DE	12	Rüdiger	Fengler
Universitätskinderklinik Kiel	DE	12	Alexander	Claviez
Universitätskinderklinik Hamburg-Eppendorf	DE	12	Gabriele	Escherich
Universitätsklinikum Ulm	DE	12	Klaus Michael	Debatin
Universitätskinderklinik der TU Dresden	DE	12	Meinolf	Suttorp
CHU de Nancy - Hopital d'Enfants de Brabois	FR	12	Claudine	Schmitt
Addenbrooke's Hospital, Cambridge	GB	12	Amos	Burke

Medizinische Hochschule Hannover	DE	12	Karl- Walter	Sykora
Universitäts-Kinderklinik Freiburg	DE	11	Charlotte	Niemeyer
Hospital Niño Jesús Madrid	ES	11	David	Ruano Dominguez
CHRU de Tours - Centre Pédiatrique Gatien de Clocheville	FR	11	Odile	Lejars
Alder Hey Children's NHS Foundation Trust, Liverpool	GB	11	Lisa	Howell
Sir James Spence Institute, Royal Victoria Infirmary, Gt North Children's Hospital, Newcastle-upon-Tyne	GB	11	Kevin	Windebank
Jagiellonian University Medical College Krakow	PL	11	Angelina	Moryl-Bujakowska
Medical University Lublin	PL	11	Malgorzata	Mitura-Lesiuk
Landeskinderklinik Linz	AT	10	Georg	Ebetsberger
Universitäts-Klinik für Kinder und Jugendliche Erlangen	DE	10	Wolfgang Stephan	Holter
Hospital Infantil Vall d´Hebron Barcelona	ES	10	Constantino	Sabado
Oxford Children's Hospital, John Radcliffe Hospital	GB	10	Georgina	Hall
Southampton General Hospital	GB	10	Juliet	Gray
The Queen Silvia children`s Hospital, Göteborg University	SE	10	Marianne	Jarfelt
CHU de Nantes - Hopital Mère Enfant	FR	9	Françoise	Mechinaud
Manchester Christie Hospital	GB	9	Bernadette	Brennan
Universitäts-Kinderklinik Mainz	DE	9	Helmut-Matthias	Dittrich
Universitäts-Kinderklinik Giessen	DE	9	Alfred	Reiter
Universitäts-Kinderklinik Würzburg	DE	9	Paul-Gerhardt	Schlegel
CHU d'Angers	FR	9	Xavier	Rialland
CHU de Grenoble	FR	9	Dominique	Plantaz
CHU de Reims Hopital Américain	FR	9	Martine	Munzer

Glasgow Royal Hospital for Sick Children	GB	9	Elizabeth	Chalmers
Royal Manchester Children's Hospital	GB	9	Bernadette	Brennan
Erasmus MC - Sophia Children's Hospital University Medical Center Rotterdam	NL	9	Auke	Beishuizen
Astrid Lindgren Children's Hospital Stockholm	SE	9	Jonas	Karlen
Kinderklinik und Poliklinik der TU München - Kinderklinik München Schwabing	DE	9	Angela	Wawer
Universitätskinderklinik Graz	AT	8	Christian	Urban
Universitätsklinikum Regensburg	DE	8	Selim	Corbacioglu
Children's Faculty Hospital Bratislava	SK	8	Andrea	Hraskova
Universitätsklinik für Kinder- und Jugendmedizin Homburg	DE	8	Norbert	Graf
Klinik für Kinder- und Jugendmedizin Lübeck	DE	8	Melchior	Lauten
Evangelisches Krankenhaus Bielefeld	DE	8	Norbert	Jorch
CHU de Nice - Hopital Archet 2	FR	8	Marilyne	Poirée
University Medical Center Nijmegen	NL	8	P. M.	Hoogerbrugge
University Hospital Lund	SE	8	Ingrid	Öra
CHU de Besançon - Hopital Saint Jacques	FR	8	Emmanuel	Plouvier
CHU Caen	FR	8	Patrick	Boutard
Landeskliniken Salzburg	AT	7	Gabriele	Dobrovoljski
Universitäts-Kinderklinik Bonn	DE	7	Olga	Moser
Helios Klinikum Berlin-Buch	DE	7	Lothar	Schweigerer
Universitäts-Kinderklinik Magdeburg	DE	7	Peter	Vorwerk

Hospital universitario La Fe Valencia	ES	7	Jose Maria	Fernández
CHRU de Montpellier - Hopital Arnaud de Villeneuve	FR	7	Nicolas	Sirvent
Children´s Faculty Hospital Banská Bystrica	SK	7	Eva	Bubanska
Professor-Hess-Kinderklinik Bremen	DE	6	Arnulf	Pekrun
Universitätsklinik für Kinderheilkunde und Jugendmedizin Tübingen	DE	6	Martin	Ebinger
Universitäts-Kinderklinik Aachen	DE	6	Rolf	Mertens
Rigshospitalet, University Kopenhagen	DK	6	Harald Thomassen	
Hospital 12 de octubre Madrid	ES	6	Jose Luis	Vivanco
Hospital Clinico Universitario de Santiago de Compostela	ES	6	Jose Miguel	Couselo Sanchez
CHU de Rouen	FR	6	Jean Pierre	Vannier
CHU Brest	FR	6	Philippe	Lemoine
Great Ormond Street Hospital, London	GB	6	Antony	Michalski
Universitätskinderklinik Halle	DE	6	Dieter	Körholz
University Hospital Gent	BE	5	Genevieve	Laureys
Kinderspital Luzern	CH	5	Johannes	Rischewski
Vestische Kinder- und Jugendklinik Datteln	DE	5	Thomas	Wiesel
Klinikum Braunschweig	DE	5	Wolfgang	Eberl
Universitäts-Kinderklinik Göttingen	DE	5	Max	Lakomek
CHU d'Amiens - Hopital Nord	FR	5	Brigitte	Pautard
CHRU de Clermont Ferrand Hopital Hotel-Dieu	FR	5	François	Demeocq
Institut de Cancérologie de la Loire	FR	5	Jean Louis	Stephan
Medical University Gdansk	PL	5	Teresa	Stachowicz-Stencel
Hopital Saint-Louis	FR	5	Thierry	Leblanc

Children´s Faculty Hospital Kosice	SK	5	Irina	Oravkinova
Universitätskinderklinik Mannheim	DE	4	Matthias	Dürken
Leicester Royal Infirmary, Children's Hospital	GB	4	Kaljit	Bhuller
UZ Leuven	BE	4	Anne	Uyttebroeck
Städt. Klinikum Kemperhof Koblenz	DE	4	Rudolf	Ferrari
Klinikum Dortmund gGmbH	DE	4	Dominik	Schneider
Klinikum Bayreuth GmbH	DE	4	Thomas	Rupprecht
HELIOS Klinikum Erfurt/Klinik für Kinder- und Jugendmedizin	DE	4	Axel	Sauerbrey
Universitätsklinik und Poliklinik für Kinder und Jugendliche Leipzig	DE	4	Holger	Christiansen
Hospital General Universitario Gregorio Marañón Madrid	ES	4	Carmen	Garrido
Haukeland Universitetssykehus Bergen	NO	4	Mikael	Donnér
Children's Hospital of Chorzów	PL	4	Maria	Wieczorek
Children's Memorial Health Institute Warsaw - Pomnik-Centrum Zdrowia Dziecka	PL	4	Danuta	Perek
Children's University Hospital Uppsala	SE	4	Gustaf	Ljungman
Royal Aberdeen Children's Hospital	GB	4	Hugh	Bishop
CHU de Dijon Hopital du Bocage	FR	3	Gérard	Couillault
Hôpital Universitaire des Enfants Reine Fabiola (ULB), Bruxelles	BE	3	Christine	Devalck
Universitäts-Kinderspital Beider Basel	CH	3	Thomas	Kühne
Universitätsklinik für Kinderheilkunde - Inselspital Bern	CH	3	Kurt	Leibundgut
CHUV-Départment de pédiatrie, Lausanne	CH	3	Nicolas	von der Weid
Kantonspital Aarau AG	CH	3	Regula	Angst
Faculty Hospital Olomouc	CZ	3	Zbynek	Novak

Diakonie Neuendettelsau Nürnberg	DE	3	Wolfram	Scheurlen
Klinikum Chemnitz gGmbH	DE	3	Andre	Hofmann
Hospital Cruces Bilbao	ES	3	Aizpea	Echebarria
HOSPITAL VIRGEN DEL CAMINO Pamplona	ES	3	Maria	Sagaseta de Ilurdoz Uranga
Hospital Universitario Miguel Servet Zaragoza	ES	3	Ana	Carbone
Hopital d'Enfants La Timone	FR	3	Carole	Coze
Edinburgh Royal Hospital for Sick Children	GB	3	Hamish	Wallace
Nicolaus Copernicus University Collegium Medicum Bydgoszcz	PL	3	Andrzej	Koltan
Helios Klinikum Krefeld	DE	3	Thomas	Imschweiler
Universitätskinderklinik Jena	DE	2	Bernd	Gruhn
Cliniques Universitaires Saint-Luc (UCL), Bruxelles	BE	2	Christiane	Vermylen
Kinderklinik am städtischen Klinikum Karlsruhe	DE	2	Alfred	Leipold
Kinderkrankenhaus Köln-Rhiel	DE	2	Aram	Prokop
Klinikum Kassel GmbH	DE	2	Martina	Rodehüser
SLK Kliniken Heilbronn	DE	2	Hermann	Full
Gemeinschaftskrankenhaus Herdecke	DE	2	Alfred	Längler
Carl-Thiem-Klinikum Cottbus	DE	2	Elisabeth	Holfeld
Universitätsklinik für Kinder- und Jugendmedizin Greifswald	DE	2	Holger	Lode
Universitäts-Kinder- und Jugendklinik Rostock	DE	2	Carl Friedrich	Classen
Hospital Sant Joan de Deu Barcelona	ES	2	Ofelia	Cruz
Hospital Universitario Son Espases Palma de Mallorca	ES	2	Mercedes	Guibelalde
Universitetssykehuset i Nord-Norge Tromsø	NO	2	Tore	Stokland

Ullevål Universitetssykehus Oslo	NO	2	Eva	Widing
Emma Children's Hospital/ AMC, Amsterdam	NL	2	H.N.	Caron
Warsaw Medical University	PL	2	Barbara	Sopylo
Silesian Academy of Medicine Zabrze	PL	2	Tomasz	Szczepanski
Medical University Bialystok	PL	2	Katarzyna	Muszynska-Roslan
Medical University Lodz	PL	2	Aleksandra	Jasinska
Children's Hospital of Kielce	PL	2	Grazyna	Karolczyk
University Hospital Linköping	SE	2	Mikael	Behrendtz
University Children's Hospital Ljubljana	SI	2	Vladan	Rajic
Landeskrankenhaus Klagenfurt	AT	2	Barbara	Jauk
Beatrix children's hospital University Medical Center Groningen	NL	2	E.S.J.M.	de Bont
Johannes Wesling Klinikum Minden	DE	1	Bernhard	Erdlenbruch
CHU de Limoges Hopital Mère-Enfant	FR	1	Christophe	Piguet
Steiermärkische Krankenanstaltenges, Leoben	AT	1	Reinhard	Moser
Klinik für Kinder- und Jugendheilkunde Innsbruck	AT	1	Bernhard	Meister
Asklepios Klinik St. Augustin	DE	1	Harald	Reinhard
Mutterhaus der Borromäerinnen Trier	DE	1	Isa	Feddersen
Klinikum der Stadt Wolfsburg	DE	1	Sally	Mukodzi
Helios Kliniken Schwerin	DE	1	Matthias	Kasbohm
Klinikum Lippe Detmold	DE	1	Hans	Broede
Odense Universitetshospital	DK	1	Eckhard	Schomerus
Århus Universitetshospital	DK	1	Pernille	Wendlandt
Hospital Torrecardenas de Almería	ES	1	Maria	Angeles Vazquez

Hospital Universitario Virgen de las Nieves Granada	ES	1	Maria	José Ortega
Hospital Las Palmas	ES	1	Antonio	Molines
Hospital Materno-Infantil Carlos-Haya Malaga	ES	1	Antonio	Herrero Hernandez
Hospital Central Universitario Asturias Oviedo	ES	1	Maria	Galbe
Hospital Universitario Virgen Macarena Sevilla	ES	1	Ana	Fernández-Teijeiro
Hospital Virgen de la Arrixaca, El Palmar (Murcia)	ES	1	Mar	Bermúdez
University Hospital Trondheim	NO	1	Erling	Moe
VU University Medical Center, Amsterdam	NL	1	G.J.L.	Kaspers
Umea University Hospital	SE	1	Ulf	Hjalmars

4. Demographic data for non-randomised per protocol patients compared to randomised per protocol patients

Table S2. Demographic data for non-randomised per protocol patients compared to randomised per protocol patients

		**COPP Rando mised**		**COPDAC rando-mised**		**COPP chosen**		**COPDAC Chosen**		**COPDAC asigned**	
Variable		ALL	%	ALL	%	ALL	%	ALL	%	ALL	%
Total numbers		444	100	448	100	65	100	82	100	248	100
Age	Over 13	317	71.4	335	74.8	50	76.9	61	74.4	171	69
	Under 13	127	28.6	113	25.2	15	23.1	21	25.6	77	31
Sex	female	244	55	250	55.8	30	46.2	58	70.7	135	54.4
	male	200	45	198	44.2	35	53.8	24	29.3	113	45.6
Combined stage*	IIA	1	0.2	0	0	2	3.1	0	0	0	0
	IIAE	30	6.8	36	8	4	6.2	6	7.3	17	6.9
	IIB	73	16.4	67	15	2	3.1	13	15.9	39	15.7
	IIBE	49	11	36	8	7	10.8	5	6.1	17	6.9
	IIIA	56	12.6	56	12.5	12	18.5	8	9.8	25	10.1
	IIIAE	8	1.8	12	2.7	2	3.1	1	1.2	7	2.8
	IIIB	51	11.5	46	10.3	6	9.2	14	17.1	31	12.5
	IIIBE	20	4.5	21	4.7	1	1,5	1	1.2	11	4.4
	IVA	49	11	63	14.1	6	9.2	15	18.3	34	13.7
	IVAE	17	3.8	9	2	5	7.7	3	3.7	10	4
	IVB	61	13.7	69	15.4	13	20	11	13.4	35	14.1
	IVBE	29	6.5	33	7.4	5	7.7	5	6.1	22	8.9
Ann Arbor stage	II	153	34.5	139	31	15	23.1	24	29.3	73	29.4
	III	135	30.4	135	30.1	21	32.3	24	29.3	74	29.8
	IV	156	35.1	174	38.8	29	44.6	34	41.5	101	40.7
B Symptoms	No	161	36.3	176	39.3	31	47.7	33	40.2	93	37.5
	Yes	283	63.7	272	60.7	34	52.3	49	59.8	155	62.5
Bulky disease*	No	222	52.5	207	48.4	38	59.4	47	57.3	121	50.4
	Yes	201	47.5	221	51.6	26	40.6	35	42.7	119	49.6
RT assigned	No RT	189	42.6	160	35.7	20	30.8	38	46.3	107	43.1
	RT	255	57.4	288	64.3	45	69.2	44	53.7	141	56.9

*Combined stage = Ann Arbor stage including B-Symptoms and E-stage

§Bulky disease= defined as contiguous tumour volume >/= 200 ml

5. Chemotherapy delivery and Toxicity

5.1 Percentiles of delivered chemotherapy doses

Percentiles	5%	10%	50%	90%	95%
OEPA					
Vincistine(%)	97.0	99.7	100.0	101.2	103.0
Etoposide(%)	96.0	98.1	100.0	104.0	106.7
Prednisone(%)	92.6	95.5	100.0	104.2	106.9
Doxorubicin(%)	96.7	98.2	100.0	103.1	104.5
COPP					
Cyclophosphamide(%)	96.6	98.6	100.0	102.8	104.3
Vincistine(%)	90.0	99.4	100.0	100.0	102.6
Prednisone(%)	92.6	93.8	100.0	104.2	107.1
Procarbazine(%)	84.7	92.6	100.0	105.3	107.1
COPDAC					
Cyclophosphamide(%)	95.8	98.2	100.0	102.9	104.5
Vincistine(%)	96.3	100.0	100.0	100.0	102.6
Prednisone(%)	93.2	93.8	100.0	104.8	107.1
Dacarbazine(%)	95.8	97.8	100.0	102.9	104.4

Table S3. Percentiles of dose given in percent of the planned dose for cytostatic drugs used in OEPA, COPP and COPDAC. More than 90% of all chemotherapy cycles (OEPA, COPP, COPDAC) were administered with more than 90% of the prescribed drug doses.

5.2 Severe toxicities/SAEs and consecutive protocol deviations

Pat ID	Treatment group	Pt. included in PP analysis	Randomi-sation y/n, Randoarm	Scheduled treatment	Actual/docu-mented treatment delivery	Documented reason for treatment deviation
2260	TG-3	yes	No	2 OEPA<br>4 COPP	OEPA-OEPA-COPP-ABVD-<br>No RT	Psychosis
2349	TG-3	yes	Yes,<br>COPDAC	2 OEPA<br>4 COPDAC	OEPA-OEPA-COPDAC-ABVD-ABVD-RT	Psychosis
2530	TG-3	yes	No	2 OEPA<br>4 COPP	OEPA-OEPA-ABVD-COPP-ABVD-ICE-RT	Psychosis
2861	TG-3	yes	Yes,<br>COPP	2 OEPA<br>4 COPP	OEPA-OEPA-COPP-SAE in second COPP<br>Treatment unclear<br>No RT	Allergy, septic shock
5243	TG3	Yes	Yes<br>COPDAC	2 OEPA<br>4 COPDAC	OEPA-OEPA-COPDAC-COPDAC-COPDAC-ABVD-RT	Osteoporosis after 5th COPDAC
5370	TG-2	No	No	2 OEPA<br>2 COPDAC	OEPA- death	Sepsis
5411	TG-3	yes	No	2 OEPA<br>4 COPDAC	OEPA-COPDAC-OEPA-OEPA-COPDAC-COPDAC-No RT	SAE (not otherwise specified)

Table S4 Documented severe protocol deviations in consequence to severe toxicities/severe adverse events (SAEs)

Seven patients deviated from the assigned chemotherapy treatment sequence in reaction to severe adverse events. These patients were still included in the PP analysis. In detail, three patients developed psychosis, which was deemed steroid-induced, were switched to a non-steroid containing chemotherapy, e. g. ABVD or ICE +/-RT. One patient with osteoporosis, which was also deemed steroid-induced, changed to ABVD for the last of four consolidation cycles and got RT. In one patient with allergy and septic shock in the fourth of six scheduled chemotherapy cycles the consecutive treatment (neither chemotherapy nor RT) was not documented. In one Patient, scheduled for 2 x OEPA and 4 x COPDAC, three cycles of each were given without RT due to an SAE, which was not further specified. One patient scheduled for 2 x OEPA and 2 x COPDAC developed neutropenic sepsis after the 1st OEPA cycle and died.

Table S5A. OEPA-Toxicities

**Body System**	**Preferred Term**	**Total number**	**CTC AE 1**	**CTC AE 2**	**CTC AE 3**	**CTC AE 4**
Cardiac disorders	Arrhythmia	2	1	0	1	0
Cardiac disorders	Intracardiac thrombus	1	0	0	1	0
Cardiac disorders	Tachycardia	3	1	1	1	0
Eye disorders	Blepharitis	1	0	0	1	0
Eye disorders	Eyelid ptosis	1	0	0	1	0
Gastrointestinal disorders	Abdominal pain	36	17	18	1	0
Gastrointestinal disorders	Abdominal pain upper	14	10	2	2	0
Gastrointestinal disorders	Colitis	2	0	0	1	1
Gastrointestinal disorders	Gastritis	4	2	1	1	0
Gastrointestinal disorders	Large intestine perforation	1	0	0	0	1
Gastrointestinal disorders	Nausea	54	37	16	1	0
General disorders and administration site conditions	Extravasation	4	2	1	1	0
General disorders and administration site conditions	Mucosal inflammation	4	0	3	1	0
General disorders and administration site conditions	Pain	30	12	12	6	0
Immune system disorders	Anaphylactic reaction	1	0	0	1	0
Immune system disorders	Anaphylactic shock	1	0	0	0	1
Immune system disorders	Drug hypersensitivity	4	0	2	1	1
Immune system disorders	Hypersensitivity	4	0	2	2	0
Infections and infestations	Herpes simplex	1	0	0	1	0
Infections and infestations	Pilonidal cyst	1	0	0	1	0
Infections and infestations	Pneumonia	1	0	0	1	0
Metabolism and nutrition disorders	Decreased appetite	5	3	1	1	0

Metabolism and nutrition disorders	Hyperglycaemia	7	3	2	2	0
Metabolism and nutrition disorders	Hypokalaemia	5	4	0	1	0
Metabolism and nutrition disorders	Hyponatraemia	3	2	0	1	0
Metabolism and nutrition disorders	Hypophosphataemia	19	12	5	2	0
Metabolism and nutrition disorders	Malnutrition	1	0	0	1	0
Musculoskeletal and connective tissue disorders	Arthralgia	18	4	9	4	0
Musculoskeletal and connective tissue disorders	Back pain	16	8	7	1	0
Musculoskeletal and connective tissue disorders	Bone pain	9	4	4	1	0
Musculoskeletal and connective tissue disorders	Musculoskeletal pain	11	1	8	2	0
Musculoskeletal and connective tissue disorders	Myopathy	1	0	0	1	0
Musculoskeletal and connective tissue disorders	Osteonecrosis	2	0	1	1	0
Musculoskeletal and connective tissue disorders	Pain in extremity	22	9	12	1	0
Musculoskeletal and connective tissue disorders	Pain in jaw	18	13	4	1	0
Musculoskeletal and connective tissue disorders	Spondylolysis	1	0	0	1	0
Nervous system disorders	Cerebral venous sinus thrombosis	1	0	0	1	0
Nervous system disorders	Coma	1	0	0	0	1
Nervous system disorders	Generalised tonic-clonic seizure	1	0	0	0	1
Nervous system disorders	Headache	24	13	9	2	0
Nervous system disorders	Neuralgia	6	2	1	3	0
Nervous system disorders	Superior sagittal sinus thrombosis	1	0	0	0	1

Nervous system disorders	Syncope	1	0	0	1	0
Psychiatric disorders	Bipolar disorder	1	0	0	1	0
Psychiatric disorders	Delirium	1	0	0	1	0
Respiratory, thoracic and mediastinal disorders	Lung disorder	1	0	0	0	1
Respiratory, thoracic and mediastinal disorders	Pneumothorax	1	0	0	0	1
Respiratory, thoracic and mediastinal disorders	Pulmonary embolism	4	0	0	2	2
Skin and subcutaneous tissue disorders	Alopecia	17	7	9	1	0
Skin and subcutaneous tissue disorders	Dermatitis	2	1	0	1	0
Surgical and medical procedures	Resuscitation	1	0	0	0	1
Vascular disorders	Deep vein thrombosis	3	0	1	2	0
Vascular disorders	Embolism	1	0	0	1	0
Vascular disorders	Hypovolaemic shock	1	0	0	0	1
Vascular disorders	Jugular vein thrombosis	1	0	0	1	0
Vascular disorders	Thrombosis	7	0	1	5	1
Vascular disorders	Venous thrombosis	2	0	0	0	2

Table S5B. COPP-Toxicities

Body System	Preferred Term	Total number	CTC AE 1	CTC AE 2	CTC AE 3	CTC AE 4
General disorders and administration site conditions	Mucosal inflammation	1	0	0	0	1
General disorders and administration site conditions	Pain	8	2	4	2	0
Hepatobiliary disorders	Cholelithiasis	1	0	0	1	0
Immune system disorders	Drug hypersensitivity	3	1	1	1	0
Immune system disorders	Hypersensitivity	2	1	0	1	0
Infections and infestations	Sepsis	1	0	0	1	0
Investigations	Blood sodium decreased	1	0	0	1	0
Investigations	Weight increased	8	4	3	1	0
Musculoskeletal and connective tissue disorders	Arthralgia	10	6	3	1	0
Musculoskeletal and connective tissue disorders	Back pain	5	2	1	2	0
Musculoskeletal and connective tissue disorders	Osteonecrosis	1	0	0	1	0
Nervous system disorders	Cerebral venous sinus thrombosis	1	0	0	0	1
Nervous system disorders	Seizure	1	0	0	1	0
Nervous system disorders	Syncope	1	0	0	1	0
Skin and subcutaneous tissue disorders	Skin disorder	3	1	1	1	0
Skin and subcutaneous tissue disorders	Urticaria	3	0	2	1	0
Vascular disorders	Deep vein thrombosis	2	0	1	1	0
Vascular disorders	Thrombosis	3	1	1	1	0

Table S5C. COPDAC-Toxicities

Body System	Preferred	Total number	CTC AE 1	CTC AE 2	CTC AE 3	CTC AE 4
Gastrointestinal disorders	Abdominal pain	10	7	2	1	0
Gastrointestinal disorders	Dysphagia	2	1	0	1	0
Gastrointestinal disorders	Gastrointestinal pain	3	1	1	1	0
General disorders and administration site conditions	Pain	14	6	4	4	0
Infections and infestations	Infection	1	0	0	1	0
Investigations	Pancreatic enzymes	1	0	0	1	0
Investigations	Weight increased	9	4	3	2	0
Metabolism and nutrition disorders	Hyperglycaemia	6	4	1	1	0
Metabolism and nutrition disorders	Hypocalcaemia	6	4	1	1	0
Metabolism and nutrition disorders	Hypokalaemia	7	5	0	1	0
Metabolism and nutrition disorders	Hyponatraemia	4	3	0	1	0
Musculoskeletal and connective tissue disorders	Arthralgia	12	5	5	2	0
Musculoskeletal and connective tissue disorders	Back pain	7	2	3	2	0
Musculoskeletal and connective tissue disorders	Bone infarction	1	0	0	1	0
Musculoskeletal and connective tissue disorders	Bone pain	7	4	2	1	0
Musculoskeletal and connective tissue disorders	Musculoskeletal pain	9	3	5	1	0
Musculoskeletal and connective tissue disorders	Osteonecrosis	4	1	0	3	0
Musculoskeletal and connective tissue disorders	Pain in extremity	19	9	8	2	0
Nervous system disorders	Headache	12	10	1	1	0
Nervous system disorders	Migraine	1	0	0	1	0
Nervous system disorders	Neuralgia	3	1	0	2	0
Nervous system disorders	Petit mal epilepsy	1	0	0	1	0
Nervous system disorders	Seizure	1	0	0	1	0
Psychiatric disorders	Delirium	1	0	0	1	0
Psychiatric disorders	Depression	4	0	3	1	0
Psychiatric disorders	Psychotic disorder	1	0	0	0	1

Respiratory, thoracic and mediastinal disorders	Pulmonary embolism	3	0	0	3	0
Skin and subcutaneous tissue disorders	Acne	5	1	3	1	0
Skin and subcutaneous tissue disorders	Alopecia	8	3	4	1	0
Skin and subcutaneous tissue disorders	Rash vesicular	1	0	0	1	0

5.3 Adherence to the chemotherapy time schedule

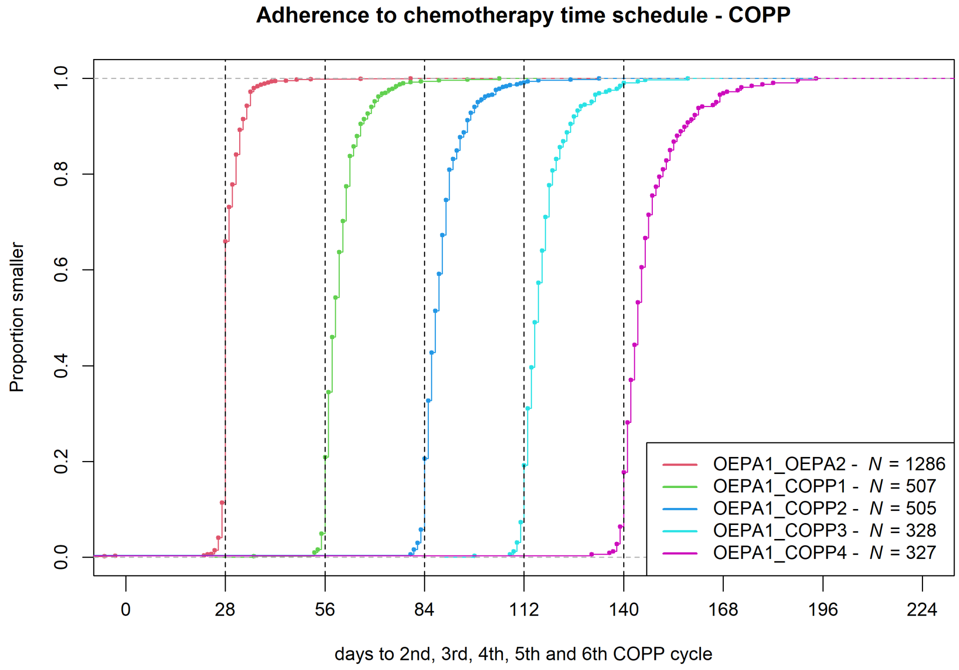

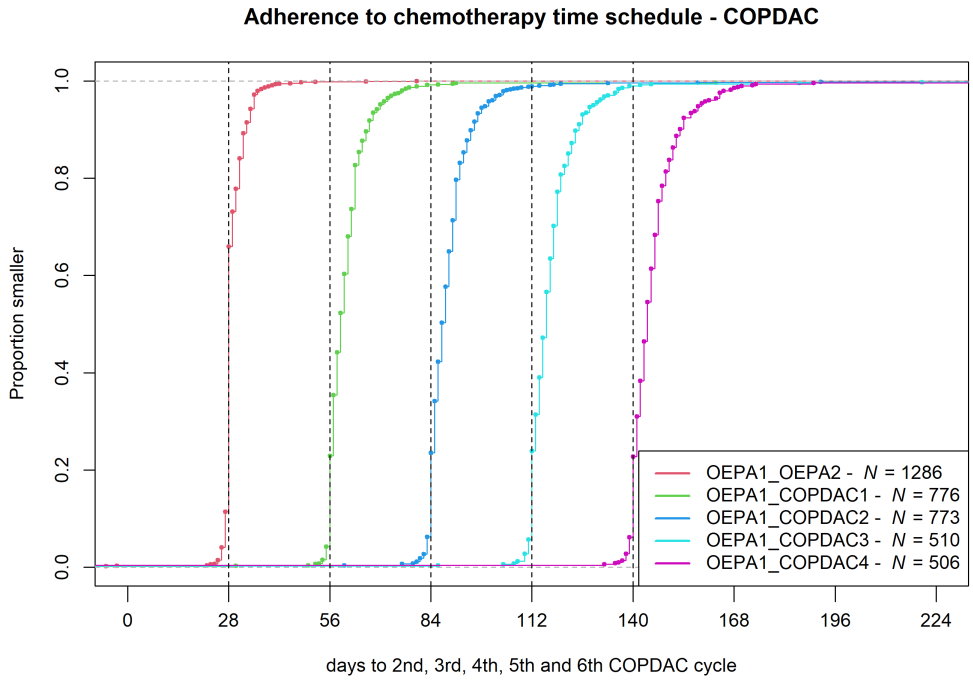

Figure S3: Cumulative distribution functions of time in days from the start of the first OEPA cycle to the start of subsequent cycles for COPP respective COPDAC.

6. Intention to treat analysis of EFS , PFS, and OS for the titration study and the embedded randomised trial

6.1 IIT EFS Titration study adequate versus inadequate response

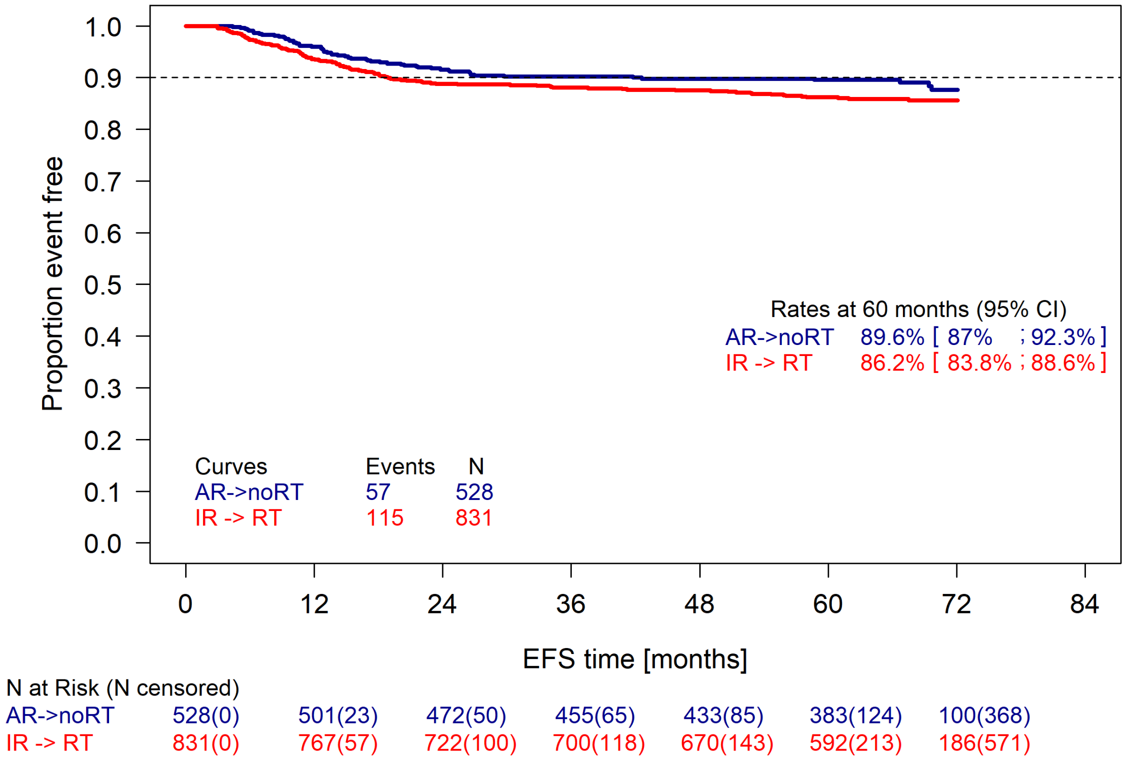

Figure S4A: Event-Free Survival in the intention to treat analysis of the titration trial showing patients with AR and no RT (blue) and patients with IR scheduled for RT (red). Note six patients without documented response at ERA are not included.

6.2 IIT PFS Titration study adequate versus inadequate response

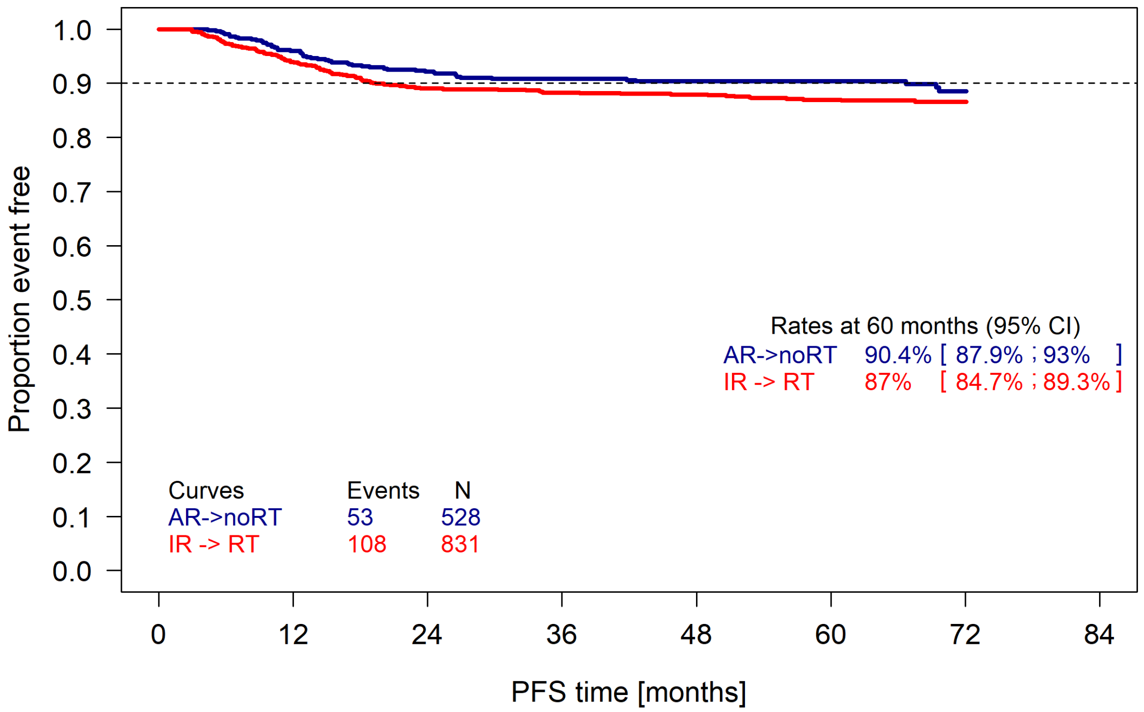

N at Risk (N censored)

AR->noRT	528(0)	501(23)	475(47)	458(62)	436(82)	387(121)	102(368)
IR -> RT	831(0)	770(54)	723(99)	701(118)	672(143)	596(212)	188(574)

Figure S4B: Progression-Free Survival in the intention to treat analysis of the titration trial showing patients with AR and no RT (blue) and patients with IR scheduled for RT (red). Note six patients without documented response at ERA are not included.

6.3 IIT OS Titration study adequate versus inadequate response

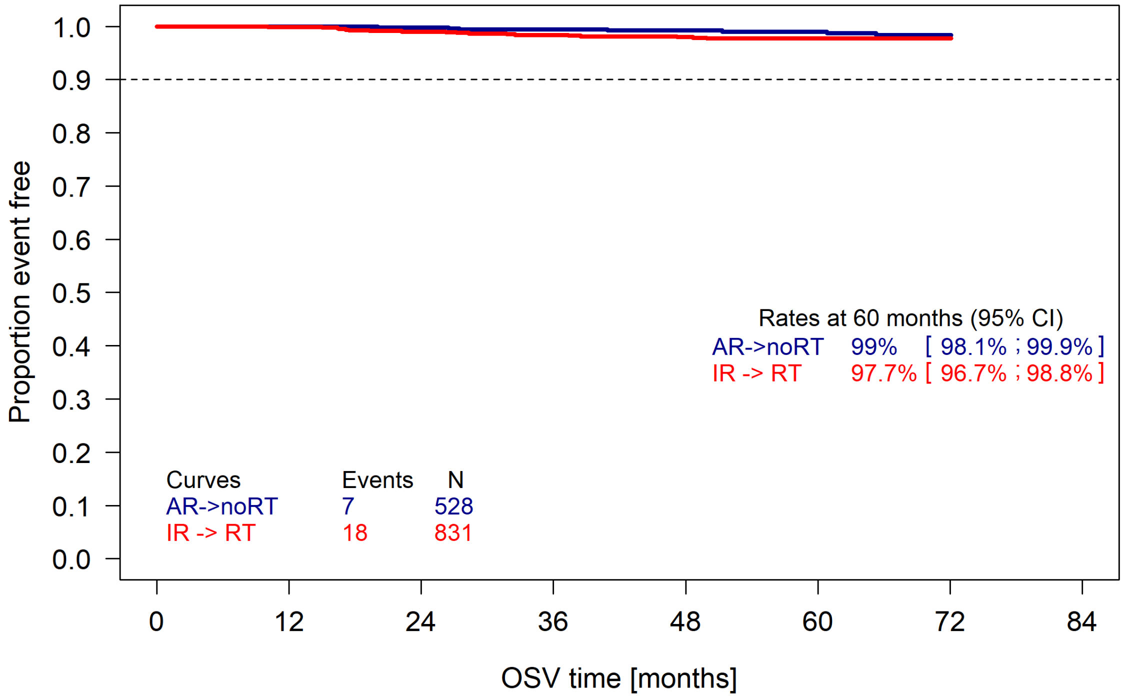

N at Risk (N censored)							
AR->noRT	528(0)	522(6)	514(14)	501(27)	479(49)	424(102)	119(406)
IR -> RT	831(0)	818(12)	802(28)	776(54)	745(86)	667(163)	229(592)

Figure S4C: Overall Survival in the intention to treat analysis of the titration trial showing patients with AR and no RT (blue) and patients with IR scheduled for RT (red). Note six patients without documented response at ERA are not included.

6.4 IIT EFS randomised comparison COPP versus COPDAC

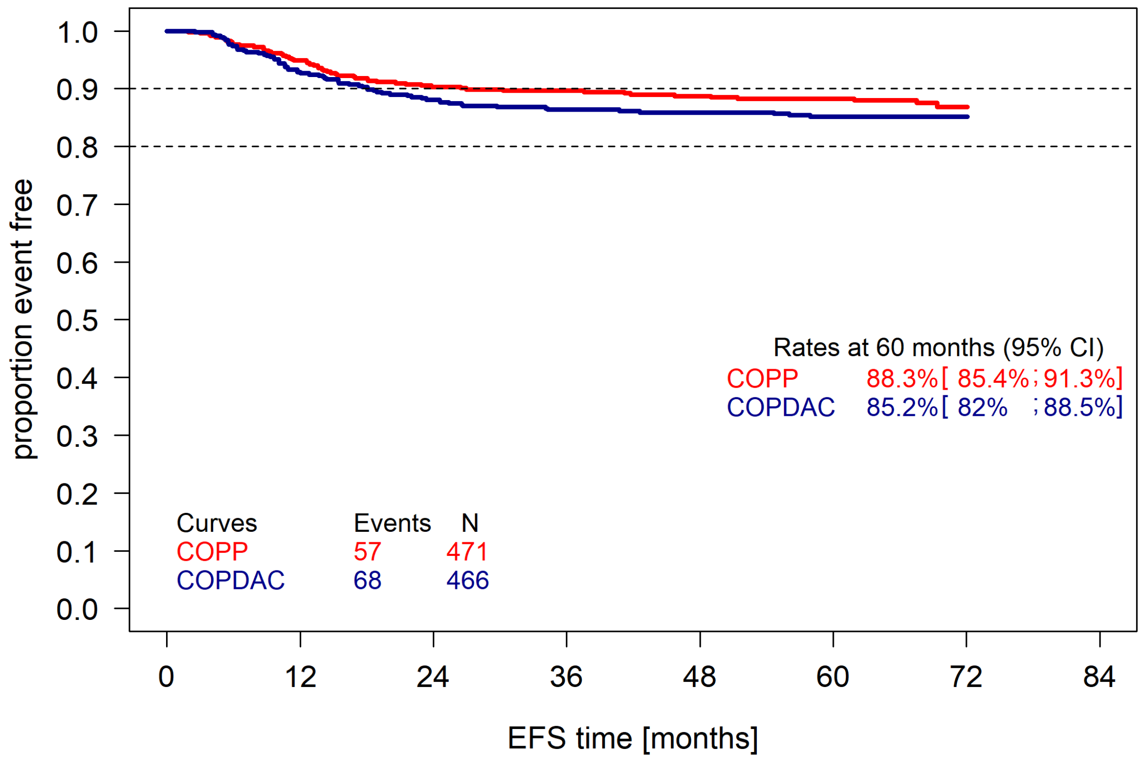

N at Risk (N censored)							
COPP	471(0)	439(42)	414(71)	403(86)	381(110)	348(149)	96(468)
COPDAC	466(0)	430(51)	402(88)	385(108)	371(122)	334(171)	131(426)

Figure S5A: Event-Free Survival in the intention to treat analysis of the embedded randomised trial for the COPP (in red) and the COPDAC (in blue).

6.5 IIT PFS randomised comparison COPP versus COPDAC

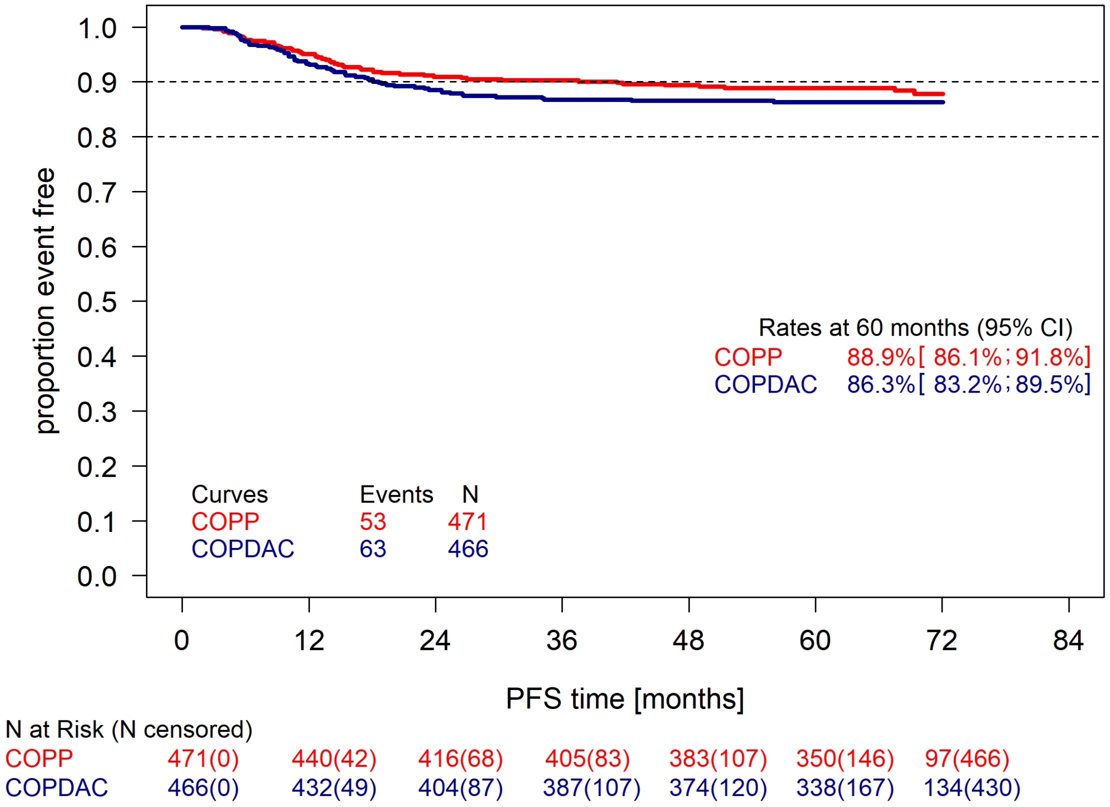

Figure S5B: Progression -Free Survival in the intention to treat analysis of the embedded randomised trial for the COPP (in red) and the COPDAC (in blue).

6.6 IIT OS randomised comparison COPP versus COPDAC

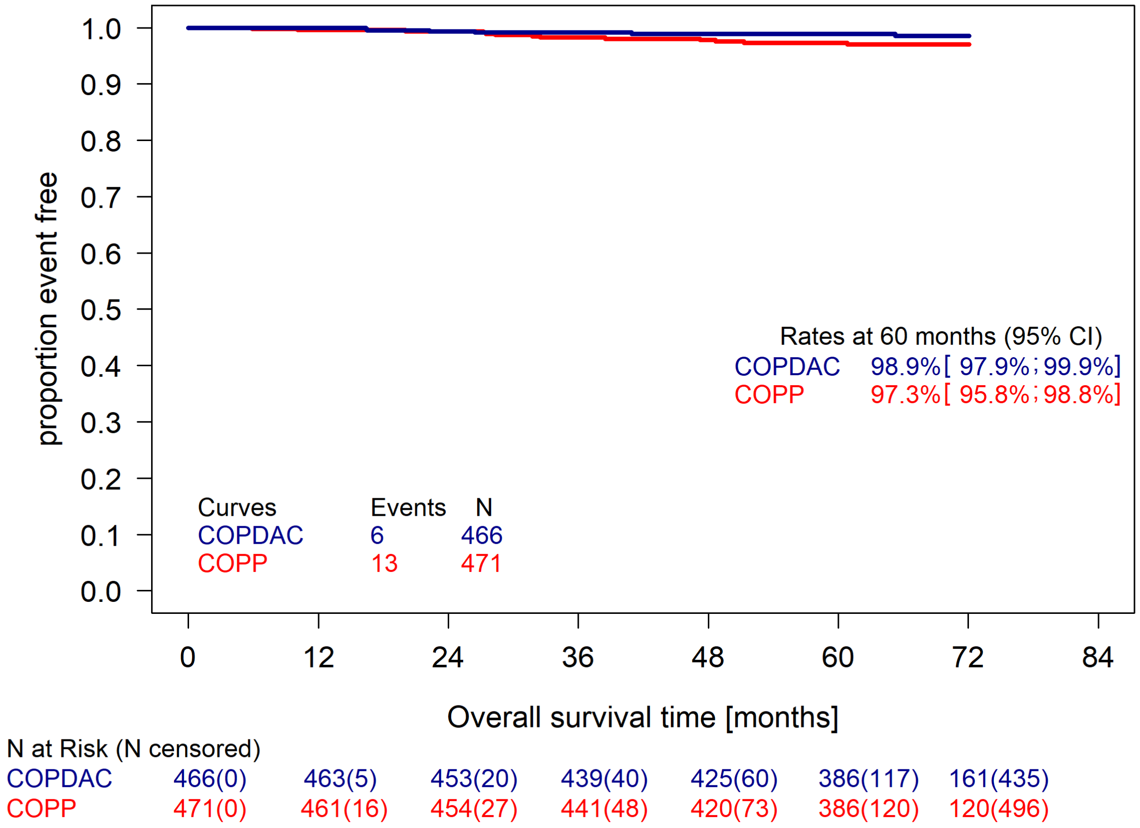

Figure S5C: Overall Survival in the intention to treat analysis of the embedded randomised trial for the COPP (in red) and the COPDAC (in blue).

7. Secondary Analyses in the Per-Protocol set

7.1 Overall Survival in AR-noRT and IR-RT

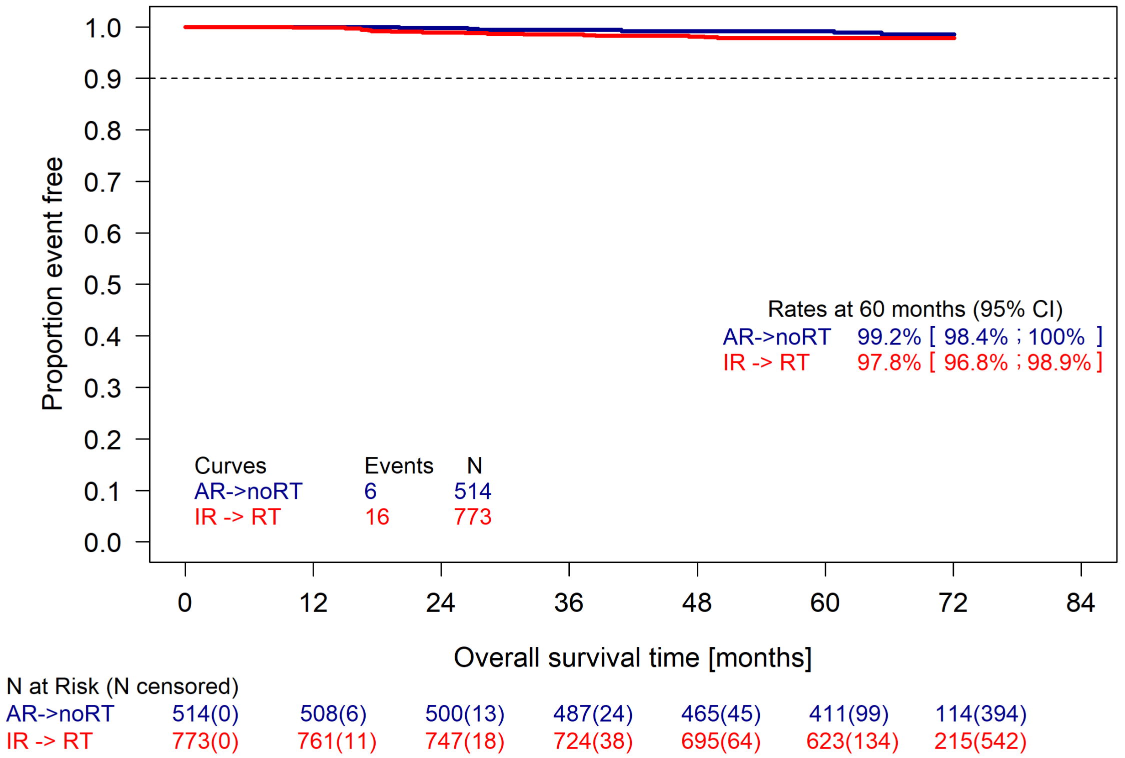

Figure S6A: In overall survival, there are no apparent treatment differences between AR and IR patients in the titration study (PP).

7.2 Overall Survival in the embedded randomised sub-study

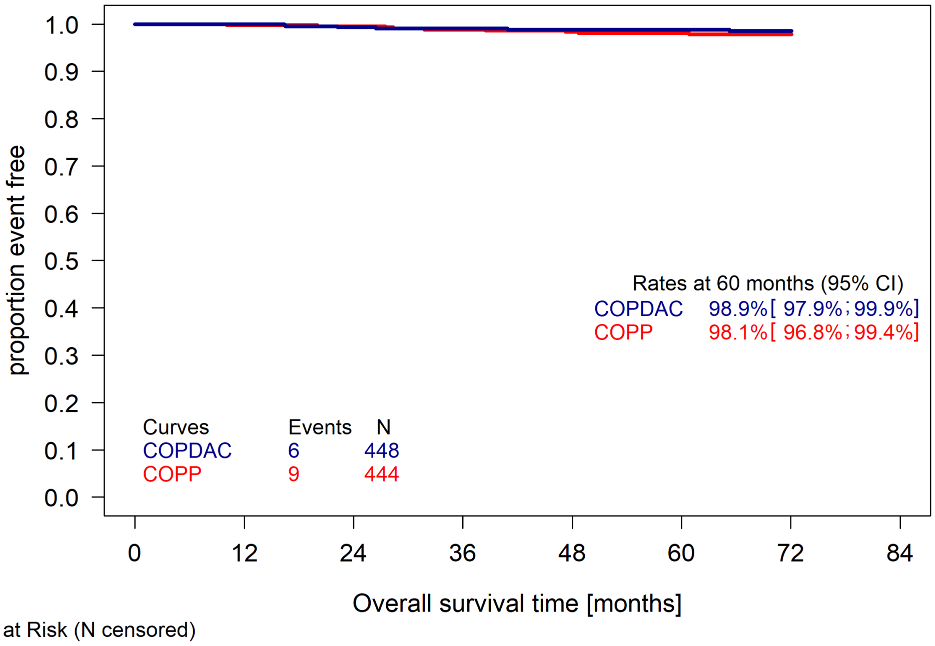

N at Risk (N censored)

COPDAC	448(0)	445(5)	435(19)	422(36)	408(55)	371(110)	155(410)
COPP	444(0)	436(12)	430(20)	419(36)	398(63)	365(109)	113(470)

Figure S6B: In overall survival, there are no apparent treatment differences between COPP and COPDAC in the embedded randomised sub-study (PP).

7.3 Progression Free Survival (PFS) in AR-noRT and IR-RT

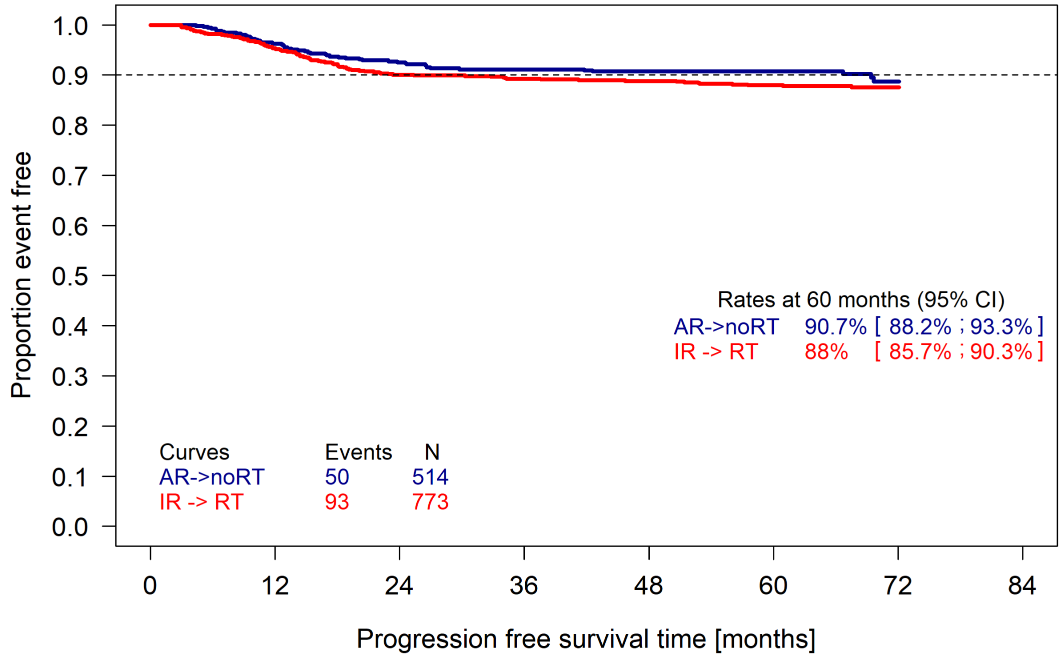

N at Risk (N censored)							
AR->noRT	514(0)	489(6)	464(12)	447(22)	425(42)	376(91)	98(366)
IR -> RT	773(0)	727(10)	681(16)	659(32)	631(57)	561(121)	181(499)

Figure S7A: PFS comparison between AR patients without RT and IR patients with RT in the in titration study (PP).

7.4 Progression Free Survival (PFS) in the embedded randomised sub-study

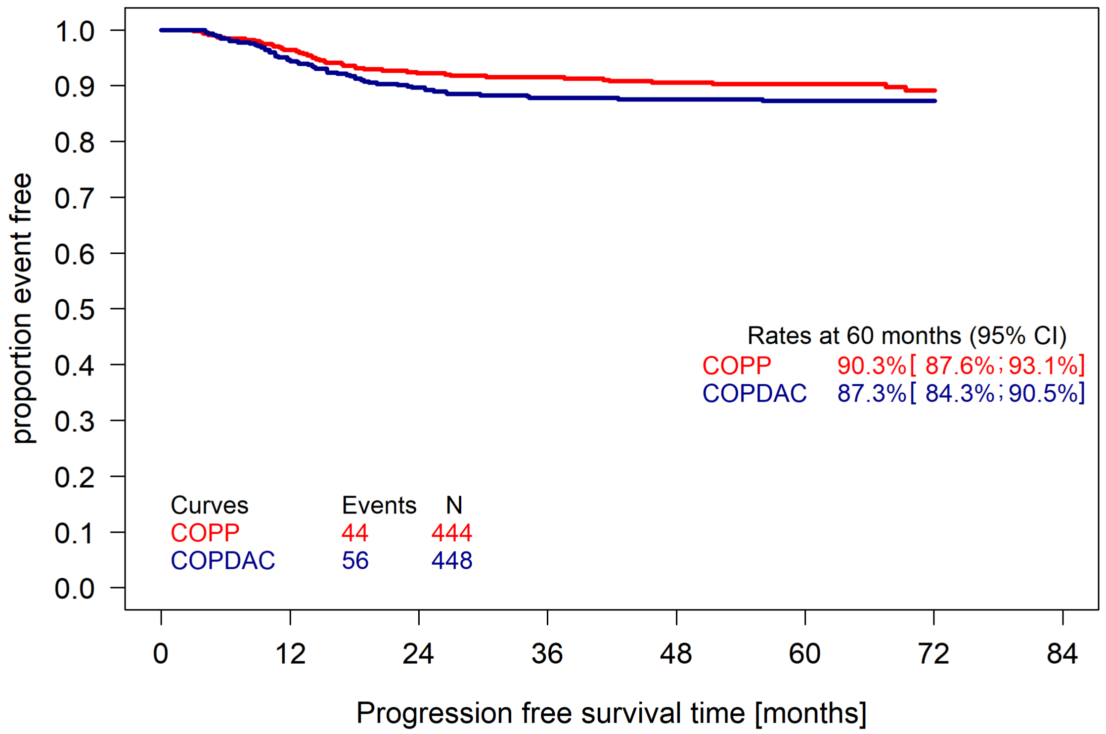

N at Risk (N censored)

COPP	444(0)	421(29)	398(57)	387(72)	365(98)	333(138)	93(445)
COPDAC	448(0)	421(36)	393(73)	376(94)	363(110)	329(157)	132(407)

Figure S7B PFS comparison between COPP and COPDAC in the embedded randomised sub-study (PP).

8. Unplanned subgroup analyses by AR versus IR in the embedded randomised sub-study

8.1 EFS of COPP versus COPDAC in irradiated patients with inadequate response (PP)

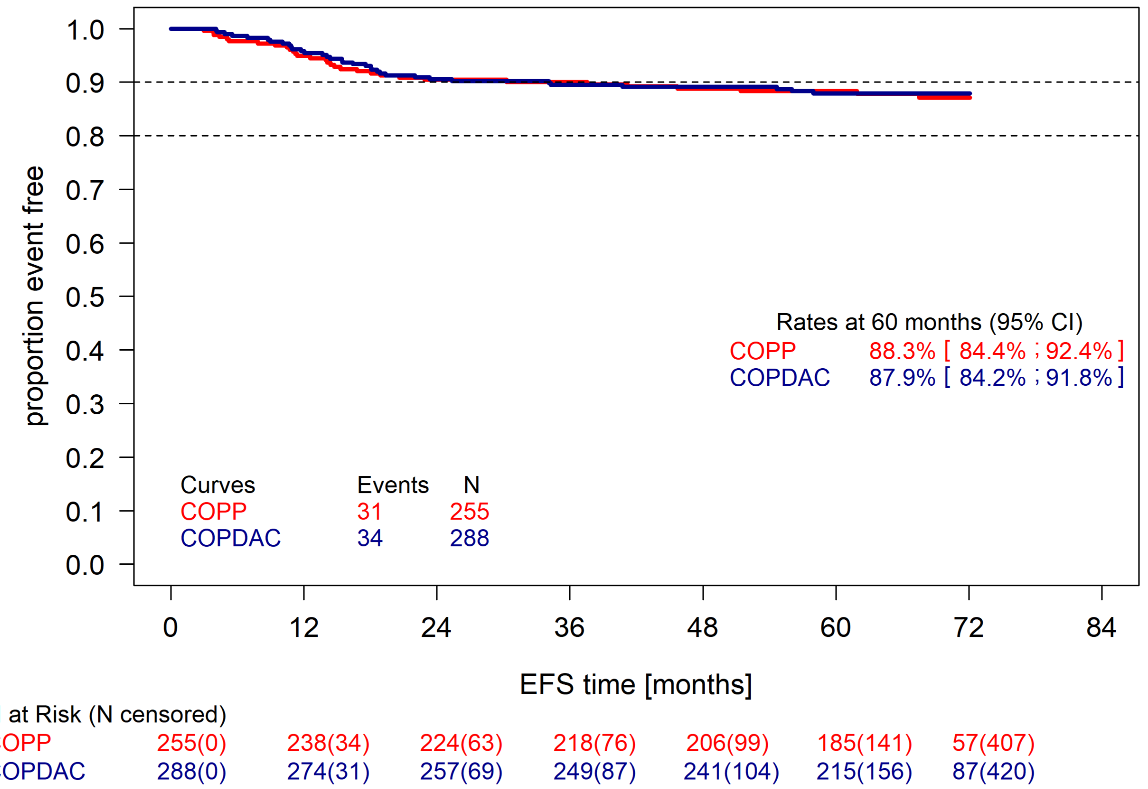

Figure S8A: Unplanned subgroup analysis in the in the embedded randomised sub-study. EFS curves of COPP and COPDAC are superimposable in IR patients scheduled for radiotherapy.

8.2 EFS of COPP versus COPDAC in not irradiated patients with adequate response (PP)

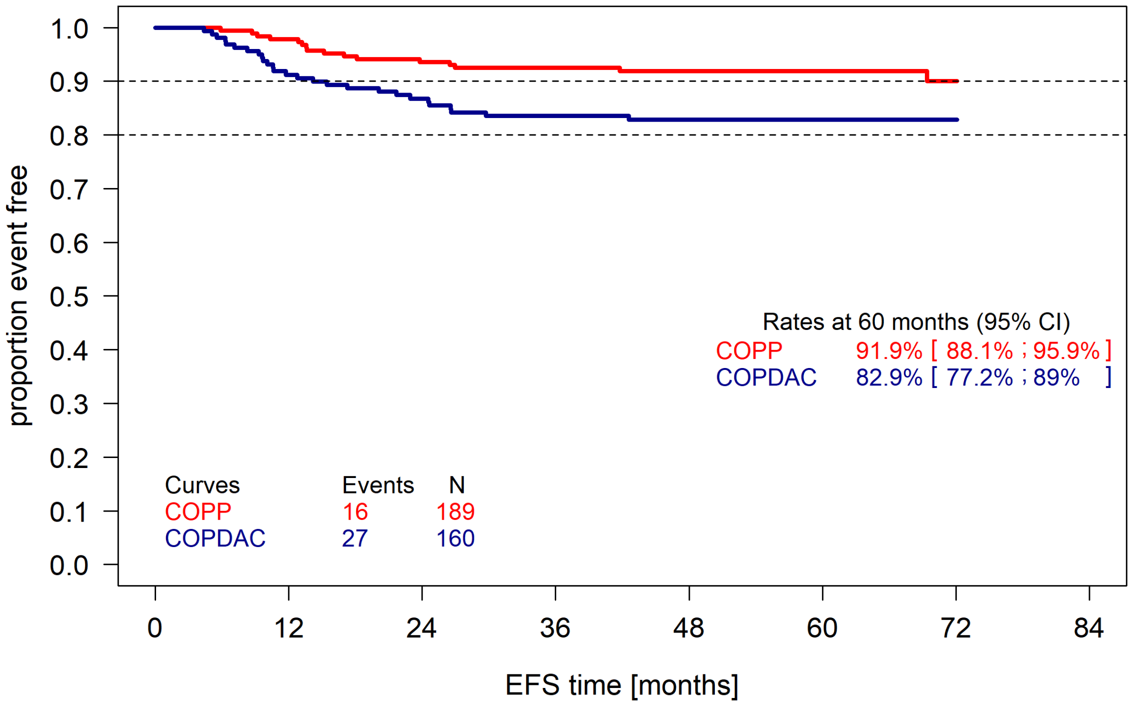

N at Risk (N censored)							
COPP	189(0)	182(23)	173(54)	168(72)	158(101)	147(137)	36(497)
COPDAC	160(0)	145(49)	134(83)	125(111)	119(128)	110(158)	42(381)

Figure S8B: Unplanned subgroup analysis in the in the embedded randomised sub-study (PP). In non-irradiated AR patients, 5yrEFS with COPDAC is 9% less than with COPP.

9. Further Analysis of the fertility data

9.1 Inhibin B

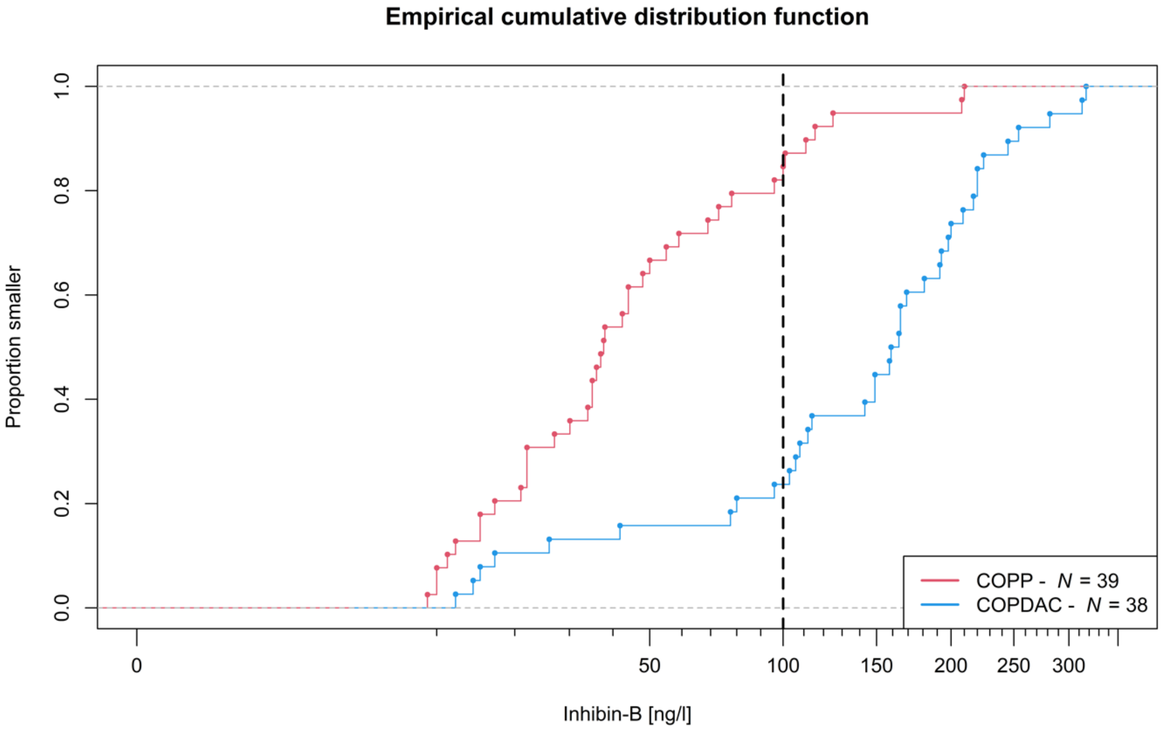

Figure S9: Empirical cumulative distribution function of Inhibin B serum levels in follow-up in boys by randomised consolidation chemotherapy. The dotted vertical line indicates a cut-off below which Inhibin B levels indicate gonadal damage. Inhibin-B is clearly reduced after COPP (median 37.7 ng/l) as compared to COPDAC (median 161.5 ng/l) ($p < 0.0001$).

10.List of secondary neoplasia as first events in Per Protocol Set

TG	Response	Consolidation	RCT	Diagnosis	Time to neoplasia[months]	Died
TG-2	AR	COPDAC	yes	Acute Myeloid Leukemia	21.7	
TG-2	AR	COPP	yes	Marginal zone B-cell Lymphoma	13.6	
TG-2	IR	COPDAC	yes	Papillary thyroid carcinoma	57.9	
TG-3	AR	COPDAC	no	Papillary thyroid carcinoma	58.7	
TG-3	IR	COPDAC	yes	Langerhans cell histiocytosis	6.9	
TG-3	IR	COPDAC	yes	Alveolar rhabdomyosarcoma	54.7	
TG-3	IR	COPDAC	no	Thyroid carcinoma	55.8	
TG-3	IR	COPDAC	yes	Diffuse Large B-Cell Lymphoma	40.7	
TG-3	IR	COPP	yes	Acute Myeloid Leukemia	10.7	yes
TG-3	IR	COPP	yes	Thyroid carcinoma	61.9	

Table S6 Details on secondary neoplasia as first events in the per protocol set up to 72 months

EuroNet-PHL-C1

EuroNet-Paediatric Hodgkin's Lymphoma Group

First international Inter-Group Study for classical Hodgkin's Lymphoma in Children and Adolescents

- No radiotherapy in patients with adequate response at first restaging after two cycles of chemotherapy
- Randomised comparison of Procarbazine versus Dacarbazine (within COPP versus COPDAC) in patients in intermediate and advanced stages
- Standardised risk- and response-adapted salvage strategy

EudraCT-No.: 2006-000995-33

Inter-group chairpersons

Prof. Dr. Dieter Körholz
(Coordinating investigator)
Zentrum für Kinderheilkunde
Universitätsklinik und Poliklinik für Kinder- und Jugendmedizin
Ernst-Grube-Straße 40, 06120 Halle (Saale)
Germany

Prof. Dr. W. Hamish Wallace
Royal Hospital for Sick Children,
Sciennes Road, Edinburgh EH9 1LF,
Scotland, UK.

Prof. Dr. Judith Landman-Parker
Service d'hématologie et d'oncologie pédiatrique
Hôpital Trousseau AP-HP Paris
France

Start of study:	30.01.2007	
End of study accrual:	29.01.2013 (Germany 29.01.2012)	
Date of Version:	12.11.2012	
Version:	Final (dated 07.08.2006)	+ 1st amendment (dated 20.07.2007)
		+ 2nd amendment (dated 05.12.2007)
		+ 3rd amendment (dated 17.11.2009)
		+ 4th amendment (dated 22.12.2011)
		+ 5th amendment (dated 22.02.2012)
		+ 6th amendment (dated 12.11.2012)

Confidentiality

The content of the protocol and the case report forms must be **treated confidentially** and may not be imparted to uninvolved persons without consent of the study chairpersons neither in oral nor in written form.

Important information

The protocol was written by the trial steering committee to the best of their knowledge and belief. Nevertheless mistakes can never be completely excluded.

Therefore every doctor is responsible for checking the treatment plans of the protocol before treating a patient.

CONTENTS

Page

1 GENERAL INFORMATION...........13

1.1 RESPONSIBILITIES...........13

1.2 REFERENCE FACILITIES...........18

1.2.1 Reference pathology...........18

1.2.2 Central staging and response assessment...........18

1.2.3 Reference radiotherapy...........19

1.3 TRIAL RELATED COMMITTEES...........21

1.3.1 Inter-group trial steering committee...........21

1.3.2 Committees of the pre-existing study groups...........21

1.3.3 Scientific side projects (Scientific Advisory Board)...........21

1.3.4 Data Monitoring Committee...........21

1.4 PROTOCOL SYNOPSIS...........23

1.5 FLOWCHART OF THE STUDY *(UPDATED 2012-02-2012)*...........27

2 STUDY OBJECTIVES...........28

2.1 MAIN GOALS OF THE STUDY...........28

2.2 PRIMARY OBJECTIVES...........30

2.3 SECONDARY OBJECTIVES...........30

2.4 TERTIARY OBJECTIVE...........30

3 RATIONALE OF THE STUDY...........31

3.1 PRIOR EXPERIENCE OF THE DAL / GPOH-HD STUDY GROUP...........31

3.1.1 First study generation DAL-HD 78...........31

3.1.2 Second study generation DAL-HD 82...........31

3.1.3 Third study generation DAL-HD 85...........31

3.1.4 Fourth study generation DAL-HD 87...........32

3.1.5 Fifth study generation DAL-HD 90...........32

3.1.6 Sixth study generation GPOH-HD 95...........33

3.1.7 GPOH-HD 2002 Pilot study...........33

3.2 FORMAL INTEGRATION OF FDG-PET IMAGING INTO STAGING AND RESPONSE ASSESSMENT...........40

3.2.1 Relevance of FDG-PET for the initial staging in Hodgkin's lymphoma 41

3.2.2 Timing of FDG-PET response assessment after chemotherapy........43
3.2.3 FDG-PET in response assessment at the end of treatment 43
3.2.4 Early response assessment with FDG-PET 44
3.2.5 Stop of Randomisation..46
3.2.6 Identification of a high risk group in TG1 47
3.3 MODIFICATIONS IN RELATION TO GPOH-HD 95 49
3.3.1 Modifications for all patients...49
3.3.2 Randomised comparison of COPP and COPDAC in TG-2+3..............50
3.4 FURTHER EXPERIENCE OF PARTICIPATING STUDY GROUPS 50
3.4.1 Response-based strategies in Hodgkin Lymphoma: Experience of the SFCE (Société Française de Lutte contre le Cancer de L'Enfant et de l'adolescent)...50
4 TRIAL DESIGN AND DESCRIPTION 52
4.1 TRIAL DESIGN..52
4.2 REQUIREMENTS FOR PARTICIPATING INVESTIGATORS AND TRIAL SITES 52
4.3 TRIAL SITES AND NUMBER OF TRIAL SUBJECTS...53
4.4 EXPECTED DURATION OF TRIAL 53
4.5 PREMATURE TERMINATION 54
4.5.1 Premature closure of a trial site..54
4.5.2 Premature termination of the trial or of trial arms..............................54
5 SELECTION OF TRIAL SUBJECTS FOR PRIMARY THERAPY 56
5.1 INCLUSION CRITERIA 56
5.2 EXCLUSION CRITERIA 56
5.3 SUBSEQUENT EXCLUSION OF PATIENTS..57
5.4 GENDER DISTRIBUTION 57
6 DIAGNOSTICS 58
6.1 CONFIRMATION OF DIAGNOSIS 58
6.2 CLINICAL AND LABORATORY DIAGNOSTICS BEFORE / DURING THERAPY....................58
6.2.1 Diagnostics prior to chemotherapy..58
6.2.2 Diagnostics assessment before each course of chemotherapy........59
6.3 IMAGING DIAGNOSTICS 60
6.3.1 Initial staging...60

6.3.2 FDG-PET examination 62
6.4 ASSESSMENT OF INVOLVED REGIONS 62
6.4.1 Assessment of nodal involvement 63
6.4.2 Assessment of extra-nodal involvement 63
6.4.3 Organ involvement 64
6.5 INDICATIONS FOR INVASIVE DIAGNOSTICS 65
6.5.1 Bone marrow biopsy 65
6.5.2 Selective laparoscopy 66
6.5.3 Ovariopexy 66
6.6 OPTIONAL PROCEDURES 66
6.7 RESTAGING 66
6.7.1 General information 66
6.7.2 First restaging after 2 cycles of chemotherapy 67
6.7.3 Second restaging after full chemotherapy in therapy groups 2 + 3 ..67
6.7.4 Follow-up 67
6.7.5 Assessment of puberty and fertility 69

7 STAGE CLASSIFICATION AND DEFINITION OF THERAPY OUTCOME...70

7.1 STAGE CLASSIFICATION 70
7.2 DEFINITION OF TREATMENT RESPONSE 71
7.2.1 Local response definitions for nodal involvement with measurable tumour volume 71
7.2.2 Local response definitions for extra-nodal involvement or for nodal involvement with non-measurable tumour volume 73
7.2.3 Overall (patient level) response definitions 73
7.3 EARLY FDG-PET RESPONSE ASSESSMENT 75
7.3.1 Definition local FDG-PET response 75
7.3.2 Definition worst local FDG-PET response 77
7.4 RESPONSE GROUP DEFINITION 78

8 THERAPY PLAN FOR PRIMARY THERAPY 80

8.1 TREATMENT GROUPS 80
8.2 TREATMENT GROUP 1 (TG-1) 80
8.3 TREATMENT GROUP 2 AND 3 (TG-2, TG-3) 81
8.4 CHEMOTHERAPY PLANS 82

8.4.1 OEPA.....82
8.4.2 COPDAC.....83
8.5 DOSE MODIFICATIONS.....84
8.6 CONTRACEPTION.....84
8.7 SIDE EFFECTS OF CHEMOTHERAPY.....84
8.7.1 Etoposide.....85
8.7.2 Dacarbazine (DTIC).....85
8.7.3 Vincristine (VCR).....85
8.7.4 Cyclophosphamide.....85
8.7.5 Adriamycin (Doxorubicine).....85
8.7.6 Prednisone/Prednisolone.....85
8.7.7 Procarbazine - erased.....86
8.8 RADIOTHERAPY.....86
8.8.1 Target volumes (Modified Involved Field and Boost Radiotherapy).....86
8.8.2 Radiation planning.....86
8.8.3 Technical requirements.....87
8.8.4 Side Effects of radiotherapy.....87
8.9 SUPPORTIVE CARE.....88
8.9.1 Antibacterial prophylaxis.....88
8.9.2 Prevention of GvH reaction / infection through blood transfusions.....88
8.9.3 Antifungal prophylaxis.....88
8.10 ONCOLOGICAL EMERGENCIES.....88
8.10.1 Large mediastinal tumour.....88
8.10.2 Tumour lysis syndrome.....89

9 RELAPSE TREATMENT.....90

9.1 RATIONALE OF THE RELAPSE THERAPY AS PROVIDED IN THE PROTOCOL.....90
9.1.1 Results of previous studies.....90
9.1.2 Prognostic factors.....91
9.1.3 High dose chemotherapy and autologous stem cell transplantation in relapsed patients.....92
9.2 RELAPSE TREATMENT WITHIN EURONET-PHL-C1.....93
9.3 CHEMOTHERAPY PLANS IN SECOND LINE TREATMENT.....97
9.3.1 IEP.....98

9.3.2 ABVD 98
9.4 BEAM HIGH-DOSE CHEMOTHERAPY AND AUTOLOGOUS SCT 99
9.5 SIDE EFFECTS OF THE DRUGS USED IN SECOND LINE THERAPY 99
9.6 RADIOTHERAPY IN RELAPSE PATIENTS 100
9.6.1 Indication for radiotherapy...100
9.6.2 Radiation volume and radiation dose 100
10 ORGANISATION..101
10.1 INFORMED CONSENT 101
10.1.1 Withdrawal of informed consent...102
10.2 PRIMARY THERAPY 103
10.2.1 Procedures for all treatment groups 103
10.2.2 Further procedures in treatment group 1 (TG-1) 105
10.2.3 Further procedures in treatment groups 2 and 3 (TG-2 + TG-3).......105
10.3 PATIENTS WITH FIRST RELAPSE THERAPY..107
10.3.1 Procedures for all patients 107
10.3.2 Procedures for early response assessment (first restaging)...........108
10.3.3 Further procedures for patients with late relapse after therapy within TG-1 or for patients with adequate response after early relapse or (late relapse after therapy within TG-2 or TG-3) 110
10.3.4 Further procedures for patients with progression or with inadequate response after early relapse or (late relapse after therapy within TG-2 or TG-3) 111
10.4 PREMATURE TERMINATION OF THERAPY OR FOLLOW-UP.....................................113
10.4.1 Premature termination of trial therapy 113
10.4.2 Premature termination of follow-up..114
10.5 ORGANISATION OF FDG-PET EXAMINATIONS 114
11 ADVERSE EVENTS (AE/SAE) 115
11.1 SAFETY MONITORING 115
11.2 ADVERSE EVENT (AE)...115
11.2.1 Definition...115
11.2.2 Documentation and Reporting 115
11.3 SERIOUS ADVERSE EVENT (SAE) 115
11.3.1 Definition...115

11.3.2 Documentation and reporting......116
11.4 SUSPECTED UNEXPECTED SERIOUS ADVERSE DRUG REACTIONS (SUSAR)......118
11.4.1 Definition......118
11.4.2 Evaluation and reporting......118
11.5 THERAPEUTIC PROCEDURES......119
11.6 CLASSIFICATION OF THE ADVERSE EVENT......119
11.6.1 Severity......119
11.6.2 Causal relationship......120
11.6.3 Expected/Unexpected......121
11.6.4 Outcome......121

12 BIOMETRICAL ASPECTS OF THE STUDY......122

12.1 OVERALL STUDY DESIGN CONSIDERATIONS......122
12.2 STUDY POPULATIONS......124
12.2.1 Stratification by method of staging and response assessment (central versus local)......124
12.2.2 Treatment groups......125
12.2.3 Randomisation groups......125
12.2.4 Early Response Groups......125
12.3 EXPECTED NUMBER OF PATIENTS......125
12.4 BIOMETRY FOR PRIMARY THERAPY: ANALYSIS BY EARLY RESPONSE GROUPS......128
12.4.1 AR1 in TG-1......128
12.4.2 AR2 in TG-1 and AR in TG-2+3......128
12.4.3 IR in TG-1 and TG-2+3......128
12.4.4 IRu......129
12.4.5 Power considerations......129
12.5 BIOMETRY FOR FIRST LINE THERAPY: RANDOMISED COMPARISON OF CHEMOTHERAPIES IN TG-2 AND TG-3......130
12.5.1 Randomisation algorithm......130
12.5.2 Statistic formulation (TG-2 + TG-3)......131
12.6 BIOMETRY FOR RELAPSE STUDY......132
12.7 ADDITIONAL QUESTIONS......133
12.7.1 Acute toxicity comparison COPP versus COPDAC......133
12.7.2 Fertility assessment......133

12.7.3 Late toxicities ***136***
12.7.4 Comparison of CSRA and LSRA ***136***
12.8 END POINTS OF STUDIES 136
12.8.1 Primary endpoint ***136***
12.8.2 Secondary endpoints ***136***
12.9 INTERIM ANALYSES AND STOPPING CRITERIA 137
12.9.1 Early assessment of randomisation feasiblity ***137***
12.9.2 Stopping treatment arms with response adaptation ***137***
12.9.3 Stopping the randomised chemotherapy comparison ***138***
12.9.4 Termination of arms of the relapse study ***138***
12.10 STATISTICAL ANALYSES 138
13 REFERENCE EVALUATION **140**
13.1 CENTRAL STAGING AND RESPONSE ASSESSMENT 140
13.1.1 Determination of reference stage ***140***
13.1.2 Organisation of the tumour conference ***140***
13.2 REFERENCE RADIATION PLANNING 142
13.2.1 Tasks of the reference radiotherapist (central review) ***142***
13.2.2 Working group for quality control in radiotherapy ***143***
13.2.3 Documentation of radiogenic late effects ***143***
13.3 REFERENCE PATHOLOGY 143
14 SCIENTIFIC AND BIOLOGICAL ADD ON STUDIES **144**
15 ETHICAL AND LEGAL ASPECTS AND AGREEMENTS **145**
15.1 SPONSORSHIP 145
15.2 REQUEST OF AUTHORISATION TO THE COMPETENT AUTHORITIES 145
15.3 APPLICATION FOR NATIONAL ETHICS COMMITTEE OPINIONS 146
15.4 NOTIFICATION OF SUBSTANTIAL AMENDMENTS 146
15.5 DECLARATION OF THE END OF TRIAL 146
15.6 INSURANCE 147
15.7 FURTHER AGREEMENTS 147
16 DATA HANDLING AND RECORD KEEPING **148**
16.1 CASE REPORT FORMS (CRF) 148
16.2 DATA MANAGEMENT 149

16.3 ARCHIVING 149

17 QUALITY CONTROL AND QUALITY ASSURANCE 150

17.1 DIRECT ACCESS TO SOURCE DATA 150
17.2 MONITORING 150
17.3 AUDITS 150
17.4 INSPECTIONS 150

18 DATA PROTECTION 151

18.1 CONFIRMATION OF DATA PROTECTION 151

19 ADMINISTRATIVE AGREEMENTS 152

19.1 ADHERENCE TO THE PROTOCOL 152
19.2 PROTOCOL AMENDMENTS 152
19.3 PUBLICATION POLICY 153

20 REFERENCES 154

21 CONFIRMATION OF THE FINAL PROTOCOL 161

22 PROTOCOL AGREEMENT 162

23 APPENDIX 163

23.1 FACTS OF PUBERTY 163
23.2 CHEMOTHERAPUTIC DRUG MONOGRAPHS 165
23.2.1 BLEOMYCIN 165
23.2.2 CARMUSTINE 167
23.2.3 CYCLOPHOSPHAMIDE 169
23.2.4 CYTARABINE 172
23.2.5 DACARBAZINE 175
23.2.6 DOXORUBICIN 177
23.2.7 ETOPOSIDE 182
23.2.8 IFOSFAMIDE 186
23.2.9 MELPHALAN (HIGH DOSE INTRAVENOUS) 190
23.2.10 PROCARBAZINE 193
23.2.11 VINBLASTINE 195
23.2.12 VINCRISTINE 197

23.3 ABBREVIATIONS....201
23.4 GPOH-HD HODGKIN'S LYMPHOMA WORKING GROUP....206
23.5 NCRI HODGKIN'S LYMPHOMA WORKING GROUP....207
23.6 SFCE HODGKIN'S LYMPHOMA WORKING GROUP....208
23.7 PPLLSG HODGKIN'S LYMPHOMA WORKING GROUP....209
23.8 SCIENTIFIC ADVISORY BOARD....210

LIST OF TABLES Page

Table 1 Toxicity evaluation for OE*PA in the GPOH-HD 2002 Pilot Study 35
Table 2 Toxicity evaluation for COPDAC in the GPOH-HD 2002 Pilot Study 37
Table 3 Treatment evolution since DAL HD 78 up to EuroNet-PHL-C1 38
Table 4 Comparison of the significance of CT/MRI and FDG-PET for the assessment of therapy success 41
Table 5 FDG-PET integrated into initial staging in Hodgkin's lymphoma 42
Table 6 FDG-PET results in late assessment of therapy 44
Table 7 Early response assessment with PET (from Hutchings et al, 2004) 44
Table 8 Early response assessment with FDG-PET after 2 OPPA or OEPA in GPOH-HD 2002 Pilot 45
Table 9 Planned therapy duration 54
Table 10 Recommandations for follow-up examinations 68
Table 11 Independent lymph node regions 70
Table 12 Stage classification Hodgkin's lymphoma 71
Table 13 Annotations to stage definitions 71
Table 14 Response groups in non progressing patients 79
Table 15 OEPA scheme 83
Table 16 COPP scheme 83
Table 17 COPDAC scheme 83
Table 18 IEP scheme 98
Table 19 ABVD scheme 98
Table 20 BEAM high-dose chemotherapy 99
Table 21 Expected sample sizes in relevant subgroups. 127

LIST OF FIGURES Page

Fig. 1. Flowchart on study proceedings 27
Fig. 2. FDG-PET staging results 42
Fig. 3. Definition of overall response 73
Fig. 4. Definition local FDG-PET response 76
Fig. 5. Response group definition 78
Fig. 6. Treatment overview for TG-1 80
Fig. 7. Treatment overview for TG-2 and TG-3 81
Fig. 8. Results of DAL ST-HD-86 90
Fig. 9. Overall survival according to prognostic groups (DAL ST-HD-86) 91
Fig. 10. Disease free survival according to prognostic groups (DAL ST-HD-86) 91
Fig. 11. Treatment plan for second line treatment 96
Fig. 12. Expected precision of rate estimates 129
Fig. 13. Expected accuracy of estimate for the estimation of rate differences 131

1 GENERAL INFORMATION

On 16 Sept 2005 in Leipzig the GPOH-HD group and study groups from Poland, Czech Republic, Slovakia and the UK agreed to run a European protocol in children and young people with classical Hodgkin's lymphoma within the EuroNet-Paediatric Hodgkin's Lymphoma group (EuroNet-PHL). Further study groups are encouraged to join.

The EuroNet-PHL-C1 trial is a multinational inter-group trial, involving pre-existing national or cross-national study groups.
The study groups involved are:

- GPOH-HD (Central Review)
  German Society for Paediatric Oncology and Haematology – Study Group for Hodgkin's lymphoma. This group involves centres from Germany, Austria and Switzerland, Sweden, Denmark and Norway (NOPHO group), Czech Republic, Slovakia. These countries are committed to central review of diagnostic staging and response assessment.
- National Cancer Research Institute (NCRI) Lymphoma Clinical Study Group (Central review of imaging) - England, Scotland, Ireland and Wales
- Poland (Non-central review)
  Polish Pediatric Leukemia/Lymphoma Study Group
- France - SFCE (Non-central review)
  Société Française de lutte contre les cancers et les leucémies de L'enfant et de l'adolescent (SFCE)
- Spain (Non-central review)
  Spanish Society of Pediatric Oncology (SEOP)

Further study groups may join either the central or the non-central review group after the start of the study .

1.1 RESPONSIBILITIES

**Sponsor**<br>according to GCP	**Martin Luther University of Halle/Wittenberg**<br>Medizinische Fakultät, Prodekanat Forschung<br>Magdeburger Str. 27<br>06097 Halle (Saale)<br>Tel +49-345-5575420, Fax +49-345-5575424<br>siegfried.bauer@medizin.uni-halle.de<br>**Representative of the sponsor: Prof. Dr. D. Körholz**

**Coordinating chairperson**<br>**Inter-group chairperson**<br>**Study chairperson GPOH-HD**	**Prof. Dr. Dieter Körholz**<br>Zentrum für Kinderheilkunde<br>Universitätsklinik und Poliklinik für Kinder- und Jugendmedizin<br>Ernst-Grube-Straße 40, 06120 Halle (Saale)<br>Tel +49-345-5572387, Fax +49-345-5572389<br>Email: dieter.koerholz@medizin.uni-halle.de
**Inter-group chairperson and National chairperson NCRI**	**Prof. Dr. W. Hamish Wallace**<br>Consultant Paediatric Oncologist<br>Royal Hospital for Sick Children,<br>Sciennes Road, Edinburgh EH9 1LF, Scotland, UK.<br>Tel +44-131 536 0426, Fax +44-131 536 0430<br>Email: hamish.wallace@luht.scot.nhs.uk
**Inter-group chairperson and Study chairperson SFCE**	**Prof. Dr. Judith Landman-Parker**<br>Hopital d`Ènfants Armand Trousseau<br>26, Avenue du Dr Arnold Netter<br>75571 Paris Cedex 12, France<br>Tel +33-1-44737475, Fax +33-1-44736573<br>Email : judith.landman-parker@trs.ap-hop-paris.fr
**Study chairperson Austria**	**Dr. Georg Mann**<br>St. Anna Kinderspital,<br>Zentrum f. Kinder und Jugendheilkunde<br>Kinderspitalgasse 6A<br>A-1090 Wien, Austria<br>Tel +43-1-401704780, Fax +43-1-401707430<br>Email: georg.mann@stanna.at
**Study chairperson Belgium BSPHO**	**Dr Anne Uyttebroeck**<br>Paediatric haemato-oncology<br>University Hospitals of Leuven,<br>Herestraat 49<br>3000 Leuven, Belgium<br>Tel +32 00 343972, Fax +32 16 343842<br>Email: Anne.uyttebroeck@uz.kuleuven.be
**Study chairperson Czech Republic**	**Dr. Michaela Cepelová**<br>Dpt. of Pediatric Hematology and Oncology, Faculty Hospital Motol,<br>V Úvalu 84, Prague 5, 150 06, Czech Republic<br>Tel +420 224 436 454, Fax: + 420 224 436 420<br>Email: michaela.cepelova@fnmotol.cz
**Study chairperson Denmark**	**Dr. Eckhard Schomerus**<br>Odense Universitetshospital<br>5000 Odense, Denmark<br>Tel +45 6541 2086; Fax +45 6312 2703<br>Email: Eckhard.Schomerus@ouh.regionsyddanmark.dk
**Study Chairperson Netherlands**	**Study chairperson Netherlands DCOG**<br><br>**Dr. Auke Beishuizen**<br><br>Erasmus MC - Sophia Childrens Hospital University

	Medical Center Rotterdam, Dept of Pediatric Oncology/Hematology, PO-Box 2060, 3000 CB Rotterdam, The Netherlands,<br><br>e-mail: a.beishuizen@erasmusmc.nl
**Study chairperson Norway**	**Alexander Fosså, MD**<br>Department of Medical Oncology and Radiotherapy<br>Rikshospitalet - Radiumhospitalet HF<br>Montebello, 0310 Oslo, Norway<br>Phone: +4722934000, Fax: +4722935599<br>E-mail: alexander.fossa@radiumhospitalet.no
**Study chairperson Poland**<br>**Polish Pediatric Leukemia /Lymphoma Study Group**	**Prof. Dr. Walentyna Balwierz**<br>Head of Department of Pediatric Oncology and Hematology, Polish-American Pediatric Institute, Jagiellonian University Medical Faculty<br>Wielicka Str. 265, 30-663 Krakow, Poland<br>Tel./Fax +48-12-65802-61,<br>Email: balwierz@mp.pl
**Study chairperson Slovakia**	**Dr. Andrea Hraskova**<br>Clinic of Pediatric Oncology<br>University Children`s Hospital<br>Limbova 1, 83340 Bratislava<br>Tel +4212-59371230, Fax +421254788000<br>Email: andrea.hraskova@zoznam.sk
**Study chairperson Spain**	**Dr Ana Fernández-Teijeiro Álvarez**<br>Jefe de Sección de Onco-Hematología Pediátrica<br>Hospital Universitario Virgen Macarena<br>Avda. Dr. Fedriani nº 3<br>41071-Sevilla<br>Tel: +34677903132, Fax: +34954370892<br>E-mail: anateijeiro@hotmail.com
**Study chairperson Sweden**	**Dr. Jonas Karlén**<br>Pediatric Cancer Unit, Astrid Lindgrens Childrens Hospital, Karolinska University Hospital,<br>S-171 76 Stockholm, Sweden<br>Tel +46-8-51773016, Fax +46-8-51774467<br>E-mail: jonas.karlen@karolinska.se
**Study chairperson Switzerland**	**Dr. Eva Bergsträsser**<br>Abteilung Onkologie, Universitäts-Kinderklinik Zürich<br>Steinwiesstr. 75, CH-8032 Zürich, Switzerland<br>Tel. +41 44 266 7723, Fax: +41-44-2667171<br>Email:eva.bergstraesser@kispi.unizh.ch
**Study Secretary**	**Prof. Dr. Christine Mauz-Körholz**<br>Zentrum für Kinderheilkunde<br>Universitätsklinik und Poliklinik für Kinder- und Jugendmedizin<br>Ernst-Grube-Straße 40, 06120 Halle (Saale)<br>Tel +49-345-5572746, Fax +49-345-5572389<br>Email: christine.mauz-koerholz@medizin.uni-halle.de

	Email : hodgkin@medizin.uni-halle.de
**Responsible Biometrician**	**Dr. Dirk Hasenclever**<br>Institut für Medizinische Informatik, Statistik und Epidemiologie, Universität Leipzig<br>Härtelstr.16-18, D-04107 Leipzig, Germany<br>Tel +49-341-9716121, Fax +49-341-9716109<br>Email: dirk.hasenclever@imise.uni-leipzig.de

**Coordinator salvage therapy (GPOH)**	**PD Dr. Alexander Claviez**<br>Klinik für allgemeine Pädiatrie,<br>Universitätsklinikum Schleswig-Holstein, Campus Kiel<br>Schwanenweg 20, D-24105 Kiel, Germany<br>Tel +49-431-5971622/1826, Fax: +49-431- 597-1816<br>Email: a.claviez@pediatrics.uni-kiel.de
**Coordinator salvage therapy (UK)**	**Dr. Steven Daw**<br>Consultant paediatric oncologist<br>UCLH, 1st Floor West, 250 Euston Road<br>London NW1 2PG<br>Tel 0207 380 9950 (PA direct line)<br>Email: stephen.daw@uclh.org
**Coordinator salvage therapy (SFCE, France)**	**Hélène Pacquement**<br>Institut Curie<br>Email: helene.pacquement@curie.net
**Coordinator salvage therapy**<br>**(PPLLSG, Poland)**	**Dr. Angelina Moryl-Bujakowska**<br>Department of Pediatric Oncology and Hematology,<br>Polish-American Pediatric Institute, Jagiellonian University Medical Faculty<br>Wielisca Str. 265, 30-663 Krakow, Poland<br>Tel/Fax+48-12-6580261<br>e-mail: amb6000@op.pl
**Study office GPOH-HD**<br>**Central data base**	**Dr. Oana Brosteanu**<br>Koordinierungszentrum für Klinische Studien Leipzig,<br>Universität Leipzig<br>Härtelstr.16-18, D-04107 Leipzig, Germany<br>Tel +49-341-97 16 - 266 and -275,<br>Fax: +49-341-9716-189<br>Email.: studsek@kksl.uni-leipzig.de
**Study office CRCTU**	**Study office UK**<br>**CRCTU Trial Coordinator**<br>Janis Hayward<br>University of Birmingham<br>Birmingham. B15 2TT<br>Tel: +44 (0)121 415 1045<br>Fax: +44 (0)121 414 9520<br>Email: EuroNetPHL-LP1@trials.bham.ac.uk
**Study office SFCE**	**Prof. Dr. Judith Landman-Parker**<br>Hopital d`Ènfants Armand Trousseau<br>26, Avenue du Dr Arnold Netter<br>75571 Paris Cedex 12, France<br>Tel +33-1-44737475, Fax +33-1-44736573<br>Email : judith.landman-parker@trs.ap-hop-paris.fr

1.2 REFERENCE FACILITIES

1.2.1 Reference pathology

**Reference pathology**<br>**GPOH-HD**	**Association of German Lymphoma reference pathologists**<br>**Coordinating reference pathologist:**<br>**Prof. Dr. Feller**<br>Universitätsklinikum Schleswig-Holstein<br>Campus Lübeck, Institut für Pathologie<br>Ratzeburger Allee 160<br>23538 Lübeck<br>Tel: +49 451 500 2705<br>Fax: +49 451 500 3328<br>Email: ac.feller@uksh.de
**Reference pathology**<br>**NCRI**	**NCRI**<br>**Dr Alan Ramsay**<br>**Consultant Histopathologist**<br>Department of Pathology<br>Rockefeller Building<br>University College London Hospitals NHS Foundation Trust<br>235 Euston Road, London, NW1 2BU<br>Email: alan.ramsay@uclh.nhs.uk
**Reference pathology**<br>**SFCE**	**Dr Josette Brière**<br>Laboratoire d'anatomie pathologique<br>GELA comité Hodgkin pédiatrique<br>Hôpital Saint Louis 1 av Claude Vellefaux<br>75010 Paris<br>Email: josette.briere@sls.aphp.fr
**Reference pathology**<br>**PPLLSG, Poland**	**Jadwiga Małdyk, MD, PhD**<br>Department of Pediatric Pathology<br>Medical College<br>ul. Marszałkowska 24, 00-828 Warszawa, Poland<br>Tel.: +4822 6291040<br>Email: jagusiamaldyk@wp.pl
**Reference pathology**<br>**Czech Republic**	**Prof. Dr. Roman Kodet**<br>Dpt. of pathology and molecular biology<br>Faculty Hospital Motol,<br>V Úvalu 84, Prague 5, 150 06,<br>Czech Republic<br>Tel +420 224 435 601, Fax: + 420 224 435 620<br>Email: roman.kodet@lfmotol.cuni.cz

1.2.2 Central staging and response assessment

**Central review coordinator**	**Prof. Dr. Christine Mauz-Körholz**<br>Zentrum für Kinderheilkunde

	Universitätsklinik und Poliklinik für Kinder- und Jugendmedizin<br>Ernst-Grube-Straße 40, 06120 Halle (Saale)<br>Tel +49-345-5572746, Fax +49-345-5572389<br>Email : hodgkin@medizin.uni-halle.de
**Radiology**	**Prof. Dr. med. Rolf Peter Spielmann**<br>Universitätsklinikum der<br>Martin-Luther-Universität Halle-Wittenberg<br>Universitätsklinik und Poliklinik für Diagnostische Radiologie<br>Ernst-Grube-Straße 40<br>06120 Halle (Saale) Germany<br>Tel +49-345-5572441, Fax +49-345-5572157<br>Email: rolf.spielmann@medizin.uni-halle.de
**Nuclear medicine**	**Prof. Dr. Regine Kluge, Prof. Dr. Osama Sabri**<br>Klinik und Poliklinik für Nuklearmedizin<br>Universitätsklinikum Leipzig<br>Stephanstr. 11, D-04103 Leipzig, Germany<br>Tel. +49-341- 97 18 031 or 97 18 000,<br>Fax +49-341- 97 18 009<br>Email: klur@medizin.uni-leipzig.de,<br>sabri@medizin.uni-leipzig.de

1.2.3 Reference radiotherapy

**Reference radiotherapy**<br>**GPOH-HD**	**Prof. Dr. D. Vordermark**<br>Universitätsklinikum der Martin-Luther-Universität Halle-Wittenberg<br>Universitätsklinik und Poliklinik für Strahlentherapie und Radioonkologie<br>Ernst-Grube-Str. 40, D-06120 Halle (Saale), Germany<br>Tel.: +49 345- 5573422,<br>Fax: +49 345- 5577207<br>Email: thomas.kuhnt@medizin.uni-halle.de<br><br>**Prof. Dr. Karin Dieckmann**<br>Universitätsklinik für Strahlentherapie und Strahlenbiologie<br>Medizinische Universität Wien, Allgemeines Krankenhaus der Stadt Wien<br>A-1090 Wien,Währingergürtel 18 - 21<br>Tel 0043-1-40400-2692<br>Email: Karin.dieckmann@meduniwien.ac.at
**Reference radiotherapy**<br>**NCRI**	**Dr. Eve Gallop-Evans**<br>Velindre Cancer Centre<br>Velindre Road, Whitchurch,<br>Cardiff, CF14 2TL, Wales, UK<br>Email: eve.gallop-evans@wales.nhs.uk
**Reference radiotherapy**<br>**SFCE**	**Dr. Christian Carrie**<br>Centre Léon Berard

	28 rue Laennec<br>F 69373 Lyon cedex 08<br>tel :(33) (0) 4..78.78.26.52<br>fax:(33) (0) 4.78.78.51.40<br>Email: carrie@lyon.fnclcc.fr
**Reference Radiotherapy**<br>**PPLLSG**	**Krzysztof Paprota, MD, PhD**<br>Department of Radiotherapy<br>Children’s Hospital<br>tel.:+4881 7185520,fax:+4881 7477220<br>Email: lekkp@gazeta.pl

1.3 TRIAL RELATED COMMITTEES

1.3.1 Inter-group trial steering committee

The inter-group trial steering committee is responsible for writing the protocol and organizing the study in the participating countries.
The inter-group trial steering committee consists of the study chairpersons of the participating national study groups and the responsible biometrician.
The study secretary is responsible for the minutes of inter-group trial steering committee meetings.
Further experts can be invited to participate as consultants.

1.3.2 Committees of the pre-existing study groups

The GPOH-HD committee as well as the other national committees of the pre-existing study groups are responsible for endorsing and implementing EuroNet–PHL approved protocols. The members of the GPOH-HD committee, of the NCRI, SFCE and PPLLSG Hodgkin's lymphoma working parties are listed in the Appendix.

1.3.3 Scientific side projects (Scientific Advisory Board)

The *Scientific Advisory Board of EuroNet-PHL-C1* evaluates applications of interested working teams and decides on the eligibility of applications in agreement with the study chairpersons and the biometrician of the study. This advisory panel consists of representatives of the study groups, reference pathology and biometry as well as of further recognized experts in the field of malignant lymphomas and paediatric oncology.
Collection of tumour tissue and serum will be handled by different national protocols which will include national regulation for personal data safety. These protocols will be closely related to the EuroNet-PHL-C1 protocol. Scientific study projects that have been favourably reviewed by the SAB will be provided with anonymous clinical data from the study database (see chapter 14).
The current members of the Scientific Advisory Board are listed in the Appendix.

1.3.4 Data Monitoring Committee

The Data Monitoring Committee (DMC) consists of independent medical and biometrical experts.

The DMC receives confidential information on the progress of the study at annual intervals and has the following tasks:

- assessment of the study’s progress
- assessment of safety
- advise on serious adverse events
- assessment of the results of interim analyses if stopping rules are met.

The DMC gives recommendations to the study chairpersons referring to conduct, modification or early termination of the study. These recommendations are discussed and decided upon by the inter-group trial steering committee.

1.4 PROTOCOL SYNOPSIS

**Title of the study**	First international Inter-Group Study for classical Hodgkin`s Lymphoma in Children and Adolescents
**Acronym**	EuroNet-PHL-C1
**Sponsor**	University of Halle/Wittenberg
**Indication**	Classic Hodgkin's lymphoma in childhood and adolescence – first and second line treatment
**Objective**	Building on the experience of the GPOH-HD study group since 1978, first and second line therapy for childhood Hodgkin's lymphoma shall be further optimised to avoid over-treatment and decrease long-term complications.<br>▪ FDG-PET currently is routinely used in most centres. Results of FDG-PET are now formally integrated both into staging and response assessment.<br>▪ In all treatment groups, radiotherapy after completion of chemotherapy will be omitted in patients with adequate response (CR or PR with negative PET) after two cycles of OEPA.<br>▪ In intermediate and advanced stages (TG-2 & TG-3), COPDAC chemotherapy (replacing Procarbazine by Dacarbazine in order to reduce risk of infertility) is randomised versus standard COPP.<br>▪ Relapse treatment is standardised for three relapse groups based on time to failure and initial treatment group.
**Primary objectives**	1. Are 5 year event free survival (EFS) rate estimates in patients with adequate response after 2 OEPA treated without radio-therapy consistent with a target EFS rate of 90% in all treatment groups?<br>2. Can Procarbazine be safely replaced by Dacarbazine in therapy groups TG-2 and TG-3 without a deterioration of EFS (randomised comparison of COPDAC and COPP)?<br>3. Description of treatment outcome to a standardised risk adapted relapse strategy

**Secondary objectives**	1. Is the 5 year event free survival (EFS) rate in patients with inadequate response after 2 OEPA who receive standard involved field radiotherapy consistent with a target EFS rate of 90% estimates in all treatment groups?<br>2. Does substitution of Dacarbazine for Procarbazine in TG-2 and -3 patients decrease the rate of infertility in males and premature menopause for females?
**Tertiary objective**	Exploration of the impact of real-time central staging and response assessment on treatment outcome.
**Study design**	• Quality control treatment titration study in a stable patient population addressing consistency of absolute 5-year EFS rate estimates with a target rate of 90%.<br>• Embedded randomised controlled chemotherapy comparison in TG-2 and TG-3 concerning efficacy and toxicity. Stop randomization 13.2.2012 9:00 a.m.<br>• Quality control treatment titration study for standardised risk adapted relapse therapy. In two subgroups, "patients with late relapses after TG-1" and "adequately responding patients with early relapse or late relapse after TG-2 or TG-3" consistency of absolute 5-year EFS rate estimates with a target rate of 90% is addressed.
**Study population**	• Patients with untreated classical Hodgkin's lymphoma under 18 years of age. (In France only above one year.) and<br>• Patients with 1st relapse of Hodgkin's lymphoma after EuroNet-PHL-C1 first line treatment or after treatment according to or comparable to previous GPOH/DAL studies.
**Sample size**	At least 1200 patients with real time central review will be included in the study on primary therapy. In addition about 600 patients of the SFCE, the PPLLSG and further national study groups using local staging and response assessment are expected. According to past experience these patients are distributed among the therapy groups 1 or 2 and 3 in a ratio of 36:28:36.<br>For the relapse study at least 150-250 patients are expected.
**Therapy**	All first line patients get two cycles of OEPA and then undergo response assessment including FDG-PET. Patients in TG-1 do not receive further chemotherapy. Patients in TG-2 and -3 are randomised to receive either COPP or COPDAC for two or four

	cycles respectively. If an adequate response was documented treatment stops after chemotherapy. In case of inadequate response to 2 OEPA involved field radiotherapy follows for all treatment groups.<br>Relapse patients get therapy adapted to risk and response (details cp. chapter 9).
**Primary end point**	Event free survival (EFS) defined as time from registration until the first of the following events:<br>• progression/relapse of disease<br>• diagnosis of a secondary malignancy<br>• death of any cause.
**Secondary end points**	1. Overall survival (OS)<br>2. Progression free survival (PFS)<br>3. CTC (Common toxicity criteria) toxicity levels of therapy elements<br>4. Evidence of male infertility score / Female sexual functioning score<br>5. Long-term consequences (premature menopauses, secondary cancer etc.)
**Biometry**	• 5-year EFS rates for TG-1 and TG-2 & -3 will be estimated (with 95% confidence intervals) in patients with adequate response after 2 OEPA (and secondarily also in patients with inadequate response). Precision (i.e. halve width of the 95% confidence interval) is expected to be ± 5-6%. The target rate is set at 90%.<br>• In TG-2 and TG-3 COPP is randomly compared to COPDAC. The log hazard ratio will be estimated along with a 95% confidence interval within a proportional hazard model including the factors treatment group (TG-2 versus TG-3), central versus local staging and response assessment and therapy (COPP versus COP-DAC). Differences in (particularly gonadal) toxicity will be compared.<br>• In the relapse study 5-year EFS rates are described in defined subgroups.
**Schedule**	The study started in Germany on 30.01.2007. For insurance reasons the study accrual in Germany is limited to less than 5 years and an individual follow-up of up to 5 years. Other countries join the study as soon as possible. Overall accrual stops on 29.01.2013.

	Individual follow-up for 5 years after study entry is required for this protocol. Long-term follow-up is strongly recommended and will be organised according to national circumstances.
**Financial support for GPOH-HD group**	**Main support: Deutsche Krebshilfe e.V. / Dr. Mildred Scheel Foundation**

1.5 FLOWCHART OF THE STUDY *(UPDATED 2012-02-2012)*

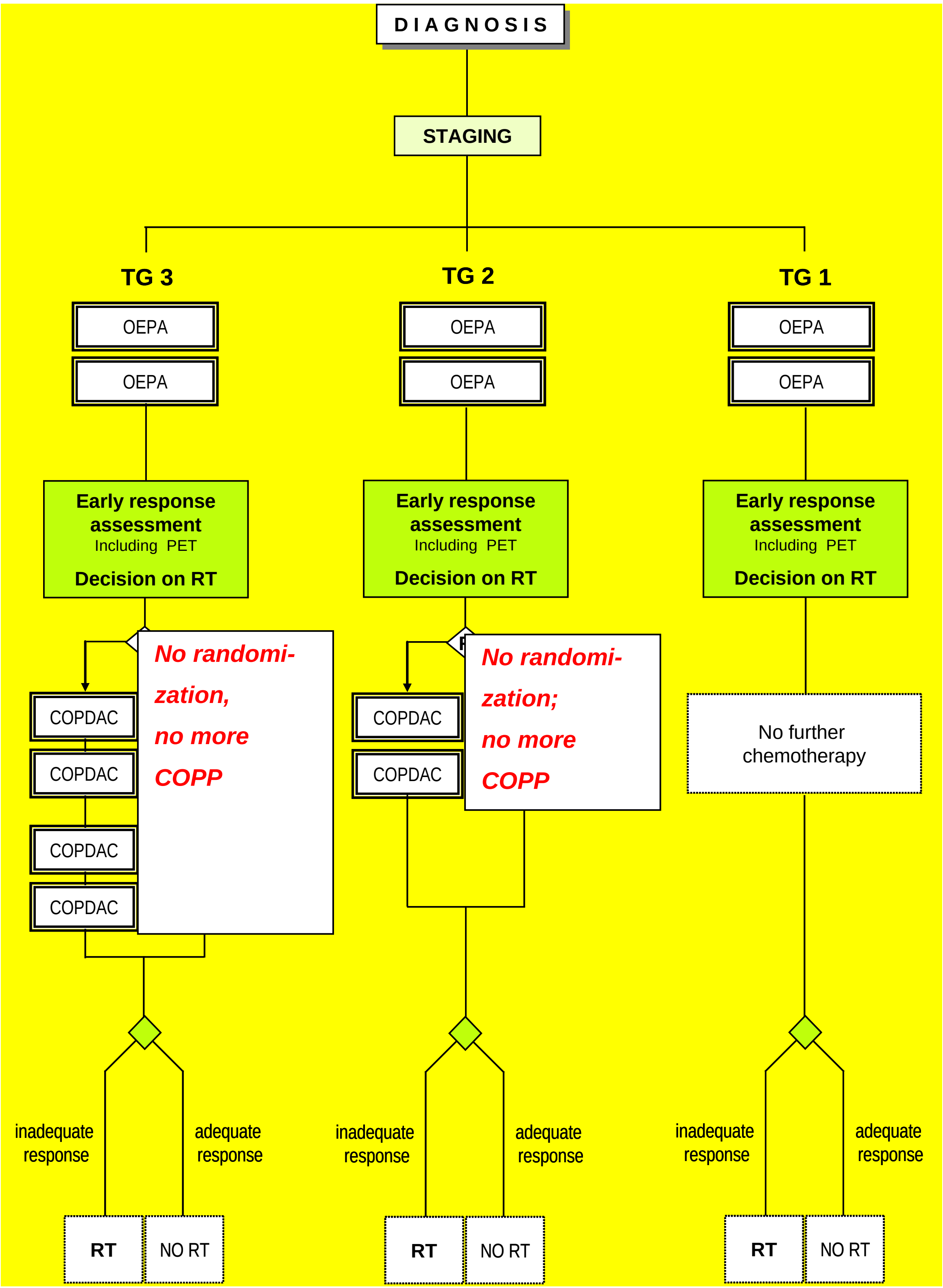

Fig. 1. Flowchart on study proceedings

Flowcharts for patients in relapse therapy can be viewed in chapter 9.

2 STUDY OBJECTIVES

2.1 MAIN GOALS OF THE STUDY

Satisfactory disease control rates (>90%) can be achieved in paediatric Hodgkin's lymphoma with established therapeutic modalities (documented for the GPOH-HD study group since the DAL-HD-82 study).

The remaining challenges for further treatment optimisation are:

- Reduction of acute and long-term toxicity of the chemotherapy and radiotherapy employed.
- Reduction of the amount of treatment in those children who are currently over-treated.

This study aims to eliminate Procarbazine from chemotherapy. Procarbazine is gonadotoxic and may lead to male infertility and premature menopauses. Previous studies (DAL-HD 85 and DAL-HD 87) have shown that Procarbazine is a very effective drug in Hodgkin's Lymphoma and cannot safely be omitted:

- Intensified OEPA using Etoposide instead of Procarbazine replaces OPPA for both boys and girls in the first two cycles.
- COPDAC in which Dacarbazine replaces Procarbazine is compared to standard COPP chemotherapy using a randomised study design.

Both OEPA and COPDAC have been piloted (DAL-HD 90 + GPOH-HD 95 and GPOH-HD 2002 Pilot respectively), are feasible, effective and have an acceptable toxicity profile.

The main question for further therapy optimization is a strategy for treatment adapted to response (STAR), i.e. to tailor the amount of treatment to the individual needs of the patient, safely reduce treatment where not needed, and intensify treatment where indicated.

In the study GPOH-HD 95 the STAR concept was investigated restricting indication for radiotherapy to patients not in complete remission after chemotherapy as assessed by CT/MRI. Radiotherapy is a major cause of secondary malignancies.

The CT/MRI imaging techniques used cannot reliably distinguish between active and fibrotic/necrotic residual masses. Therefore sensitivity (rate of test positive results in true positives) is reasonably high, but the specificity (rate of test-negative results in true negatives) is rather low (in the order of 30%) and a high negative predictive value can only be achieved, if most patients are already cured after chemotherapy.

This was the case in TG-1 in which excellent results in CR patients without radiotherapy were seen. In TG-2 & -3 probably one third of all patients still require radiotherapy for cure. In this setting, patients with radiotherapy did better than patients in CR without radiotherapy.

Meanwhile FDG-PET has become available and is routinely used in most clinics. FDG-PET can better distinguish between vital and fibrotic/necrotic residual masses and thus may resolve the specificity problem of CT/MRI. To further increase sensitivity response assessment should be performed early, e.g. after 2 OEPA.

FDG-PET is now formally integrated into staging and response assessment and used to implement a modified STAR strategy:

- Patients with an adequate response after 2 OEPA (roughly: CR or PR but PET-negative, details see chapter 7.2) will not be irradiated. This holds for all treatment groups.

The objective is to show that 5 year EFS rates in these positively selected patients without radiotherapy are consistent with a target rate of 90%.

On the other hand, patients with inadequate response to 2 OEPA constitute a negatively selected group. If their outcome with radiotherapy turned out not to be consistent with 90% cure, the study would provide an indication for treatment intensification in this sub-group.

In contrast to many other malignant diseases, patients with Hodgkin's lymphoma in first relapse have a realistic chance of cure. The number of relapses is expected to be small.

The treatment strategy is based on previous experience from the DAL-ST-HD-86 study that used IEP-ABVD chemotherapy and involved field radiotherapy. Patients are now stratified based on the dominant prognostic factor "time to first treatment failure" (Schellong 2005). Some patients (late relapse from TG-1) are expected to have very good results with standard 2 (IEP-ABVD) + radiotherapy. High risk patients (progression during or up to three months after first line treatment) have an unsatisfactory prognosis with standard salvage therapy. Therefore in this group high dose BEAM chemotherapy with autologous stem cell transplantation is introduced after 2 (IEP-ABVD). In all other patients (early relapse or late relapse after TG-2 or TG-3) early response assessment with FDG-PET after one double cycle of (IEP-ABVD) decides whether standard chemotherapy with two (IEP-ABVD) is completed with radiotherapy (adequate response) or treatment is intensified with high dose BEAM chemotherapy and autologous stem cell transplantation (inadequate or unclear response).

The objective of the study in second line therapy is to document the outcome with this treatment strategy as quality control and basis for further improvement.

2.2 PRIMARY OBJECTIVES

The primary objectives of the EuroNET-PHL-C1 study are:

(1) Are 5 year event free survival (EFS) rate estimates in patients with adequate response after 2 OEPA treated without radiotherapy consistent with a target event free survival (EFS) rate of 90% in TG-1 and TG-2 & TG-3?

(2) Can Procarbazine be safely replaced by Dacarbazine in therapy groups 2 and 3 without a deterioration of EFS (randomised comparison of COPDAC and COPP)?

(3) Description of treatment outcome to a standardised risk adapted relapse strategy

2.3 SECONDARY OBJECTIVES

In addition, the following secondary study objectives are of interest:

(1) Is the 5 year event free survival (EFS) rate in patients with inadequate response after 2 OEPA who receive standard involved field radiotherapy consistent with a target EFS rate of 90% estimates in TG-1 and TG-2 & TG-3?

(2) Does substitution of Dacarbazine for Procarbazine in TG-2 and -3 patients decrease the rate of infertility in males and premature menopause for females?

2.4 TERTIARY OBJECTIVE

The EuroNet-PHL-C1 study is an inter-group trial. The GPOH-HD group pursues a quality assurance strategy with real time central review of staging and response assessment. The CCLG decided to join this process. Other involved study groups (CCLG and others) do not. Therefore, the exploration of the impact of real-time central staging and response assessment on treatment outcome is a further objective of the trial.

3 RATIONALE OF THE STUDY

3.1 PRIOR EXPERIENCE OF THE DAL / GPOH-HD STUDY GROUP

This study builds on the experience from six successive DAL / GPOH study generations that step by step optimised the treatment of paediatric Hodgkin's lymphoma starting in 1978 and established the de facto treatment standard in the participating countries. For an overview see Table 3.

3.1.1 First study generation DAL-HD 78

Already the first study generation DAL-HD 78 set the general therapeutic paradigm: Chemotherapy starting with 2 courses of intense and effective OPPA (followed by COPP consolidation in intermediate and advanced stages) plus radiotherapy. In 1978 radiotherapy consisted of 36 – 40 Gy to the involved field and 18 – 20 Gy in the adjacent fields (Schellong 1986). Later study generations modified treatment within this framework mainly with the objective to reduce acute and long-term toxicity while preserving good treatment results.

3.1.2 Second study generation DAL-HD 82

In the second study generation DAL-HD 82 (Schellong et al., 1986A), patients for the first time were divided into three treatment groups (TG-1, TG-2, TG-3) based on stage. The number of consolidation COPP cycles was scaled according to treatment group (0, 2, 4 respectively). Irradiation volume was reduced from extended to involved field. Indication for splenectomy was limited and the number of splenectomies dropped to about 40%. Radiation doses were reduced to 35 – 30 – 25 Gy in TG-1, TG-2, TG-3 respectively. In case of insufficient response to chemotherapy the radiation dose was increased by 5 – 10 Gy. 5-year-EFS rates of 99%, 96% and 90% in TG-1, TG-2 and TG-3 respectively (Schellong et al., 1988B) were observed. Due to these excellent results DAL-HD 82 for a long time was regarded as the gold standard.

3.1.3 Third study generation DAL-HD 85

After the gonadotoxic effect of Procarbazine became apparent this drug was completely eliminated from OPPA–COPP chemotherapy in the DAL-HD 85 study generation. Chemotherapy was OPA-COMP, so that in the first two cycles only three agents were administered and Procarbazine was replaced by Methotrexate in consolidation. Involved field radiotherapy was dosed according to TG with 35, 30 or 25 Gy (Schellong et al., 1988). By

eliminating Procarbazine fertility in boys indeed was preserved (Brämswig et al., 1990; Hassel et al., 1991), but treatment efficacy was compromised: For patients with early stages (TG-1) the 10-year EFS rate dropped to 85%, however practically all patients could be salvaged by relapse therapy and an overall survival rate of 98% after 10 years was seen (Schellong et al., 1994 A). For patients in intermediate (TG-2) and advanced stages (TG-3) the 3 year EFS rate dropped to unacceptable 59% and 62% respectively (Schellong et al., 1988A).

3.1.4 Fourth study generation DAL-HD 87

Therefore in the fourth study generation DAL-HD 87 Procarbazine was reintroduced into the COPP cycles while it was still not administered in the OPA. In addition, the radiation dose was further reduced to 30-25-20 Gy for TG-1, TG-2 and TG-3 respectively (Schellong et al., 1994 b). Indication for splenectomy was further restricted so that only in 29% of the patients the spleen was removed. The 7-year EFS and overall survival (OS) rates for all patients (85% and 97% respectively) were better than in DAL-HD 85 but still clearly worse than those of the DAL-HD 82 study generation and were felt to be unsatisfactory.

3.1.5 Fifth study generation DAL-HD 90

Therefore initial therapy in the DAL-HD 90 study was re-intensified. All girls got OPPA again. Boys got OEPA, i.e. OPPA with Procarbazine replaced by 500 mg/m^2 Etoposide given over 4 days, in the hope that this would preserve fertility (Schellong et al., 1999). Splenectomy was abandoned and the radiotherapy dose was further reduced to 25 – 25 - 20 Gy for TG-1-3 respectively.

With this strategy, a 5-year EFS rate of 91% was achieved with OPPA and 89% with OEPA. Overall survival after 5 years was 98% in both groups. The results are comparable with the very good results of the DAL-HD 82 study although therapy intensity was clearly reduced. By introducing Etoposide the infertility rate of boys was significantly reduced (Gerres et al., 1998) in TG-1, while about half of the male patients in TG-2 and TG-3 still showed abnormal FSH values after the COPP cycles.

In the study generation DAL-HD 90 a real-time central review process for all patients was established. Staging, therapy group assignment and response assessment for all patients is performed centrally assessing the clinical data and reviewing all cross-sectional imaging. In about 20% of the patients the stage was revised by central appraisal. 11.7% of patients were assigned to a higher therapy group while 1.6% downstaged to a lower therapy group (Dieckmann et al., 2002).

3.1.6 Sixth study generation GPOH-HD 95

A major concern apart from infertility in boys is the development of secondary malignancies. The rate of secondary haematological malignancies, which occur mostly 1 – 10 years after therapy, is very low. The estimated risk after 15 years is about 1% for the patients in the studies DAL-HD 78 to DAL-HD 90 (Schellong et al., 1997). After the introduction of Etoposide no leukaemias have been reported so far (Schellong 1998, Schellong and Riepenhausen 2002).

On the other hand, the number of non-hematological secondary tumours still increases after a latency period of 20 and more years (Schellong and Riepenhausen 2002). The cumulative risk of secondary solid tumours for the DAL / GPOH-HD study patients is 5.7% after 20 years (standard error (SE) 1.5%). This is almost identical to the 20-year risk for solid tumours reported by the American Late Effects Study Group (LESG). In their study the rate of secondary tumours increased steeply between 20 and 30 years. After 30 years the rate of secondary malignant tumours approached 25% (Bhatia et al., 2003). Secondary solid tumours (SST) are the main cause for this late increase. The most important risk factor for the development of SST is radiation therapy (Meadows et al., 1989). 22 of the 25 SST in our patients occurred in or at the border of the radiation field.

Therefore in the GPOH-HD 95 study generation the dose of radiotherapy was reduced to 20 Gy in all treatment groups. In addition, radiotherapy was omitted in patients with complete remission (CR) at the end of chemotherapy. Event free survival after 5 years was 88% for all patients; overall survival 97% (Dörffel et al., 2003).

In TG-1 there was no significant EFS difference between patients with (94%) and without (97%) radiotherapy. Therefore omission of radiotherapy in CR patients is adopted as standard treatment.

However, in TG-2 and TG-3 omission of radiotherapy for CR patients lead to a significant decrease in EFS (without radiation 79%, with radiation 91%). Therefore radiotherapy for all remained standard.

3.1.7 GPOH-HD 2002 Pilot study

In GPOH-HD 2002 Pilot, all boys received an intensified OE*PA therapy (20% more Etoposide) and COPDAC instead of the COPP cycles.

*3.1.7.1 Rationale for intensified OE*PA and toxicity results*

In the previous studies DAL-HD 90 and GPOH-HD 95, boys showed a tendency for worse EFS than girls. In the GPOH-HD 95 study boys had significantly worse 5 years DFS rates than girls (0.86 vs. 0.93%; p=0.006). This may or may not be related to girls receiving OPPA and boys receiving less gonadotoxic OEPA. Male gender has been reported as an unfavourable prognostic factor in the adult setting and is in the international prognostic score (Hasenclever 1998).

Based on the interpretation that OEPA was less effective than OPPA, OEPA was intensified in the GPOH-HD 2002 Pilot study extending etoposide administration from 4 to 5 days.

The total etoposide dose increased from 4 to 5 x 125 $mg/m^2/d$ as 2-hour infusion per OEPA cycle. This corresponds to a cumulative dose of 1250 mg/m^2 in 2 OE*PA cycles which is still far below the critical cumulative dose of 2000 mg/m^2 above which an increased risk of secondary AML was described (Kollmannsberger et al., 1998; Whitlock et al., 1993; Winick et al., 1993). Internationally, the increased cumulative Etoposide dose is still below that of other studies, e.g. the VBVP cycles of the French study MHD 90 (SD 167 mg/m^2, cum. dose 2000 mg/m^2) or the VEEP cycles of the Australian – New Zealand study (SD 150mg, cum. dose 3000 mg/m^2).

Feasibility of intensified OE*PA was confirmed based on 510 OE*PA blocks for 255 patients from a total of 308 registered male patients (status: 01.08.2005). Apart from the expected haematoxicity (Table 1) CTC degree 3 or 4 toxicity were rare.

Table 1 Toxicity evaluation for OE*PA in the GPOH-HD 2002 Pilot Study

According to CTC criteria of the NCI; for 510 OEPA blocks documented so far

Toxicity	Number of documented incidences					
	Not documented	0	I°	II°	III°	IV°
Performance status	47	179	200	73	11	2
Haemoglobin	9	119	164	179	34	5
Leukocytes	1	27	63	136	208	75
Neutrophils	120	35	28	35	105	187
Platelets	1	447	25	29	5	3
Infection	52	355	35	55	12	1
Fever	7	377	64	55	7	0
Nausea	184	197	95	31	2	1
Vomiting	7	359	85	59	0	0
Stomatitis	3	339	74	64	22	8
Constipation	11	368	37	77	17	0
Diarrhoea	10	428	50	16	6	0
Allergy	192	312	3	3	0	0
Pain	179	249	48	27	6	1
Neurotoxicity sensory	13	434	52	7	2	2
Neurotoxicity motor	13	449	32	10	6	0
Neurotoxicity central	194	310	4	0	0	2
Cardiotoxicity	291	214	2	2	0	0
Creatinine	17	465	23	5	0	0
Proteinuria	256	246	8	0	0	0
Haematuria	251	251	8	0	0	0
Creatinine clearance	365	140	3	1	0	0

3.1.7.2 Rationale for COPDAC and toxicity results

The experiences of previous studies show that Procarbazine is mainly responsible for the infertility of treated boys. A Procarbazine dose-dependent increase of hormonal disturbances was described: 28.9% in patients after two OPPA, 43.8% after two OPPA and two COPP and 62.5% after two OPPA and 4 COPP cycles (Brämswig et al., 1990).

The ratio of girls delivering a child to boys fathering a child in patients from the DAL-HD90 study was 2.6 in TG-1, in which male patients got less or no Procarbazine, compared to 5.3 in TG-2/TG-3. This data may be slightly biased since the reproduction age of men is higher than that

of women. On the other hand no disturbance was observed in boys who had been treated in the DAL-HD 85 study or who had only received OEPA in the DAL-HD 90 study (Gerres et al.1998).

Therefore in GPOH-HD 2002 Pilot Procarbazine was replaced by Dacarbazine which is less likely to cause infertility in males and a premature menopause in females. Our previous studies DAL-HD 82 and DAL-HD 87 showed that Procarbazine cannot be dropped without being replaced by an appropriate substitute.

A literature research showed that Dacarbazine (DTIC) could be an appropriate substitute for Procarbazine (Frei et al., 1972; Klener et al., 1977). Dacarbazine acts like Procarbazine as an alkylating agent and inhibits both the DNA and the RNA synthesis. It is an approved drug which has been extensively used in Hodgkin's lymphoma. Even after 6 ABVD cycles male patients are unlikely to become azoospermic.

In contrast to Procarbazine, DTIC is administered intravenously. This reduces another possible problem – drug non-compliance.

In 1972 Dacarbazine was tested by Frei as a monotherapeutic in pre-treated patients. He achieved „objective remissions“ in 56% at a daily dosage of 250 mg/m² for 5 days at intervals of 3 weeks. Klener and Donner (1977) treated 10 GPOH-HD patients, who had become resistant to combination chemotherapy, with DTIC, 300 mg/m^2 i.v. for 5 days running at an interval of 4 weeks which led to CR in 2 patients and PR in 7 patients. They particularly emphasized that DTIC causes only a minor myelosuppression. Both publications put together, „objective remission“ was achieved in 67% and complete remission in 13%. Since a response rate of 69% (38% CR) was achieved with a dose of 3000 mg Procarbazine, administered in 3-week intervals in 366 patients (Carter et al., 1973) an approximate equivalence factor of about 2.4 may be assumed. Thus a dose of 750 mg/m² Dacarbazine used in the proven ABVD cycles would correspond in effectiveness to approximately 1800 mg/m^2 Procarbazine. It should therefore be equivalent to the Procarbazine dose of 1400 mg/m^2 applied in the COPP cycle. In this protocol DTIC is to be administered over three days at the beginning of the COPDAC cycle in a dose of 250 mg/m^2 in 30-minute infusions to achieve the highest possible dose intensity.

So far the evaluation of chemotherapy toxicity is available for 473 COPDAC blocks of 170 male patients (status: 01.06.2005). The toxicity profile is detailed in Table 2.

Table 2 Toxicity evaluation for COPDAC in the GPOH-HD 2002 Pilot Study

According to CTC criteria of the NCI of 473 COPDAC blocks documented so far

Toxicity	Number of documented incidences					
	Not documented	0	I°	II°	III°	IV°
Performance status	59	230	159	23	1	1
Haemoglobin	6	208	177	67	15	0
Leukocytes	5	207	151	83	25	2
Neutrophils	104	183	80	62	31	13
Platelets	5	448	14	3	2	1
Infection	38	393	24	14	4	0
Fever	10	407	32	22	2	0
Nausea	140	281	51	1	0	0
Vomiting	4	438	21	10	0	0
Stomatitis	2	434	35	2	0	0
Constipation	12	411	15	33	2	0
Diarrhoea	11	452	8	2	0	0
Allergy	164	300	7	1	1	0
Pain	156	269	24	13	11	0
Neurotox. sensorial	10	378	71	10	4	0
Neurotox. motorical	12	408	46	6	1	0
Neurotox. central	170	300	0	0	0	3
Cardiotoxicity	353	118	2	0	0	0
Creatinine	20	423	27	3	0	0
Proteinuria	259	208	6	0	0	0
Haematuria	252	217	4	0	0	0
Creatinine clearance	346	121	6	0	0	0

These preliminary data indicate that the COPDAC block and with it the DTIC are well tolerated.

One SUSAR (suspected unexpected serious adverse reaction) was reported most probably related to DTIC. The patient developed a delayed type hypersensitivity reaction after the second COPDAC cycle. Consequently he presented with acute renal failure, shock and rhabdomyolysis. In the literature (Hauschild et al., 2001) only few cases of irreversible acute renal failure have been described after treatment with DTIC. However, as to our knowledge this is the first case of severe rhabdomyolysis possibly related to DTIC. The patient died of progressive multi-organ failure.

Thus preliminary results of the GPOH-HD 2002 Pilot protocol show that the 2 OE*PA + 2 or. 4 COPDAC treatment is feasible with an acceptable toxicity that is comparable to 2 OPPA + 2 or. 4 COPP. An interim analysis on efficacy in the GPOH-HD2002 pilot study was performed as of 2006-10-31. At a median observation time of 24 months PFS curves of girls (N=199) receiving OPPA-COPP and boys (N=205) receiving OEPA-COPDAC in TG-2+TG-3 are superimposable with PFS rates 90% (se=2%) at 24 months. This is in line with the experience of previous studies. Based on 2 year PFS-rates OPPA-COPP and OEPA-COPDAC appear to be exchangeable treatment options for TG-2+3. No Etoposide induced secondary leukaemias were observed.

Table 3 Treatment evolution since DAL HD 78 up to EuroNet-PHL-C1

(a) for patients of therapy group 1 (TG-1)

**Study**	**Stages in TG-1**	**Chemotherapy**	**Radiotherapy**
DAL-HD 78	I, IIA	2 x OPPA	36 – 40 Gy extended field
DAL-HD 82	I, IIA	2 x OPPA	35 Gy involved field
DAL-HD 85	I, IIA	2 x OPA	35 Gy involved field,
DAL-HD 87	I, IIA	2 x OPA	30 Gy involved field,<br>boost for remaining tumour: 35 Gy
DAL-HD 90	I, IIA	girls: 2 x OPPA<br>boys: 2 x OEPA	25 Gy involved field;<br>boost for remaining tumour:<br>30/35 Gy
GPOH-HD 95	I, IIA	girls: 2 x OPPA<br>boys: 2 x OEPA	CR: no radiotherapy<br>non-CR 20 Gy involved field; resaturation for remaining tumour: 30/35Gy
GPOH-HD 2002 Pilot	I, IIA	girls: 2 x OPPA<br>boys: 2 x OE*PA	CR no radiotherapy<br>non-CR 20 Gy involved field; boost for remaining tumour: 30/35Gy
EuroNet-PHL-C1	I, IIA	Girls and boys: 2 x OE*PA	CR or non-CR but PET negative: no radiotherapy<br>All others: 20 Gy involved field; boost for remaining tumour: max. 30Gy

* raised Etoposide dose

(b) for patients of therapy group 2 (TG-2)

Study	Stages in TG-2	Chemotherapy	Radiotherapy
DAL-HD 78	> IIA	2 x OPPA, 4 x COPP	36 – 40 Gy extended field
DAL-HD 82	IIB, IIIA	2 x OPPA, 2 x COPP	30 Gy involved field,<br>boost for remaining tumour: 35Gy
DAL-HD 85	IIB, IIIA	2 x OPA, 2 x COMP	30 Gy involved field,<br>boost for remaining tumour: 35Gy
DAL-HD 87	IIB, IIIA	2 x OPA, 2 x COPP	25 Gy involved field,<br>boost for remaining tumour: 35Gy
DAL-HD 90	IE, IIB, IIEA, IIIA	girls:<br>2 x OPPA, 2 x COPP<br>boys:<br>2 x OEPA, 2 x COPP	25 Gy involved field;<br>boost for remaining tumour: 30/35Gy
GPOH-HD 95	IE, IIB, IIEA, IIIA	girls:<br>2 x OPPA, 2 x COPP<br>boys:<br>2 x OEPA, 2 x COPP	CR no radiotherapy,<br>non-CR 20 Gy involved field; boost for remaining tumour: 30/35Gy
GPOH-HD 2002 Pilot	IE, IIB, IIEA, IIIA	girls:<br>2 x OPPA, 2 x COPP<br>boys:<br>2 x OE*PA, 2 x COPDAC	all: 20 Gy involved field; boost for remaining tumour: 30/35Gy
EuroNet-PHL-C1	IE, IIB, IIEA, IIIA	Girls and boys:<br>2 x OE*PA and<br>2 x COPP or COPDAC	No radiotherapy for patients in at least PR and PET negative after two cycles of OEPA;<br>20 Gy involved field; boost for remaining tumour: max. 30Gy

(c) for patients in therapy group 3

Study	Stages in TG-3	Chemotherapy	Radiotherapy
DAL-HD 78	like TG-2	2 x OPPA 4 x COPP	36 – 40 Gy extended field
DAL-HD 82	IIIB, IV	2 x OPPA, 4 x COPP	25 Gy involved field,<br>boost for remaining tumour: 30Gy
DAL-HD 85	IIIB, IV	2 x OPA, 4 x COMP	25 Gy involved field,<br>boost for remaining tumour: 30Gy
DAL-HD 87	IIIB, IV	2 x OPPA, 2 x COPP	25 Gy involved field,<br>boost for remaining tumour: 30 Gy
DAL-HD 90	IIEB, IIIB, IIIEA, IV	girls:<br>2 x OPPA, 4 x COPP<br>boys:<br>2 x OEPA, 4 x COPP	20 Gy involved field;<br>boost for remaining tumour: 30/35Gy
GPOH-HD 95	IIEB, IIIB, IIIEA, IV	girls:<br>2 x OPPA, 4 x COPP<br>boys:<br>2 x OEPA, 4 x COPP	IF-RT: non-CR 20 Gy, max. 35 Gy
GPOH-HD 2002 Pilot	IIEB, IIIB, IIIEA, IV	girls:<br>2 x OPPA, 4 x COPP<br>boys:<br>2 x OE*PA, 4 x COPDAC	all: 20 Gy involved field;<br>boost for remaining tumour: max. 30Gy
EuroNet-PHL-C1	IIEB, IIIB, IIIEA, IV	Girls and boys:<br>2 x OE*PA and<br>4 x COPP or COPDAC	No radiotherapy for patients in at least PR and PET negative after two cycles of OEPA;<br>20 Gy involved field; boost for remaining tumour: max. 30Gy

* raised Etoposide dose

3.2 FORMAL INTEGRATION OF FDG-PET IMAGING INTO STAGING AND RESPONSE ASSESSMENT

Functional FDG-PET imaging was increasingly used in Hodgkin’s lymphoma already during GPOH-HD 95. It is now routinely used in most GPOH centres.

FDG-PET can better distinguish between vital and fibrotic/necrotic residual masses and thus may resolve the problem of low specificity with CT/MRI (compare Table 4).

Table 4 Comparison of the significance of CT/MRI and FDG-PET for the assessment of therapy success

**Reference**	**FDG-PET**		**CT/MRI**	
	sensitivity	*specificity*	*sensitivity*	*specificity*
Lang (2001)	95%	89%	95%	42%
De Wit (2001)	100%	78%	70%	26%
Spaepen (2001)	50%	100%	70%	28%
Dittmann (2001)	88%	94%	25%	56%

In the EuroNet-PHL-C-1 study FDG-PET is formally integrated into staging and response assessment. In order to safeguard against possible uptake artefacts and to avoid a major shift in staging results, the integration of FDG-PET results into staging and response assessment is based on two principles:

1. All functional FDG-PET information formally used in staging and response assessment must be paired with findings on conventional imaging.
2. At diagnosis FDG-PET results are only used to decide on involvement in regions that are suspicious but inconclusive by conventional imaging.

For details of how these principles are implemented see chapter 6.

Selected results of a FDG-PET literature review (Körholz et al. 2003) and the experience with these definitions within the GPOH-HD-2002 Pilot study is given below.

3.2.1 Relevance of FDG-PET for the initial staging in Hodgkin's lymphoma

FDG-PET can image the entire body detecting peripheral metastatic lesions. Several papers have demonstrated that more lesions are identified by FDG-PET than with CT/MRI. This may change the stage and thus treatment group for some patients (Table 5).

15% of patients in adult studies have lesions seen on conventional imaging which are negative on initial FDG-PET. In such cases local response assessment by FDG-PET is not valid. This is further discussed in chapters 3.3, 6 and 8.

Table 5 FDG-PET integrated into initial staging in Hodgkin's lymphoma

Reference	Modifications by FDG-PET		
	Upstaging	Downstaging	Therapy modification
Partridge et al. (2000)	18/44	3/44	11/44
Weidmann et al.(1999)	3/20	0/20	ND
Weihrauch et al. (2002)	4/22	ND	1/22
Jerusalem et al. (2001)	3/33	4/33	0/33
Montravers et al. (2002)	4/7	0/7	1/7

ND not defined

In the pilot study GPOH-HD 2002 200 children were staged with FDG-PET between November 2002 and March 2005. A total of 5400 possible regions of involvement were assessed (27 areas per patient) using the assessment rules detailed in chapter 6. In 302 / 5400 areas either CT/MRI or (rarely) FDG-PET was missing or invalid due to artefacts or both methods had unclear results, so that the respective involvement had to be decided by additional criteria.

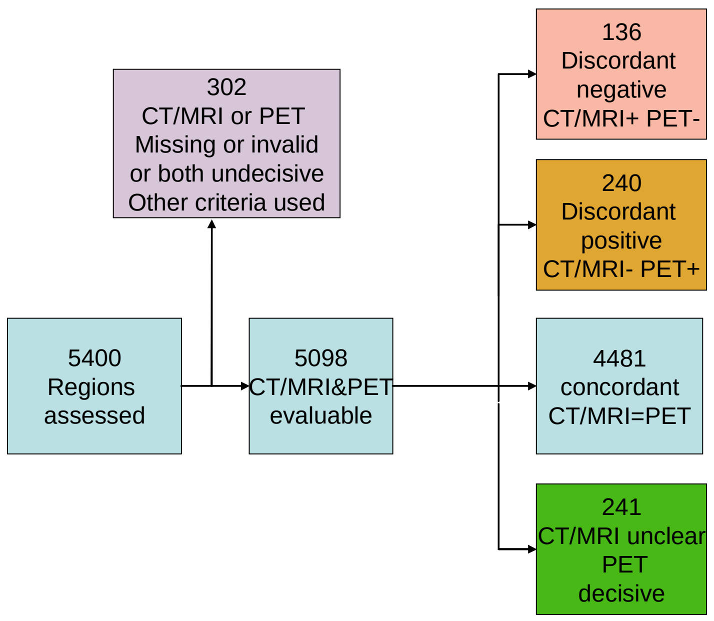

Fig. 2. FDG-PET staging results

In regions in which both CT/MRI and PET were evaluable and conclusive, FDG-PET results were concordant in 4481/4857 (92.3%), discordantly positive in 240/4857 (4.9%) and discordantly negative in 136/4857 (3.0%) cases. In 241 regions CT/MRI was unclear and FDG-PET decided on the involvement following the principles outlined above.

Concordance rates were below 90% in the following regions: high-cervical, cervical, lung hilum, and supra-diaphragmatic recessus.

Aggregating on patient level,

- 119/200 (59.5%) patients had at least one region in which CT was unclear and involvement was thus decided by FDG-PET. But this region was below the diaphragm in only 28 patients (14%). Thus upstaging was limited.
- 88/200 (44%) patients had at least one region in which CT/MRI was negative, but PET positive. According to the principles outlined above these regions were considered uninvolved.
- 115/200 (57.5%) patients had at least one region in which CT/MRI was clearly positive, but PET negative. These regions pose a problem for early response assessment with FDG-PET because zero-conversion cannot be observed

These data demonstrate that integration of FDG-PET into staging according to the principles exposed above is feasible without major stage shifts. But for reproducible results **strict adherence to a common decision algorithm** (see chapter 6) is required.

3.2.2 Timing of FDG-PET response assessment after chemotherapy

It is known that false negative (Cremerius et al., 1998) and false positive FDG-PET can be seen after chemotherapy. However it is important to minimize delay between chemotherapy cycles. The timing of FDG-PET is therefore crucial.

The EORTC recommendations (Young et al., 1999) state that at least 2 weeks should elapse after chemotherapy prior to FDG-PET. Therefore in this study the early response assessment FDG-PET is scheduled on day 14 (preferably until day 17) after the last chemotherapy application, i.e. d28 -31 of the second OEPA cycle (cp. section 10.2.1).

3.2.3 FDG-PET in response assessment at the end of treatment

FDG-PET for response assessment of Hodgkin's lymphoma has been studied in adult patients. Literature review of papers differentiating between Hodgkin's lymphoma and NHL shows the sensitivity of FDG-PET (the probability of true identification of patients with later relapse) is in the order of 50 to 100%. Specificity (the probability of true identification of patients without later relapse) was 64 to 100%. In the studies listed in Table 6, 187 patients were imaged after completion of therapy. The average follow-up period was more than 20 months. True positive FDG-PET findings were seen in 28/187 patients, in 137/187 patients FDG-PET was true

negative, in 13/187 patients false positive and in 9/187 patients false negative. Pooling all data gives a sensitivity of 75% and a specificity of 91.3%.

Table 6 FDG-PET results in late assessment of therapy

Reference	Follow-up period median / range	Time interval*	Case number #	Sensitivity	Specificity
Stumpe (1998)	ND/>6	ND	43	86	96
Lang (2001)	22,5/5 – 43	4 – 8	63	95	89
De Wit (2001)	25,6/ 2 – 45	10+/-9	33	100	78
Weihrauch (2001)	28/16 – 68	> 4**	29	67	80
Spaepen (2001)	32/ND	4 – 12	60	50	100
Naumann (2001)	37/15 – 58	1 – 24	43	100	64
Dittmann (2001)	ND/>6	>3	26	88	94

patients or PET examinations

*time interval after last therapy in weeks or months (**)

ND not defined

3.2.4 Early response assessment with FDG-PET

Data on early response assessment is relatively rare and not easily interpretable since often HL and NHL cases are pooled and details of treatment and general prognosis have to be taken into the account. The following table taken from Hutchings et al 2004 summarizes the literature on early response assessment in HL and NHL (Table 7).

Table 7 Early response assessment with PET (from Hutchings et al, 2004)

First author	Year	Chemotherapy before PET	Patients	PET-positive		PET-negative		Follow-up
				Total	Treatment failure	Total	Treatment failure	
Hoekstra	1993	1-2 cycles	13 HL / 13 NHL	10	7 (70%)	16	1 (6%)	6-20 months
Mikhaeel	2000	2-4 cycles	23 NHL	8	7 (88%)	15	0 (0%)	7-56 months
Spaepen	2002	3-4 cycles	70 NHL	33	33 (100%)	37	6 (16%)	2-62 months
Jerusalem	2000	2-3 cycles	28 NHL	5	5 (100%)	23	7 (30%)	4-46 months
Kostakoglu	2002	1 cycle	13 HL / 17 NHL	15	13 (87%)	15	2 (13%)	5-24 months

During the GPOH-HD 2002 Pilot study a certain proportion of patients underwent early FDG-PET response assessment. Although this group might represent a slightly negative selection of the total patient population we can now crudely assess how many patients will be PET-negative

after 2 cycles of OPPA resp. OEPA (Table 8):

Table 8 Early response assessment with FDG-PET after 2 OPPA or OEPA in GPOH-HD 2002 Pilot

After 2 cycles	TG-1	TG-2	TG-3	TG-2 + TG-3
PET-negative	27/41 (66%)	5/16	8/23	13/39 (33%)

As far as TG-1 is concerned, GPOH-HD 95 showed that radiotherapy can safely be dropped in about 30% of all TG-1 patients, namely in CR after chemotherapy. According to the pilot data, FDG-PET-based response evaluation has the prospect to avoid radiotherapy in about 60 – 70% of all patients with early stage HL.

The observed test-negative rate of about 2/3 agrees well with what we earlier expected (based on sensitivity and specificity guesstimates from the literature and the presumption that more than 80% of the TG-1 patients are already cured before radiotherapy which is based on a model-based interpretation of the DAL-HD 90/GPOH-HD 95 results. So the pilot data strengthen the expectation that in TG-1 radiotherapy can safely be omitted in PET-negative cases.

In TG-2+3 we may anticipate that about 1/3 of patients will be PET negative after 2 OEPA and about 50% (40/77) after full chemotherapy (i.e. additional 2 respective 4 cycles of COPP or COPDAC).

A PET-based STAR strategy to limit the indication for radiotherapy can be implemented

- late - just before radiotherapy or
- early on - e.g. after 2 OEPA.

Given

- the unsatisfactory results of GPOH-HD 95 in TG-2 +TG-3 (5yr-EFS 79% with no radiotherapy in the about 20% patients in conventional CR after full chemotherapy) and
- the observation that patients in conventional CR after full chemotherapy rarely will be still PET positive,

a late PET-based STAR strategy does not appear promising.

In addition, based on a model-based interpretation of the DAL-HD 90/GPOH-HD 95 results we expect about 35% of patients in TG-2 + TG-3 to require radiotherapy after full chemotherapy.

To use FDG-PET for late response adaptation would require sensitivity in the upper range of the literature results cited above.

However, there are reasons to expect better discrimination with early PET after only 2 cycles: Sensitivity of PET is a function of the vital tumour mass still present. In patients that are not yet cured before radiotherapy there is a risk that rather small residual tumour masses lie under the detection limit. Earlier on (after two cycles) these masses are still larger. Thus early response assessment should have a higher sensitivity than a late one.

Predictive specificity of early PET-based response assessment must be lower than specificity of late assessment because some patients who are positive after two cycles should be expected to be cured and converted to PET-negative by the additional chemotherapy. Thus using early FDG-PET response assessment after 2 OEPA to define the indication for radiotherapy appears to be promising.

During the final editing stages of the protocol this hypothesis was supported by a prospective Danish study (Hutchings 2006) in N=74 adult HL patients mainly treated with ABVD: 3 of 61 patients with negative FDG-PET after 2 cycles progressed within 3 years, while 11 of 16 patients with positive FDG-PET progressed and two died.

3.2.5 Stop of Randomisation

Further to a Clinical Board Meeting of EuroNet-PHL and the Annual EuroNet-PHL Clinicians Meeting (from 09.02.2012 to 11.02.2012), a decision was made to end the randomisation between COPP and COPDAC for eligible Patients in the EuroNet-PHL-C1 trial. This decision was made on the basis of the results of the second interim data analysis which showed emerging evidence that COPP and COPDAC are similarly efficacious.

The interim results are consistent with data from the non-randomised GPOH-HD-2002 study (Mauz-Körholz et al. , J Clin Oncol. 2010) which showed that boys with OEPA-COPDAC did equally well as girls with OPPA-COPP. Historically, girls tended to have slightly more favourable outcomes than boys.

The randomised data from EuroNet-PHL-C1 is still immature. However, a sample size of 880 randomised Patients (who have completed treatment) has been realised, which is within the range of what was deemed adequate in the protocol (700-1100) to answer the randomised question on efficacy. Randomisation in Germany had already stopped on 30.01.2012 (for insurance reasons) and randomisation in all other countries was scheduled to end on 31.01.2013.

Prior to the start of EuroNet-PHL-C1, it was known that chemotherapy regimens containing Procarbazine tended to reduce semen concentration and quality in a dose dependent manner which may lead to azoospermia. Data on FSH in EuroNet-PHL-C1 confirms this expectation, showing that FSH (which is negatively correlated to sperm count/concentration) is elevated, compared to normal values, after treatment with COPP.

COPDAC was a regimen specifically designed to avoid gonadal damage in boys by replacing Procarbazine with Dacarbazine. Emerging data from EuroNet-PHL-C1 indicates that the FSH distribution after treatment with COPDAC remains in the normal range. FSH after COPP is clearly higher than after COPDAC ($p<0.001$).

In addition, there is early evidence of a recovery in FSH values after COPDAC, but this is not the case after treatment with COPP. Data on semen analyses post chemotherapy are still sparse, but azoospermia was observed in 2/3 Patients who had COPP and in 0/4 Patients who had COPDAC.

In view of the evidence that emerged from the interim data analysis of EuroNet PHL-C1 the Clinical Board and associated parties saw a change in the risk-benefit assessment for the trial and decided that randomisation should be stopped on 13.02.2012. Although the data was immature, it was clear that the sample size was sufficient to answer the randomised question on efficacy and that furthermore it was essential to avoid the potentially detrimental effects of COPP on male fertility.

All Patients in EuroNet-PHL-C1 (in TG-2 and TG-3) should now receive COPDAC.

The trial remains open to recruitment (outside of Germany) because that data is required to fully answer the other important questions in the protocol relating to the STAR approach to treatment and whether it is safe to omit radiotherapy.

3.2.6 Identification of a high risk group in TG1

At the meeting of the Clinical Board of EuroNET-PHL in Paris on the 9th and 10th November 2012 the study biometrician, presented the third interim analysis of the EuroNet-PHL-C1 trial.

Trial recruitment has been excellent with over 2000 patients entered into the study. The study closed in Germany on the 29th January 2012 and is due to close in all participating countries on the 30th January 2013. The formal stopping rules for the trial have not been met.

Nevertheless, there is a sub-group result in the third interim analysis that led the Clinical Board to decide that the risk-benefit analysis for EuroNet-PHL-C1 has changed:

On the occasion of the second interim analysis in 2011 we analysed the prognostic homogeneity of the treatment group one (TG-1) because study groups in the adult setting use a different treatment delineation leading to a markedly smaller proportion of patients treated with chemotherapy limited to two cycles.

With prognostic factors used by the other study groups, we found that patients in TG-1 with a high ESR ≥ 30 mm/h and/or a bulk volume ≥ 200 mls had a worse prognosis (EFS approx 80%) when compared to TG-1 patients without these risk factors (EFS 97%).. At the time it was decided that these results were immature and had to be confirmed by more patients and longer follow up in the next interim analysis.

The third interim analysis now confirms this finding beyond reasonable doubt.

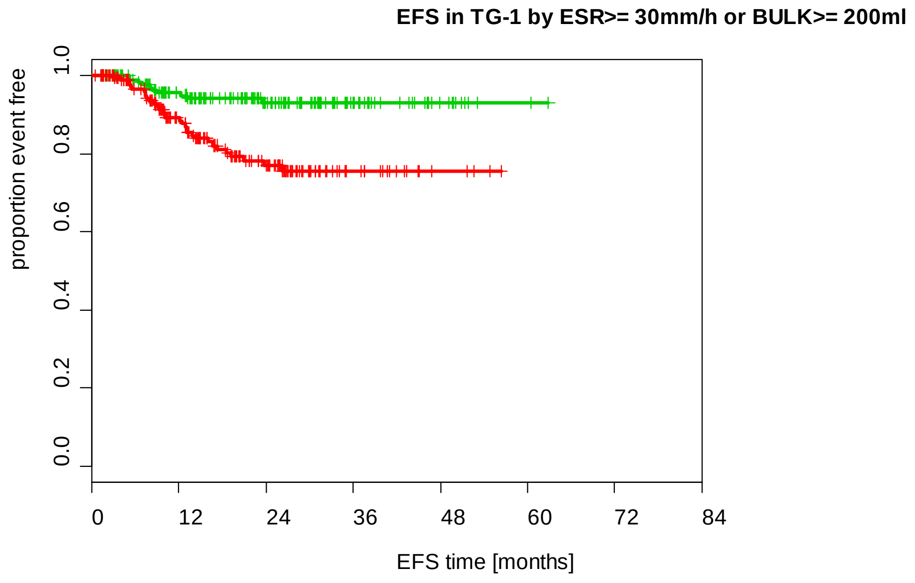

Based on the excellent results in TG-2 (comprising patients with a higher disease burden) and the experience of several other study groups, the Clinical Board feels confident that TG-1 patients with bulky disease and /or ESR>30mm/hr will have an outcome comparable to TG-1 patients without these risk factors.

3.3 MODIFICATIONS IN RELATION TO GPOH-HD 95

3.3.1 Modifications for all patients

3.3.1.1 Intensified OEPA for both boys and girls

Girls as well as boys will receive intensified OEPA (5 days of etoposide). Intensified OEPA was introduced for boys in the GPOH-HD 2002 Pilot study and was shown to be effective and to have acceptable toxicity.

Using OEPA also in girls as well as in boys simplifies the treatment schema. In addition, oral medication and the associated risk of non-compliance is avoided and risk of premature menopause may be reduced.

For younger women there is a risk of premature ovarian insufficiency and premature menopause after chemotherapy. Long-term assessment of female adolescents, who received radiation therapy below the diaphragm and / or chemotherapy with alkylating agents in the scope of cancer therapy, show that the rate of premature menopause is increased in comparison to healthy volunteers (Byrne et al., 1992). This seems to apply also for women who have been treated with COPP for Hodgkin's lymphoma. Kreuser et al. (Kreuser et al., 1987) described ovarian insufficiency and premature menopause after COPP chemotherapy in 2/7 women who were under the age of 24 at the time of chemotherapy and in 6/7 women who were older than 24 at the beginning of treatment.

3.3.1.2 Modifications in radiotherapy

Radiotherapy indication: Given the widespread use of FDG-PET and the potential of early FDG-PET based response assessment as discussed in 3.2, a major objective of this new first line study generation is to clarify the role of FDG-PET for STAR: In all three treatment groups the indication for radiotherapy will be based on early response assessment with CT/MRI and FDG-

PET after 2 OEPA. Patients with adequate response after two OEPA (i.e. CR or at least PR but PET-negative, details of response definitions see 7.3) will not be irradiated.

Radiation fields and technique: According to the GPOH experience opposed field radiotherapy is performed to all initially involved lymph nodes with a safety margin of 1-2cm. CT based 3D-dose calculation is now recommended for all children.

Radiation dose: During the past studies the radiation dose has been reduced gradually. In case of incomplete response after 2 cycles of chemotherapy all primarily involved lymph nodes now receive a dose of 19.8Gy. All patients with poor response get an additional boost of 10 Gy.

Indication for radiation boost: Boost radiotherapy is based on response after two cycles of chemotherapy which applies now for all treatment groups.
Poor response is defined as follows:

- residual volume is larger than 25% of initial volume (i.e. <75% response)
- and the residual volume is more than 5 cm^3.

  or
- residual volume is >100 cm^3

3.3.2 Randomised comparison of COPP and COPDAC in TG-2+3

In TG-2 + TG-3 patients will be randomised to receive either COPP or COPDAC chemotherapy after 2 OEPA. COPDAC was piloted in GPOH-HD 2002 Pilot (compare 3.1.7) is feasible, effective and had an acceptable toxicity profile. Rationale for COPDAC is extensively described in 3.1.7.

On 13.02.2012 the randomisation was stopped. The rationale for stopping randomisation is given in Section 3.2.5. Treatment will be continued with COPDAC

3.4 FURTHER EXPERIENCE OF PARTICIPATING STUDY GROUPS

3.4.1 Response-based strategies in Hodgkin Lymphoma: Experience of the SFCE (Société Française de Lutte contre le Cancer de L'Enfant et de l'adolescent)

The French SFCE study group ran two studies MDH82 (1982-1988) and MDH90 (1990-1997) in stage I-III patients with the objective of limiting late effects after cure of Hodgkin Lymphoma children. During the same period of time patients with stage IV Hodgkin lymphoma were treated in the SIOP 87 study based on the German strategy DAL-HD 90.

Protocol MDH 82 (82-88) was reported in 92 (Oberlin et al, J Clin Oncol, 1992, 10, 1602-1610). Patients (n=136) with stage I-IIA received either two double cycles of MOPP/ABVP or 4 ABVP and 20Gy involved fields or additional boost up to 10Gy in case of poor response to CT. In advanced stages I-II B, III A/B patients received 3 MOPP/ABVP cycles and 20Gy involved fields or additional boost up to 10Gy if poor response. Two hundred and thirty eight patients were included in MDH82 study, 95% of the patients achieved good response with over 70% reduction volume whatever the stage. The 10 year overall disease free survival was 90% in stage I and II A and identical in the 2 randomized groups. In advanced stage DFS was 86 %. Whatever the stage, the only prognostic factor was the response to primary chemotherapy (DFS 89%vs 18%). One of the major conclusions of the study was that if good response is achieved after efficient CT, 20 GY involved field irradiation is enough to consolidate remission.

MDH 90 (90-96) was reported in 2000 (Landman-Parker J et al, J Clin. Oncol 2000, 18, 1500-07). In order to go further in de-escalating treatment and avoiding related sequelae the SFCE investigated treatment by VBVP (Vinblastin, VP 16, Prednisone, Bleomycin) without anthracyclines nor alkylating agents in stage I and II (65%of all the patients) and a response based adapted strategy with OPPA cycle for patients who were poor responders (less than 70% of reduction volume) after 4 cycles of VBVP. All patients received 4 VBVP cycles followed for good responders by 20Gy IF (and lombo splenic field if SR was >70). For patients with poor response the same strategy was applied after one or 2 cycles of OPPA. 202 patients were enrolled in the study. Overall survival was 97% and DFS 91%. 85% of the patients were good responders to VBVP; 6 yr DFS of good and poor responding patients was respectively 92.5% and 77% (p = 0.06). In multivariate analysis three prognostic factors were identified: anaemia with Hb <10.5G/l, biologic inflammatory features, and nodular sclerosis histology. Overall 90% of the patients received 20Gy IF and 10% boost up to 10Gy on mediastinum. Conclusion of MDH 90 study: Response adapted strategy was able to avoid over-treatment in a large proportion of patients with stage I and II Hodgkin Lymphoma. A prognostic factor index was proposed adapted to child patients.

4 TRIAL DESIGN AND DESCRIPTION

4.1 TRIAL DESIGN

The studies of the GPOH-HD study group since 1978 differ clearly in design and intention from typical randomised clinical trials (RCT). These non-commercial, investigator initiated trials are intended to optimise standard care for children and adolescents with Hodgkin's lymphoma.

For 25 years the study group has been developing a comprehensive therapy concept for the treatment of paediatric Hodgkin's lymphoma in six study generations so far (GPOH-HD 78, 82, 85, 87, 90 and 95). The respective study concept is de facto an area-wide guideline for the treatment of this disease in Germany and all associated countries.

The EuroNet-PHL-C1 study is an inter-group, international multi-centre, treatment optimisation trial, involving the following aspects:

- Quality control treatment titration study in a stable patient population addressing consistency of absolute 5-year EFS rate estimates with a target rate of 90% in specified subgroups.
- Embedded randomised controlled chemotherapy comparison in TG-2 and TG-3 using a two-armed, parallel-group open-label approach.
- Quality control observation study for standardised risk adapted relapse therapy.

4.2 REQUIREMENTS FOR PARTICIPATING INVESTIGATORS AND TRIAL SITES

The principal investigators have to be specialised in paediatric haematology/oncology.

Requirements for trial sites:

- Access to intensive care unit,
- Access to diagnostic facilities: CT, MRI, FDG-PET
- Access to radiotherapy facilities

In addition in Germany the GPOH requires:

- 24h on-call service in paediatric haematology/oncology
- At least two investigators specialised in paediatric haematology/oncology per trial site

The common qualification criteria required by ICH-GCP and the pertinent national laws will be assessed by the involved ethics committees before start of the trial. There are no further specific requirements for participating investigators or trial sites.

The study chairpersons involved are responsible for the selection of qualified investigators and trial sites in their country.

The co-ordinating investigator on behalf of the sponsor is responsible for setting up written agreements with every trial site involved in Germany. The authorised institutions (cf. chapter 15) in all other participating countries are responsible for setting up written agreements with every trial site involved in the respective country.

4.3 TRIAL SITES AND NUMBER OF TRIAL SUBJECTS

At least 200 trial sites, associated with the participating study groups, are expected to take part in the EuroNet-PHL-C1 study.
At least 1200 patients with real time central review will be included in the study on primary therapy. In addition about 500 patients of the SFCE study group and possibly further patients from other study groups using local staging and response assessment are expected. The expected number of annually recruited patients with real time central reviewing of staging and response assessment is 250-300.
According to past experience these patients are distributed among the therapy groups 1 or 2 and 3 in a ratio of 36:28:36.
For the relapse study 150-250 patients are expected.

4.4 EXPECTED DURATION OF TRIAL

The EuroNet-PHL-C1 study is planned to run for 5 years.
For all patients an individual 5-year follow-up plan will be followed. In addition, long-term follow up and monitoring is recommended to evaluate the risk of infertility in males and premature menopause in females. Risk of infertility in males and premature menopause in females as well as the risk of secondary malignancy will also be monitored within a late effects registry.
The EuroNet-PHL-C1 trial formally ends with the follow-up after 5 years of the last patient included. This will probably be achieved in spring 2018.
The planned treatment duration varies according to therapy group and the patient’s response. Table 9 gives an overview on the planned treatment duration.

Table 9 Planned therapy duration

Patient group	Planned therapy period (according to response)
primary therapy TG-1	6 – 9 weeks
primary therapy TG-2	19 – 22 weeks
primary therapy TG-3	27 – 30 weeks
2nd line treatment after late relapse from TG-1	17 weeks
2nd line treatment after early relapse (TG-1 – TG-3) or late relapse from TG-2 /TG-3	17 – 20 weeks
2nd line treatment after progression	20 weeks

4.5 PREMATURE TERMINATION

4.5.1 Premature closure of a trial site

Premature closure of a trial site is to be considered if:

it does not meet the technical requirements of the protocol,

the conduct of the study is not compliant with the protocol, or

data quality does not meet required standards

data return is insufficient for trial purposes.

The premature closure of a site will be decided by the study chairperson of the respective country after consultation with the responsible biometrician and the inter-group chairmen.

Investigators and trial sites deciding not to take part in the trial any longer have to inform the coordinating investigator immediately. The decision should be well-founded.

Details on further treatment and follow-up of patients already on study have to be discussed with the co-ordinating investigator.

4.5.2 Premature termination of the trial or of trial arms

In case of the following situations, a premature termination of the trial or of trial arms has to be considered:

Serious adverse drug reactions leading to substantial changes in risk-benefit considerations

Unacceptable toxicity (e.g. cumulative occurrence of deaths conditional on therapy)

Insufficient efficacy

Superiority of one therapy arm

New insights from other trials

Insufficient recruitment rate

Unsustainable trial organization

The Data Monitoring Committee will monitor the study conduct and the safety aspects of the trial on a regular basis, and will give recommendations to the Inter-Group Trial Steering Committee whether to stop the trial or to change the trial protocol. The Inter-Group Trial Steering Committee will then decide on the actions to be taken.

5 SELECTION OF TRIAL SUBJECTS FOR PRIMARY THERAPY

5.1 INCLUSION CRITERIA

Trial sites register patients at their corresponding study office if the following requirements are met:

- diagnosis of classic Hodgkin's lymphoma
- patient aged under 18 years at time of diagnosis
- written informed consent of the patient and/or the patient's parents or guardian according to national laws

Country-specifically very young patients may be excluded to comply with national laws or formal insurance requirements.

5.2 EXCLUSION CRITERIA

Patients with one of the following circumstances are excluded:

- pre-treatment of Hodgkin's lymphoma differing from study protocol (except recommended pre-phase therapy of a large mediastinal tumour)
- known hypersensitivity or contraindication to study drugs
- diagnosis of lymphocyte predominant Hodgkin's lymphoma
- prior chemotherapy or radiotherapy
- other (simultaneous) malignancies
- pregnancy and / or lactation
- females who are sexually active refusing to use effective contraception (oral contraception, intrauterine devices, barrier method of contraception in conjunction with spermicidal jelly or surgical sterile)
- Current or recent (within 30 days prior to start of trial treatment) treatment with another investigational drug or participation in another investigational trial
- severe concomitant diseases (e.g. immune deficiency syndrome)
- known HIV positivity

5.3 SUBSEQUENT EXCLUSION OF PATIENTS

Patients are excluded from the study after registration if:

- documents or material ascertained before study inclusion show that an exclusion criterion was fulfilled or an inclusion criterion was not met
- the patient and/or the patient's parents or guardian withdraw(s) his/her/their consent to further study participation

If the diagnosis of Hodgkin's lymphoma is not confirmed by the reference pathologist the patient is still documented in the scope of the study. Patient's physician decides then together with the patient/parents or guardian on the further therapy.

The study chairpersons decide on the exclusion together with the biometrician of the study. A trial site may request the subsequent exclusion of a patient by sending a written detailed account of the reasons to the study office.

Subsequent exclusion of a patient differs from an individual therapy withdrawal. In the latter case the treatment of the patient according to protocol is terminated, but follow-up and documentation (data collection in the CRF) is continued according to protocol and the patient appears in all relevant analyses.

5.4 GENDER DISTRIBUTION

All eligible patients (and his/her parents / guardian) will be asked for informed consent for participation in the trial independent of gender. Therefore, the gender distribution will reflect the underlying gender distribution in paediatric Hodgkin's lymphoma.

6 DIAGNOSTICS

If Hodgkin's lymphoma is suspected, an open biopsy should be obtained as soon as possible (exception large mediastinal tumour see chapter 8). After confirmation of the diagnosis by the local pathologist **FDG-PET** scanning should be performed **before starting treatment** (exception oncologic emergency).

6.1 CONFIRMATION OF DIAGNOSIS

The histopathological diagnosis is based on a biopsy of a lymph node or as the case may be of a biopsy of another primarily involved organ. Biopsies using a fine needle are not appropriate.

Reference pathology is mandatory and is organized country specific as detailed in the **pathology manual**.

If a Hodgkin's lymphoma is diagnosed and the patient and his / her parents / guardian declares his / her consent the investigator registers the patient in the corresponding study office of the EuroNet-PHL-C1 study.

In case of CD20+ positivity do not start treatment until the differential diagnosis of classic Hodgkin's lymphoma versus lymphocyte predominant HL is confirmed by reference pathology. Patients with LP-HL qualify for the planned EuroNet-PHL-LP1 study.

6.2 CLINICAL AND LABORATORY DIAGNOSTICS BEFORE / DURING THERAPY

6.2.1 Diagnostics prior to chemotherapy

Exact clinical history including:

- previous and concomitant diseases (e.g. **paraneoplastic phenomena** such as nephrotic syndromes and other autoimmune diseases),
- systemic symptoms and
- prior treatment.

Clinical examination

- detailed documentation of all **palpable lymph nodes and their localisation**.
- examination and inspection of Waldeyer's ring preferably by ENT-physician.

- Palpation of spleen and liver

Laboratory examinations:

- Complete blood count, erythrocyte sedimentation rate, ALAT (GPT), ASAT (GOT), GGT, LDH, AP, creatinine and albumin in the serum, fibrinogen, Immunoglobulin A, G, M
- Protein electrophoresis: gamma-globuline and alpha-2-globuline
- Baseline virology recommended to include serologic examinations for antibodies against VZV, EBV, CMV, HSV, HIV, toxoplasmosis, hepatitis A, B, C (HCV-PCR).

Functional examinations

- ECG
- Echocardiography
- EEG (optional)

Pubertal development and fertility

Boys

- Tanner stage of secondary sexual characteristics (Tanner 1962).
- Assessment of testicular volume using the Prader Orchidometer
- Measurement of serum FSH, LH, testosterone and inhibin B (if available)
- Semen analysis if sexually mature (in particular in case of cryopreservation)

Cryopreservation of semen should be offered to young male patients if sexually mature

Girls

- Tanner staging of secondary sexual characteristics
- Date of menarche and regularity of menses
- Measurement of serum FSH, LH, oestradiol and inhibin B (if available) unless on contraceptive pill

6.2.2 Diagnostics assessment before each course of chemotherapy

Before start of each chemotherapy cycle:

- Presence of infections
- detailed clinical examination

- Lansky/Karnofsky's score depending on age
- blood counts including differential blood count
- ALAT, ASAT, GGT, bilirubin, creatinine

Further diagnostic measures (such as ECG, lung function etc.) are carried out according to the individual circumstances of the patient.

6.3 IMAGING DIAGNOSTICS

6.3.1 Initial staging

In GPOH-associated centres every patient requires a FDG-PET examination before starting any therapy (exception vital emergencies)!
In centres without central review a FDG-PET scan is strongly recommended before treatment commences.

Cross sectional imaging is mandatory. It should preferably be performed according to options A and B, see below.
Low dose chest CT should be performed with weight-dependent dosage if possible.

A) CT chest with mediastinum, MRI neck / abdomen / pelvis

CT examination:

CT examination of thorax is mandatory, since lung foci can be best diagnosed with CT. (Recommendations for CT examination performance see B).

MRI examinations should include the following sequences:

- **neck**: transversal and coronal T2 fat saturated T2 (T2-TIRM, T2-STIR)
- **thorax**: If performed in addition to chest CT an MRI examination of lung and mediastinum should provide ECG triggered transversal sequences to avoid pulsation artefacts.
- **abdomen**: transversal T2 fat saturated and T1-FLASH 2d dynamic in arterial, portal venous and venous phase
- **pelvis**: T1-SE transversal, T2- fat saturated transversal and coronal, T1-SE-transversal after contrast agent with fat saturation

B) CT neck / thorax / abdomen / pelvis

Recommendation for examination performance:

examination region: epipharynx to lower edge of symphysis

layer thickness:	reconstructed 5 mm layers
oral contrasting:	yes
i.v. contrasting:	depending on KG 1.5 – 2.0 ml/kg KG (recommended up to 10 kg KG 2 ml/kg, up to 40 kg KG 1.5 ml/kg, from 40 kg 60 ml; choose delay according to device so that a parenchymal phase of liver and spleen is achieved)
reconstruction:	lung: sharp kernel and pulmonary window, mediastinum with involvement of axillae, supra- and infraclavicular region: soft kernel and mediastinal window, abdomen and pelvis involving inguinal region: abdominal window (e.g. W 400/C 60)

C) CT in combination with PET:

If **only** a PET-CT is performed, the CT-images have to be “state of the art” quality. This includes: oral and i.v.contrasting (as described in B), the choice of an adequate mAs dose (not low dose) and a slice thickness not higher than 5 mm. The imaging quality of the PET-CT-images has to be comparable to normal diagnostic CT.

D) Whole body-MRI

Whole body-MRI (WB-MRI) is a promising technique for initial staging and restaging. The real value of WB-MRI is currently under investigation. **Thus, it should not replace staging options A to C.**

Additional imaging procedures

Sonography of spleen and liver are mandatory.

Chest X-ray should be performed with measurement of mediastinal ratio of the maximum diameter mediastinal mass to the maximum intrathoracic diameter (at the level of T5/T6).

Bone scan: This examination is only performed in patients with suspected skeletal involvement based on FDG-PET or clinical symptoms

Additional points to consider

For central review digital transmission of cross sectional imaging and bone scans is appreciated (CD with automatically opening (DICOM) viewer if necessary as teleradiology link). If this is not possible central review is performed on the basis of original or copied images. **The original report of the local radiologist** should be included for orientation.

In non-GPOH-associated trial sites, the tumour volume is determined by central review or at the local hospital.

After lymph node biopsy the respective region has to be re-examined with imaging methods before starting chemotherapy as a prerequisite for accurate response assessment.
PET positive regions not investigated by cross-sectional imaging (e.g. extremities) must be examined using conventional imaging (CT or MRI) **before starting therapy**.

6.3.2 FDG-PET examination

For detailed instructions please confer to the FDG-PET procedural manual.

6.4 ASSESSMENT OF INVOLVED REGIONS

The EuroNet-PHL-C1 study is an inter-group trial. The GPOH-HD group pursues a quality assurance strategy with real time central review of staging and response assessment. Some participating study groups take part in this process; other involved study groups do not.

Depending on country patients qualify for either **c**entral **s**taging and **r**esponse **a**ssessment (CSRA) or only **l**ocal **s**taging and **r**esponse **a**ssessment (LSRA).

For all patients the **clinical stage** is determined by the local investigator.

In CSRA patients the **reference stage** is defined by the GPOH-HD central review.

Decision on treatment group is determined by the clinical stage (LSRA) or the reference stage (CSRA).

The region of the diagnostic biopsy is involved by histological evidence. If staging FDG-PET was performed **after** the biopsy and the biopsy region is not PET-positive then the region is considered PET-unclear and does not constitute a discordantly negative PET result

6.4.1 Assessment of nodal involvement

6.4.1.1 Assessment of lymph node involvement

1. If the largest diameter of a lymph node or a lymph node conglomerate is smaller than 1 cm the region is considered not involved – independent of the PET result. Unidentified micro-findings do not impair therapy results according to previous experience. If however small FDG-PET positive lymph nodes accumulate this region is considered involved.

2. If the largest diameter of a lymph node or a lymph node conglomerate exceeds 2.0 cm the region is considered involved. If such an involved region is FDG-PET-negative this discordancy must be documented, since this is relevant for the decision on radiotherapy because response in such a region cannot be reliably assessed by FDG-PET (cp. chapter 8).

3. If a lymph node or a lymph node conglomerate has a diameter of 1.0 – 2.0 cm the region is considered
   - involved if it is FDG-PET positive.
   - not involved if it is FDG-PET negative
   - doubtfully involved if the FDG-PET has not been performed or is not evaluable (e.g. in muscle artefacts). In these cases the decision on involvement of this region is made including further criteria, such as clinical data, ultrasound findings, proximity to larger involved region.

6.4.1.2 Assessment of Waldeyer's ring

1. Involvement is defined by clinical assessment preferably by ENT physician and is not measurable: involvement yes/ no; localisation left/right;
2. FDG-PET assessment is irrelevant.
3. Biopsy is not required and not appreciated since too invasive

6.4.2 Assessment of extra-nodal involvement

6.4.2.1 Pleura and pericardium

Involvement of the pleura is assumed if

- the lymphoma is contiguous with the pleura without fat lamella or
- the lymphoma invades the chest wall or
- a pleural effusion occurs which can not be explained by a venous congestion.

Pericardial involvement is assumed if

- the lymphoma has a broad area of close contact towards the heart surface beyond the valve level (ventriculus area) or
- a pericardial effusion occurs.

Pleura and/or pericardial involvement are generally considered E-lesions.

6.4.2.2 Extra-nodal involvement

Extra-lymphatic structures or organs that are infiltrated per continuum out of a lymphatic mass are termed E-lesion (examples: lung, intestine, bones) and do not automatically qualify for stage IV. **Exceptions: Liver or bone marrow involvement always implies stage IV.**

6.4.3 Organ involvement

6.4.3.1 Lung involvement

A disseminated lung involvement (implying stage IV) is assumed if

- there are more than three foci or
- an intrapulmonary focus has a diameter of more than 10 mm.

If a smaller than 10 mm involvement is seen (also if FDG-PET positive) or there is only a PET positive finding, stage IV is not assumed since these patients have had a very good prognosis in the past without upstaging.

E-lesion of the lung is restricted to one pulmonary lobe or perihilar extension with homolateral hilar lymphadenopathy.

6.4.3.2 Liver and spleen involvement

- Liver involvement implies always stage IV.
- Exclusive splenic involvement without other lymphatic disease is classified as stage I.
- Mere enlargement of liver / spleen only is not considered as involvement.

- Focal changes in the liver / spleen structure that are **tumour suspicious** in ultrasonography are considered involved – independent of the FDG-PET result.
- In case of doubtful involvement of liver or spleen (e.g. structures atypical of tumour in sonography or MRI) the liver / spleen is considered
  - o involved if FDG-PET is positive.
  - o not involved if FDG-PET is negative.
  - o doubtfully involved if the FDG-PET was not performed or if it is not evaluable. In these rare cases the decision on involvement is made taking further criteria into the account.

6.4.3.3 Bone / bone marrow involvement

Bone involvement is assumed if

- A bone biopsy is positive **or**
- CT bony window is positive with or without further confirmation by other imaging methods in the same region **or**
- A positive bone scan is confirmed by either FDG-PET or MRI.
- MRI positive in bone and adjacent soft tissue (T2 fat saturated sequences).

In the event of an isolated lesion or relevant history (e.g. trauma) only a positive biopsy indicates bony involvement.

Bone marrow involvement is assumed if

- bone marrow biopsy is positive **or**
- FDG-PET and MRI are both positive ***in the same region***

A bone marrow biopsy is mandatory unless stage I or IIA. Bone marrow involvement implies stage IV.

6.5 INDICATIONS FOR INVASIVE DIAGNOSTICS

6.5.1 Bone marrow biopsy

All patients with a stage >IIA get a bone marrow biopsy in one or two regions.

6.5.2 Selective laparoscopy

Selective laparoscopy is indicated only in rare cases where involvement can not be clarified with **any available imaging method** including FDG-PET.

6.5.3 Ovariopexy

Whenever an iliac lymph node region is to be irradiated in girls lateral movement of the adjacent ovary should be considered.
Ovariopexy is particularly recommended if both ovaries are expected to receive a dose of more than 5 Gy which may lead to significant long-term ovarian impairment. Using opposed fields with 20 Gy, this can be usually be avoided, if the ovary is more than 2 cm from the adjacent field (shield) border.

When performing an ovariopexy sutures should be marked with clips! After consultation with the radiotherapist surgery should be carried out immediately before infra-diaphragmatic irradiation.

6.6 OPTIONAL PROCEDURES

Male patients who are post-pubertal with testicular volumes, as measured using a Prader Orchidometer of >10mls should be offered semen cryopreservation before treatment begins.Techniques to preserve fertility in pre-pubertal males remain entirely experimental. For females the only established method of fertility preservation is embryo freezing which requires a consenting male partner. Harvesting of ovarian cortical tissue or eggs for cryopreservation remains experimental and should only be undertaken in institutions with appropriate ethical consents in place.

6.7 RESTAGING

6.7.1 General information

The following cross-sectional imaging procedure is recommended for restaging:

neck:	**MRI** / (CT)
thorax	low dose **CT** (obligatory in lung involvement) / **MRI**
abdomen:	**MRI + sonography** / (CT)
pelvis (inguinal)	**MRI** / (CT)

6.7.2 First restaging after 2 cycles of chemotherapy

All patients get an FDG-PET examination on day 14 (day 17 as latest) after the last application of chemotherapy. All initially involved regions are checked. CT/MRI/ultrasound examinations have to be performed between day 10 and 14 after the last application of chemotherapy.

Response in initial bone or bone marrow involvement is only assessed by FDG-PET, assuming that it would be still detectable by conventional imaging.

Patients with suspected progression get a complete staging of all lymph node regions.

6.7.3 Second restaging after full chemotherapy in therapy groups 2 + 3

After 4 or 6 cycles of chemotherapy all initially involved regions of the patients in TG-2 +3 are re-examined. CT / MRI / ultrasound examinations have to be performed between day 10 and 14 after the last application of chemotherapy in the fourth or sixth cycle respectively. **FDG-PET is not performed**. Patients with suspected progression should have a complete staging of all lymph node regions.

6.7.4 Follow-up

Follow-up starts six weeks after completion of therapy according to the recommendations (Table 10; cf section 10.2).

Table 10 Recommendations for follow-up examinations

	**Time**					
**Procedures**	**In 1st year**<br>**First follow-up 6 weeks after end of treatment**	**In 2nd year**	**In 3rd year**	**In 4th year**	**In 5th year**	**from 6th year on**
Clinical history and examination	4 - 8x	4 - 8x	4x	2x	2x	Individually
Blood counts, ESR	4x	4x	2x	2x	2x	
After radiation of mediastinum and/or lung:<br>Functional lung evaluation	1x	Individually				
Consultation of radiotherapist	1x	1x	1x	1x	1x	
Assessment of quality of life (Germany only)	1x	1x	1x	1x	1x	
Sonography abdomen	4x	4x	2x	2x	2x	
MRI in involved region	2-4x	1-2x	1-2x	1-2x	1-2x	
CT thorax in case of lung involvement	2x	1x	Individually			
After neck radiation:<br>thyroidsonography, fT4, TSH, TG	1x	1x	1x	1x	1x	
ECG / echo-CG		1x			1x	
FDG-PET	Only if relapse confirmed					
After radiation of supra-/infraclavicular region, axillae, mediastinum or lungs:<br>Mammary carcinoma screening (Sono, MRI)	To be considered in women above age 25 annually					
Pubertal development and fertility assessment (compare 6.7.5 and appendix)	Annually:<br>▪ Tanner stage of secondary sexual characteristics<br>• Boys: Assessment of testicular volume using Prader Orchidometer<br>• Girls: Regularity of menses,<br>if feasible: ovarian volume (trans-vaginal sonography)<br>• Measurement of serum FSH, LH, testosterone (boys)/ oestradiol (girls) and inhibin B (if available) from age 11-12yrs. (Except girls taking the pill).<br><br>Semen analysis once sexually mature, off treatment for >12 months and patient willing to provide a specimen.					

6.7.5 Assessment of puberty and fertility

The assessment of gonadal function involves regular clinical assessment of pubertal progression, biochemical assessment of plasma gonadotrophins and sex steroids, semen analysis in males and menstrual history in females.

Testicular enlargement is the first sign of puberty in normal boys, followed by penis enlargement and the development of pubic hair (Tanner, 1962). In females puberty commences with development of the breast bud, with menarche occurring around two years later (Tanner, 1962).

Biochemical analysis of patients who have impaired fertility following treatment will usually demonstrate a raised early follicular phase Follicle Stimulating Hormone (FSH), and reduced oestradiol in females. In males FSH is increased, but Leydig cell function may be normal, characterised by normal plasma testosterone and normal or only marginally elevated Luteinizing Hormone (LH).

Assessment of fertility, both clinically and biochemically, is not possible before and during puberty. At present there is no reliable method of assessing gonadal function in this age group. However, measurement of Inhibin B, a glycoprotein secreted predominantly from Sertoli cells in males and developing antral follicles in females (Anderson and Sharpe, 2000; Roberts *et al*, 1993) that plays an important role in spermatogenesis and folliculogenesis in adult males and females respectively, may represent a potential marker of gonadotoxicity. There is evidence to suggest that gonadotoxic chemotherapy is associated with a reduction in inhibin B levels in adult males (Wallace *et al*, 1997), presumably indicating reduced sperm production (Anderson and Sharpe, 2000). However, this relationship has not been clearly demonstrated in childhood (Crofton *et al*, 2003), and it remains to be seen if inhibin B will become a useful tool in fertility assessment of these patients in the future.

7 STAGE CLASSIFICATION AND DEFINITION OF THERAPY OUTCOME

7.1 STAGE CLASSIFICATION

Stage classification is performed according to Cotswolds revision of the classical Ann Arbor staging system.

The diagnostic criteria for involvement formally integrating FDG-PET results are explained in chapter 6.4.

Table 11 details the definition of lymph node regions and extra-nodal sites on which staging is based.

Table 11 Independent lymph node regions

Independent lymph node regions are:

- **Waldeyer's ring (left and right)**
- **cervical (left and right) with sub-regions relevant for irradiation:**
    - o upper neck: up to upper edge of larynx
    - o lower neck: up to supraclavicular fossa
- **supraclavicular (left and right)**
- **infraclavicular (left and right)**: subpectoral on the thoracic wall
- **axillar (left and right)**
- **lung hilus (left and right)**: bronchopulmonary LN
- **mediastinum with sub-regions relevant for irradiation**:
    - o upper mediastinum: down to bifurcation
    - o middle mediastinum: hilus down to subcarinal region
    - o lower mediastinum: down to diaphragm
- **supradiaphragmatic:** diaphragmatic recessus
- **spleen**
- **splenic hilus**
- **liver hilus**
- **mesenteric**: mesentery, mesocolon
- **paraortic:** coeliac, paraaortocaval, pararenal, paralienal, parapancreatic
- **iliac (left and right)**: parailiac
- **inguinal (left and right)**: inguinal, femoral

Table 12 Stage classification Hodgkin's lymphoma

**Stages of Hodgkin's lymphoma according to the Cotswolds revision of the Ann Arbor staging system**
I Involvement of a single independent lymph node region or lymph node structure
II Involvement of 2 or more lymph node regions on the same side of the diaphragm
III Involvement of lymph node regions or lymph node structures on both sides of the diaphragm
IV Involvement of extra-nodal sites beyond "E"-sites

Table 13 Annotations to stage definitions

A. No B symptoms

B. At least one of the following systemic symptoms
   a. Inexplicable weight loss of more than 10% within the last 6 months
   b. Unexplained persisting or recurrent temperature above 38 °C
   c. Drenching night sweats

E. Involvement of a single extra-nodal site contiguous or proximal to known nodal site. (For the distinction between stage IV and the E-stages see chapter 6.4.2.)

7.2 DEFINITION OF TREATMENT RESPONSE

7.2.1 Local response definitions for nodal involvement with measurable tumour volume

At staging all measurable nodal sites (i.e. all nodal except for the spleen and Waldeyer's ring) are grouped in **separately measurable reference volumes**. Reference volumes can include multiple sites if these are contiguous.

The composition of these reference volumes is defined and documented. Initial volumes of reference volumes are measured.

Volumes are approximated as ellipsoids. If a, b, c denote the principal axes of the ellipsoid the volume is calculated as V= (a x b x c)/2.

In this protocol overall response to treatment is determined according to a systematc assessment of tumour response in all involved sites.
In those sites where the tumour is measurable from CT/MRI scanning the change in tumour volume is compared the original pre-treatment reference volume and then assigned a treatment response for that local site.

7.2.1.1 Local Complete remission (localCR)

A reference volume is in "local complete remission"(in short: localCR) if:

- the residual tumour volume is less or equal 5% of the reference volume (CT/MRI) **and**
- the residual tumour volume is less or equal 2 ml.

7.2.1.2 Local complete remission unconfirmed (localCRu)

A reference volume is in "local complete remission unconfirmed "(in short: localCRu) if:

- No localCR **and**
  - o the residual tumour volume is less or equal 25% of the reference volume (CT/MRI) **or (!)**
  - o the residual tumour volume is less or equal to 2 ml

7.2.1.3 Local partial remission (localPR)

A reference volume is in "local partial remission"(in short: localPR) if:

- No localCR or localCRu **and**
- the residual tumour volume is less or equal 50% of the reference volume (CT/MRI) **or** the residual tumour volume is less or equal 5 ml (to saveguard against artefacts due to measurement errors).

7.2.1.4 Local no change (localNC)

A reference volume is in "local no change" (in short: localNC) if

- no localCR or localCRu or localPR **and**
- no local Progression

7.2.1.5 Local Progression (localPRO)

A reference volume is in "local progression" (in short: localPRO) if

- The residual tumour volume is larger than 125% of the reference volume or significantly increases compared to the best previous response – be aware of possible measurement error in small tumour volume.

7.2.2 Local response definitions for extra-nodal involvement or for nodal involvement with non-measurable tumour volume

For all extra-nodal sites or for nodal involvement with non-measurable tumour volume three response categories are distinguished by radiological or clinical criteria:

- **Locally undetectable**
- **Locally detectable**
- **Locally progressive**

Only “Locally undetectable” is consistent with overall CR.

Note: In case of multi-focal bone or bone marrow involvement multiple sites are assessed separately. Initial bone or bone marrow involvement is only assessed by FDG-PET assuming it is still detectable by conventional imaging.

7.2.3 Overall (patient level) response definitions

Overall (patient level) response categories are obtained from the worst local response in reference volumes and the worst local response in non measurable nodal or extra-nodal disease as illustrated in the following figure:

Fig. 3. Definition of overall response

		**worst local response in nodal reference volumes**					
		no involvement	local CR	local CRu	local PR	local NC	local PRO
worst local response in extranodal or non-measurable nodal regions	no involvement		**overall CR**	**overall CRu**	**overall PR**	**overall NC**	**overall PRO**
	undetectable	**overall CR**	**overall CR**	**overall CRu**	**overall PR**	**overall NC**	**overall PRO**
	still detectable	**overall CRu**	**overall CRu**	**overall CRu**	**overall PR**	**overall NC**	**overall PRO**
	local PRO	**overall PRO**	**overall PRO**	**overall PRO**	**overall PRO**	**overall PRO**	**overall PRO**

legend: overall CR, overall CRu, overall PR, overall NC, overall PRO

7.2.3.1 Complete remission (CR)

"Complete remission"(in short: CR) is achieved if in restaging

- all disease symptoms have disappeared **and**
- no new lymphatic or extra-lymphatic lesions have occurred **and**
- ***all*** initially involved extra-nodal sites or involved regions with non-measurable tumour volume are locally undetectable **and**
- ***all*** reference volumes are in localCR

7.2.3.2 Complete remission unconfirmed (CRu)

"CR unconfirmed"(in short: CRu) is achieved if in restaging

- no CR **and**
- all disease symptoms have disappeared **and**
- no new lymphatic or extra-lymphatic lesions have occurred **and**
- ***all*** initially involved extra-nodal sites or involved regions with non-measurable tumour volume are not locally progressive **and**
- ***all*** reference volumes are at least in localCRu

7.2.3.3 Partial remission (PR)

"Partial remission" (in short: PR) is achieved if in restaging

- no CR or CRu **and**
- all disease symptoms have disappeared **and**
- no new lymphatic or extra-lymphatic lesions have occurred **and**
- ***all*** initially involved extra-nodal sites or involved regions with non-measurable tumour volume are not locally progressive **and**
- ***all*** reference volumes are at least in localPR

7.2.3.4 No change (NC)

"No change" (in short: NC) is achieved if in restaging

- no CR or CRu or PR **and**
- no PRO

7.2.3.5 Progression (PRO) / Relapse (R)

Progression / Relapse of the disease occurs if

- recurrence or occurrence of new disease symptoms which can not be explained otherwise **or**
- occurrence of new lymphatic or extra-lymphatic lesions **or**
- ***at least one*** initially involved extra-nodal site or involved region with non-measurable tumour volume is locally progressive **or**
- at least one reference volume is in localPRO

Biopsy of an enlarging region or new lesion is mandatory.

A progression / relapse of the disease is called

- **progression** if it occurs until three months after the end of therapy (last day of chemotherapy application (including Prednisone/prednisolone) or last day of radiotherapy respectively).
- **early relapse** if it occurs between three and twelve months after the end of therapy.
- **late relapse** if it occurs later than twelve months after the end of therapy.

7.3 EARLY FDG-PET RESPONSE ASSESSMENT

After two cycles of chemotherapy early response reassessment including FDG-PET is performed. **FDG-PET examinations are assessed only for initially involved regions** (except in case of suspected progression).

7.3.1 Definition local FDG-PET response

For each initially involved reference volume or non-measurable nodal or extra-nodal site a local PET response is defined based on the initial PET and the response assessment PET results as illustrated in:

Fig. 4. Definition local FDG-PET response

		initial PET – involved regions <u>only</u>		
		+	-	? / nd
early response assessment PET	+	+	+	+
	-	-	?	-
	? / nd	?	?	?

+ = positive FDG-PET

- = negative FDG-PET

? / nd = questionable FDG-PET or FDG-PET not done

Definition local FDG-PET response

- **Local PET response positive** if the response assessment PET is positive anywhere in the reference volume or involvement site.
- **Local PET response unclear,** if the response assessment PET is unclear **or** the response assessment PET is negative, but the initial PET was discordantly negative.
- **Local PET response negative** if the response assessment PET is negative **and** the initial PET was positive or unclear (i.e. not discordantly negative).

NOTE: A PET examination is locally **unclear** if

- Not done
- Not evaluable due to technical problems

in rare cases of questionable PET results.

7.3.2 Definition worst local FDG-PET response

In case of **Local PET response unclear** a further distinction is made by local CT/MRI response**:**

- **Locally PET unclear, but localCR or locally undetectable (by CT/MRI)**
- **Locally PET unclear, but not localCR or locally detectable (by CT/MRI)**

The **worst local PET response** is defined based on the following order relation:

“Local PET response positive” worse than

“Locally PET unclear, but not localCR or locally detectable” worse than

“Locally PET unclear, but localCR or locally undetectable” worse than

“Local PET response negative”

7.4 RESPONSE GROUP DEFINITION

If **no tumour progression** is found, response groups are obtained from the overall response and the worst local PET response in reference volumes and in non measurable nodal or extra-nodal disease as illustrated in Fig. 5 and Table 14:

Fig. 5. Response group definition

		**worst local PET response**			
		PET negative	PET unclear, but local CR / undetectable	PET unclear, detectable and not local CR	PET positive
**overall response**	**CR**	☐ **AR1**	☐ **AR1**		☐ **AR1**
	**CRu**	☐ **AR2**	☐ **AR2**	☐ **IRu**	☐ **IR**
	**PR**	☐ **AR2**	☐ **AR2**	☐ **IRu**	☐ **IR**
	**NC**	☐ **IRu**	☐ **IRu**	☐ **IRu**	☐ **IR**

AR1 = adequate response group 1 ⇨ **no radiotherapy**

AR2 = adequate response group 2 ⇨ **no radiotherapy**

IRu = inadequate response qroup unconfirmed ⇨ **radiotherapy**

IR = inadequate response group ⇨ **radiotherapy**

Table 14 Response groups in non progressing patients

**Adequate response 1**<br>**(AR1)**	Patients in overall complete remission irrespective of PET results.
**Adequate response 2**<br>**(AR2)**	Patients in overall CRu or overall PR for whom<br>**all** initially involved regions are<br>▪ **PET-negative** or<br>▪ **PET-unclear** and<br>o in local CR or<br>o undetectable
**Inadequate response**<br>**(IR)**	Patients<br>▪ not in overall complete remission **and**<br>▪ at least one initially involved region is **PET-positive**.
**Inadequate response unconfirmed**<br>**(IRu)**	▪ Patients in overall CRu or overall PR **and**<br>▪ **no** initially involved region is **PET-positive and**<br>▪ at least one initially involved region is **PET-unclear and**<br>o not in local CR or<br>o still detectable<br>**or**<br>▪ Patients in overall No Change **and**<br>▪ **no** initially involved region is **PET-positive**.

The distinction between AR1 and AR2 is made because in TG-1 AR1 the GPOH 95 study has already shown that omitting radiotherapy is safe, while in TG-1 AR2 this is an open study question.

8 THERAPY PLAN FOR PRIMARY THERAPY

In case of <u>CD20+ positivity do not start treatment</u> until the differential diagnosis of classical Hodgkin's lymphoma versus lymphocyte predominant HL is confirmed by reference pathology. Patients with LP-HL qualify for the planned EuroNet-PHL-LP1 study.

8.1 TREATMENT GROUPS

Patients are divided into treatment groups (TG) according to the reference stage (defined by the central review in the GPOH-HD study office) or the local stage (non-GPOH associated hospitals).

Treatment groups are as follows:

TG-1: patients of stages I A/B and II A without bulk>=200 ml and without ESR>=30mm/hr

TG-2: patients of stages I_EA/B, II_EA, II B or III A

and patients of stages I A/B and II A with bulk>=200 ml and/or ESR>=30mm/hr

TG-3: patients of stages II_EB, III_EA/B, III B or IV A/B

8.2 TREATMENT GROUP 1 (TG-1)

All patients receive two cycles of OEPA. Patients in TG-1 with adequate response (response groups AR1 and AR2) receive no further therapy. Patients with inadequate response (IR and IRU) will receive involved field radiotherapy (see chapter 8.8 and **radiotherapy manual**). Radiation therapy should start by day 35 after the last dose of chemotherapy in the second OEPA-cycle (i.e. day 77 after start of treatment) **at latest**.

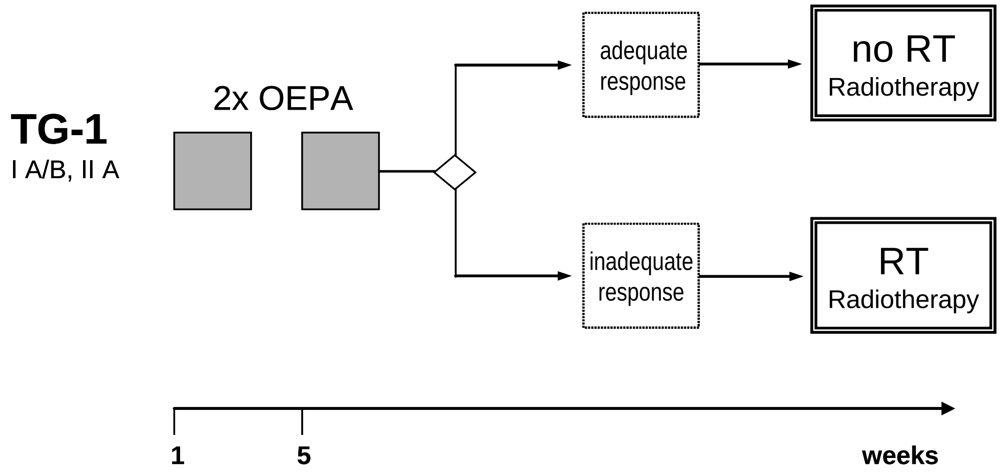

Fig. 6. Treatment overview for TG-1

8.3 TREATMENT GROUP 2 AND 3 (TG-2, TG-3)

After initial staging and assignment to treatment groups 2 or 3, patients are treated with COPDAC (see section 10.2.1) from 13.2.2012 9:00 a.m.

This Patient data is required to fully answer the other important questions in the protocol relating to the STAR approach to treatment and whether it is safe to omit radiotherapy. It will also help build up the safety data. Following completion of 2 cycles of OEPA and after early response assessment including FDG-PET, patients in TG-2 receive two cycles, patients in TG-3 four cycles of COPDAC.

Patients in TG-2 / TG-3 with adequate response in early response assessment (response groups AR1 and AR2) receive no radiotherapy.

Patients with inadequate response (IR and IRU) will receive involved field radiotherapy after the end of chemotherapy (see chapter 8.8 and **radiotherapy manual**). Radiotherapy begins 14 days after last dose of prednisone/prednisolone of the 4th respectively 6th chemotherapy cycle (TG-2: day 112 after start of treatment, TG-3: day 168 after start of treatment).

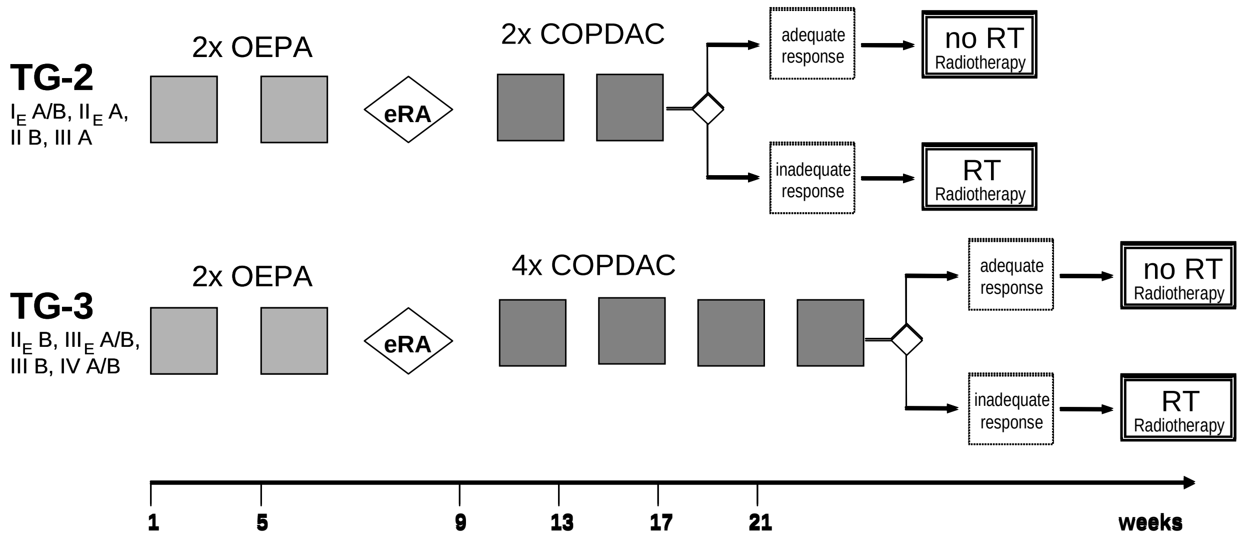

Fig. 7. Treatment overview for TG-2 and TG-3

8.4 CHEMOTHERAPY PLANS

The first cycle of OEPA starts immediately after completion of staging. In the rare case of a staging laparoscopy, the first cycle of OEPA should start about 5 days after surgery. Details of chemotherapy administration are available in the chemotherapy drug monographs in the appendix.

The subsequent chemotherapy cycle starts on d29 of each cycle when the following criteria are fulfilled:

- general condition satisfactory
- WBC over 2,000 / mm^3
- ANC over 500 / mm^3
- platelets over 80,000 / mm^3
- no contraindication to any of the prescribed drugs

In case of patients with an expected delay of more than one week, please contact your regional study chairperson.

Severe side effects are not expected with OEPA, COPP or COPDAC. Chemotherapy should only be interrupted in case of severe inter-current infections. In parallel to chemotherapy patients may receive hyperhydration with 2.5-3 l/m^2 per day of glucose-saline solution.
For oncological emergencies in patients with initially large tumour mass please see chapter 8.10.

8.4.1 OEPA

Application schedule and dosage of cytotoxic drugs during a cycle are shown in Table 15. After each cycle there is a treatment-free interval between day 16 and 28. The next cycle starts on day 29.

Table 15 OEPA scheme

**Prednisone/prednisolone**<br>60 mg/m^2/day p.o. divided into 3 doses<br>day 1 – 15	•	•	•	•	•	•	•	•	•	•	•	•	•	•	•
**Vincristine**<br>1.5 mg/m^2 i.v., max. SD 2 mg<br>day 1 + 8 + 15	•							•							•
**Doxorubicine**<br>40 mg/m^2 as 1-6 hour infusion<br>day 1 + 15	•														•
**Etoposide/Etopophos**<br>125 mg/m² as 1-2 hour infusion<br>day 1 – 5	•	•	•	•	•										
Day	1	2	3	4	5	6	7	8	9	10	11	12	13	14	15

8.4.2 COPDAC

Application scheme and dosage of cytostatic drugs during a cycle are shown in Table 16 and 0. After each cycle there is a treatment-free interval between day 16 and 28. The next cycle starts on day 29.

To minimise toxicity to the urinary tract, there is the option to give the uroprotector Mesna along with every application of Cyclophosphamide.

Dacarbazine is highly emetogenic. Therefore 5-HT3-antagonists, possibly supplemented with Dexamethasone (optional) or neuroleptics (e.g. Levomepromazin), are recommended for antiemesis.

Table 16 COPP scheme - *erased*

Table 17 COPDAC scheme

**Prednison/Prednisolone**<br>40 mg/m^2/day p.o. divided into 3 doses<br>day 1 – 15	•	•	•	•	•	•	•	•	•	•	•	•	•	•	•
**Dacarbazine**<br>250 mg/m^2 as 15 - 30-min. inf.<br>day 1 – 3	•	•	•												
**Vincristine**<br>1.5 mg/m^2 i.v. max. SD 2 mg<br>day 1 + 8	•							•							

**Cyclophosphamide**<br>500 mg/m^2, 60-min. inf.<br>day 1 + 8<br>Intravenous hydration with glucose/ saline solution at a rate of 3 l / m^2/ 24 hours commencing with the first cyclophosphamide dose and continuing for at least six hours after last cyclophosphamide dose.	•							•							
Day	1	2	3	4	5	6	7	8	9	10	11	12	13	14	15

8.5 DOSE MODIFICATIONS

Since chemotherapy is well tolerated no detailed provisions for dose modifications are given, even in patients with haematotoxicity CTC grade 4. A short therapy delay may be indicated in a few cases. For the criteria for the continuation of chemotherapy see section 8.4.

In case of drug-specific toxicity (examples: impaired cardiac function after Adriamycin, severe neuropathy during or after Vincristine) or other unexpected severe adverse events the study chairpersons should be consulted to discuss therapeutic alternatives.

8.6 CONTRACEPTION

All patients are advised that during chemo-/radiotherapy and up to one year afterwards procreation of children is not recommended, there is a risk of an adverse effect on the fetus. The treating physician should advise about methods of contraception individually. The occurrence of pregnancy in patients or their partners should be reported to the study office.

8.7 SIDE EFFECTS OF CHEMOTHERAPY

General acute side effects of chemotherapy include: nausea, vomiting, weight loss and alopecia. General late side effects of chemotherapy include: increased risk of secondary malignancies, infertility or premature menopause, and increased cardiac event risk.

For additional information on side effects please check chemotherapy drug monographs in the appendix of this protocol.

8.7.1 Etoposide

Acute side effects of Etoposide include: allergic reaction, mucositis, peripheral neuropathies CNS toxicities, mild bone marrow depression and secondary leukaemia.

8.7.2 Dacarbazine (DTIC)

Dacarbazine is highly emetogenic. Diarrhoea, influenza-like symptoms, allergic skin reactions, fever, photosensitization, local vein irritation as well as flush symptoms can occur during or after injection. Bone marrow toxicity is generally low. Rarely liver, kidney and CNS toxicities (apathy, seizures) occur. A mutagenic, carcinogenic and teratogenic effect for DTIC has been demonstrated in animal experiments. During the GPOH-HD 2002 Pilot study one patient died with rhabdomyolysis after DTIC.

8.7.3 Vincristine (VCR)

Acute side effects after application of Vincristine are: peripheral neuropathy, constipation, rarely syndrome of inappropriate ADH secretion (= SIAD). In case of severe peripheral neuropathy, especially in motor disturbances or paralysis of hands and/or legs, a replacement of Vincristine by Vinblastine in a dose of 6 mg/m^2 is recommended.

8.7.4 Cyclophosphamide

Under Cyclophosphamide the following side effects can occur: bone marrow depression, increased infection risk, haemorrhagic cystitis.

8.7.5 Adriamycin (Doxorubicine)

Even at a low cumulative dose Adriamycin can lead to a permanent damage of the heart muscle. However, the extent of the long-term cardiac risk is unknown. Therefore before starting chemotherapy the heart function has to be examined (echocardiography) and documented. In case of initial damage of the heart function a therapeutic alternative should be discussed with the study chairpersons.

8.7.6 Prednisone/Prednisolone

Changes in the bone metabolism have been detected (especially in patients with leukaemia) which can lead to osteonecrosis. In rare cases an artificial joint replacement may be required. In

addition, the Prednisone/Prednisolone therapy can lead to reversible retention of water, increase in weight, increased infection risk and psychosis/mental disorders.

8.7.7 Procarbazine *- erased*

8.8 RADIOTHERAPY

For patients in all therapy groups (TG-1, TG-2 and TG-3), the decision on radiotherapy is based on early response assessment after 2 cycles of OEPA. Patients with adequate response (response groups **AR1** and **AR2,** see section 7.3) will not receive radiotherapy.

A detailed description of the principles of radiotherapy, radiation planning and technical requirements are provided by the **radiotherapy manual**. Briefly, a short overview of important definitions is provided within this protocol:

8.8.1 Target volumes (Modified Involved Field and Boost Radiotherapy)

Two different target volumes (Planning Target Volumes (PTV)) are defined within this protocol:

- **Modified involved field (PTV_1) -**
  includes **all initially involved lymph nodes as recorded before chemotherapy** with a minimum safety margin of 1 - 2 cm taking into account the "area" of involvement.
  Exceptions are the mediastinum, the para-aortic region,and pelvic areas: chemotherapy response is taken into account (in particular for lateral borders) to minimise irradiation of sensitive normal tissue. The radiation dose is 19,8 Gy.
- **Boost radiotherapy (PTV_2) –**
  includes **all lymph nodes with poor response after two cycles of chemotherapy** with a 1 – 2 cm safety margin.
  If in a given region the residual tumour volume after two cycles of OEPA is > 100 ml or less than 75% regression of the initial tumour volume, but still > 5 ml an additional boost of 10 Gy is given to this region with a safety margin of 1 - 2 cm. The boost volume can (by definition) not exceed the size of the modified involved field.

Asymmetric irradiation of the spine should be avoided to prevent growth disorder.

8.8.2 Radiation planning

For patients with central review the radiation plan will be provided by the GPOH-study office.

Details of field definitions are provided in the **radiotherapy manual**.
For TG-1 patients radiotherapy should begin latest by day 35 after the last dose of chemotherapy.
For TG-2 and TG-3 patients the decision to treat with radiotherapy is taken after restaging following two courses of OEPA and treatment should be started up to day 25 after the last dose of chemotherapy. Radiotherapy planning should be scheduled as soon as possible after the decision to treat and can be started already during chemotherapy to minimize delays.

8.8.3 Technical requirements

The following equipment is required:

- Treatment Simulator
- Computed Tomography
- 3D Treatment Planning System integrating 3D sectional imaging
- Linear Accelerators with photon energies of 4 - 6 (- 10) MV and high energy electrons
- Multileaf Collimators and/or conformal blocks for individual shielding

8.8.4 Side Effects of radiotherapy

Acute side effects:
Acute side effects as moderate **mucositis, skin reactions, hairloss, dry mouth, leucocytopenia, and thrombocyctopenia** during radiotherapy with low doses are rare and mostly temporary. Time and intensity of side effects may additionally depend on prior chemotherapy.

Long term side effects:
Radiosensitivity of normal tissues varies with age. Radiation effects are more more pronounced in young age (0-6 years) and pre-pubertal children compared to older children or adults. In general, the radiosensitivity of normal tissues, especially late reacting tissues, decreases with advancing age. Especially high radiosensitivity occurs during periods of growth. Side effects can be aggravated by certain drugs e.g. Adriamycin.

Radiation induced changes in organs and tissues (**e.g. hypothyroidism, premature menopause)** may develop after long latency periods and may not become clinically evident until puberty or adulthood.
Secondary malignancies (e.g. increased risk of breast cancer) may increase after 15 to 20 years.

8.9 SUPPORTIVE CARE

Management of febrile neutropenia is according to locally agreed guidelines.
In the UK, Portacath/lHickman line insertion prior to treatment is recommended to avoid extravasation particularly of adriamycin.

8.9.1 Antibacterial prophylaxis

All patients receive Trimethoprim during chemo- and radiotherapy and up to three
months after end of chemo-radiotherapy as per local/national guidelines.
If WBC is below 1000/ mm3, patients may receive, e.g. oral colistin sulfate optional as per local guidelines.

8.9.2 Prevention of GvH reaction / infection through blood transfusions

Transfusions of packed red cells or platelets should be leukocyte-depleted and irradiated with 30 Gy.

8.9.3 Antifungal prophylaxis

During chemotherapy and radiotherapy patients may receive antifungal prophylaxis according to local recommendations.

8.10 ONCOLOGICAL EMERGENCIES

8.10.1 Large mediastinal tumour

For patients with large mediastinal tumour the level of respiratory insufficiency should be determined.

- level 0: No respiratory insufficiency. No restriction of trachea or bronchi visible in X-ray and / or thorax CT. No venous congestion.
- level 1: Clinically no signs of respiratory insufficiency but radiological restriction of trachea or bronchi.
- level 2: Stridor and / or upper venous congestion (first sign among others headache).
- level 3: Orthopnoea

Only in patients up to level 1 a lymph node biopsy under local anaesthesia or a minithoracotomy in general anaesthesia can be performed. Before surgery it should be discussed with the anaesthetist that the patient may need prolonged mechanical ventilation for 1 – 2 days until the tumour has shrunk during treatment. **In all other cases before biopsy a pre-phase with Prednisone/prednisolone 30 – 60 mg / m2 (5 – 10 days) should be initiated.**

A therapeutic pleura puncture should be performed under local anaesthesia in case of respiratory insufficiency caused by a large pleural effusion. The same applies to a clinically relevant pericardial effusion.

8.10.2 Tumour lysis syndrome

In the rare case of tumour lysis syndrome (in patients with hyperuricemia or patients with bulky disease) the following is recommended:

- Hyperhydration with a liquid volume of 3000 (up to 5000 ml/m^2; maximal 7000 ml) per day.
- For forced diuresis the infusion should contain 10 mg Furosemide /1000 ml. Every 6 hours fluid balance should be calculated and if needed Furosemide should be applied additionally.
- The initial infusion should not contain KCl (addition of KCl only in patients with hypokalaemia only under strict indication with in short-term electrolyte checks).
- Urine alkalinization is not recommended as increasing the pH will reduce the solubility of phosphate.
- Prophylactically all patients should receive Allopurinol. In case of hyperuricaemia Rasburicase (Fasturtec® or a comparable drug) may be considered.

9 RELAPSE TREATMENT

9.1 RATIONALE OF THE RELAPSE THERAPY AS PROVIDED IN THE PROTOCOL

9.1.1 Results of previous studies

After 20 years disease-free survival (DFS) of the pooled first line studies DAL-HD 78 to DAL-HD 90 is 89% (Schellong und Riepenhausen, 2002). Therefore, studying patients with therapy failure is difficult due to the small number of cases.

The data of previous DAL / GPOH-HD studies show that patients with a relapse after primary therapy of Hodgkin's lymphoma have a realistic chance of cure by chemo-radiotherapy. In 1986 the DAL-ST-HD-86 study for second line treatment used salvage therapy consisting of alternating IEP (Ifosfamide, VP-16, Prednisone/prednisolone) and ABVD (Adriamycin, Bleomycin, Vinblastine, Dacarbazine) followed by radiotherapy. An updated analysis (Schellong et al 2005) of 176 patients with relapse / progression from DAL-HD 78 to GPOH-HD 95 studies describe an EFS of 57%, a DFS of 62% and an OS of 75% after 10 years (Fig. 8).

Fig. 8. Results of DAL ST-HD-86

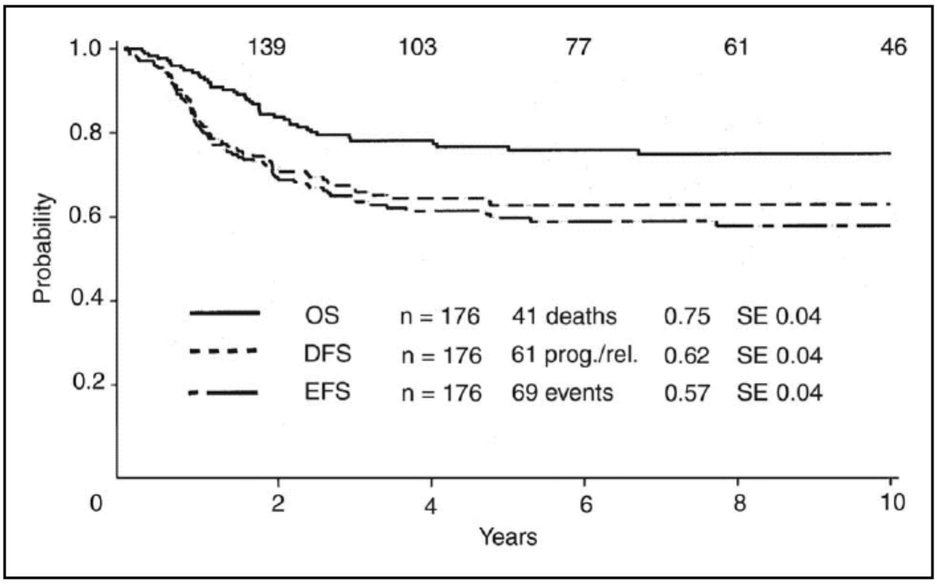

Probabilities of overall survival (OS), disease-free survival (DFS), and event-free survival (EFS) of the total group of 176 patients treated with salvage therapy ST-HD-86 until 10 years of follow-up after diagnosis of first recurrence. prog., progressions; rel., relapses.

9.1.2 Prognostic factors

In a multivariate analysis time of first failure was the only significant prognostic factor. Patients with progression show a DFS of 41% and an OS of 51% after 10 years. DFS for patients with early relapse was 55%, whereas DFS and OS for patients with late relapse were 86% and 90% (Fig. 9, Fig. 10). It is noteworthy that within the group of late relapse the patients of TG-1 performed better (DFS of 96% ± SE 4%) than those of TG-2 and TG-3.

Fig. 9. Overall survival according to prognostic groups (DAL ST-HD-86)

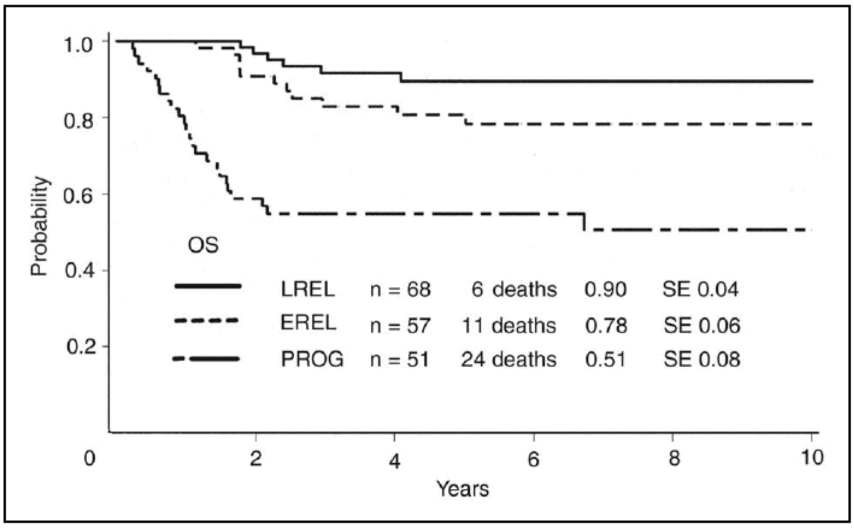

Probabilities of overall survival (OS) for the three prognostic groups until 10 years of follow-up. LREL, late relapses; EREL, early relapses; PROG, progressions.

Fig. 10. Disease free survival according to prognostic groups (DAL ST-HD-86)

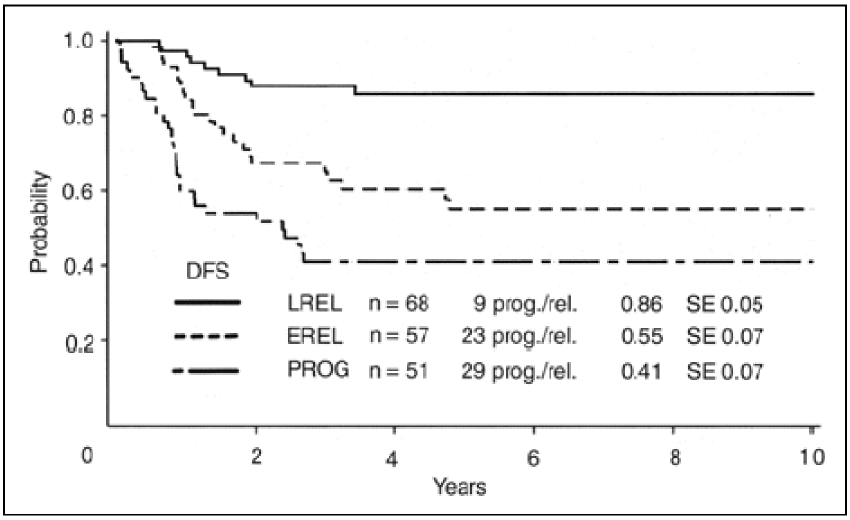

Probabilities of disease-free survival (DFS) for the three prognostic groups until 10 years of follow-up. LREL, late relapses; EREL, early relapses; PROG, progressions.

The dominant role of time to first treatment failure is confirmed (Josting et al, 2002a) in an analysis of 422 relapsed patients of the German adult Hodgkin lymphoma therapy studies. Additional independent prognostic factors were stage of relapse and anaemia in relapse.

Patients with primarily refractory disease or multiple relapses have a particularly bad prognosis (Hudson & Donaldson, 1999). Josting et al. (2000) reported freedom from second failure (FF2F) rates of 17% and OS of 26% after 5 years in 206 primarily progressive patients.

9.1.3 High dose chemotherapy and autologous stem cell transplantation in relapsed patients

There are two randomised studies comparing conventional chemotherapy with high dose chemotherapy with autologous stem cell transplant (SCT) in patients with relapsed Hodgkin's lymphoma. Linch et al. (1993) showed significantly better EFS for 40 adult patients (20 mini-BEAM, 20 BEAM and SCT), but no better OS for the group of transplanted patients. Schmitz et al. (2002) compared BEAM with autologous SCT with further two cycles of DexaBEAM in 117 patients (16-60 years, median: 32 years) with chemo-sensitive relapse (complete or partial remission after 2 DexaBEAM). After 3 years freedom from treatment failure (FFTF) was significantly better for patients with autologous SCT than for patients with conventional chemotherapy (55% vs. 34%, $p<0.02$). This applied for patients with early relapse (<12 month: 41% vs. 12%, p=0,008) and also for those with late relapse (≥12 months: 75% vs. 44%, p=0,02), while patients with multiple relapses did not benefit from SCT (34% vs. 44%). However, OS was not significantly different (71% vs. 65%). Transplant related mortality is < 5-10% (Sureda et al, 2001a) for adult patients in large series.

Series with high dose chemotherapy and SCT in paediatric relapse patients are small and report EFS rates between 31 to 67% (Baker et al., 1999; Frankovich et al., 2001; Verdeguer et al., 2000; Williams et al., 1993). In our study group 38 children have been treated with high dose chemotherapy plus autologous SCT. Their EFS was 42% after 4.8 years, thus well within the range of other international studies (Zintl et al, 2002). It appears that outcome of paediatric and adult patients are similar with high dose chemotherapy with autologous SCT.

Disease status at transplantation is an important prognostic factor (Schmitz et al., 1993). A small study (Becherer et al. 2002) suggests that patients with negative PET prior to high dose therapy have an excellent prognosis while patients with positive PET finding relapse. This hypothesis should be confirmed with sufficient patient numbers.

9.2 RELAPSE TREATMENT WITHIN EURONET-PHL-C1

We build on the experience with DAL-ST-HD-86 salvage treatment using alternating 2 (IEP-ABVD) and radiotherapy. Patients will now be stratified according to the dominant prognostic factor, namely time to first treatment failure. Low risk patients continue to receive the DAL-ST-HD-86 standard treatment. Based on results obtained in the adult setting, high dose chemotherapy with autologous SCT is introduced for all high risk patients in whom outcome is not yet satisfactory. In intermediate risk patients early response assessment with FDG-PET is used: Patients with adequate response receive the standard treatment while patients with inadequate response will receive intensified treatment with high dose chemotherapy and autologous SCT with or without radiotherapy.

9.2.1.1 Eligibility criteria second line treatment

Inclusion criteria

Patients will be registered for second line treatment if the following criteria are met:

- progression or relapse to first line treatment
- first line treatment according to protocols of studies DAL-HD 90, GPOH-HD 95, GPOH-HD 2002 Pilot, EURONET-PHL-C1 or similar treatment
- written informed consent of the patient and/or the patient's guardian according to national laws

Country-specifically, very young patients may be excluded to comply with national laws or formal insurance requirements (e.g. Germany patients under the age of 6 years).

Exclusion criteria

Patients with one of the following factors are excluded:

- second line pre-treatment of Hodgkin's lymphoma differing from study protocol (except recommended pre-phase therapy of a large mediastinal tumour)
- known hypersensitivity or contraindication to study drugs
- diagnosis of lymphocyte predominant Hodgkin's lymphoma
- prior chemo- or radiotherapy (except first line treatment for Hodgkin's lymphoma)
- concomitant other malignant tumours
- pregnancy and / or lactation

- girls with child bearing potential without effective contraception (oral contraception, intrauterine devices, barrier method of contraception in conjunction with spermicidal jelly or surgical sterile)
- Current or recent (within 30 days prior to start of trial treatment) treatment with another investigational drug or participation in another investigational trial
- severe concomitant diseases (e.g. immune deficiency syndrome)
- known HIV positivity
- expected non-compliance of patient or parents / guardian

If exclusion criteria occur the study centre should be informed.

<u>Subsequent exclusion of patients</u>

Patients are excluded from the study after registration if

- documents or material ascertained before study inclusion show that an exclusion criterion was fulfilled or an inclusion criterion was not met
- the patient withdraws his/her consent to further study participation

If the diagnosis of Hodgkin's lymphoma is not confirmed by the reference pathologist the patient is still documented in the scope of the study. Patient's physician decides then together with the patient/parents or guardian on the further therapy.

The study chairpersons decide on the exclusion together with the biometrician of the study. A trial site may request the subsequent exclusion of a patient by sending a written detailed account of the reasons to the study office.

Subsequent exclusion of a patient differs from an individual therapy withdrawal. In the latter case the treatment of the patient according to protocol is terminated, but follow-up and documentation (data collection in the CRF) is continued according to protocol and the patient appears in all relevant analyses.

9.2.1.2 Diagnostics for patients in first relapse therapy

Every progression or relapse should be confirmed by biopsy before treatment starts (exception see 9.2.1.1 large mediastinal tumour).

After diagnosis of relapse / progression all patients (except for patients who are diagnosed with a progression during a regular restaging of the first line therapy) get a complete staging according to the principles in first line therapy (see chapter 6). **Patients with diagnosed relapse / progression should get an FDG-PET before starting therapy** (except emergencies).

After the first double cycle (IEP-ABVD) early response assessment is performed according to the principles in first line therapy (see chapter 6). **Exception**: Patients with late relapse after first line treatment according to TG-1.

For diagnostics under chemotherapy see Chapter 6.2.

Prior to autologous stem cell transplantation (SCT) a complete restaging of all regions involved at relapse including FDG-PET is performed and the following investigations are recommended:

- search for infection foci
  - ENT examination incl. X-ray of paranasal sinus
  - dermatological examination
  - dental examination
  - ophthalmologic examination
  - gynaecological consultation if necessary
- functional examinations
  - EEG
  - Echocardiography
  - lung function tests
  - creatinine clearance
- laboratory diagnostics
  - blood counts
  - liver enzymes, bilirubin, alkaline phosphatase
  - creatinine, urea, uric acid
  - coagulation status
  - HLA antibodies
- Infection diagnostics
  - PCR for EBV, hepatitis B and C, CMV, HSV, HHV-6, adenoviruses
  - isolation of CMV, adenoviruses, papilloma-viruses from urine
  - serology for toxoplasmosis, candida, lues

After autologous SCT patients are restaged on **day 50 – 54**.

Follow-up in relapse patients follows the schema for first line patients (cp. Chapter 6.7.4).

9.2.1.3 *Treatment plan for second line treatment*

The treatment plan for second line treatment is summarized in Fig. 11.

Fig. 11. Treatment plan for second line treatment

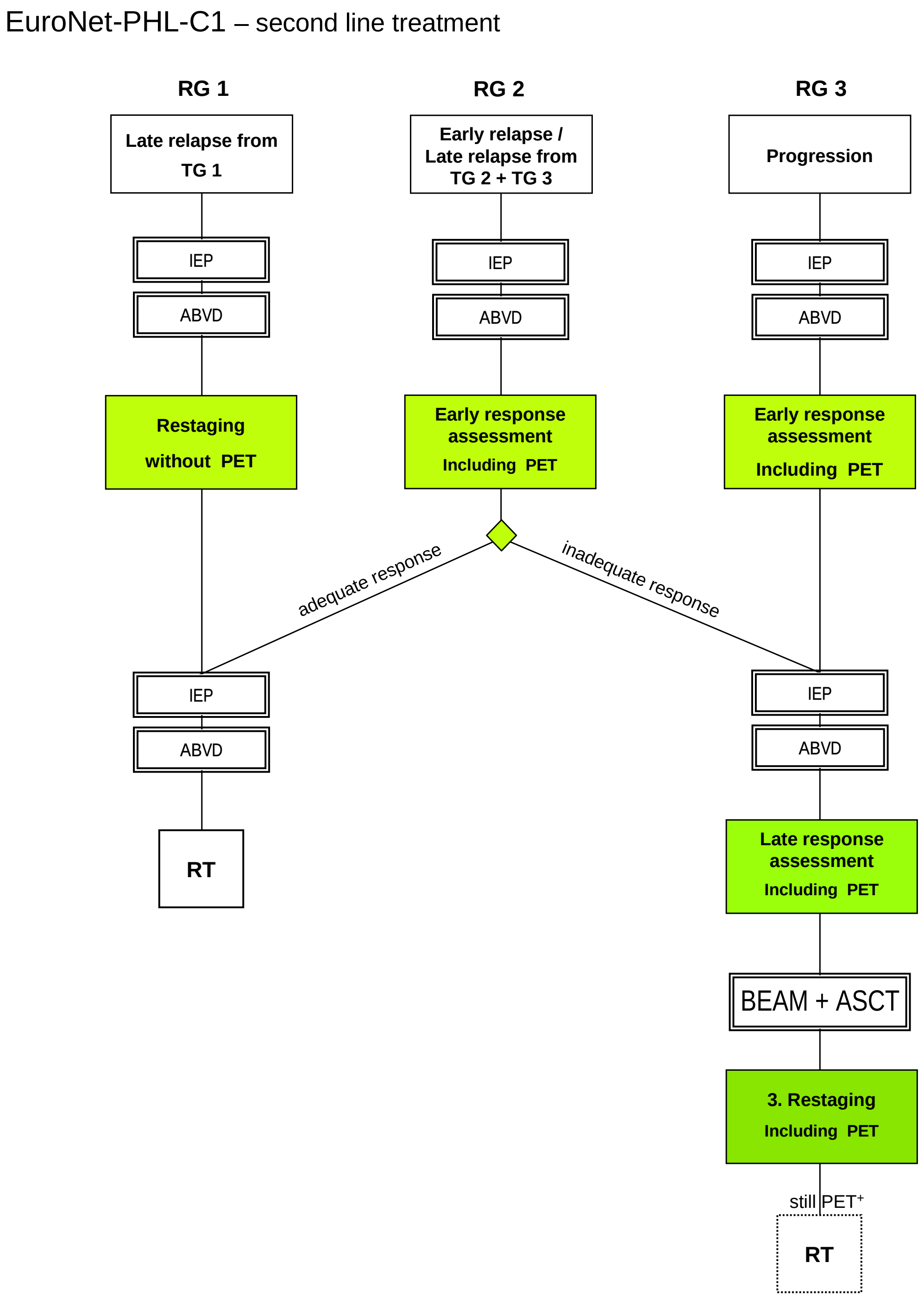

Stem cell aphaeresis after IEP as soon as indication for HDCT is given.

All patients receive two double cycles of (IEP-ABVD). Thereafter:

- Patients with **late relapse** (i.e. relapse more than 12 months after end of first line treatment) **after TG-1 first line treatment** (**RG1**) then receive modified involved field radiotherapy.
- Patients with **progression** (i.e. progression of disease during or up to three months after end of first line treatment) (**RG3**) then receive high dose chemotherapy BEAM with autologous SCT. Stem cell aphaeresis after the first IEP cycle.
- **All other patients** (i.e. early relapse or late relapse after TG-2 or TG-3 first line treatment) (**RG2**) are treated according to early response assessment after the first double cycle of (IEP-ABVD):
  - Patients with **adequate response** (cf. chapter 7.3) receive modified involved field radiotherapy.
  - Patients with **inadequate response** (cf. chapter 7.3) receive high dose chemotherapy BEAM with autologous SCT. Stem cell aphaeresis after second IEP cycle.

Note: Rare cases in whom radiotherapy is deemed unacceptable due to previous irradiation receive high dose chemotherapy BEAM with autologous SCT. Stem cell aphaeresis after the first IEP cycle.

Patients with progression during second line therapy are off study. In these patients further treatment should be discussed with the national study chairperson or trial coordinator.

9.3 CHEMOTHERAPY PLANS IN SECOND LINE TREATMENT

The first cycle should start immediately after completion of diagnostic work up (For oncological emergencies in patients with initially large tumour mass please see chapter 8.10). All other cycles should be given without delays as soon as the following criteria are met:

- general condition satisfactory
- WBC over 2,000 / mm^3
- ANC over 500 / mm^3
- platelets over 80,000 / mm^3
- no contraindications against drugs used

Severe side effects are not expected with IEP-ABVD. Chemotherapy should only be interrupted in case of severe inter-current infections. In parallel to chemotherapy patients receive hyperhydration.

9.3.1 IEP

Prior to IEP the creatinine clearance (GFR) should be within the normal range. To prevent urinary tract toxicity of Ifosfamide the uroprotector Mesna is given (see Table 18).

Table 18 IEP scheme

**Drug**	**day**	**DD (mg/m² per day)**	**Application**
Ifosfamide	1 - 5	2000	i.v., 22-hour infusion (experience of the HD-ST-86 study)
Mesna*	1	700	Bolus
Mesna*	1 - 7	2000	i.v., 24-hour infusion
Etoposide/Etopophos	1 - 5	125	i.v., 1-2 hour infusion
Prednisone/ Prednisolone	1 - 5	100	p.o. in 3 doses

* for UK centres: **Ifosfamide by 1 hour infusion** Intravenous hydration (with glucose/saline + potassium chloride 20 mmol/l.)*, containing **mesna** at **120%** (mg/mg) of the prescribed **daily ifosfamide dose.** Infuse this solution at a rate of 3l / m^2 / 24 hours, commencing 3 hours before the first ifosfamide dose and continuing for a **minimum** of 12 hours after completion of the last ifosfamide infusion. Recommendations are of 24 hours post hydration.

Therapy should be continued after IEP by or before day 22 after start of cycle.

9.3.2 ABVD

Besides the criteria mentioned above heart and lung function should be normal before starting on ABVD. If this is not the case please contact the study centre.

Table 19 ABVD scheme

**Drug**	**Day**	**DD (mg/m²)**	**application**
Doxorubicin	1, 15	25	i.v., 1-6 hour infusion
Bleomycin	1, 15	10	i.v., Bolus
Vinblastine	1, 15	6	i.v., Bolus
Dacarbazine	1, 15	375	i.v., 15 – 30 minutes infusion

Therapy should be continued after ABVD on day 29.

9.4 BEAM HIGH-DOSE CHEMOTHERAPY AND AUTOLOGOUS SCT

BEAM is used as high-dose chemotherapy (Table 20). In patients with restrictive lung disease, BCNU dose is reduced by 1/3 or other alternative regimen (e.g. CVB) can be used after consultation of the study chairpersons.

In general unselected peripheral blood stem cells should be used as stem cell source. Stem cell should be collected after the first or second IEP cycle.

Patients must be free of infections for 4 days before high-dose chemotherapy.

Table 20 BEAM high-dose chemotherapy

Drug	DD (mg/m²/day)	-7	-6	-5	-4	-3	-2	-1	0
Carmustine (BCNU*)	300	X							
Etoposide***	200		X	X	X	X			
Cytarabine	400**		X	X	X	X			
MEL	140						X		
SCT									X

* in case of restrictive lung disease 200 mg/m^2 BCNU

** i.v., in 2 doses each as 30-min. infusion in 12-hour interval

*** or Etopophos.

Supportive care follows the guidelines of the respective transplantation centre.

At day 50-54 a restaging including FDG-PET will be performed.

9.5 SIDE EFFECTS OF THE DRUGS USED IN SECOND LINE THERAPY

For side effects of drugs also used in primary therapy see chapter 8.7. as well as chemotherapy drug monographs

<u>Carmustine BCNU:</u> bone marrow depression, gastrointestinal haemorrhage, retinitis, interstitial pneumonia, gynaecomastia, allergy, secondary malignant tumours

Bleomycin: pneumonitis, lung fibrosis, muscle pain, chills, allergic reactions, Raynaud's syndrome, hyperkeratosis, oedemas, striae

Ifosfamide: tubular nephropathy, renal excretion dysfunction, nausea, vomiting, hemorrhagic cystitis, convulsions, secondary malignant tumours

Melphalan: bone marrow depression, haemolytic anaemia, lung fibrosis, allergy

9.6 RADIOTHERAPY IN RELAPSE PATIENTS

9.6.1 Indication for radiotherapy

In patients not requiring high-dose chemotherapy all involved lymph nodes at time of relapse are irradiated with generous margins.
After BEAM and SCT patients receive radiotherapy to all regions involved in relapse if at least one initially involved region is FDG-PET positive at the time of restaging on day 50 – 54 after transplantation.

9.6.2 Radiation volume and radiation dose

The radiation volume (PTV_1) is defined by the involvement at the time of relapse / progression diagnosis similar to radiotherapy in first line treatment (cp. chapter 8.8 and **radiotherapy manual**).
The radiation dose is adjusted individually according to previous radiotherapy:

- Patients without previous radiotherapy or with relapse localization in previously not irradiated lymph nodes get an IF-RT of 25-30 Gy.
- In case of relapse in lymph nodes which were previously irradiated with 20 Gy, 20 – 25 Gy are given.
- In case of relapse in a lymph nodes which were previously irradiated with more than 20 Gy, up to 20 Gy are given.

Radiotherapy planning for second line treatment should be discussed with the reference radiotherapist or the national study chairperson or trial co-ordinator. In patients with extensive radiotherapy during first line treatment individual adaptations may be necessary which are not specifically addressed in this protocol.

10 ORGANISATION

10.1 INFORMED CONSENT

All patients admitted to the trial site with suspected or diagnosed Hodgkin's lymphoma are to be considered for enrolment and the inclusion and exclusion criteria to be checked.

<u>NOTE</u>: If any eligibility criteria can be checked only by study specific invasive procedures that are not part of standard care, eligibility check has to be finished after receiving written informed consent from the patient and his/her parents or guardian. No study related procedures are allowed before informed consent has been obtained.

The investigator must explain to each patient and his/her parents or guardian the nature of the study, its purpose, the procedures involved, the expected duration, the potential risks and benefits involved and any discomfort it may entail. Each patient and his/her parents or guardian must be informed that participation in the study is voluntary and that he/she may withdraw from the study at any time and that withdrawal of consent will not affect his/her subsequent medical treatment or relationship to the treating physician. The patient and his/her parents or guardian should be provided with enough time to think about the participation in the study.

The informed consent is documented on a standard form, written in non-technical language. The patient (if applicable) and his/her parents or guardian should read the statement and consider his/her decision before signing and dating the document, and should be given a copy of the signed document. No patient can enter the study before informed consent in compliance with the pertinent national laws has been obtained.

Informed consent forms are provided in the investigator site file. The signed informed consent form has to be duplicated to provide one version to the trial subject and one original for the trial site which has to be filed in the investigator site file.

The informed consent of the patient and his/her parents or guardian must also refer specifically to the assessment and processing of data on the patients' health. The patient and his/her parents or guardian are to be informed explicitly on the purpose and extent of the data collection and the use of his/her personal data, especially the health-related data.

<u>NOTE</u>: The following procedures apply only to German patients.
The patient and his/her parents or guardian are additionally asked to give consent that the personal and health related data is transmitted to the:

- *German Childhood Cancer Registry in Mainz (Dr. Kaatsch),*
- *Registry for radiogenic long-term sequelae (Prof. Dr. Willich/ Prof. Dr. Schuck) and*

- *In case of relapse therapy involving stem cell transplantation, to the paediatric registry for stem cell transplantation (Prof. Dr. Klingebiel).*

Furthermore, patients in trial sites associated with the GPOH-HD study group and their parents or guardian are informed on the central staging and response assessment performed in the GPOH-HD study office. For this purpose, all medical experts involved in the central review process need to have access to all required medical files and images. The aims and purposes of this data collection are explained to the patient and their parents or guardian.

One of the main objectives of the EuroNet-PHL-C1 trial is to reduce long-term sequelae of therapy without compromising cure rates. To monitor this, complete follow-up data are crucial. Therefore, the patient and his/her parents or guardian may additionally be asked for their consent to be contacted directly by the corresponding study office, in case no follow-up has been provided by the responsible trial site.

10.1.1 Withdrawal of informed consent

Patients and their parents or guardian may withdraw their consent to participate at any time of the trial without giving the reason for it. Nevertheless, the patient and his/her parents or guardian should be asked for the reason of the premature termination but they should also be aware, that they need not answer this question. The patient and his/her parents or guardian must be informed that choosing not to participate or to withdraw the consent will not affect his/her subsequent medical treatment or relationship to the treating physician.
Date of enrolment and date of and reason for withdrawal (if available) are to be documented in any case. The patient and his/her parents or guardian is to be informed that in case of revocation of his/her consent, the data already stored will be used further, as may be necessary:

to assess effects of the study drug to be tested

to guarantee that the interests of the patient are not impaired

to comply with the regulatory requirements.

10.2 PRIMARY THERAPY

10.2.1 Procedures for all treatment groups

**Registration**	Immediately after informed consent has been obtained and the diagnostic procedures (cf. chapter 6) have been completed and **before start of therapy**, the investigator **registers** the patient in the respective study office (**fax of the patient registration form**).<br><br>The investigator receives a return fax with the **assigned patient ID**. The patient ID has **to be entered in the patient identification list (PIL) in the investigator site file** together with the patient‘s name and date of birth.<br><br>NOTE: In Germany, the patient should be registered simultaneously in the German Childhood Cancer Registry in Mainz, according to the common procedure for all paediatric-oncological studies.
**Staging**	Staging is performed according to chapter 6.<br><br>*Procedures for trial sites with central review*<br>Immediately after registration, at the latest **until first day of therapy**, the investigator has to send all required documents and images concerning the initial staging to the corresponding study office. These documents are an **indispensable condition** for the central staging and the **assignment to the treatment group**. If the patient is not assigned to a treatment group until the start of the second chemotherapy cycle the patient’s therapy according to protocol is endangered. Thus, this **procedure is time-critical**.<br><br>As soon as possible after central review, the investigator receives the reference staging documentation and results necessary for treating according to protocol.<br><br>*Procedures for trial sites without central review*<br>Once the diagnosis is confirmed and the staging investigations completed, this information is documented on the staging forms and sent to the corresponding study office. The staging results are checked, and then the investigator receives the information and documentation required for treating according to protocol.
**OEPA**	**Immediately after** completion of the **staging procedures**, the first **OEPA**

**chemotherapy**	**cycle is started**.<br><br>All patients receive **2 OEPA cycles**, as described in section 8.4.1.<br><br>After completion of OEPA chemotherapy, the respective chemotherapy and chemotherapy-toxicity forms have to be sent to the corresponding study office immediately.
**Early response assessment (first restaging)**	The dates for imaging diagnostics for **early response assessment (first restaging)** (sonography, CT/MRI, FDG-PET) are **time-critical** and therefore have to be fixed **by the start of the second OEPA cycle at the latest**.<br><br>Only initially involved regions are examined (except if progression is suspected). It is crucial that the **FDG-PET** is performed **on day 14, at the latest on day 17 after the last application of chemotherapy** of the second OEPA cycle.<br><br>*<u>Procedures for trial sites with central review</u>*<br><br>All **required documents and images** concerning the **early response assessment (first restaging)** have to be sent to the corresponding study office **at the latest until day 21** after the last application of chemotherapy in the second OEPA cycle.<br><br>As soon as possible after central review, the investigator receives the information **whether radiotherapy is indicated** and - if so - the **final reference radiotherapy plan**.<br><br>NOTE: It is not necessary to wait for the results of the central review before starting the next chemotherapy cycle (TG-2 and TG-3).<br><br>*<u>Procedures for trial sites without central review</u>*<br>Early response assessment is the responsibility of the local investigator. Documentation should be completed and returned to the corresponding study office.

10.2.2 Further procedures in treatment group 1 (TG-1)

**Radiotherapy**	If indicated **radiotherapy must start immediately** at **latest on day 35** after the last application of chemotherapy in the second OEPA cycle.<br><br>**The investigator is responsible** for forwarding the radiotherapy and the radiotherapy-toxicity forms to the responsible radiotherapist before start of radiotherapy. After radiotherapy, the radiotherapist completes the appropriate forms and returns them to the investigator immediately.
**Follow-up**	The **first follow-up** examination is scheduled **6 weeks after end of therapy** (i.e. last radiation day or last day of chemotherapy application for patients without radiotherapy).<br>After the first follow-up examination, the radiotherapy and radiotherapy toxicity form (if applicable), as well as the first follow-up form should be sent to the corresponding study office as soon as possible.<br>Further follow-up visits are scheduled according to section 6.7.4.<br>Follow-up forms are due at latest four weeks after the follow-up visit has been performed.<br>In case of relapse, progression after end of therapy or in case of death the study office should be informed immediately by completing and sending a follow-up form.

10.2.3 Further procedures in treatment groups 2 and 3 (TG-2 + TG-3)

**COPDAC chemotherapy**	**The third chemotherapy cycle (COPDAC) has to start on the day of early response assessment (first restaging) PET**.<br><br>According to treatment group the patients receive 2 or 4 cycles of COPDAC, as described in section 8.4.2.<br><br>After completion of chemotherapy, the chemotherapy as well as the chemotherapy-toxicity forms have to be sent to the corresponding study office immediately.

**Late response assessment (second restaging)**	The dates for imaging diagnostics for the **late response assessment (second restaging)** (sonography, CT / MRI) are time-critical and therefore have to be fixed **at latest by start of the fourth respectively sixth chemotherapy cycle.** This also applies for the dates of radiotherapy planning and start of radiotherapy.<br><br>Only initially involved regions are examined (except if progression is suspected).<br><br>*Procedures for trial sites with central review*<br><br>All required documents and images concerning the late response assessment (second restaging) have to be sent to the corresponding study office at latest by day 21 after the last application of Prednisone/Prednisolone in the fourth resp. sixth chemotherapy cycle.<br><br>As soon as possible after central review, the investigator receives the information on the results of the central tumour conference<br><br>*Procedures for trial sites without central review*<br>End of treatment response assessment is the responsibility of the local investigator. Documentation should be completed and returned to the corresponding study office.
**Radiotherapy**	If indicated **radiotherapy must start immediately after completion of the late response assessment (second restaging) examinations if no progression occurs**, since the final reference radiotherapy plan based on the findings of early response assessment (first restaging) has already been sent in advance.<br><br>**The investigator is responsible** for forwarding the radiotherapy and the radiotherapy-toxicity forms to the responsible radiotherapist before start of radiotherapy. After radiotherapy, the radiotherapist completes the appropriate forms and returns them to the investigator immediately.
**Follow-up**	The **first follow-up** examination is scheduled **6 weeks after end of therapy** (i.e. last radiation day or last day of chemotherapy application for patients without radiotherapy).<br>After the first follow-up examination, the radiotherapy and radiotherapy toxicity form (if applicable), as well as the first follow-up form should be sent

	to the corresponding study office as soon as possible.<br>Further follow-up visits are scheduled according to section 6.7.4.<br>Follow-up forms are due at latest four weeks after the follow-up visit has been performed.<br>In case of relapse, progression after end of therapy or in case of death the study office should be informed immediately by completing and sending a follow-up form.

10.3 PATIENTS WITH FIRST RELAPSE THERAPY

10.3.1 Procedures for all patients

**Registration**	Immediately after informed consent has been obtained and the diagnostic procedures (cf. chapter 9.2.1.2 and 6) have been completed and **before start of therapy**, the investigator **registers** the patient in the respective study office (**fax of the patient registration form for relapse therapy**).<br>If the patient has not been treated in primary therapy within the scope of EuroNet-PHL-C1 and therefore has no study specific patient ID, the investigator receives a return fax with the **assigned patient ID**. The patient ID has **to be entered in the patient identification list (PIL) in the investigator site file** together with the patient‘s name and date of birth.
**Staging**	Staging is performed according to chapter 6.<br>*Procedures for trial sites with central review*<br>Immediately after registration, at the latest **until first day of therapy**, the investigator has to send all required documents and images concerning the initial staging to the corresponding study office. These documents are an **indispensable condition** for the central staging<br>As soon as possible after central review, the investigator receives the reference staging results necessary for treating according to protocol (provisional **reference radiotherapy plan**) as well as documents necessary for further documentation.<br>*Procedures for trial sites without central review*

	Once the diagnosis of relapse or progression is confirmed and the staging investigations completed the relapse staging is documented on the relapse staging form and sent to the corresponding study office. The staging results are checked, and the investigator receives the information necessary for treating according to protocol (provisional **reference radiotherapy plan**) as well as documents necessary for further documentation.
**IEP-ABVD chemotherapy**<br><br>**(1st double cycle)**	**Immediately after** completion of the **staging procedures**, the **IEP-ABVD** chemotherapy is **started**.<br><br>All patients receive a **double-cycle IEP-ABVD**, as described in section 9.3.<br><br>After completion of IEP-ABVD chemotherapy, the respective chemotherapy and chemotherapy-toxicity forms have to be sent to the corresponding study office immediately.<br>Note: In patients with progression after first line treatment, stem cell aphaeresis might be performed after the first IEP, in all other patients stem cell apheresis will be performed after indication for autologous SCT is given, i.e. after the second IEP, when the results of the early response assessment (first restaging) are definitive.

10.3.2 Procedures for early response assessment (first restaging)

**Patients with late relapse after treatment within TG-1**	The dates for imaging diagnostics for **early response assessment (first restaging)** (sonography, CT/MRI, **no** FDG-PET!) are **time-critical** and therefore have to be fixed **by the start of the ABVD cycle at the latest**.<br><br>Only initially involved regions are examined (except if progression is suspected).<br><br>*Procedures for trial sites with central review*<br><br>All **required documents and images** concerning the **early response assessment (first restaging)** have to be sent to the corresponding study office **at the latest until day 21** after the last application of chemotherapy in the **ABVD** cycle.<br><br>As soon as possible after central review of the early response assessment (first restaging), the investigator receives the **final reference radiotherapy**

	**plan**.<br><br><u>NOTE</u>: It is not necessary to wait for the results of the central review before starting the next chemotherapy cycle.<br><br>*<u>Procedures for trial sites without central review</u>*<br>Early response assessment is the responsibility of the local investigator. Documentation should be completed and returned to the corresponding study office.
**Patients with early relapse or with (late relapse after treatment within TG-2 or TG-3):**<br><br>**<u>Early response assessment</u>**	The dates for imaging diagnostics for **early response assessment (first restaging)** (sonography, CT/MRI, **FDG-PET**) are **time-critical** and therefore have to be fixed **by the start of the ABVD cycle at the latest**.<br><br>Only initially involved regions are examined (except if progression is suspected). It is crucial that the **FDG-PET** is performed **on day 14, at the latest on day 17 after the last application of chemotherapy** of the ABVD cycle.<br><br>*<u>Procedures for trial sites with central review</u>*<br><br>All **required documents and images** concerning the **early response assessment (first restaging)** have to be sent to the corresponding study office **at the latest until day 21** after the last application of chemotherapy in the **ABVD** cycle.<br><br>As soon as possible after central review, the investigator receives the information whether autologous SCT is indicated, and – if not so - the **final reference radiotherapy plan**. If autologous SCT is indicated, patients need stem cell apheresis after the second IEP.<br><br><u>NOTE</u>: It is not necessary to wait for the results of the central review before starting the next chemotherapy cycle.<br><br>*<u>Procedures for trial sites without central review</u>*<br>Response assessment is the responsibility of the local investigator. Documentation should be completed and returned to the corresponding study office.
**Patients with progression:**<br><br>**<u>Early response assessment</u>**	The dates for imaging diagnostics for **early response assessment (first restaging)** (sonography, CT/MRI, **FDG-PET**) are **time-critical** and therefore have to be fixed **by the start of the ABVD cycle at the latest**.<br><br>Only initially involved regions are examined (except if progression is

	suspected). It is crucial that the **FDG-PET** is performed **on day 14, at the latest on day 17 after the last application of chemotherapy** of the ABVD cycle.<br><br>*Procedures for trial sites with central review*<br><br>All **required documents and images** concerning the **early response assessment (first restaging)** have to be sent to the corresponding study office **at the latest until day 21** after the last application of chemotherapy in the **ABVD** cycle.<br><br>As soon as possible after central review, the investigator receives the information on the results of the central tumour conference.<br><br>NOTE: It is not necessary to wait for the results of the central review before starting the next chemotherapy cycle.<br><br>*Procedures for trial sites without central review*<br>Response assessment is the responsibility of the local investigator. Documentation should be completed and returned to the corresponding study office.

10.3.3 Further procedures for patients with late relapse after therapy within TG-1 or for patients with adequate response after early relapse or (late relapse after therapy within TG-2 or TG-3)

**IEP-ABVD chemotherapy**<br><br>**(2nd double cycle)**	**The second double cycle IEP-ABVD has to start immediately after restaging**. All patients receive a **double-cycle IEP-ABVD**, as described in section 9.3.<br><br>After completion of chemotherapy, the chemotherapy as well as the chemotherapy-toxicity forms have to be sent to the corresponding study office immediately.
**Radiotherapy**	**Radiotherapy must start as soon as possible after completion of the second IEP-ABVD without further restaging**, since the final reference radiotherapy plan based on the findings of early response assessment (first restaging) has already been sent in advance.<br><br>**The investigator is responsible** for forwarding the radiotherapy and the

	radiotherapy-toxicity forms to the responsible radiotherapist before start of radiotherapy. After radiotherapy, the radiotherapist completes the appropriate forms and returns them to the investigator immediately.
**Follow-up**	The **first follow-up** examination is scheduled **6 weeks after end of therapy** (i.e. last radiation day).<br>After the first follow-up examination, the radiotherapy and radiotherapy toxicity form (if applicable), as well as the first follow-up form should be sent to the corresponding study office as soon as possible.<br>Further follow-up visits are scheduled according to section 6.7.4.<br>Follow-up forms are due at latest four weeks after the follow-up visit has been performed.<br><br>In case of relapse, progression after end of therapy or in case of death the study office should be informed immediately by completing and sending a follow-up form.

10.3.4 Further procedures for patients with progression or with inadequate response after early relapse or (late relapse after therapy within TG-2 or TG-3)

**IEP-ABVD chemotherapy**<br><br>**(2nd double cycle)**	**The second double cycle IEP-ABVD has to start immediately after early response assessment (first restaging)**. All patients receive a **double-cycle IEP-ABVD**, as described in section 9.3.<br><br>After completion of chemotherapy, the chemotherapy as well as the chemotherapy-toxicity forms have to be sent to the corresponding study office immediately.
**Late response assessment (second restaging)**	The dates for imaging diagnostics for the **late response assessment (second restaging)** (sonography, CT / MRI, **FDG-PET**) are time-critical and therefore have to be fixed **at latest by start of the ABVD cycle.** This also applies for the dates of radiotherapy planning and start of radiotherapy.<br><br>Only initially involved regions are examined (except if progression is suspected). It is crucial that the **FDG-PET** is performed **on day 14, at the latest on day 17 after the last application of chemotherapy** of the ABVD cycle.

	Procedures for trial sites with central review<br><br>All **required documents and images** concerning the **late response assessment (second restaging)** have to be sent to the corresponding study office **at the latest until day 21** after the last application of chemotherapy in the **ABVD** cycle.<br><br>As soon as possible after central review, the investigator receives the information on the results of the central tumour conference.<br><br>NOTE: It is not necessary to wait for the results of the central review before starting the high dose chemotherapy.<br><br>*Procedures for trial sites without central review*<br>Response assessment is the responsibility of the local investigator. Documentation should be completed and returned to the corresponding study office.
**Autologous SCT**	Immediately after **late response assessment (second restaging),** the patients receive BEAM high-dose chemotherapy with autologous stem cell transplantation (SCT).<br><br>After completion of SCT, the chemotherapy and SCT as well as the chemotherapy-toxicity forms have to be sent to the corresponding study office immediately.
**Third restaging**	A third restaging (**Restaging after SCT**) with imaging diagnostics (sonography, CT/MRT, FDG-PET) of all regions initially involved in relapse is performed between day 50 – 54 after application of stem cells. Dates for this have to be fixed timely.<br><br>*Procedures for trial sites with central review*<br><br>All **required documents and images** concerning the **third restaging** have to be sent to the corresponding study office **at the latest until day 65** after the stem cell application.<br><br>As soon as possible after central review, the investigator receives the information **whether radiotherapy is indicated** and - if so - the **final reference radiotherapy plan**.<br><br>*Procedures for trial sites without central review*<br>Response assessment is the responsibility of the local investigator.

	Documentation should be completed and returned to the corresponding study office.
**Radiotherapy**	If indicated **radiotherapy must start as soon as possible after completion of the third restaging examinations if no progression occurs**.<br><br>**The investigator is responsible** for forwarding the radiotherapy and the radiotherapy-toxicity forms to the responsible radiotherapist before start of radiotherapy. After radiotherapy, the radiotherapist completes the appropriate forms and returns them to the investigator immediately.
**Follow-up**	For patient with radiotherapy, the **first follow-up** examination is scheduled **6 weeks after end of therapy** (i.e. last radiation day).<br>After the first follow-up examination, the radiotherapy and radiotherapy toxicity form (if applicable), as well as the first follow-up form should be sent to the corresponding study office as soon as possible.<br><br>For patients without radiotherapy, the third restaging is equivalent to the first follow-up.<br><br>Further follow-up visits are scheduled according to section 6.7.4.<br>Follow-up forms are due at latest four weeks after the follow-up visit has been performed.<br><br>In case of relapse, progression after end of therapy or in case of death the study office should be informed immediately by completing and sending a follow-up form.

10.4 PREMATURE TERMINATION OF THERAPY OR FOLLOW-UP

Any premature termination of the trial therapy as well as any premature termination of follow-up has to be documented by the responsible investigator. If possible, date, circumstances of and reasons for the termination should be documented in detail, and communicated to the corresponding study office.

10.4.1 Premature termination of trial therapy

The premature termination of trial therapy may be considered for the following reasons:

adverse / serious adverse event(s)
no response to therapy according to protocol criteria
excessive toxicity
at the discretion of the investigator, for reasons of medical prudence
severe protocol violation
non-compliance of the patient
administrative problems

In addition, the trial therapy may be terminated on request of the patient, in case of withdrawal of consent or in case of lost to follow-up.

Premature termination should be avoided. In case of premature termination of therapy, reasons/circumstances and if applicable the final status should be documented. If the patient does not withdraw the consent for further follow-up, he or she should be followed-up as planned.

10.4.2 Premature termination of follow-up

Patients in whom no follow-up has been received for more than two years are directly contacted by the corresponding study office (if consent to this procedure has been given).

NOTE: In Germany, the German Childhood Cancer Registry in Mainz may be asked for current residence.

10.5 ORGANISATION OF FDG-PET EXAMINATIONS

FDG-PET examinations are time-critical. FDG-PET examinations immediately after chemotherapy can lead to false positive results. On the other hand, the interval between two chemotherapy cycles should not be prolonged. **FDG-PET examination** should be performed on **day 14 after the last application of chemotherapy**. Therefore the **examination date has to be fixed in advance**.

11 ADVERSE EVENTS (AE/SAE)

11.1 SAFETY MONITORING

For safety reasons, all laboratory parameters relevant for the application of chemotherapeutic drugs should be measured before start of every new cycle of chemotherapy (cf. 6.2.2).
Additionally, blood counts should be measured at least twice during every cycle, especially at time of nadir. The patient's general condition (Lansky resp. Karnofsky index) should be documented before therapy and regularly during therapy.

11.2 ADVERSE EVENT (AE)

11.2.1 Definition

An Adverse Event (AE) is any untoward medical occurrence in a patient or clinical trial subject administered a pharmaceutical or medical product and which does not necessarily have a causal relationship with this treatment. An AE can therefore be any unfavourable and unintended sign (including abnormal laboratory findings for example), symptom, or disease temporally associated with the use of a medicinal (investigational) or medical product, whether or not it is considered to be related to the medicinal (investigational) product. (ICH-Guideline E2A)

Adverse events encompass illness, signs of illness (including pathological laboratory findings) and symptoms that initiate during the trial or previous conditions that become worse.

11.2.2 Documentation and Reporting

Adverse events will be documented on the chemotherapy and radiotherapy toxicity forms. If adverse events occur which are not explicitly named in case report forms, these events have to be named and classified using the severity levels mentioned in section 11.6.1.

11.3 SERIOUS ADVERSE EVENT (SAE)

11.3.1 Definition

A Serious Adverse Event (SAE) is any untoward medical occurrence that at any dose:

- results in death

- is life-threatening

  NOTE: The term "life-threatening" in the definition of "serious" refers to an event in which the patient was at risk of death at the time of the event; it does not refer to an event which hypothetically might have caused death if it were more severe.

- requires in-patient hospitalisation or prolongation of existing hospitalisation
- results in persistent or significant disability/incapacity
- is a congenital anomaly/birth defect or
- other medical important condition (see below)

  NOTE: Medical and scientific judgement should be exercised in deciding whether expedited reporting is appropriate in other situations, such as important medical events that may not be immediately life-threatening or result in death or hospitalisation, but may jeopardise the patient or may require intervention to prevent one of the other outcomes listed in the definition above. These should also usually be considered serious. (see ICH guideline E2A, section IIB)

In case of further events stated as serious by the treating doctors the study chairpersons should be consulted.

Events exclusively related to tumour progression are not stated as SAE.
SAE reporting is restricted to events occurring within 3 months after the end of the study treatment.

11.3.2 Documentation and reporting

The occurrence of ANY serious adverse event (including death, irrespective of the reason) has to be reported by the investigator **immediately (within 24 h)** at latest on the next working day.
Exceptions:
Hospitalisation due to diagnostic procedures or standard supportive care (e.g. implant of central venous catheter) does not constitute an SAE.
In-patient hospitalisation or prolongation of existing hospitalisation due to adverse reactions explicitly covered on the toxicity forms need not be reported as an SAE:

The chemotherapy regimens OEPA, COPP, COPDAC, IEP and ABVD are known to be associated with the following acute adverse reactions:

- Changes in laboratory parameters (haemoglobin, WBC, neutrophils, platelets, creatinine, bilirubin, liver enzymes)

- Fever
- Infection
- Stomatitis / pharyngitis
- Vomiting
- Diarrhoea
- Constipation
- Sensory neuropathy
- Motor neuropathy

These toxicities are documented on the respective chemotherapy toxicity forms.

Radiotherapy is known to be associated with the following acute adverse reactions:

- Changes in laboratory parameters (haemoglobin, WBC, neutrophils, platelets)
- Infection
- Vomiting
- Diarrhoea
- Dysphagia (pharyngeal and oesophagal)
- Mucositis
- Salivary gland toxicity
- Pain caused by radiation
- Skin toxicity
- Fever

These toxicities are documented on the radiotherapy toxicity forms.

These expected adverse reactions are to be reported immediately on the SAE pages only if they

- result in death
- are life-threatening
- result in persistent or significant disability/incapacity

Every SAE must be documented by the investigator on the SAE pages to be found in the investigator site file. The Serious Adverse Event Report Form must be completed, signed and sent to the corresponding study office immediately by fax (see further details in the investigator site file). The initial report shall be promptly followed by detailed, written reports. The initial and follow-up reports shall identify the trial subjects by unique code numbers assigned to the latter. The investigator shall supply the corresponding study office, the national study chairperson, the coordinating investigator and the Ethics Committee(s) with any additionally requested information.

The corresponding study office shall forward the SAE form and all further reports immediately upon receipt to the coordinating investigator (see further details in the investigator site file). The corresponding study office is responsible for the timely provision of all information required by the coordinating investigator for the evaluation of the SAE.

11.4 SUSPECTED UNEXPECTED SERIOUS ADVERSE DRUG REACTIONS (SUSAR)

11.4.1 Definition

Suspected Unexpected Serious Adverse Drug Reactions (SUSARs) are side effects (probably or definitely connected with the administration of the investigational product), the nature or severity of which are inconsistent with the information available about the product. Information about the trial product contained in the SmPC (Summary of medicinal Product Characteristics) should be used to verify if the adverse reaction has been previously described.

11.4.2 Evaluation and reporting

The study coordinator on behalf of the coordinating investigator keeps detailed records of all SAEs reported by the investigators and performs an evaluation with respect to seriousness, causality and expectedness.

The study coordinator submits via the KKSL all information available about a SUSAR immediately, at latest within 15 days after the event becomes known to the sponsor, to the competent authorities and the study offices concerned. In case of death or immediate danger of life caused by a SUSAR the competent authorities and the study offices concerned must be informed within 7 days after the event becomes known to the sponsor. Additional information has to be given within further 8 days.

The involved study offices are responsible for information of the Ethics Committee(s) concerned as well as of the respective investigators. The reporting procedure has to comply with the national legislation.

In addition, other safety issues may also qualify for expedited reporting if they materially alter the current benefit-risk assessment of an investigational product or that would be sufficient to consider changes in the investigational product administration or in the overall conduct of the trial. The study coordinator is responsible for reporting such issues as described above.

The study coordinator on behalf of the coordinating investigator submits yearly or on request a list of all SARs documented, together with an extensive safety report on the investigational products to the concerned competent authorities and Ethics Committee(s) via the national study offices.

The study coordinator on behalf of the coordinating investigator submits every 6 months a list of all SUSARs occurring in the period covered by the report accompanied by a brief report to the concerned competent authorities and Ethics Committee(s) via the national study offices.

11.5 THERAPEUTIC PROCEDURES

AEs requiring therapy must be treated with recognized standards of medical care to protect the health and well being of the subject. Appropriate resuscitation equipment and medicines must be available to ensure the best possible treatment of an emergency situation.

The action taken by the investigator must be documented on the SAE forms according to the following classification:

a) in general	b) on the investigational product
the action taken has to be documented by the investigator in the appropriate section of the CRF and/or by additional documents	• drug withdrawn<br>• dose reduced<br>• dose increased<br>• dose not changed<br>• unknown<br>• not applicable

11.6 CLASSIFICATION OF THE ADVERSE EVENT

11.6.1 Severity

The severity of adverse events will be assessed according to the CTC criteria. Adverse events which are not explicitly listed in the CTC criteria are assessed analogously by the following 5-point system.

Assessment of severity according to *CTCAE V3.0*

a) Adverse event (AE):

**Mild Adverse Event**	• No special medical treatment necessary<br>• All laboratory parameters or x-ray diagnoses without clinical symptoms<br>• Low medical relevance
**Moderate Adverse Event**	• minimal or local medical treatment necessary<br>• exclusively non-invasive treatment (e. g. bandages) necessary
**Severe and undesirable Adverse Event**	• significant symptoms, leading to in-patient treatment or invasive therapy<br>• e. g. transfusions, elective interventional radiology treatment, therapeutic endoscopy or surgery

b) Severe adverse event (SAE):

**Severe and undesirable Adverse Event**	• significant symptoms, leading to in-patient treatment or invasive therapy<br>• e. g. transfusions, elective interventional radiology treatment, therapeutic endoscopy or surgery
**Life-threatening or disabling Adverse Event**	• exacerbated by acute life-threatening complications affecting the metabolism or circulation, e. g. break down of the circulation, hemorrhage, sepsis<br>• life-threatening physiological consequences<br>• necessity of intensive care, immediate invasive, interventional or radiotherapy, therapeutic endoscopy or surgery
**Death related to Adverse Event**	**Death caused by the AE**

11.6.2 Causal relationship

The investigator must judge whether or not, in his opinion, the Adverse Event was connected with the administration of the investigational product according to the classification given below (Venulet and Ham, 1996).

- Possible
- Not possible

Each Adverse Event has to be reported, even if the investigator feels that it is not related to the administration of study drugs.

11.6.3 Expected/Unexpected

An Unexpected AE is an AE, the nature or severity of which is not consistent with the applicable product information (SmPC).

The distinction expected/unexpected only depends on whether the untoward reactions have been previously described.

The following events related to therapy are adverse but expected:

- myelosuppression
- nausea / vomiting
- alopecia
- infections, especially during leukopenic phases
- hemorrhagic cystitis
- cardiomyopathy
- necroses in paravasal injection
- peripheral polyneuropathy, paralytic ileus
- radiation consequences in lung, pericardium or intestine

Expected AEs are also described in 8.7and 9.5.

11.6.4 Outcome

The outcome of an AE has to be classified as follows:

- recovered/resolved
- recovering
- not recovered/not resolved
- recovered/resolved with sequelae
- fatal*
- unknown

*NOTE: A subject's death per se is not an event, but an outcome. The event which resulted into subject's death must be fully documented and reported, even in case the death occurs within four weeks after test drug treatment end, and without respect of being considered treatment-related or not.

12 BIOMETRICAL ASPECTS OF THE STUDY

12.1 OVERALL STUDY DESIGN CONSIDERATIONS

The design of the EuroNet-PHL-C1 study differs from those of standard randomised controlled trials (RCT). It is a therapy titration trial (TTT) with embedded randomised chemotherapy comparison (TG-2+TG-3) (Hasenclever 2003).

In contrast to comparative trials that assess treatment differences, therapy titration trials (TTT) focus on absolute event free survival rates, addressing the question: Is a certain therapy good enough in a particularly selected subgroup of patients?

Under what circumstances does such a question arise?

In Hodgkin's lymphoma

- (Almost) all patients are theoretically curable with established treatment modalities.
- Every patient needs an individual (unknown) amount of therapy to be cured.
- This individual amount of therapy varies considerably.
- Excessive therapy should be avoided due to serious side-effects.

Ideally, every patient should get an optimal amount of therapy based on

- His/her initial disease characteristics (disease burden, stage etc.) and
- an assessment of response to therapy by diagnostic tests

such that he/she is cured with high probability (target cure rate e.g. 90%).

Based on disease burden, patients with Hodgkin's lymphoma are allocated to treatment groups determining the number of chemotherapy cycles required. In addition, early response assessment after 2 OEPA with FDG-PET determines the response group and decides whether radiotherapy is used.

The guiding idea is: All patients (subgroups) should have the same (high) target cure rate with individually minimal therapy.

We know that giving maximum treatment to all patients would result in very high cure rates, but in considerable over-treatment and serious side effects. When we reduce treatment in favourable subgroups the question is whether cure rates remain still satisfactory.

In order for the estimation of an absolute cure rate to be statistically reasonable a representative random sample from a well defined population is needed, not only a

convenience sample as in many RCTs. The assumption of a well defined population is plausible if

- an established study group with many trial sites wants to optimise a collective therapy strategy which functions as treatment guideline for all patients
- patients in each trial site are registered consecutively
- only few patients are treated outside the protocol (high study discipline)

This is given in the GPOH-HD study group. The studies are de facto treatment guidelines in the scope of the GPOH. The high study inclusion rate can be verified by comparison with the German Childhood Cancer Registry. Trial sites may vary in risk profile of their patients. With more than 100 participating clinics these risk profiles are effectively averaged and thus a stable patient population may be expected. (In the German adult Hodgkin study group, which also meets these requirements, in seven out of seven comparisons the same therapy nearly identically reproduced its results in the following study generation (GHSG personal communication and Loeffler et al. 1997). The same applies for the CCLG.

In some subgroups a established standard therapy will be given. As quality control, it is only observed whether the results previously assessed as satisfactory can be maintained. Treatment in other subgroups is modified and/or treatment is reduced or intensified compared to the last study generation.

It would be **nice to have** a randomised comparison to determine the differences that these treatment modifications make. The advantage of randomisation is that the groups to be compared can be assumed identical in structure qua design. Therefore the estimator of the therapy difference is unbiased and a possible effect can be assigned causally to the compared therapies. In TTTs however, the main question is not the therapy difference, but whether therapy results are satisfactory in comparison to a target cure rate. Qualitative statements on efficacy are not intended since they are already assumed evident.

Of course randomisation provides certain additional information and security if enough patients were at disposal. But the problem of sample sizes is rendered critical by the sub-grouping strategy in TTTs. Four fold sample sizes are needed for a study to test (estimate) a difference as compared to a study to test against a target rate or estimate an absolute event rate.

Therefore we will estimate 5 year event free survival rates in those subgroups (adequate response after 2 OEPA) in which treatment is reduced and give 95% confidence intervals in order to assess whether results are compatible with pre-specified target rates. The chosen design maximizes the information gathering in relation to the available case numbers.

Randomised clinical studies remain of course the gold standard for the causal proof of basic efficacy of a new therapy. They are methodologically indicated as soon as a causal assessment of effectiveness to a therapy principle is intended. Therefore patients are randomised concerning the chemotherapy question in TG-2/TG-3 since also a basic statement is intended whether Procarbazine can be replaced by Dacarbazine without loss of efficacy.

12.2 STUDY POPULATIONS

12.2.1 Stratification by method of staging and response assessment (central versus local)

The study population of this inter-group trial will consist of two strata corresponding to how countries organise **s**taging and **r**esponse **a**ssessment (SRA).

Patients from those national study groups that agree to submit all images to real-time **c**entral **s**taging and **r**esponse **a**ssessment (CSRA) in the GPOH-HD study office form the CSRA stratum. This stratum will include patients from countries already participating in the GPOH-HD 2002 Pilot study. The CCLG decided to join the central review process.

Patients from countries that rely on **l**ocal decision making concerning **s**taging and **r**esponse **a**ssessment (LSRA) form the LSRA stratum. Most patients in this group will be entered by the SFCE and PPLLSG.

Both strata will be jointly analysed concerning the randomised chemotherapy comparison.

Whether response based omission of radiotherapy results in sufficiently high 5-year Event Free Survival rates may or may not turn out to be stratum dependent. Therefore both a pooled analysis and separate analyses will be provided.

If a descriptive comparison of the results by SRA stratum showed similar results, this would suggest that success of a strategy for treatment adapted to response (STAR) with FDG-PET does not critically depend on centralised SRA. If a significant difference in this non-randomised comparison were found, the interpretation would have to be very cautious since many country-specific factors might be responsible.

In case of marked differences at an interim safety analysis, a stratum specific discontinuation of the trial will be considered.

12.2.2 Treatment groups

Patients are registered for the study as soon as the diagnosis of Hodgkin's lymphoma is established and informed consent given. After staging the patients are assigned to treatment groups TG-1, TG-2, TG-3. In the CSRA stratum this is documented by central staging, in the LSRA stratum by the local clinician after clearing with the national study coordinator.
Registered patients assigned to treatment groups TG-1, TG-2 or TG-3 form three main study populations for subsequent analyses.

12.2.3 Randomisation groups

Within TG-2 and TG-3, patients in addition will be randomised to receive either COPP or COPDAC chemotherapy after 2 OEPA.
Registered patients who are not willing to be randomised can get standard COPP within this protocol, will be documented and will be informative concerning the STAR question.

12.2.4 Early Response Groups

Very few patients (<5%) will experience progression during 2 OEPA. They only enter the overall analysis by treatment group.

Within each TG, early response will be assessed after 2 OEPA and responding patients assigned to one of the following early response groups:
Adequate response AR:

- AR1: Complete remission based on CT/MRI irrespective of FDG-PET results.
- AR2: At least partial remission and FDG-PET negative (precise definition see 7.3)

Inadequate response IR:

- IR: At least partial remission, but FDG-PET positive (precise definition see 7.3)
- IRu: No change (NC) or at least partial remission and FDG-PET unclear or missing (precise definition see 7.3)

In the CSRA stratum, early response is documented by central response assessment, in the LSRA stratum by the local clinician after clearing with the national study coordinator.

Irrespective of the TG, radiotherapy shall be omitted for AR patients.

12.3 EXPECTED NUMBER OF PATIENTS

Based on the experience of the GPOH-HD 95 and GPOH-HD 2002 Pilot studies and CCLG estimates we expect 250-300 patients per year in the CSRA stratum .

About 150 patinets per year are expected from France and Poland. Additional LSRA patients will be entered from further national study groups.

The EuroNet-PHL-C-1 study shall recruit for 5 years. So it should be possible to include at least 1200 CSRA patients in the first line treatment study. A further 700 patients are expected in the LSRA stratum.

According to previous experience the division into therapy groups TG 1:2:3 is expected as 36:28:36. Proportions in response groups were based on pilot data from GPOH-HD 2002 Pilot.

A randomisation rate of 90% and FDG-PET availability of about 90% were assumed.

The expected sample sizes are detailed in 0. For the relapse study we expect 30-50 patients a year, i.e. a total of approximately 150-250.

Sample sizes for the EuroNet-PHL-C-1 study

			CSRA	LSRA	pooled
total population			1200	700	1900
TG1		0,36	432	252	684
	AR1	0,25	108	63	171
	AR2	0,45	194	113	308
	IRu	0,07	30	18	48
	IR	0,23	99	58	157
TG2		0,28	336	196	532
	AR	0,31	104	61	165
	IR?	0,10	34	20	53
	IR	0,59	198	116	314
	COPP	0,45	151	88	239
	COPDAC	0,45	151	88	239
TG3		0,36	432	252	684
	AR	0,31	134	78	212
	IRu	0,10	43	25	68
	IR	0,59	255	149	404
	COPP	0,45	194	113	308
	COPDAC	0,45	194	113	308
TG2+3			768	448	1216
	AR		238	139	377
	IRu		77	45	122
	IR		453	264	717
	COPP		346	202	547
	COPDAC		346	202	547

Table 21 Expected sample sizes in relevant subgroups.

12.4 BIOMETRY FOR PRIMARY THERAPY: ANALYSIS BY EARLY RESPONSE GROUPS

Data from TG-2 and TG-3 will be pooled in the principal analysis concerning the STAR-question. Secondarily additional separate analyses within TG-2 and TG-3 will be provided.

We do not expect a major difference between COPP and COPDAC based on experience in the GPOH-HD 2002 Pilot study. In the unlikely event of a significant difference the STAR analysis may be restricted to patients receiving the superior regimen.

5-year EFS rates will be estimated and a two-sided 95% confidence interval given for the following early response groups after 2 OEPA:

- TG-1: AR1, AR2, IR, (IRu)
- TG-2+3: AR, IR, (IRu)

In all these early response groups the target 5-year event free survival rate is 90%.

As the main focus is on AR2 in TG-1 and AR in TG-2+3 (as explained below) no multiplicity adjustment is performed.

12.4.1 AR1 in TG-1

In GPOH-HD 95, AR1 patients from TG-1 (complete remission based on CT/MRI irrespective of FDG-PET) had a 97% 5-year event free survival rate. Modifications of diagnostics and therapy are regarded as minor. Main objective of the study in AR1 is quality control, i.e. reproduction of satisfactory results.

12.4.2 AR2 in TG-1 and AR in TG-2+3

The main question of the study concerns AR2 in TG-1 (at least partial remission and FDG-PET negative) and AR in TG-2+3. In these response groups, compared to the previous standard GPOH-HD-95 radiotherapy will be omitted based on negative FDG-PET results. The objective is to demonstrate that therapy results remain nevertheless in the order of a target rate of 90% EFS after 5 years.

12.4.3 IR in TG-1 and TG-2+3

IR patients (at least partial remission, but positive FDG-PET findings), both in TG-1 and TG-2+3, receive an established standard treatment with radiotherapy. As a subgroup IR is negatively selected based on previously not systematically available diagnostics (FDG-PET). If

treatment outcome in IR dropped significantly below the target rate of 90% 5-year EFS-rate, therapy intensification in this group would be indicated in the next study generation.

12.4.4 IRu

This hopefully small response group is given standard treatment with radiotherapy. Main interest in subgroup IRu is its size since it comprises patients in whom either FDG-PET was not available or was inconclusive due to technical problems.

12.4.5 Power considerations

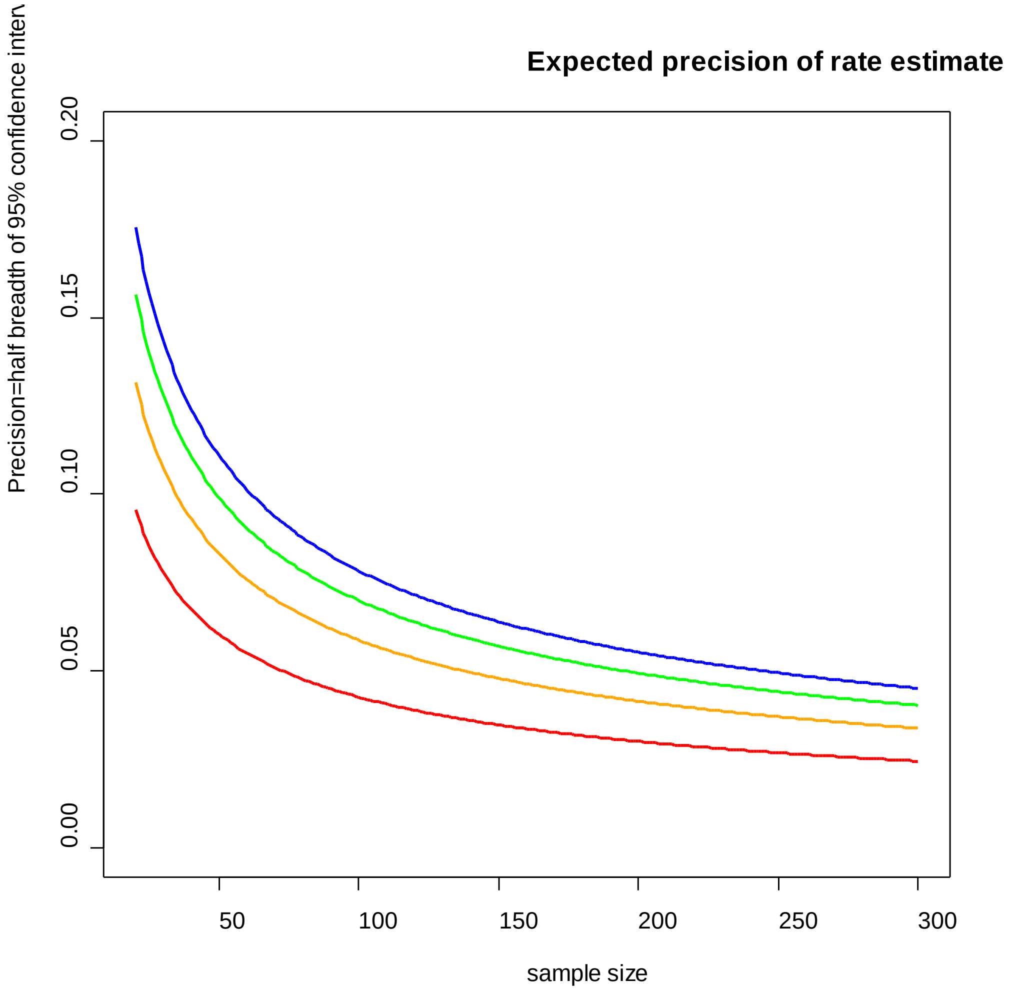

Fig. 12. Expected precision of rate estimates

Expected precision (half width of 95% confidence interval) of estimate for rate estimations as a function of sample size and actual rate: 95%: red, 90%: orange, 85%: green, 80%: blue.

Fig. 12 together with table 20 illustrates the achievable precision of estimation for different sub-groups and plateaus.

Both in TG-1 AR2 and TG-2+3 AR assuming about 200 patients and cure rates of 90%, the expected half width of the 95% confidence interval is less than 5%. In terms of a one-sided test against the target cure rate 90% this corresponds to a power of more than 80% to detect a difference of 6%.

12.5 BIOMETRY FOR FIRST LINE THERAPY: RANDOMISED COMPARISON OF CHEMOTHERAPIES IN TG-2 AND TG-3

In TG-2 and TG-3 randomisation is performed according to the following scheme

2 OEPA + 2 resp. 4 COPP ± RT (depending on response to 2 OEPA)
versus
2 OEPA + 2 resp. 4 COPDAC ± RT (depending on response to 2 OEPA)

In CSRA countries, randomisation is performed after the staging tumour conference which determines the definite treatment group. Therapy with OEPA, which is equal in all three treatment groups, normally has already begun at this time point. Progressions during OEPA are extremely rare.

In order to avoid bias, decisions on response adaptation and radiotherapy planning (boost on slowly responding regions) are based on the restaging data after 2 OEPA. For CSRA, members of the central tumour conference will be blinded to the assigned chemotherapy arm.

12.5.1 Randomisation algorithm

Randomisation of patients into both chemotherapy arms is performed centrally with a modified minimisation procedure with stochastic component according to Pocock (1983) with a 1:1 proportion.

To avoid imbalances, the randomisation will be stratified according to the following criteria:

- TG-2 versus TG-3
- Countries
- trial site
- girls versus boys

The clinical stratification factors have priority in the given order.

12.5.2 Statistic formulation (TG-2 + TG-3)

Aim of the study in TG-2 + TG-3 is to estimate the difference in EFS

- 2 OEPA + 2 and 4 COPP ± RT respectively and
- 2 OEPA + 2 and 4 COPDAC ± RT respectively.

The difference is primarily quantified as log hazard ratio. To estimate it a proportional hazard model with the factors treatment group (TG-2 versus TG-3), sex, SRA stratum and therapy (COPP versus COP-DAC) is fitted. A two-sided 95% confidence interval is given for the treatment effect.

Secondarily this difference (and its confidence interval) is represented as a difference in 5-year EFS rates. Depending on acceptance of randomisation 700 to 1100 patients are expected for this comparison.

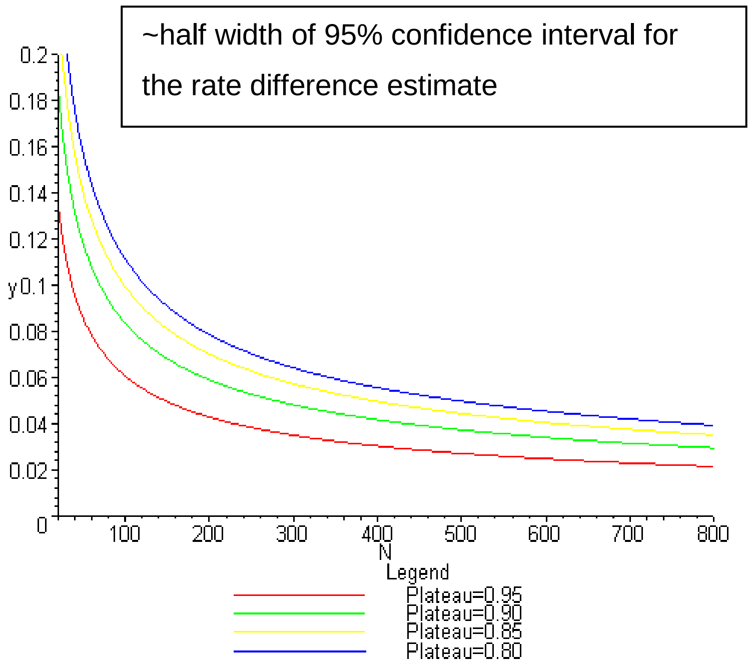

Fig. 13. Expected accuracy of estimate for the estimation of rate differences
Half width of 95% confidence interval) as function of total number of cases and the actual average rate.

The diagram (Fig. 13) illustrates approximately the expected half width of the 95% confidence interval for the difference in 5 year EFS rates between the arms as a function of the total sample size. With a plateau of 90% EFS and no difference in the arms the expected half width of the 95% confidence interval is approximately 4%. The power of such estimating accuracy is illustrated by the following scenarios:

scenario 1: (inferiority of COPDAC)
Assuming EFS of about 90% with COPP and an underlying true difference of 8%, we have 80-90% power to exclude the value 0 from the 95% confidence interval for the difference.

scenario 2: (COPDAC maximal 2% worse than COPP)
Assuming EFS of about 90% with COPP and an underlying true difference of 0-2% the power is 80-90% to exclude a difference of 8-9% from the 95% confidence interval.

Analysis follows the intent-to-treat-principle. If a relevant number of patients should swich arms an additional "as-treated" analysis is performed, since intent-to-treat-analyses can be anti-conservative in equivalence questions. In case of discrepancies a sensitivity analysis will be performed.

A subgroup analysis according to treatment group (TG-2 versus TG-3) can be carried out by inserting a respective interaction term into the Cox model described above. We expect, if at all, at most a moderate interaction (in terms of moderately stronger differences in TG-3). A qualitative interaction is not expected.

12.6 BIOMETRY FOR RELAPSE STUDY

The relapse study is a quality control treatment optimisation study. Therapy results and toxicity of relapse therapies are described for the subgroups resulting from the general treatment strategy.

The statistical aim of the relapse study is the estimation of 5 year EFS rates in the following patient populations:

- All second line patients
- Patients with late relapse after TG-1 therapy. Here an outcome consistent with a target cure rate of 90% is expected based on previous study results.
- Patients with progression during or after first line treatment. With standard salvage treatment these patients had a 5-year EFS rate of about 40%. High dose chemotherapy is introduced to improve this unsatisfactory outcome.
- Patients with early relapse or late relapse after TG-2 or TG-3 therapy. With standard salvage treatment these patients had a 5-year EFS rate of about 60-70%.
    - Patients with early relapse or late relapse after TG-2 or TG-3 therapy with **adequate early response (AR)**. Objective of the trial is to demonstrate that

outcome in this positively selected group is consistent with a target cure rate of 90%.

- o Patients with early relapse or late relapse after TG-2 or TG-3 therapy with **inadequate early response (IR)**.

12.7 ADDITIONAL QUESTIONS

12.7.1 Acute toxicity comparison COPP versus COPDAC

The acute toxicity profiles are comparatively described on the basis of CTC criteria.

12.7.2 Fertility assessment

Elimination of the very effective drug Procarbazine from chemotherapy in TG-2+3 is motivated by the hope that with COPDAC male fertility is preserved in a relevant proportion of boys and premature menopauses are avoided in girls.

Fertility endpoints are notoriously problematic from a biometric point of view. The most relevant endpoint would be the number of children born or fathered. We will try to organise long term follow-up (see 12.7.3) and to collect these data, but as most children will be born after the formal end of this study the number of children cannot be an endpoint.

12.7.2.1 Fertility assessment in boys

In boys, the second best endpoint is based on semen analysis in sexually mature patients performed at least 12-18 months from chemotherapy (since temporary azoospermia after chemotherapy is documented).

We will systematically remind the responsible physicians to consider and propose **optional** semen analysis once patients are sexually mature (Tanner stage >3) and are in the study for 2 years.

We doubt that it would be feasible to perform an obligatory semen analysis in all treated boys and expect a substantial proportion of missing values.

- Boys under the age of say 10 years at diagnosis may not be through puberty within the 5 year study period.
- Boys need to give additional informed consent at the time semen analysis is proposed.

- The responsible clinician may be reluctant to address the question of infertility in certain cases.

Semen analysis may turn out to be preferentially performed in patients with prior abnormal biochemistry. Thus the comparison of the rates of azoospermia after COPP resp. COPDAC in patients who had a semen analysis may turn out to be conservatively biased if more frequent analyses are performed in the more gonadotoxic arm.

In addition, several laboratory parameters LU, FSH, Inhibin B, testosterone will be collected with the respective local reference ranges. In these parameters a reasonable degree of data completeness is expected. It also remains to be seen whether these parameters are sufficiently standardised to allow pooling of data in a Europe-wide study. It is logistically not feasible to measure these values in a reference laboratory.

It should be noted that these parameters, although widely used in studies, are not formally established biometrical surrogate endpoints: Their correlation with the results of semen analysis and actual fertility is only moderate and it is not clearly documented that differences in biochemistry regularly translate into differences in fertility.

Considering all these biometrical difficulties, the following compromise endpoint is proposed for comparative purposes of this protocol only :

Evidence of male infertility score (EMI-score):

Missing =	no fertility assessment after treatment
0 =	Normospermia documented **or**<br>Biochemistry (LU, FSH, Inhibin B, testosterone) results consistent with normal gonodal function
1 =	Abnormal biochemistry by local standards (LU, FSH, Inhibin B, testosterone) **without** documented Normospermia and/or<br>Oligozoospermia (Low spermatozoa concentration) and/or<br>Asthenozoospermia (Low spermatozoal motility) and/or<br>Teratozoospermia (Low normal morphology)
2 =	Azoospermia (No spermatozoa)

In case of multiple fertility assessments the latest biochemistry or sperm analysis results will be used. Possibly conflicting data will be reviewed by an expert without knowledge of treatment received.

The EMI-Score will be used to assess differential effects of chemotherapy on male fertility. Note that the EMI-score is neither appropriate nor intended to be used for describing fertility status or impact of treatment on fertility status in absolute terms.

A proportional odds model including treatment (COPP vs. COPDAC), Radiotherapy (y/n), treatment group (TG-2 vs. TG-3) and puberty status at diagnosis will be fitted to the data of boys randomised between COPP and COPDAC chemotherapy.

With 2 OEPA + 2(-4) COPP we expect that pubertal development will be normal or only slightly delayed and that about 50% of the boys will show abnormal biochemistry (Gerres at al 1998). Data on semen analysis are available only in rare selected cases with abnormal biochemistry and show a high azoospermia rate. With the envisaged sample sizes in TG2+TG3, we will have sufficient power to detect any clinically meaningful reduction in the EMI-Score.

12.7.2.2 Fertility assessment in girls

We expect that pubertal development will be normal or only slightly delayed in girls after treatment. Ovarian failure was not observed without pelvic irradiation in the GPOH/DAL experience (Schellong et al. 1996).

In adult women, substantial rates of secondary amenorrhea after chemotherapy have been reported (Behringer 2006) which increased with age at diagnosis and amount of chemotherapy. Toxic effects of chemotherapy are expected to reduce the ovarian reserve and may thus lead to a premature menopause.

The most relevant long-term endpoint “time to menopause” is not observable within the formal five year study-period and may only be addressed in long-term follow-up. Ovarian volume has been proposed as a predictor of time of menopause based on a mathematical model (Wallace at al 2004, 2005), but its use – in particular in young girls - has not been validated so far.

Thus fertility assessment in girls will be mainly descriptive and explorative: Data on the menstrual cycle, biochemistry in girls not taking the pill or under hormone replacement treatment, and - if possible - trans-vaginal measurements of ovarian volume will be collected.

The following Female fertility score (FFS) will be used as a summary endpoint:

Missing = no assessment after chemotherapy

0 = regular menses (consistent with contraceptive use of pill)

1 = abnormal biochemistry and/or
irregular menses and/or
hormone replacement treatment
2 = no menses for at least one year

In case of multiple fertility assessments the latest results will be used.

We will compare the FSF-Score in girls randomised to COPP or COPDAC chemotherapy (as above for the boys), but do not expect major differences since premature menopauses will probably not be observable during the study.

12.7.3 Late toxicities

Study patients should be followed-up concerning late toxicities for as long as possible also beyond the time of final analysis. Therefore, in Germany a co-operation with the German Childhood Cancer Registry is intended.

12.7.4 Comparison of CSRA and LSRA

Results with and without central staging and response assessment will be described and exploratively compared.

12.8 END POINTS OF STUDIES

12.8.1 Primary endpoint

Primary endpoint is event free survival (EFS):

EFS:= time from registration until the first of the following events:

- progression/relapse of disease
- occurrence of a secondary malignancy
- death by any cause

12.8.2 Secondary endpoints

- Overall survival (OS)
  OS:= time period from registration until death of any cause

- Progression free survival (PFS):
  PFS:= time period from registration until the first of the following events:
  - o progression/relapse of disease
  - o death of any cause

- Acute toxicity documented by CTC criteria
- Evidence of male infertility score (EMI-score)
- Female sexual functioning score

- Long-term data (offspring, premature menopause, secondary cancer etc.)

12.9 INTERIM ANALYSES AND STOPPING CRITERIA

12.9.1 Early assessment of randomisation feasiblity

After one year of accrual the rate of successful randomisation will be assessed. If it turns out to unsatisfactory adequate organisational measures or protocol amendments will be proposed.

12.9.2 Stopping treatment arms with response adaptation

Not the number of patients is relevant for the question of early termination, but the number of relapses observed during the recruitment period of the study. Since relapses are fortunately rare and furthermore typically occur with a delay of 0.5-5 years it is improbable that enough relapses for a decision on termination occur in any of the groups until completion of recruitment.

Concerning STAR, in the treatment response groups IR and IRu (all TGs) as well as in AR1 in TG-1 a well-established standard treatment is given. No stopping rules are required in these subgroups.

In AR2 in TG-1 and AR in TG-2 + TG-3 for protection from unexpected catastrophe scenarios starting after the second year of recruitment annual interim analyses are planned if at least 40 patients have a median observation time of at least 18 months. The evolving EFS curves will be tested against a EFS reference curve adjusted to a target 5-year EFS of 90% based on the known hazard function in Hodgkin's lymphoma (one sample test assuming reference curve and proportional hazards). These tests are performed on the 0.1% significance level, the final analysis will thus remain unaffected (Peto-sequential testing scheme).

If one of these tests is significant the Data Monitoring Committee is informed immediately. After the vote of the Data Monitoring Committee the Inter-Group Trial Steering Committee decides on the continuation of the study in the relevant group appreciating all concomitant circumstances.

12.9.3 Stopping the randomised chemotherapy comparison

Starting after the second year of recruitment annual intermediate analyses are intended if at least 80 randomised patients have a median observation time of at least 18 months. A proportional hazard model with the factors treatment group (TG-2 versus TG-3), SRA stratum and therapy (COPP versus COPDAC) is fitted. Tests of the treatment effect are carried out on the 0.1% level, the final analysis will remain unaffected by this (Peto-sequential testing scheme). According to data of the pilot study with COPDAC dramatic differences are not to be expected.

If one of these tests is significant the Data Monitoring Committee is informed immediately. After its vote the Inter-Group Trial Steering Committee decides on the continuation of the study in TG-2/TG-3 appreciating all concomitant circumstances.

12.9.4 Termination of arms of the relapse study

Starting after the second year of recruitment annual descriptive interim analyses are planned. Due to the relatively small case numbers and unclear comparison standards a formal termination strategy is not provided (cp. however 13.6.2).

12.10 STATISTICAL ANALYSES

If not yet specified above the following statistical methods are applied:

Time-to-event end points are described with the Kaplan-Meier estimator and 5-year survival rates are stated with 95% confidence interval. Regression analyses are performed with the proportional hazard model.

Qualitative variables are described by frequency tables (with 95% confidence interval where relevant) and compared using contingency tables (Chi^2-test if relevant).

Metric variables are described by adequate measures of location (average, median) and of spread (standard deviation, inter-quartile range). Histograms or box-plots are used for graphic representation. For comparisons adequately chosen difference measures with 95% confidence interval can be estimated and t-test or Mann-Whitney-U-test can be used respectively.

The following lists are compiled:

- Registered, but excluded patients.
- Arm switches / major protocol deviations
- Patients with SAEs.
- Patients with treatment failure.
- Deceased patients with cause of death.

A detailed analysis plan is developed on the occasion of the first interim analysis. The final evaluation concerning EFS is performed as soon as at least 80% of patients have more than five years of follow-up.

13 REFERENCE EVALUATION

13.1 CENTRAL STAGING AND RESPONSE ASSESSMENT

For those groups not using central review (e.g. SFCE, PPLLSG), the determination of the stage is the responsibility of the local investigator after discussion if necessary with the national study chairperson or his/her deputy. Stage will be checked by the corresponding study office based on information provided on the staging form.

13.1.1 Determination of reference stage

The reference stage is defined for every patient by the central review panel during tumour conferences taking place regularly (at least weekly) at the GPOH-HD study office. The following information and material is required for the central staging and response assessment, and has to be provided by the treating investigator:

- Results of clinical examination (including ENT findings)
- Results of sonography
- Results, images (preferably electronically) of all CT / MRI examinations and FDG-PET data sets

The relapse reference stage is determined by the same procedure.

13.1.2 Organisation of the tumour conference

13.1.2.1 Preparations before tumour conference

Participating trial sites send the necessary written results and films or data sets (except: PET images – these are transmitted directly by the local nuclear medicine physician) to the GPOH-HD study office (see procedure manual and checklists in the investigator site file).

The study secretary and her authorised assistant check the documents for completeness. If necessary documents are missing, a reminder telephone call is made immediately. As soon as all necessary documents are available the patient is scheduled by the study secretary in the weekly tumour conference.

Prior to the tumour conference both the reference radiologist and the reference nuclear medicine physician independently evaluate the submitted images or data sets (being aware of the written reports of the local centre).

13.1.2.2 Organisation of FDG-PET examinations

The cooperation of the nuclear medicine centres in Germany (list of available centres is provided in the FDG-PET manual) with the reference centre is regulated by a cooperation agreement which includes the following commitments:

- FDG-PET examinations are performed according to study protocol for study patients of the own trial sites and the trial sites of the surrounding regions at the adequate time point according to the protocol
- a person in charge of the organisation (making appointments, transmitting data) and two experienced doctors for evaluation of PET results are appointed
- measuring data are sent to the reference nuclear medicine physician in the GPOH-HD study office within one working day via internet and the report on diagnostic results is transmitted to the investigator **at the latest** on the following day

For complete and timely data acquisition the following has to be ensured:

- The nuclear medicine reference centre gets preliminary information on place and time of arranged PET examinations based on the data of patient registration and the automatic default of an optimal date by the checklists.
- The electronic data transmission of measured data from the respective PET facility to the PET reference centre is carried out on the day of examination.
- The dispatch of the finding by the local PET centre to the investigator and the reference nuclear physician is also performed electronically or by fax at the latest on the following day.
- If necessary the reference nuclear medicine physician reminds the local nuclear medicine physician of missing data.
- The nuclear medicine physician and the paediatric oncologist of the trial site are responsible for the instigation and initial assessment of skeletal scintigraphy in case of positive PET results in the skeletal system.
- In preparation of the tumour conference PET findings are evaluated by the reference nuclear medicine physician or his deputy. If discrepancies with the local report occur they are discussed with the contact persons appointed by the cooperating PET centres.

13.1.2.3 Tumour conference

Participants of the tumour conference are: the study chairperson, the study secretary, the reference radiologist, the reference nuclear medicine physician and the reference radiotherapist or the respective deputies.

In the tumour conference as a first step the clinical findings and the findings collected in sonography are presented by the study secretary. Next the reference radiologist reports and compares the results based on his CT/MRI evaluation. Then these data are related to the PET results by the reference nuclear medicine physician. Finally the reference stage is determined in consultation with the study chairpersons or his deputy considering all results applying the rules of chapter 6.4.

13.1.2.4 Transmission of information to the investigators

The result of the tumour conference is noted on the staging / response assessment forms – tumour conference. Subsequent to the tumour conference these forms are sent to the investigators by fax and/or letter within maximal two working days with corresponding annotations by the study secretary after consultation of the study chairperson.

The GPOH-HD study office is responsible for establishing the reference stage after final evaluation of the PET / CT / MRI examination findings in synopsis with clinical findings. Also the assignment to therapy groups as well as the PET oriented therapy stratification in TG-1 and in relapse therapy is performed by the GPOH-HD study office.

13.2 REFERENCE RADIATION PLANNING

13.2.1 Tasks of the reference radiotherapist (central review)

For all study patients a reference radiation plan is designed by the reference radiotherapist or their deputy. This plan considers the involvement pattern, for relapse patients also the preceding radiotherapy, the response to treatment and also the vicinity to risk organs (see chapter 8.6). The reference radiotherapist or their deputy are also at the study participant‘s disposal for concrete questions regarding the patient’s radiation planning and performance.

Within the scope of primary therapy the preliminary reference radiotherapy plan is developed subsequent to the staging tumour conference after defining the reference stage and transmitted to the treating investigators together with the results of the first tumour conference for the respective patient. This should prevent a delay in the start of planning.

The definite radiotherapy plan (with additional information on boost regions and also lung radiation if necessary) is defined within the scope of the first restaging tumour conference (after

two cycles of chemotherapy). Also this plan, which is signed by the reference radiotherapist and the study chairperson, is transmitted to the treating investigators together with the result of the restaging tumour conference.
In the framework of relapse therapy the definite radiotherapy plan is developed according to response and therapy group after the relevant restaging.

13.2.2 Working group for quality control in radiotherapy

A radiotherapy quality assurance working group will be formed. This working group will provide consultation for treating investigators.
In addition, radiation planning will be checked for a certain amount of patients (e.g. 10%) in primary therapy, which are randomly chosen, and for all relapse patients. The respective documents are requested by the working group directly from the treating radiotherapist. If in individual cases a severe protocol deviation is detected, the according radiotherapeutic facility will be advised in detail.

13.2.3 Documentation of radiogenic late effects

Due to the legislation in Germany radiotherapists are obliged to do annual checkups of irradiated patients concerning the development of late effects within a period of 5 years. The data obtained in this are collected in the registry for radiogenic late effects (chairperson: Prof. Dr. Willich, Universitätsklinik Münster). Therefore the registry gets the information from the GPOH-HD study office at the time of restaging which patient is irradiated in which clinic at what point of time. The registry for radiogenic late effects then contacts the treating clinic to get the according information from the treating radiotherapist.

13.3 REFERENCE PATHOLOGY

Histologic diagnosis confirmation is an indispensable basic requirement for admission of a patient to the study. The participating reference pathology centres as well as the diagnostic criteria and markers are described within the **reference pathology manual**.

14 SCIENTIFIC AND BIOLOGICAL ADD ON STUDIES

It is planned to collect tumour material and serum from patients for scientific and biological add on studies. Side projects need approval by the scientific advisory board. A separate protocol and respective informed consent form will be developed and submitted to the relevant ethics committees.

Risk of long-term infertility (beyond five years of follow-up) in males and premature menopause in females in particular comparing COPP and COPDAC will be addressed by a side project. It is planned to set up a European late effects registry which will also collect this data.

15 ETHICAL AND LEGAL ASPECTS AND AGREEMENTS

All persons participating in the conduct of the trial commit themselves to observe the Declaration of Helsinki and all its revisions (incl. the Edinburgh Amendment from October 2000), as well as all pertinent national laws and the ICH guidelines for Good Clinical Practice (GCP) issued in June 1996 and CPMP/ICH/135/95 from September 1997, taking into account the Directive 2001/20/EC of the European Parliament and of the Council of 4 April 2001.

15.1 SPONSORSHIP

The Martin Luther University of Halle/Wittenberg is sponsor of the international clinical trial EuroNet-PHL-C1 in the legal sense as defined by the Directive 2001/20/EC of the European Parliament and of the Council of 4 April 2001. The sponsor internally transfers his duties for every participating country to an authorised institution (as nominated by the national study group and / or the national study chairperson) by written agreement.

The authorised institution will fulfil the transferred duties for the sponsor and warrants the compliance with all the statutory provisions relevant for the sponsor. The authorised institution is responsible for pointing out to the sponsor those duties required by a respective national regulation that were not transferred to the authorised institution by the written agreement and has to support the sponsor in fulfilling these duties as required.

Beginning with the date of the agreement the authorised institution will semi-annually draw up a written report on the progress of the clinical trial in the respective country, the fulfilment of the transferred duties and the compliance with legal requirements. In addition the authorised institution will on demand provide all documents (if applicable as copy) necessary for the Trial Master File of the sponsor.

The sponsor reserves the right to audit the authorised institution to control adherence to all legal requirements.

15.2 REQUEST OF AUTHORISATION TO THE COMPETENT AUTHORITIES

The coordinating investigator is responsible for the request of authorisation to the competent authority in Germany, taking into account the German laws. Furthermore, the coordinating investigator will provide all authorised institutions of the participating countries with all documents necessary for request of authorisation according to German law.

The authorised institutions of the participating countries are responsible for provision of all further documents required by national law and for request of authorisation to the competent authority in the respective country, taking into account the national laws.

15.3 APPLICATION FOR NATIONAL ETHICS COMMITTEE OPINIONS

The coordinating investigator on behalf of the sponsor is responsible for the application for an Ethics Committee opinion in Germany, taking into account the German laws. Furthermore, the coordinating investigator will provide all authorised institutions of the participating countries with all documents required for application for an Ethics Committee opinion according to German law.

The authorised institutions of the participating countries are responsible for provision of all further documents required by national law and for application for an Ethics Committee opinion in the respective country, taking into account the national laws.

15.4 NOTIFICATION OF SUBSTANTIAL AMENDMENTS

Any protocol amendments prepared by the Inter-Group Trial Steering Committee, which are likely to have an impact on the safety of the trial subjects or to change the interpretation of the scientific documents in support of the conduct of the trial, or which are otherwise significant, require an authorisation of the competent authorities of the participating countries, as well as approval of the concerned Ethics Committees.

The coordinating investigator on behalf of the sponsor is responsible for the request of authorisation to the competent authority in Germany as well as for application for an Ethics Committee opinion in Germany, taking into account the German laws. Furthermore, the coordinating investigator will provide all authorised institutions of the participating countries with all documents for request of authorisation respectively for application for an Ethics Committee opinion according to German law.

The authorised institutions of the participating countries are responsible for provision of all further documents required by national law and for request of authorisation to the competent authority as well as for application for an Ethics Committee opinion in the respective country, taking into account the national laws.

15.5 DECLARATION OF THE END OF TRIAL

The coordinating investigator on behalf of the sponsor is responsible for providing all documents required for the notification of the regular or premature termination of the trial. The coordinating investigator is responsible for notification of the end of trial to the competent authority and the Ethics Committee concerned in Germany, according to German laws.

The authorised institutions of the participating countries are responsible for notification of the end of trial to the competent authority and the ethics Committee concerned in the respective country, taking into account the national laws.

15.6 INSURANCE

In Germany, patients are insured by the insurance company Gerling Vertrieb Deutschland GmbH (Theodor-Heuss-Allee 108, 60486 Frankfurt, Tel 0049-69-7567-466). The number of the certificate of insurance is: 70-005890284-7. A copy of the certificate of insurance and the insurance conditions will be filed in the investigator site file. Copies of both documents should be handed over to the patients and/or their parents or guardian.

The authorised institutions of all other participating countries are responsible for the provision of insurance or indemnity to cover the liability of the investigator and the sponsor in the respective country (if necessary according to national law), as required by the Directive 2001/20/EC of the European Parliament and of the Council of 4 April 2001 and the corresponding national laws.

15.7 FURTHER AGREEMENTS

Procedures and agreements for safety management are described in chapter 11, for archiving in section 16.3, for selection of qualified investigators and trial sites in section 4.2.

16 DATA HANDLING AND RECORD KEEPING

16.1 CASE REPORT FORMS (CRF)

The CRF will be prepared by the KKSL and printed on 3-part, no carbon required (NCR) paper. The regional study offices receive the original and the first copy (yellow) of the CRF pages and the second copy (blue) is retained in the trial site.

Case report forms (CRFs) should always be completed with a black ballpoint pen. Pens or pencils cannot be used. Attention has to be paid to a clear, legible script, preferably block letters. Mistakes in completion of CRFs are cancelled with a simple horizontal line and correction is written above or next to it. All corrections have to be clearly signed and dated. The use of correction fluid is not permitted. Data fields which cannot be completed due to missing information have to be annotated. The forms have to be completed in a timely manner and finally checked and signed with date by the investigator.

The signatures serve to attest that the information contained in the CRF is true and has not been falsified. In case of a major correction or missing data, the reason for it shall also be given. The investigator must assure completion, review and approval of all CRFs. At all times the principal investigator has final responsibility for the accuracy and authenticity of all clinical and laboratory data entered in the CRF. Even if there are no changes from a previous examination the questions which are repeated in each section of the case report forms should be answered completely.

The carefully completed case report forms (original and first copy (yellow)) are sent to the regional study office at the scheduled time points.

As source data are regarded:

- all data contained in the patient’s medical record
- all data provided by the reference pathology
- all data provided by the tumour conference (in countries were a centralized staging and response assessment procedure is installed)

Further information on data collection and CRFs is given in specific **handling guidelines**, which are stored in the investigator site file.

16.2 DATA MANAGEMENT

Each participating study group has to specify a regional study office. Data management will be performed by the regional study offices according to common standard operating procedures (SOPs).

The study management software Oracle Forms will be used for creation of the study database. The database will be validated according to the corresponding SOPs of the KKSL prior to data capture. The regional study offices will be linked through internet to this common data base. The communication will be realized using a standard web protocol (https), in most cases no configuration of firewalls will be necessary. The local installation will be supported by a detailed to-do-list and email/telephone support.

The responsibilities of the regional study offices include:

- Handling of registrations from its participating local trial sites
- Shipment of investigator site file, CRFs etc. to local trial sites
- Data entry
- Collection of paper CRFs including reminder-campaigns
  (Reminder lists will be generated automatically from the database)
- Clinical check of CRFs and queries on missing or implausible data
  (Queries will be generated automatically from the database)
- DB access to own data (views, reports)

An audit trail of all changes concerning the contents of the study database will be automatically recorded.

Once the database has been declared complete and accurate, the database will be locked. Thereafter, any changes to the database are possible only by joint written agreement between the coordinating investigator, the biometrician and the data manager.

16.3 ARCHIVING

The archiving of all study relevant documents at the trial site, at the regional study offices and at the coordinating investigator will be performed according to the requirements of ICH-GCP, the Commission directive 2005/28/EC of 8 April 2005, and the pertinent national laws.
In countries were a central staging and response assessment procedure is installed, all CT/MRI/PET images sent to the study office will be archived electronically by the responsible reference radiologist and the responsible reference nuclear medicine physician.

17 QUALITY CONTROL AND QUALITY ASSURANCE

17.1 DIRECT ACCESS TO SOURCE DATA

According to ICH-GCP and to the applicable European laws, the principal investigator must permit all authorised third parties access to the trial site and insight into the medical records of the trial subjects (source data). This permission includes the clinical trial monitors, auditors and other authorised employees of the sponsor, as well as members of the competent authorities. All these persons are sworn to secrecy.

17.2 MONITORING

The authorised institutions (cf. chapter 15) are responsible for the organisation of an adequate monitoring process in the respective country.

In general, only a random on-site monitoring is provided in the context of this study. Nevertheless, in case of frequent protocol violations, incomplete documentation, unanswered queries or other problems, for cause monitoring visits may be performed.

The main emphasis of on-site monitoring should be a check of the informed consent forms and of the inclusion and exclusion criteria, as well as on the main efficacy and safety endpoints. In addition on-site monitoring visits make sure that the study is performed according to ICH-GCP, and that the protocol is adhered to. Thus, on-site monitoring plays an important role in the support and training of participating trial sites.

Details of the country specific monitoring procedures will be provided within a **monitoring manual**.

17.3 AUDITS

In order to guarantee that the conduct of the study is in accordance with ICH-GCP and the national laws, the sponsor or his legal representatives reserves the right to audit selected trial sites. The auditor will be independent from the staff involved in the proceedings of this clinical study.

17.4 INSPECTIONS

According to the pertinent European legislation, inspections of the trial sites may be performed by the competent authorities at any time during or after completion of the trial.

18 DATA PROTECTION

Within this study personal data of the trial subjects (initials, date of birth) and data regarding the therapy and the course of disease will be collected.

If the patients and their parents or guardian have consented, the name and address of the patient are stored in the study office, in order to allow for direct contact in case of overdue follow-up information. Name and address of the trial subjects will be stored apart from the database, and will be handled strictly confidentially.

All data will be stored electronically and handled strictly confidentially. Throughout documentation and analysis, subjects will be identified only by the individual patient code, whereas all subject names will be kept secret by the investigator.

In countries where a central staging and response assessment is installed, the members of the reference team and their authorised assistants will have access to the patients' full name. All these persons are sworn to secrecy.

The investigators are obliged to keep all study data and information confidential and to use those data only in context with the persons involved in the trial conduct. Study material or information generated in this trial must not be made available to third parties, except for official representatives of the sponsor or regulatory authorities.

Data will be processed in the KKSL, according to the written safety concept of this institution. Access to the data will be strictly limited to authorised persons. Extensive back-up procedures are installed to prevent loss of data. All legal requirements concerning data protection and confidentiality will be respected. All authorised persons are sworn to secrecy.

All data are completely backed up every day. By applying a hierarchic role-based access method unauthorised access to patient data is excluded as far as possible. Anonymity of data in the scope of statistical analysis is ensured.

18.1 CONFIRMATION OF DATA PROTECTION

During data entry, handling and analysis in the KKSL all requirements of the data protection act will be taken into account. Access to the data is strictly limited to authorized persons. Data are protected against unauthorised access.

19 ADMINISTRATIVE AGREEMENTS

19.1 ADHERENCE TO THE PROTOCOL

Protocol violations are any deviations from the procedures outlined in this document:

- missed evaluations/ incorrect timing of evaluations
- non-compliance with study medications/ intake of prohibited medications

After a patient has been enrolled, it is the investigator's responsibility to make a reasonable effort to avoid any protocol violation in order to keep the subject in the study.

Major protocol violations will be reported immediately to the corresponding study chairperson and the coordinating investigator during the course of the study. All protocol violations will be listed and discussed with the coordinating investigator and the biometrician prior to statistical analysis.

The investigator makes every effort to record data according to the protocol. Under practical working conditions, however, some minor variations may occur due to circumstances beyond the control of the investigator. All such deviations will be documented in the records, together with the reason for their occurrence, and where appropriate, detailed in the study report.

19.2 PROTOCOL AMENDMENTS

In order to ensure most comparable conditions during all stages of the trial and in the interests of valid statistical analysis, the investigators, the coordinating investigator or any other person involved in the trial conduct may not alter the study conditions agreed upon and set out in this protocol.

Amendments should be made only in exceptional cases and by mutual agreement within the Inter-Group Trial Steering Committee. Any amendment and the reasons therefore must be set out in writing, and signed by all parties concerned. The amendment then becomes part of the study protocol, and is to be filed in the Trial Master File (TMF).

The Inter-Group Trial Steering Committee also decides when such protocol changes become effective. If protocol changes are profound, i.e. if they regard modifications of a therapy arm, inclusion or exclusion criteria, period or the closing of a therapy arm or of the whole study the vote of the DMC must be obtained as a matter of principle.

Amendments which might have an impact on the well-being of the trial subject (substantial amendments) such as the use of additional invasive diagnostic procedures require an additional approval by the Ethics Committees (EC) and by the competent authorities

concerned. In addition, a further informed consent form is to be signed by all trial subjects already enrolled in the trial who might be affected by the amendment. Minor changes will only be submitted to the Ethics Committees and the competent authorities in a written form (for further details see section 15.4).

The investigator may implement a deviation from, or a change of the protocol to eliminate an immediate hazard(s) to trial subjects without prior EC approval. As soon as possible, the implemented deviation or change, the reason for it, and if appropriate, the proposed protocol amendment(s) should be submitted to the coordinating investigator for agreement.

19.3 PUBLICATION POLICY

Participating centres may if they wish publish their own cases but will agree to allow the clinical board of EuroNet exclusive right to publish the major results of the EuroNet-PHL-C1 study (primary, secondary and tertiary study questions as detailed in the protocol) in part or in total.

All such publications will acknowledge the contribution of the participating clinicians. Authorship of such publications, including abstracts, will represent members of the trial steering committee, members of the central review panel and other persons who made a major contribution to the design of the trial, the preparation of the data, its analysis or the writing of the manuscript. Every author should have participated sufficiently to take public responsibility for the content. It is the responsibility of the clinical board of EurNet to determine and agree the composition of writing committees.

Additional specific publications from the contributing medial disciplines (e.g. reference facilities, quality assurance) are welcome as long as they do not preclude the publication of major study results. Authorship of such publications, including abstracts, will represent those persons who made a major contribution to the preparation of the data, its analysis or the writing of the manuscript.

All publications (abstracts or manuscripts) require written approval of the intergroup chairpersons.

20 REFERENCES

1. Anderson RA, Sharpe RM. Regulation of inhibin production in the human male and its clinical applications. Int J Androl; 2000; 23: 136-144.
2. Baker KS, Gordon BG, Gross TG, Abromowitch MA, Lyden ER, Lynch JC, Vose JM, Armitage JO, Coccia PF, Bierman PJ.: Autologous hematopoietic stem-cell transplantation for relapsed or refractory Hodgkin's lymphoma in children and adolescents. J Clin Oncol. 1999;17:825-31.
3. Becherer A, Mitterbauer M, Jaeger U, Kalhs P, Greinix HT, Karanikas G, Potzi C, Raderer M, Dudczak R, Kletter K.: Positron emission tomography with [18F]2-fluoro-D-2-deoxyglucose (FDG-PET) predicts relapse of malignant lymphoma after high-dose therapy with stem cell transplantation. Leukemia. 2002;16:260-7.
4. Bhatia S, Yasui Y, Robison LL, Birch JM, Bogue MK, Diller L, DeLaat C, Fossati-Bellani F, Morgan E, Oberlin O, Reaman G, Ruymann FB, Tersak J, Meadows AT; Late Effects Study Group. High risk of subsequent neoplasms continues with extended follow-up of childhood Hodgkin's lymphoma: report from the Late Effects Study Group.J Clin Oncol. 2003; 21:4386-94
5. Brämswig JH, Heimes U, Heiermann E, Schlegel W, Nieschlag E, Schellong G.: The effects of different cumulative doses of chemotherapy on testicular function. Results in 75 patients treated for Hodgkin's lymphoma during childhood or adolescence. Cancer. 1990;65:1298-302.
6. Byrne J, Fears TR, Gail MH, Pee D, Connelly RR, Austin DF, Holmes GF, Holmes FF, Latourette HB, Meigs JW, et al.: Early menopause in long-term survivors of cancer during adolescence. Am J Obstet Gynecol. 1992;166:788-93.
7. Carter SK, Livingston RB.: Single-agent therapy for Hodgkin's lymphoma. Arch Intern Med. 1973;131:377-87.
8. Cremerius U, Effert PJ, Adam G, Sabri O, Zimmy M, Wagenknecht G, Jakse G, Buell U.: FDG PET for detection and therapy control of metastatic germ cell tumour. J Nucl Med. 1998;39:815-22.
9. Creutzig U, Belohradsky B (Hrsg.): Infektionsprophylaxe bei hämatologisch-onkologischen Patienten in der Pädiatrie. Sonderband Klinische Pädiatrie, 2001, S1 Band 213: A1-114.
10. Crofton PM, Thomson AB, Evans AEM, et al. Is inhibin B a potential marker of gonadotoxicity in prepubertal children treated for cancer? Clin Endocrinol (Oxf); 2003; 58: 296-301.
11. de Wit M, Bohuslavizki KH, Buchert R, Bumann D, Clausen M, Hossfeld DK.: 18FDG-PET following treatment as valid predictor for disease-free survival in Hodgkin's lymphoma. Ann Oncol. 2001;12:29-37.
12. Dieckmann K, Potter R, Wagner W, Prott FJ, Hornig-Franz I, Rath B, Schellong G.: Up-front centralized data review and individualized treatment proposals in a multicenter pediatric Hodgkin's lymphoma trial with 71 participating hospitals: the experience of the German-Austrian pediatric multicenter trial DAL-HD-90. Radiother Oncol. 2002 ; 62:191-200.
13. Dittmann H, Sokler M, Kollmannsberger C, Dohmen BM, Baumann C, Kopp A, Bares R, Claussen CD, Kanz L, Bokemeyer C.: Comparison of 18FDG-PET with CT scans in the

evaluation of patients with residual and recurrent Hodgkin's lymphoma. Oncol Rep. 2001 ;8:1393-9.

14. Dorffel W, Luders H, Ruhl U, Albrecht M, Marciniak H, Parwaresch R, Potter R, Schellong G, Schwarze EW, Wickmann L.: Preliminary results of the multicenter trial GPOH-HD 95 for the treatment of Hodgkin’s lymphoma in children and adolescents: analysis and outlook. Klin Padiatr. 2003;215:139-45.
15. Frankovich J, Donaldson SS, Lee Y, Wong RM, Amylon M, Verneris MR. High-dose therapy and autologous hematopoietic cell transplantation in children with primary refractory and relapsed Hodgkin’s lymphoma: atopy predicts idiopathic diffuse lung injury syndromes. Biol Blood Marrow Transplant. 2001;7:49-57.
16. Frei E 3rd, Luce JK, Talley RW, Vaitkevicius VK, Wilson HE.: 5-(3,3-dimethyl-1-triazeno)imidazole-4-carboxamide (NSC-45388) in the treatment of lymphoma. Cancer Chemother Rep. 1972;56:667-70.
17. Front D, Bar-Shalom R, Mor M, Haim N, Epelbaum R, Frenkel A, Gaitini D, Kolodny GM, Israel O.: Hodgkin disease: prediction of outcome with 67Ga scintigraphy after one cycle of chemotherapy. Radiology. 1999 ;210:487-91.
18. Gerres L, Bramswig JH, Schlegel W, Jurgens H, Schellong G.: The effects of etoposide on testicular function in boys treated for Hodgkin’s lymphoma. Cancer. 1998; 83:2217-22.
19. Hasenclever D, Diehl V. A prognostic score for advanced Hodgkin’s lymphoma. International Prognostic Factors Project on Advanced Hodgkin's Disease. N Engl J Med. 1998; 339:1506-14.
20. Hasenclever D, Löffler M, Diehl V: Rationale for dose escalation of first line conventional chemotherapy in advanced Hodgkin’s lymphoma. Annals of Oncology 7(Suppl.4) 1996; S95-S98
21. Hasenclever D, Brosteanu O, Gerike T, Loeffler M. Modelling of chemotherapy: The effective dose approach.Annals of Hematology 2001; 80(S3): 89-94
22. Hasenclever D, Körholz D: Design von Therapie Titrations-Studien (TTS) - Soll man randomisieren.48. Jahrestagung der GMDS, 14.-19. September 2003, Münster, Informatik, Biometrie und Epidemiologie in Medizin und Biologie 2003: 34(3) 266-268,
23. Hassel JU, Bramswig JH, Schlegel W, Schellong G.: [Testicular function after OPA/COMP chemotherapy without Procarbazine in boys with Hodgkin’s lymphoma. Results in 25 patients of the DAL-HD-85 study] Klin Padiatr. 1991;203:268-72.
24. Hauschild A, Moller M, Lischner S, Christophers E. Repeatable acute rhabdomyolysis with multiple organ dysfunction because of interferon alpha and dacarbazine treatment in metastatic melanoma. Br J Dermatol. 2001; 144:215-6
25. Hudson MM, Donaldson SS.: Treatment of pediatric Hodgkin's lymphoma. Semin Hematol. 1999; 36:313-23.
26. Hutchings M, Eigtved AI, Specht L. FDG-PET in the clinical management of Hodgkin lymphoma. Crit Rev Oncol Hematol. 2004; 52:19-32.
27. Hutchings M, Loft A, Hansen M, Moeller Pederson L, Buhl T, Jurlander J, Buus S, Keiding S, D’Amore F, Boesen AM, Kiil Berthelsen A, Specht L. FDG-PET after two cycles of

chemotherapy predicts treatment failure and progression-free survival in Hodgkin's lymphoma. Blood 2006;107:52-59

28. Jerusalem G, Beguin Y, Fassotte MF, Najjar F, Paulus P, Rigo P, Fillet G.: Whole-body positron emission tomography using 18F-fluorodeoxyglucose for posttreatment evaluation in Hodgkin's lymphoma and non-Hodgkin's lymphoma has higher diagnostic and prognostic value than classical computed tomography scan imaging. Blood. 1999;94:429-33.
29. Jerusalem G, Beguin Y, Fassotte MF, Najjar F, Paulus P, Rigo P, Fillet G.: Whole-body positron emission tomography using 18F-fluorodeoxyglucose compared to standard procedures for staging patients with Hodgkin's lymphoma. Haematologica. 2001;86:266-73.
30. Josting A, Rueffer U, Franklin J, Sieber M, Diehl V, Engert A.: Prognostic factors and treatment outcome in primary progressive Hodgkin lymphoma: a report from the German Hodgkin Lymphoma Study Group. Blood. 2000;96:1280-6.
31. Josting A, Franklin J, May M, Koch P, Beykirch MK, Heinz J, Rudolph C, Diehl V, Engert A. New prognostic score based on treatment outcome of patients with relapsed Hodgkin's lymphoma registered in the database of the German Hodgkin's lymphoma study group. J Clin Oncol. 2002; 1:221-30.
32. Josting A, Rudolph C, Reiser M, Mapara M, Sieber M, Kirchner HH, Dorken B, Hossfeld DK, Diehl V, Engert A; Participating Centers.: Time-intensified dexamethasone / cisplatin / cytarabine: an effective salvage therapy with low toxicity in patients with relapsed and refractory Hodgkin's lymphoma. Ann Oncol. 2002 (B); 13:1628-35.
33. Klener P, Donner L.: Imidazole carboxamide (DTIC) in the treatment of advanced lymphomas. Efficacy of DTIC in cases which fail to respond to conventional chemotherupetic combinations. Acta Haematol. 1977;57:272-8.
34. Körholz D, Kluge R, Wickmann L, Hirsch W, Lüders H, Lotz I, Dannenberg C, Hasenclever D, Dörffel W, Sabri O.: Importance of F18 Fluorodeoxy-D-2-Glucose positron Emission Tomography (FDG-PET) for Staging and Therapy Control of Hodgkin`s Lymphoma in childhood and adolescence – Consequences for the EURONET-PHL-C-1 protocol. Onkologie 26:489-93.
35. Kollmannsberger C, Beyer J, Droz JP, Harstrick A, Hartmann JT, Biron P, Flechon A, Schoffski P, Kuczyk M, Schmoll HJ, Kanz L, Bokemeyer C.: Secondary leukemia following high cumulative doses of etoposide in patients treated for advanced germ cell tumours. J Clin Oncol. 1998;16:3386-91.
36. Kostakoglu L, Coleman M, Leonard JP, Kuji I, Zoe H, Goldsmith SJ.: PET predicts prognosis after 1 cycle of chemotherapy in aggressive lymphoma and Hodgkin's lymphoma. J Nucl Med. 2002;43:1018-27.
37. Kostakoglu L, Leonard JP, Kuji I, Coleman M, Vallabhajosula S, Goldsmith SJ.: Comparison of fluorine-18 fluorodeoxyglucose positron emission tomography and Ga-67 scintigraphy in evaluation of lymphoma. Cancer. 2002;94:879-88.
38. Kreuser ED, Xiros N, Hetzel WD, Heimpel H.: Reproductive and endocrine gonadal capacity in patients treated with COPP chemotherapy for Hodgkin's lymphoma. J Cancer Res Clin Oncol. 1987;113:260-6.

39. Lang O, Bihl H, Hultenschmidt B, Sautter-Bihl ML.: Clinical relevance of positron emission tomography (PET) in treatment control and relapse of Hodgkin’s lymphoma. Strahlenther Onkol. 2001;177:138-44.
40. Linch DC, Winfield D, Goldstone AH, Moir D, Hancock B, McMillan A, Chopra R, Milligan D, Hudson GV.: Dose intensification with autologous bone-marrow transplantation in relapsed and resistant Hodgkin’s lymphoma: results of a BNLI randomised trial. Lancet. 1993;341:1051-4.
41. Loeffler M, Diehl V, Pfreundschuh M, Rühl U, Hasenclever D, et al. :Dose-Response Relationship of Complementary Radiotherapy Following Four Cycles of Combination Chemotherapy in Intermediate-Stage Hodgkin's Disease.Journal of Clinical Oncology 1997; 15: 2275-2287
42. Meadows AT, Obringer AC, Marrero O, Oberlin O, Robison L, Fossati-Bellani F, Green D, Voute PA, Morris-Jones P, Greenberg M, et al.: Second malignant neoplasms following childhood Hodgkin’s lymphoma: treatment and splenectomy as risk factors. Med Pediatr Oncol. 1989;17:477-84.
43. Montravers F, McNamara D, Landman-Parker J, Grahek D, Kerrou K, Younsi N, Wioland M, Leverger G, Talbot JN.: [(18)F]FDG in childhood lymphoma: clinical utility and impact on management. Eur J Nucl Med Mol Imaging. 2002; 29:1155-65.
44. Nachman JB, Sposto R, Herzog P, Gilchrist GS, Wolden SL, Thomson J, Kadin ME, Pattengale P, Davis PC, Hutchinson RJ, White K; Children's Cancer Group.: Randomised comparison of low-dose involved-field radiotherapy and no radiotherapy for children with Hodgkin’s lymphoma who achieve a complete response to chemotherapy. J Clin Oncol. 2002; 20:3765-71.
45. Naumann R, Vaic A, Beuthien-Baumann B, Bredow J, Kropp J, Kittner T, Franke WG, Ehninger G.: Prognostic value of positron emission tomography in the evaluation of post-treatment residual mass in patients with Hodgkin’s lymphoma and non-Hodgkin's lymphoma. Br J Haematol. 2001;115:793-800.
46. Partridge S, Timothy A, O'Doherty MJ, Hain SF, Rankin S, Mikhaeel G.: 2-Fluorine-18-fluoro-2-deoxy-D glucose positron emission tomography in the pretreatment staging of Hodgkin’s lymphoma: influence on patient management in a single institution. Ann Oncol. 2000;11:1273-9.
47. Pocock SJ (1983): Clinical Trials. John Wiley, Chichester, New York
48. Roberts VJ, Barth S, el-Roeiy A, Yen SS. Expression of inhibin/activin subunits and follistatin messenger ribonucleic acids and proteins in ovarian follicles and the corpus luteum during the human menstrual cycle. J Clin Endocrinol Metab; 77: 1993; 1402-1410.
49. Schellong G, Bramswig J, Ludwig R, Gerein V, Jobke A, Jurgens H, Kabisch H, Stollmann B, Weinel P, Gadner H, et al.: [Combined treatment strategy in over 200 children with Hodgkin’s lymphoma: graduated chemotherapy, involved field irradiation with low dosage and selective splenectomy. A report of the cooperative therapy study DAL-HD-82]. Klin Padiatr. 1986 A ;198:137-46

50. Schellong G, Waubke-Landwehr AK, Langermann HJ, Riehm HJ, Bramswig J, Ritter J.: Prediction of splenic involvement in children with Hodgkin’s lymphoma. Significance of clinical and intraoperative findings. A retrospective statistical analysis of 154 patients in the German therapy study DAL-HD-78. Cancer. 1986 B ;57:2049-56.
51. Schellong G, Hörnig I, Brämswig J, Bökkerink JPM, Steinhoff A, Ludwig R, Niethammer D, Reiter A, Lengerke Jv, Heinecke H, Schwarze EW, Pötter R, Müller RP, Wannenmacher M.: Zur Bedeutung des Procarbazins in der Chemotherapie des Morbus Hodgkin – Ein Bericht der kooperativen Therapiestudie DAL-HD-85. Klin Pädiatr. 1988; 200: 205 - 213
52. Schellong G, Bramswig JH, Schwarze EW, Wannenmacher M.: An approach to reduce treatment and invasive staging in childhood Hodgkin’s lymphoma: the sequence of the German DAL multicenter studies. Bull Cancer. 1988 B;75:41-51
53. Schellong G, Bramswig JH, Hornig-Franz I, Schwarze EW, Potter R, Wannenmacher M.: Hodgkin’s lymphoma in children: combined modality treatment for stages IA, IB, and IIA. Results in 356 patients of the German/Austrian Pediatric Study Group. Ann Oncol. 1994 A;5 Suppl 2:113-5.
54. Schellong G, Hornig-Franz I, Rath B, Ritter J, Riepenhausen M, Kabisch H, Goldschmitt-Wuttge B, Schmidt P, Niethammer D, Gaedicke G, et al.: [Reducing radiation dosage to 20-30 Gy in combined chemo-/radiotherapy of Hodgkin’s lymphoma in childhood. A report of the cooperative DAL-HD-87 therapy study]. Klin Padiatr. 1994 B; 206:253-62
55. Schellong G.: Treatment of children and adolescents with Hodgkin’s lymphoma: the experience of the German-Austrian Paediatric Study Group. Baillieres Clin Haematol. 1996;9:619-34.
56. Schellong G, Riepenhausen M, Creutzig U, Ritter J, Harbott J, Mann G, Gadner H.: Low risk of secondary leukemias after chemotherapy without mechlorethamine in childhood Hodgkin’s lymphoma. German-Austrian Pediatric Hodgkin's Disease Group. J Clin Oncol. 1997;15:2247-53.
57. Schellong G.: Pediatric Hodgkin’s lymphoma: treatment in the late 1990s. Ann Oncol. 1998;9 Suppl 5:S115-9
58. Schellong G, Potter R, Bramswig J, Wagner W, Prott FJ, Dorffel W, Korholz D, Mann G, Rath B, Reiter A, Weissbach G, Riepenhausen M, Thiemann M, Schwarze EW.: High cure rates and reduced long-term toxicity in pediatric Hodgkin’s lymphoma: the German-Austrian multicenter trial DAL-HD-90. The German-Austrian Pediatric Hodgkin's Disease Study Group. J Clin Oncol. 1999;17:3736-44.
59. Schellong G, Riepenhausen M. (2002) Spätfolgen nach Morbus Hodgkin bei Kindern und Jugendlichen. Ergebnisse der Studien DAL-HD-78 bis -HD-90. Projektbericht.
60. Schellong G, Dorffel W, Claviez A, Korholz D, Mann G, Scheel-Walter HG, Bokkerink JP, Riepenhausen M, Luders H, Potter R, Ruhl U; DAL/GPOH. Salvage therapy of progressive and recurrent Hodgkin’s lymphoma: results from a multicenter study of the pediatric DAL/GPOH-HD study group.J Clin Oncol. 2005;23:6181-9.

61. Schmitz N, Glass B, Dreger P, Haferlach T, Horst HA, Ollech-Chwoyka J, Suttorp M, Gassmann W, Loffler H.: High-dose chemotherapy and hematopoietic stem cell rescue in patients with relapsed Hodgkin’s lymphoma. Ann Hematol. 1993;66:251-6.
62. Schmitz N, Pfistner B, Sextro M, Sieber M, Carella AM, Haenel M, Boissevain F, Zschaber R, Muller P, Kirchner H, Lohri A, Decker S, Koch B, Hasenclever D, Goldstone AH, Diehl V; German Hodgkin's Lymphoma Study Group; Lymphoma Working Party of the European Group for Blood and Marrow Transplantation. Aggressive conventional chemotherapy compared with high-dose chemotherapy with autologous haemopoietic stem-cell transplantation for relapsed chemosensitive Hodgkin’s lymphoma: a randomised trial. Lancet. 2002;359:2065-71.
63. Spaepen K, Stroobants S, Dupont P, Thomas J, Vandenberghe P, Balzarini J, De Wolf-Peeters C, Mortelmans L, Verhoef G.: Can positron emission tomography with [(18)F]-fluorodeoxyglucose after first-line treatment distinguish Hodgkin’s lymphoma patients who need additional therapy from others in whom additional therapy would mean avoidable toxicity? Br J Haematol. 2001;115:272-8.
64. Stumpe KD, Urbinelli M, Steinert HC, Glanzmann C, Buck A, von Schulthess GK.: Whole-body positron emission tomography using fluorodeoxyglucose for staging of lymphoma: effectiveness and comparison with computed tomography. Eur J Nucl Med. 1998 ;25:721-8.
65. Sureda A, Arranz R, Iriondo A, Carreras E, Lahuerta JJ, Garcia-Conde J, Jarque I, Caballero MD, Ferra C, Lopez A, Garcia-Larana J, Cabrera R, Carrera D, Ruiz-Romero MD, Leon A, Rifon J, Diaz-Mediavilla J, Mataix R, Morey M, Moraleda JM, Altes A, Lopez-Guillermo A, de la Serna J, Fernandez-Ranada JM, Sierra J, Conde E; Grupo Espanol de Linformas/Transplante Autologo de Medula Osea Spanish Cooperative Group. Autologous stem-cell transplantation for Hodgkin’s lymphoma: results and prognostic factors in 494 patients from the Grupo Espanol de Linfomas/Transplante Autologo de Medula Osea Spanish Cooperative Group. J Clin Oncol. 2001 ;19:1395-404.
66. Tanner JM (1962). Growth at adolescence 2nd Edition. Oxford, Blackwell Science.
67. Verdeguer A, Pardo N, Madero L, Martinez A, Bureo E, Fernandez JM, Munoz A, Olive T, Fernandez-Delgado R, Cubells J, Diaz MA, Sastre A.: Autologous stem cell transplantation for advanced Hodgkin’s lymphoma in children.Spanish group for BMT in children (GETMON), Spain. Bone Marrow Transplant. 2000;25:31-4.
68. Wallace EM, Groome NP, Riley SC, Parker AC, Wu FC. Effects of chemotherapy-induced testicular damage on inhibin, gonadotrophin, and testosterone secretion: A prospective longitudinal study. J Clin Endocrinol Metab; 1997; 82: 3111-3115.
69. Wallace WH, Thomson AB, Saran F, Kelsey TW. Predicting age of ovarian failure after radiation to a field that includes the ovaries. Int J Radiat Oncol Biol Phys. 2005; 62:738-44.
70. Weihrauch MR, Re D, Scheidhauer K, Ansen S, Dietlein M, Bischoff S, Bohlen H, Wolf J, Schicha H, Diehl V, Tesch H.: Thoracic positron emission tomography using 18F-fluorodeoxyglucose for the evaluation of residual mediastinal Hodgkin disease. Blood. 2001;98:2930-4.

71. Weihrauch MR, Re D, Bischoff S, Dietlein M, Scheidhauer K, Krug B, Textoris F, Ansen S, Franklin J, Bohlen H, Wolf J, Schicha H, Diehl V, Tesch H.: Whole-body positron emission tomography using 18F-fluorodeoxyglucose for initial staging of patients with Hodgkin's lymphoma. Ann Hematol. 2002;81:20-5.
72. Whitlock JA, Greer JP, Lukens JN.: Epipodophyllotoxin-related leukemia. Identification of a new subset of secondary leukemia. Cancer. 1991;68:600-4.
73. Weidmann E, Baican B, Hertel A, Baum RP, Chow KU, Knupp B, Adams S, Hor G, Hoelzer D, Mitrou PS.: Positron emission tomography (PET) for staging and evaluation of response to treatment in patients with Hodgkin's lymphoma. Leuk Lymphoma. 1999;34:545-51.
74. Winick NJ, McKenna RW, Shuster JJ, Schneider NR, Borowitz MJ, Bowman WP, Jacaruso D, Kamen BA, Buchanan GR.: Secondary acute myeloid leukemia in children with acute lymphoblastic leukaemia treated with etoposide. J Clin Oncol. 1993;11:209-17.
75. Young H, Baum R, Cremerius U, Herholz K, Hoekstra O, Lammertsma AA, Pruim J, Price P: Measurement of clinical and subclinical tumour response using [18F]-fluorodeoxyglucose and positron emission tomography: review and 1999 EORTC recommendations. European Organisation for Research and Treatment of Cancer (EORTC) PET Study Group. Eur J Cancer. 1999;35:1773-82.
76. Zintl F, Suttorp M, Berthold F, Dörffel W (2002) Autologe Stammzelltransplantation (ASZT) bei Kindern mit rezidivierenden oder therapierefraktären Hodgkin-Lymphomen (Abstract). Monatsschr Kinderheilkd, 150, 555.
77. Declaration of Helsinki: Guiding Physicians in Biomedical Research Involving Human Subjects. Adopted by the 18th World Medical Assembly, Helsinki (Finland), June 1964. Last amendment by the 48th General Assembly, Somerset West (Rep. of South Africa) 1996
78. International Conference on Harmonisation of Technical Requirements for the Registration of Pharmaceutical Products for Human Use: ICH Harmonized Tripartite Guideline, "Guideline for Good Clinical Practice". Recommended for Adoption at Step 4 of the ICH Process on 1 May 1996. www.ifpma.org/ich5e.html#GCP
79. International Conference on Harmonisation of Technical Requirements for the Registration of Pharmaceutical Products for Human Use: ICH Harmonized Tripartite Guideline, "Clinical Data Safety Management: Definitions and Standards for Expedited Reporting". Recommended for Adoption at Step 4 of the ICH Process on 27 May 1994. www.ifpma.org/ich5e.html#Safety

21 CONFIRMATION OF THE FINAL PROTOCOL

The signatories declare that they agree to fullfill their responsibilities within this study in accordance with local law, the declaration of Helsinki, ICH-GCP and the study protocol as presented.

Coordinating investigator<br>Inter-group Chairperson<br>GPOH-HD<br>Prof. Dr. Dieter Koerholz	07/08/2006<br>Date	Signature
Inter-group Chairperson<br>CCLG<br>Hamish Wallace MD,<br>FRCPCH, FRCP (Edin)	07/08/2006<br>Date	Signature
Inter-group Chairperson<br>SFCE<br>Pr. Judith Landman-Parker	07/08/2006<br>Date	Signature
Responsible Biometrician<br>Dr. Dirk Hasenclever	07/08/2006<br>Date	Signature

22 PROTOCOL AGREEMENT

The signatory declares

- that he/she agrees to conduct his/her responsibilities within this study in accordance with local law, the declaration of Helsinki, ICH-GCP and the study protocol as presented
- that he/she has familiarised his/herself with the results of the pharmacological and toxicological trials of the investigational product and the results of other studies carried out to date
- that he/she has read the study protocol and agrees to it in its entirety
- that he/she intends to adhere to the schedule as specified below.

Date: __________________________

Signature of the principal investigator: __________________________

Affiliation /address: __________________________

____

____

____

23 APPENDIX

23.1 FACTS OF PUBERTY

MALE

Background

- The seminiferous epithelium of normal infant and child testes consists of immature Sertoli cells and spermatogonia. Primary spermatocytes, which degenerate and do not progress to spermatozoa, have been identified in some boys between the ages of 4-13 years.

Puberty

- Spermarche occurs at a median age of 13.4 (range 11.7-15.3) years at a time when median testicular size is 11.5 (range 4.7-19.6) ml.
- The prepubertal testis is approximately 2 ml in volume. The onset of puberty begins with enlargement of the testis (4 ml volume) at approximately 11.4 years. The longitudinal growth spurt starts when the testes are approximately 8 ml and is maximal at approximately 12 ml.
- The normal adult testis is 15 to 25 ml. Azoospermia is likely if the volume of each adult testis is 10 ml or less.

Puberty and growth

- The timing of the onset of the growth spurt relative to the onset of puberty differs in a characteristic fashion between the sexes, occurring earlier in girls than in boys (10-12 ml volume testes). The spinal component is an important part of the growth spurt.
- Loss of harmony in pubertal development occurs if the relationship between height velocity and pubertal stage is lost, ie: a boy with 8-10 ml volume testes should have a growth spurt (10-16 cm/yr).
- Bone age is a good guide to how much growth is past and how much is left to come. If bone age is advanced relative to chronological age, the height prediction is reduced. Bone age cannot predict the onset of puberty or the timing of the peak of the adolescent growth spurt.

FEMALE

Background

- Oogonia arising from the primordial germ cells in the yolk sac reach a complement of 6-7 million by the sixth month of gestation; these represent the total fixed number of germ cells available. Primordial follicles consist of a primary oocyte surrounded by a single layer of spindle-shaped cells. By the time of birth, the pool of primordial follicles has already been reduced to 2-4 million by ongoing apoptosis and further attrition leaves approximately 400,000 by the time of menarche.

Puberty

- The onset of normal female puberty is characterised by the appearance of breast buds (breast stage 2, B2) at a mean age of 11.4 years, but ranging from as early as 8.4 years age to as late as 13.5 years. Any girl with breast buds before 8.4 years age has precocious puberty, whilst the absence of breast development in a girl older than 13.5 years requires endocrine assessment to ascertain the cause of the delay.
- During childhood, increased amplitude, frequency and duration of gonadotrophin pulsatility, will result in consonant pubertal progression, taking an average of two years to menarche (at B3 or B4), at mean age 12.4 (range 10-14.5) years.
- The attainment of breast stage 4 (B4) is a prerequisite for the onset of menstruation.
- For the first year after menarche, menstrual cycles are often anovulatory but ovulatory cycles, and thus the potential for fertility, can occasionally occur in girls whose sexual development is not quite complete.

Puberty and growth

- The timing of the onset of the growth spurt relative to the onset of puberty differs in a characteristic fashion between the sexes, occurring earlier in girls (breast stage 2 and 3) than in boys. The spinal component is an important part of the growth spurt.
- After the onset of menarche, only 3-5 cms of growth in height remain.
- Loss of harmony in pubertal development occurs if the relationship between height velocity and pubertal stage is lost, ie: a girl who is breast stage 2-3 should have a growth spurt (10-16 cm/yr).
- Bone age is a good guide to how much growth is past and how much is left to come. If bone age is advanced relative to chronological age, the height prediction is reduced. Bone age cannot predict the onset of puberty or the timing of the peak of the adolescent growth spurt.

23.2 CHEMOTHERAPUTIC DRUG MONOGRAPHS

23.2.1 BLEOMYCIN

SECTION 1

Alternative names
None

Mechanism of action

DNA strand cleavage via free radicals produced by activated bleomycin complexes.
DNA intercalator, resulting in base excision and strand breakage.

Considerations prior to administration

Pulmonary Function
Adequate renal function

Adverse events

Early

Common
- Fevers (25%)

Occasional
- Skin (erythema, hyperkeratosis, peeling, pigmentation, bullae, ulceration.)

Rare
- Alopecia
- Nail Changes
- Anaphylaxis

Late
Occasional
- Pulmonary Fibrosis

SECTION 2

Recommended routes

intravenous bolus or 12 hr infusion
IM with lignocaine
intrapleural or intralesional

CAUTION

Dose should be reduced in the renal function is impaired[4,6].

Administration

Bolus administration - inject slowly or via a fast running drip.
Intravenous administration - dilute in up to 100 mls 0.9% sodium chloride and administer slowly.[7]

Dose/Schedule

Every 21 days as part of combined chemotherapy for teratoma 30mg stat or per m^2

Interactions

Oxygen enhances pulmonary toxicity - even years after exposure.
Cisplatin increases risk of pulmonary toxicity.

SECTION 3

Dilution specification and stability[7]

- 0.9% sodium chloride **only.**
- Protect from light.
- Stable for up to 7 days after reconstitution provided it is protected from light and store in a refridgerator.

Pharmacokinetics

Pharmacokinetics of bleomycin is confounded by the mixture of components present in the pharmaceutical preparation. Using radio-immunoassay, half-life was reported to be 2.3 hr, with clearance of 68 ml/min/m^2 and volume of distribution[6] of 19 l/m^2. 64 % of the dose was excreted in urine and clearance correlated well with serum creatinine[2]. Similar pharmacokinetic were seen after intramuscular administration[3]. The elimination of bleomycin has been reported to be faster in children less than 3 years than in older children or adults[5]. Intraperitoneal administration results in low plasma concentrations and half-lives in peritoneal fluid and plasma of 4.2 and 5.0 hours respectively[1].

Pharmacodynamics

No data are available, although pulmonary toxicity may be related to peak bleomycin plasma concentration[5].

References

1. Cancer, 1985, 56, 2420-2423. Intraperitoneal bleomycin. Pharmacokinetics and results of a phase II trial. Bitran JD.
2. Cancer Chemotherapy and Pharmacology, 1982, 9, 22-25. Bleomycin clinical pharmacology by radioimmunoassay. Hall SW et al.

3. Cancer Treatment Reports, 1981, 65, 485-489. Pharmacokinetics of bleomycin after im administration in man. Oken MM et al.
4. Seminars in Oncology, 1992, 19 (2 Suppl 5), 3-8. Bleomycin pharmacology: mechanism of action and resistance, and clinical pharmacokinetics. Dorr RT.
5. Clinical Pharmacokinetics, 1987, 12, 168-213. Pharmacokinetics of anticancer drugs in children. Crom WR et al.
6. Cancer Treatment Reports, 1977, 61, 1631-1636. Effects of variations in renal function on the clinical pharmacology of bleomycin administered as an iv bolus. Crooke ST et al.
7. The Cytotoxic Handbook. Third Edition. Ed: M Allwood A Stanley and P Wright. Radcliffe Medical Press 1997.

23.2.2 CARMUSTINE

SECTION 1

Alternative names
- BCNU
- BiCNU[1]
- 1,3-bis(2-chloroethyl)-1-nitrosourea

Mechanism of action
- A nitrosourea acting partially as an alkylating agent.
- Cell cycle non specific

Considerations prior to administration
- Full blood count
- Renal function

Adverse effects

Early

Common
- Myelosupression (nadir at 4-6 weeks)
- Emesis (dose related)
- Flushing
- Mucositis
- Alopecia

Occasional
- Abnormal liver function, dose related[2,3]
- Neurotoxicity, dose related

Rare
- Neurotoxicity including ocular events with intracarotid or high dose administration[4-6]

Late

Rare
- Pulmonary fibrosis, not dose related[7] , but age related[8]
- Renal failure, if total doses exceed 1200 mg/m^2 [9]

SECTION 2

Recommended routes
Intravenous

CAUTION
Vesicant

Administration
Slow iv or infusion over 1-2 hours

Dose/Schedule
Various. See individual protocols.

Interactions

- Sodium bicarbonate decreases BCNU stabitity[10]
- Cimetidine may increase myelosupression[11]
- Cisplatin and cyclophosphamide pretreatment may reduce the rate of elimination of BCNU[12]

Overdose
No data

SECTION 3

Dilution specification and stability

Dilute in supplied solvent and add water as per instructions to make a 3.3mg/ml solution.
Further dilute in 5% dextrose or 0.9% saline.
Store in fridge and protect from light.
Use within 48hrs
Do not use vial if BCNU is in liquid form.

Pharmacokinetics

There is little or no data in paediatrics.
Lipid soluble and distributes rapidly[13].
Crosses blood brain barrier, appears in CSF almost immediately
Rapid spontaneous degradation due to hydrolysis (< 15 minutes).
Probably has active metabolites.
An elimination half-life of 18 minutes, volume of distribution of 2.6l/kg and clearance of 17ml/min/kg have been reported[14].
High dose BCNU has the similar pharmacokinetics as conventional dose, with a half life of 22 minutes, clearance of 78ml/min/kg and protein binding of 77%.

Pharmacodynamics
The AUC of BCNU is very variable (up to 10 fold) amongst patients. An AUC
Of greater than 600μg/ml is associated with an increased risk of pulmonary injury.[15]

REFERENCES
1 BCNU, Bristol Myers, Pharmacological Issues, ref: KF/KD/0027K

2 PAACR 1980, 21 p180. Intensive BCNU and autologous bone marrow transplantation for refractory anaemia, a phase I study. Phillips et al.
3 PAACR 1980, 21, p341. Single high dose BCNU with autologous bone marrow: a phase I study, Takvoran et al.
4 Cancer 1981, 48, 1318-1327. Encephalomyopathy following high-dose BCNU therapy, Burger et al.
5 Cancer 1986, 57, 1276-1280. Intracarotid BCNU leukoencephalopathy, Kleinschmidt-DeMasters,B.
6 Neurology, 1988, 38, 284-289. Visual system toxicity following intra-arterial therapy, Kupersmith et al.
7 Cancer 1981, 48, 909-911. Pulmonary fibrosis: A complication of BCNU therapy, Ryan,B and Walters,T.
8 Late carmustine lung fibrosis. Age at treatment may influence severity and survival. Chest 107 (5): 1355-7 (1995)
9 Kidney International, 1978, 14, 611. Chronic interstitial nephritis and renal failure due to nitrosourea therapy, Schacht and Baldwin.
10 Am J Hosp Pharm 1980, 37, 677-678. Stability of carmustine in the presence of sodium bicarbonate. Colvin et al.
11 The New England Journal of Medicine 1978, 299, 834. Bone marrow depression with cimetidine plus carmustine, Selker et al.
12. Cancer Chemotherapy and Pharmacology, 1994, 35, 59-63. Cyclophosphamide, cisplatin and carmustine: pharmacokinetics of carmustine following multiple alkylating agent interactions, Jones RB et al.
13 Cancer Treat Rep, 1978, 62, 1305-1312. Pharmacokinetics of BCNU in man: a preliminary study of 20 patients, Levin et al.
14 Cancer Treatment Reports, 1981, 65, 555-562. Differential pulse polarographic determination of BCNU pharmacokinetics in patients with lung cancer. Russo, R. et al.
15 Journal of the National Cancer Institute, 1993, 85, 640-647. Acute lung injury following treatment with high-dose cyclophosphamide, cisplatin and carmustine: pharmacodynamic evaluation of carmustine. Jones RB et al.

23.2.3 CYCLOPHOSPHAMIDE

SECTION 1

Alternative names

- Cyclophospham
- Endoxana TM
- Cytoxan TM .

Mechanism of Action

Oxazaphosphorine alkylating agent. Cyclophosphamide is a prodrug which undergoes biotransformation primarily by hepatic P450 mixed function oxidases to 4-hydroxycyclophosphamide. This metabolite decomposes spontaneously to produce the bifunctional alkylating species phosphoramide mustard. Bi-functional alkylating agents are thought to exert their cytotoxicity by forming intra-stand and inter-strand DNA cross-links at the N7 position of guanine residues. The generation of phosphoramide mustard is accompanied by the production of the metabolite acrolein which is thought to be partially responsible for the dose-limiting urotoxic effects of the drug. Co-administration of the uroprotectactant agent mesna (Sodium mercaptoethane sulphonate) can help prevent urotoxicity.

Considerations prior to administration
- Concurrent acute urinary-tract infection.
- Urothelial damage following previous cytotoxic chemotherapy or pelvic irradiation.
- Full Blood Count
- Renal function
- Liver function

Adverse effects

Common
- Dose related nausea and vomiting
- Alopecia
- Chemical or haemorrhagic cystitis if administered without mesna or with inadequate hydration and micturition.

Occasional
- SIADH

Rare
Cardiotoxicity presenting as congestive cardiac failure, pericardial effusion and pericardial tamponade. Possible association with previous anthracycline therapy or mediastinal irradiation.

SECTION 2

Recommended routes
Intravenous

Administration
By slow bolus into established IV line or by intravenous infusion over 1 hour.
By IV infusion in Glucose 5%, Sodium chloride 0.9% or Glucose/saline.

Dose/schedule

In order to prevent urothelial toxicity, hydration and mesna are required, particularly with higher daily doses of the drug.

Hydration and mesna

The manufacturers recommend concurrent mesna administration at daily doses of cyclophosphamide in excess of 10 mg/kg. In paediatric clinical practice, mesna is not required until higher daily, or higher cumulative doses per course are exceeded, providing adequate hydration and micturition can be maintained.

Daily cyclophosphamide doses < 10mg/kg (< 300 mg / m^2)
No mesna required, maintain fluid intake, encourage frequent micturition.

Daily or total course cyclophosphamide dose 300 mg / m^2 to 1 g / m^2

No mesna required. Intravenous hydration with glucose/ saline solution at a rate of 3 l / m^2/ 24 hours commencing with the first cyclophosphamide dose and continuing for at least six hours after last cyclophosphamide dose.

Daily or total course cyclophosphamide dose > 1 g / m^2
Intravenous hydration with glucose/saline solution containing mesna at 120% (mg/mg) of the prescribed daily cyclophosphamide dose. Infuse this solution at a rate of 3 l / m^2 / 24 hours, commencing 3 hours before the first cyclophosphamide dose and continuing for a minimum of 12 hours after completion of the last cyclophosphamide infusion.

Interactions

Possible with previous or current exposure to hepatic enzyme inducing agents including phenytoin[2].
Concurrent dexamethasone treatment may increase cyclophosphamide metabolism[2]
Concurrent allopurinol administration may decrease cyclophosphamide metabolism[2]

SECTION 3

Dilution specification

Cyclophosphamide is reconstituted with water for Injections BP to produce a final concentration of 20 mg/ml. At this concentration, absorptive losses onto glass, PVC and polypropylene are thought to be negligible [3,4]
Compatible with glucose 5%, Sodium chloride 0.9% and glucose/saline solutions

Stability

Cyclophosphamide appears to be chemically stable when stored at 4°C. A large body of information exists on stability and compatibility of cyclophosphamide in solution.[3,4]

Pharmacokinetics

The pharmacokinetics of cyclophosphamide are complex, and since the anti-tumour activity of the oxazaphosphorines rests with their metabolites, little information can be gained from the pharmacokinetics of the parent drug. In children, the plasma half-life of cyclophosphamide ranges from 2.15 to 8.15 hours. Urinary excretion of cyclophosphamide and its metabolites is largely complete within 24 hours of administration[1]. Plasma half-life, apparent volume of distribution and total body clearance increase with increasing dose. Daily administration of cyclophosphamide over 2-4 days results in auto-induction of metabolism but this cannot be demonstrated with repeated 3-weekly courses of the drug [2].

Pharmacodynamics

The role of individual metabolites in producing tumour responses is still not clear. In children, there is significant inter-patient variation in metabolism and pharmacokinetics [1,2.], but the clinical consequences of these variations remain unknown. No correlation between either total plasma alkylating activity or individual metabolite plasma AUC's and tumour response has been demonstrated.

References

1. Pharmacokinetics and metabolism of cyclophosphamide in paediatric patients. Tasso, Boddy et al. Cancer Chemother Pharmacol (1992) 30: 207-211
2. Cyclophosphamide metabolism in Children. Yule, Boddy et al. Cancer Research 55, 803-809, 1995
3. The Cytotoxic Handbook. II Edition. Alwood M, Wright P. 1993
4. Handbook of Injectable Drugs. IX Edition. Trissel LA. 1996

23.2.4 CYTARABINE

SECTION ONE

Alternative names:

- Cytosine Arabinoside
- Ara -C
- Arabinosylcytosine
- Cytosar ™

Mechanism of action

- Pyrimidine nucleoside analogue which inhibits the synthesis of DNA.
- Cell cycle phase specific for S phase.

Considerations prior to administration

- Cytarabine is contra-indicated in patients with known hypersensitivity to the drug.
- Severe and fatal CNS, GI and pulmonary toxicities have occurred with cytarabine.
- Full blood count
- Prophylactic steroid eye drops (e.g. prednisolone) must be given with higher doses of cytarabine (>1g/m^2) to prevent conjunctivitis
- Corticosteroids can be used to treat/prevent ‘cytarabine reaction’
- Use with caution and at reduced dose in patients with poor liver function

Adverse events [1,2]

Common

- Bone marrow suppression
- Nausea and Vomiting
- Diarrhoea
- Mild oral ulceration
- Conjunctivitis with high doses

Occasional

- ‘Cytarabine’ reaction including ‘Flu’-like symptoms, fever, myalgia, bone pain, chest pain, rash, conjuctivitis and malaise 6-12 hours after administration
- CNS and cerebellar toxicity at higher doses

Rare

- Hepatotoxicity
- Pancreatitis
- Meningismus, parasthesias and paraplegias and seizures
- Severe and sometimes fatal CNS, GI and pulmonary toxicity with higher doses

SECTION TWO

Recommended routes

Cytarabine can be given by intravenous (bolus or intermittent or continuous infusion), subcutaneous (injection and infusion) and intrathecal injection[2,3,4]

Presentation

Ampoules: cytarabine isotonic solution 20mg/ml (2ml and 5ml size) Alexan™
cytarabine hypertonic solution 100mg/ml (1ml and 10ml size) Alexan™

Vials: cytarabine powder 100mg,500mg and 1000mg with diluent

CAUTION

The high strength injection solution should not be given intrathecally due to the hypertonicity of the solution.

Administration[2,3]

Bolus Administration:
SC and IV bolus injections are used in maintenance therapy.

IV infusion:
- High Dose Schedules may be administered by intermittent or continuous infusion in 0.9% Sodium Chloride or 5% Glucose.
- To reduce toxicity infusions should be not less than one hour
- Continuous infusions of cytarabine have ranged from 8-12 hours to 120-168 hours.

Interactions

- Absorption of oral digoxin may be substantially reduced in patients receiving cytarabine.
- Cytarabine may antagonise the activity of gentamicin against K pneumonia and of flucytosine against fungi
- G-CSF may increase the cytotoxic activity of cytarabine
- Fludarabine enhances the production of ara CTP (the active metabolite of cytarabine) and thereby enhances it’s cytotoxicity

Overdose

Cessation of therapy followed by supportive care for bone marrow depression.[2]

SECTION THREE

Dilution specification and stability[3]

- Dry powder vials should be reconstituted with water for injection.
- Cytarabine may then be further diluted (if necessary) with Sodium Chloride 0.9% or Dextrose 5%
- In 0.9% sodium chloride, no decomposition occurred after 24 hours, but a 3% loss at room temperature and a 6% loss at 30^0C was observed over 7 days
- Cytarabine at a concentration 20mg/ml in 0.9% sodium chloride is stable for 14 days at 4^0C
- Photo-degradation of cytarabine does not appear to be significant

Pharmacokinetics[2]

- Cytarabine is deaminated to arabinofuranosyl uracil in liver and kidneys**.** It is a prodrug, which is activated to cytarabine 5'-triphosphate (araCTP) within cells[6].
- Cytosine is metabolised rapidly, primarily by the liver and perhaps by the kidney
- After a 3hour infusion, elimination is biphasic with an initial $t_{1/2}$ of 10mins and reported terminal half-lives of 2.4 to 4.8 hours[6,11]. AraCTP has a longer half-life of 3.3 to 6.9 hours[6].
- Shorter half-lives (less than 2 hours) may be observed with bolus administration[8].
- 5.8% of the dose is excreted unchanged in the urine within 12-24 hours[7].
- 90% of the dose is excreted as the deaminated product[7].
- Widely distributed into tissues, across the blood brain barrier and placenta. Concentrations of cytarabine in the CSF are similar to those in plasma, with possible accumulation of araU in CSF on repeated dosing[10].

Pharmacoynamics

The activity of cytarabine is thought to be related to concentrations of the active metabolite araCTP in leukaemic cells[9]. However, this has not been substantiated in clinical studies[5]. Saturation of araCTP formation may occur at high doses[10].
Children appear to tolerate higher doses of Cytarabine than adults.[2]

References

1. Cancer Chemotherapy Handbook Dorr RT and Van Hoff DD 2nd Edition Appleton and Lange 1994
2. ABPI Data Sheet Compendium 1998-99
3. Cytotoxics Handbook 3rd Edition Allwood M, Stanley A and Wright P Radcliffe Medical Press 1997
4. Handbook of Injectable Drugs 10th Edition Trissel ,L ASHP 1998
5. Pharmacokinetics of mitoxantrone, etoposide and cytosine arabinoside in leukemic cells during treatment of acute myelogenous leukemia – relationship to treatment outcome and bone marrow toxicity.
   Gruber A. et al. Leukemia Research 19: 757-761 (1995).
6. Biochemical pharmacology of high dose 1-b-D-arabinofuranosylcytosine in childhood acute leukemia. Avramis V.I. et al. Cancer Research 47: 6786-6792 (1987).
7. Clinical Pharmacology of 1-b-D-arabinofuranosyl cytosine.
   Ho W.D.H. et al. Clinical Pharmacology and Therapeutics 12: 944-954 (1971).
8. Pharmacokinetics of cytosine arabinoside in patients with acute myeloid leukaemia.
   Harris A.L. et al. British Journal of Clinical Pharmacology 8: 219-227 (1979).

9. Phase I-II clinical and pharmacologic studies of high-dose cytosine arabinoside in refractory leukemia.
Kantarjian H.M. et al. American Journal of Medicine 81: 387-394 (1986).
10. Plasma and cerebrospinal fluid pharmacokinetics of 1-b-D-arabinofuranosylcytosine and 1-b-D-arabinofuranosyluracil following the repeated intravenous administration of high- and intermediate-dose 1-b-D-arabinofuranosylcytosine.
Damon L.E. et al. Cancer Research 51: 4141-4145 (1991).
11. Pharmacology studies of 1-b-D-arabinofuranosylcytosine in pediatric patients with leukemia and lymphoma after a biochemically optimal regimen of loading bolus plus continuous infusion of the drug. Avramis V.I. et al. Cancer Research49: 241-247 (1989).

23.2.5 DACARBAZINE

SECTION 1

Alternative names

5-(3,3-dimethyl-1-trazeno)imidazole-4-carboxamide. (DTIC)

Mechanism of action

- Purine analogue, inhibits purine synthesis
- Atypical alkylator
- Methylates nucleic acids
- Requires metabolic activation

Considerations prior to administration

Dose modification required in hepatic or renal abnormality

Adverse effects

Early

Common
- Myelosupression
- GI upset (nausea and vomiting most common)

Occasional:
- Flu like syndrome

Rare
- Alopecia
- Cutaneous hypersensitivity
- Hepatotoxicity
- Hepatic vein thrombosis

Late

Common
None

Occasional
- Photosensitivity

Rare
- Carcinogenic
- Teratogenic

SECTION 2

Recommended route
Intravenous

Caution

Protect from light

Administration

Intravenous bolus or infusion
Intra arterial

Dose/schedule

Interactions

Half life increases when corynebacterium parvum immunotherapy used
Interleukin-2 has been reported to increase the clearance of Dacarbazine[2].

Overdose

No recommendations

SECTION 3

Dilution specification and stability[6]

Dilute 100mg or 200 mg vials in 9.9 or 19.7 ml sterile water respectively
May be diluted in up to 250 ml of dextrose 5% or sodium chloride 0.9%.

Pharmacokinetics

With conventional doses (2.65-6.85 mg/kg) the terminal half-life of DTIC is 41 min, with a volume of distribution of 0.6 l/kg and clearance of 0.9 lk/kg/h[3].

Half-life values of 0.2 and 2.0 hr have been reported following intravenous infusion of high dose (850-1980 mg/m^2) DTIC, with a volume of distribution of 0.4 l/kg. Clearance was 0.6 l/kg/h, with 11 to 63% of the dose recovered unchanged in the urine[1].
Oral absorption is slow and incomplete[4].

Pharmacodynamics

There are no data available, but activity may depend upon metabolism[5].

References

1. Buesa JM and Urrechaga E: Clinical pharmacokinetics of high dose DTIC. Cancer Chemotherapy and Pharmacology, 1991, 28, 475-479.

2. Chabot GG et al. Alteration of dacarbazine pharmacokinetics after interleukin-2 administration in melanoma patients. Cancer Chemotherapy and Pharmacology, 1990, 27, 157-160.

3. Breithaupt H. et al. Pharmacokinetics of dacarbazine (DTIC) and its metabolite 5-aminoimidazole-4-carboxamide (AIC) following different dose schedules.Cancer Chemotherapy and Pharmacology, 1982, 9, 103-109.

4. Loo TL et al. Pharmacologic studies of the antitumor agent 5-(dimethyltriazeno)imidazole-4-carboxamide. Cancer Research, 1968, 28, 2448-2453.

5. Loo TL et al. Mechanism of action and pharmacology studies with DTIC (NSC-45388). Cancer Treatment Reports, 1976, 60, 149-152.

6. Trissel LA Handbook of Injectable Drugs 8th Edition ASHP Bethesda, Maryland , USA

23.2.6 DOXORUBICIN

SECTION ONE

Alternative Names

Adriamycin hydrochloride,
14- hydroxydaunorubicin
3- Hydroxyacetyldaunorubicin [1]

Mechanism of action

Doxorubicin is an anthracycline antibiotic active in all phases of the cell cycle with maximal activity in S phase. It has several modes of action including intercalation to DNA double helix, topoisomerase II mediated DNA damage, production of oxygen- free radicals which cause damage to DNA and cell membranes, and complex formation with iron or copper via the hydroquinone moieties [2]. Iron doxorubicin complexes may contribute to cardiotoxicity by toxic free radical generation [3].

Considerations prior to administration

Well established robust venous access. A central venous catheter or indwelling vascular access port is recommended for prolonged infusions to reduce the risk of extravasation [2] .
Full blood count
Liver function tests
Cardiac function
Creatinine, urea, electrolytes

Adverse effects

Common
- Nausea and Vomiting
- Myelosuppression
- Alopecia
- Mucositis
- Red urine
- Diarrhoea
- Severe tissue damage if extravasated

Occasional
- Increased bilirubin
- Cardiomyopathy

Rare
- Hepatocellular necrosis
- Hyperpigmentation of skin, mucous membranes, nails
- Anaphylaxis, chills, fever
- Renal damage
- Drowsiness
- Conjunctivitis

SECTION TWO

Recommended routes
Intravenous

CAUTION
A baseline echocardiogram must be done prior to treatment. This should be repeated prior to alternate courses of doxorubicin up to a total cumulative dose of 300mg/m2 , and before each course thereafter [4,5] . If the left ventricular shortening fraction (SF) is < 29% to 30% (depending on precise echocardiographic methodology [4,5]) temporary withdrawal of doxorubicin therapy should be considered [5]. If subsequent testing shows an improvement in SF consider reintroducing doxorubicin. A fall in SF by an absolute value of > 10 percentile units, or a rate of fall of > 2 to 3 percentile units per 100mg/m2, despite an SF > 29% to 30%, may also represent significant deterioration [5,6]. If the patients hepatic function is significantly impaired, doxorubicin dosage reduction should be considered [7,8,33,35] .

Dose /schedule

Due to the vesicant properties of doxorubicin it is strongly recommended that doxorubicin is given through a central venous line. For ease of administration, to reduce cardiotoxicity, and

allow haematological recovery the following schedule is recommended:
administration of doxorubicin as a 6 hour infusion (in dextrose 5% or sodium chloride 0.9%)[9,10] .
Aministration as a single daily dose or divided doses fractionated over several days [7.]
Cmulative dose of 450mg/m2 to 550mg/m2, exceeded with extreme caution [7].

Intra-arterial administration has been investigated [7].

Interactions

Doxorubicin may interact with the following:-

- ICRF-187(Dexrazone)- reduce cardiotoxicity [11,12]
- Cardiac irradiation - increased cardiac damage
- Actinomycin, mithramycin- cardiomyopathy [13]
- Mercaptopurine- increased hepatotoxicity [13]
- Mitomycin-increased incidence of late congestive heart failure[13]
- Barbiturates - increased doxorubicin elimination [2,13]
- Verapamil- increased doxorubicin serum levels, reversal of doxorubicin resistance, reduced absorption of verapamil [2,13]
- Propranolol- increased cardiotoxicity [2,13]
- Tamoxifen- reduced doxorubicin clearance, modulation of doxorubicin resistance [2,13]
- Cyclosporin- increased doxorubicin serum levels and myelotoxicity, modulation of doxorubicin resistance [2,13]
- Carbamazepine, phenytoin, sodium valproate- altered anticonvulsant serum levels [13]
- Warfarin- increased warfarin effect [2,13]
- Cimetidine, ranitidine- increased doxorubicin toxicity [2]
- Interferon alfa-altered doxorubicin disposition, doxorubicin dose reduction [2]
- Paclitaxel-increased toxicity of doxorubicin, if administered after paclitaxel [32]
- Cyclophosphamide - increases AUC and reduces clearance of parent drug and active metabolite [38]

The clinical relevance of many of these interactions is unclear.

Overdose

Doxorubicin overdosage can prove fatal. Manifestations of overdose may include acute myocardial degeneration, severe myelosuppression and delayed cardiac failure. There is no specific antidote. Symptomatic supportive measures should be implemented [7,8.]

SECTION THREE

Dilution specification

Preparation

Doxorubicin supplied in :-

(i) Vials containing 10mg and 50mg freeze dried powder. Reconstitute with water for injection or sodium chloride 0.9% injection adding 5ml to the 10mg vial and 25ml to the 50mg vial to give a 2mg/ml solution [7,8.]

(ii) Vials containing 10mg and 50mg as a 2mg/ml solution in sodium chloride 0.9% [14.]

Dilution

Doxorubicin is compatible with sodium chloride 0.9% and dextrose 5% [7,8,14.]

Stability

A large body of information is available on the stability of doxorubicin in solution [15.] Doxorubicin is compatible with polypropylene [15,16,] polyvinyl chloride (PVC) [15,16] glass [17,] ethylene vinylacetate (EVA)[18] and polyisoprene [19] containers. Solutions should be protected from light during storage and administration unless the solution is freshly prepared and the concentration is greater than or equal to 0.5mg/ml [20.] In addition [15]. Doxorubicin appears to be chemically stable in polypropylene, PVC, or EVA containers for at least 7 days, when refrigerated or stored at room temperature, protected from light, and diluted in the following:

> sodium chloride 0.9% at concentrations of 0.1mg/ml to 2mg/ml [16,18-23] : dextrose 5% at concentrations of 0.1mg/ml to 1.25mg/ml adsorptive losses which may be pronounced at low concentrations can be prevented by storage in polypropylene [16] or when doxorubicin is used at concentrations of at least 0.5mg/ml [16,18,24]. In addition, at least a 7 day expiry can be given to doxorubicin reconstituted with water for injection to a concentration of 2mg/ml, stored in polypropylene syringes at 4oC [16.]

Pharmacokinetics

The pharmacokinetics of doxorubicin in paediatric patients have been characterised in children, but the large number of protocols and different disease types make it difficult to produce representative summaries. Volume of distribution varies from 20-28 l/kg (approx. 609 l/m2) [33 .] Anthracyclines are ionised and have low lipid solubility and so do not easily cross the blood-brain barrier. Doxorubicin is metabolised to doxorubicinol, an active metabolite which may occur at higher concentrations than parent drug in plasma [33.] Excretion of drug and metabolites is via further metabolism and/or biliary excretion, with only 5 to 15% excreted by the kidney [33]. Elimination is triphasic, with no effect of age on clearance when normalised for surface area [33]. Terminal half-life is 14 to 50 hours, with clearance varying from 267 to 1443 ml/min/m2 [33]. Relatively little difference in pharmacokinetics has been observed in infants, but there was a trend to lower systemic clearance than in older children (790 vs. 1500 ml/min/m2, p=0.07)[34]. Dosage adjustment has been recommended in patients with impaired hepatic function [33,35], although this has not been validated in paediatric patients.

Pharmacodynamics

Although some data exists regarding the influence of plasma concentrations on the therapeutic and toxic effects of doxorubicin, little of this has been obtained in paediatric patients [33,36,37].

Additional Information

A number of ways to reduce cardiotoxicity have been suggested but the use of an alternative dosage schedule of weekly rather than 3 weekly [25,26], prolonged infusion schedules [9,10,27-29], adjuvant cardioprotective agents (e.g. ICRF-187) [11,12] or the administration of doxorubicin in a liposome formulation [30], whilst increasingly advocated are not yet of proven utility.
The dose/schedule in this document is recommended as a reasonable compromise.
Due to the risk of cardiac abnormalities developing many years after doxorubicin therapy, long term cardiac follow up is recommended [31,39].

References

1. ***Reynolds JF***. (1996).Martindale. The extra pharmacopoeia. 31st edition. The Pharmaceutical Press.
2. ***Dorr, RT***. et al. (1994). Cancer chemotherapy handbook. 2nd edition Appleton and Lange.
3. ***Hale, JP. Lewis, IJ***. (1994). Anthracyclines: cardiotoxicity and its prevention. Arch. Dis Child.71, 457-462.
4. ***Bu'Lock, FA***. (1995). Anthracyclines and the heart. Primary Cardiology. 21(9),5-17
5. ***Steinherz, LJ.*** et al. (1992) Guidelines for cardiac monitoring of children during and after anthracycline therapy: Report of the Cardiology Committee of the Children's Cancer Study Group. Articles. 89 (5), 942-949.
6. ***Bu'Lock, FA.*** et al. (1996). Early identification of anthracycline cardiomyopathy: possibilities and implications. Arch Dis Child. 75, 416-422.
7. ***Pharmacia & Upjohn***: Data Sheet Doxorubicin Rapid Dissolution
8. ***Faulding Pharmaceuticals Ltd*** (David Bull Laboratories): Data Sheet Doxorubicin hydrochloride
9. ***Speyer, JL.*** et al. (1985). Prospective evaluation of cardiotoxicity during a six-hour doxorubicin infusion regimen in women with adenocarcinoma of the breast. Am J Med. 78,555-563
10. ***Shapira, J.*** et al. (1990). Reduced cardiotoxicity of doxorubicin by a 6-hour infusion regimen: A prospective randomised evaluation. Cancer. 6(4), 870-873
11. ***Bu'Lock, FA.*** et al. (1993). Cardioprotection by ICRF187 against high dose anthracycline toxicity in children with malignant disease. Br Heart J. 70, 185-188.
12. ***Wexler, L***. et al. (1996). Randomised trial of the cardioprotective agent ICRF-187 in paediatric sarcoma patients treated with doxorubicin. J Clin Oncol. 14(2), 362-372.
13. ***Stockley, I***. (1996) Drug Interactions. 4th edition. The Pharmaceutical Press. 14. ***Pharmacia & Upjohn*** : Data Sheet Doxorubicin Solution for Injection.
15. ***Allwood, M***. et al.(1993).The cytotoxic handbook. 2nd edn.
16. ***Wood, MJ***. et al (1990). Stability of doxorubicin, daunorubicin, and epirubicin in plastic syringes and PVC minibags. J Clin Pharm Ther. 15, 279- 289.
17. ***Hoffman, DM***. et al.(1979). Stability of refrigerated and frozen solutions of doxorubicin hydrochloride. Am. J. Hosp. Pharm. 36, 1536-1538
18. ***Rochard, EB.*** et al. (1992). Stability of fluorouracil, cytarabine, or doxorubic hydrochloride in ethylene vinylacetate portable infusion-pump reservoirs. Am J Hosp. Pharm. 49, 619-623.

19. ***Nyhammar, EK***.et al. (1996). Stability of doxorubicin hydrochloride and vincristine sulphate in two portable infusion pump reservoirs. Am J Health-syst Pharm. 53, 1171-1173.
20. ***Wood, MJ***. et al (1990). Photodegradation of doxorubicin, daunorubicin, and epirubicin measured by high-performance liquid chromatography. J Clin Pharm Ther. 15, 291-300
21. ***Stiles, ML. Allen, LV***. (1991). Stability of doxorubicin hydrochloride portable pump reservoirs. Am J Hosp Pharm. 48, 1976-1977.
22. ***Walker, S.*** et al.(1991). Doxorubicin stability in syringes and glass vials and evaluation of chemical contamination. Can J Hosp Pharm. 44, 71-78, 88 (Apr).
23. ***Hiller, C***. Welsh pharmaceutical services (1994) UK Stability database.
24. Benvenuto, JA. et al (1981). Stability and compatibility of antitumour agents in glass and plastic containers. Am J Hosp Pharm. 38, 1914-1918.
25. ***Torti, FM***. et al. (1983). Reduced cardiotoxicity of doxorubicin delivered on a weekly schedule: assessment by endomyocardial biopsy. Ann Intern Med. 99,745-749.
26. ***Von Hoff, DD.*** (1979). Risk factors for doxorubicin-induced congestive heart failure. Ann Intern Med. 91,710-716.
27. ***Legha, SS***. et al. (1982). Reduction of doxorubicin cardiotoxicity by prolonged continuous intravenous infusion. Ann Intern Med. 96,133-139.
28. ***Ortega, JA***. et al. (1991). Effective treatment of unresectable or metastatic hepatoblastoma with cisplatin and continuous infusion doxorubicin chemotherapy: A report from the Children's Cancer Study Group. J Clin Oncl. 9(12), 2167-2176.
29. ***Lippens, RJ***. (1987). Tolerance of 24 hour infusions or low and high dose bolus injections of adriamycin in children. Paediatr Haematol Oncol. 4(3), 189-197
30. ***Uziely, B.*** et al.(1995) Liposomal doxorubicin: antitumour activity and unique toxicity's during two complementary phase I studies. J Clin Onc. 13,1777- 1785
31. ***Steinherz, LJ***. et al. (1991). Cardiac toxicity 4 to 20 years after completing anthracycline therapy. JAMA. 12,1672-1677

32 ***Gianni L, Vigano L. Locatelli A ,Capri G,Giani A, Tarenzi E., and Bonadonna G.*** (1997). Human pharmacokinetic characterization and in vitro study of the interaction between doxorubicin and paclitaxel in patients with breast cancer. J Clin Oncol 15(5): 1906-1915.

33. ***Crom, WR*** et al. (1987). Pharmacokinetics of anticancer drugs in children. Clin Pharmacokin. 12, 168-213.
34. ***McLeod, HL*** et al. (1992). Disposition of antineoplastic agents in the very young child. Br J Cancer. 66 suppl XVIII, S23-S29.
35. ***Brenner DE*** et al. (1983). Acute doxorubicin toxicity: Relationship to pre-treatment liver function, response and pharmacokinetics in patients with acute nonlymphocytic leukaemia. Cancer 53, 1042-1048.
36. ***Bielack SS*** et al. (1989). Doxorubicin: Effect of different schedules on toxicity and anti-tumour efficacy. Eur J Cancer Clin Oncol. 25, 873-882.
37. ***Preisler HD*** et al. (1984). Relationship between plasma adriamycin levels and the outcome of remission induction therapy for acute non lymphocytic leukaemia. Cancer Chemother Pharmacol. 12, 125-130.

38 ***Evans WE*** et al. (1980). Adriamycin pharmacokinetics in children. Proc. Am. Assoc. Cancer Res. Abs 705.

39 ***Kissen GKW, Wallace WHB***.(1995).Therapy based guidelines for long term follow up for children treated for cancer. Publication by Pharmacia on behalf of the late effects group at the UKCCSG.

23.2.7 ETOPOSIDE

SECTION ONE

Alternative names

- VP16

- VEPESID

Mechanisms of action:
Acts by inhibition of Topoisomerase II which results in DNA strand breakage

Considerations prior to administration
- FBC
- Renal function
- Liver function

Adverse Effects[1]
Common
- Alopecia
- Myelosuppression

Occasional
- Nausea/vomiting

Rare
- Anaphylactic reactions
- Fever
- Hypotensive reactions
- Headache
- Pruritus
- Pigmentation
- Mucositis
- Second tumours

SECTION TWO

Recommended routes

1) Intravenous

Administration

- Ampoules 100mg in 5ml
- Dilute to a concentration of 0.4mg/ml in 0.9% Sodium Chloride Injection, give as an IV infusion over 1 to 4 hours[2]. One hour infusion is recommended in order to avoid problems with "line time" during complex chemotherapy regimens.
- Data sheet recommends administration over at least 30 minutes, to avoid hypotensive reaction.[3]
- Due to potential solubility problems care should be taken when mixing with other agents[2].

Dose/schedule

Daily administration has greater anti-tumour efficacy than an equivalent dose given over 24 hours with comparable toxicity + AUC e.g. 100mg/m^2/day for 5 days versus 500mg/m^2 over 24 hours[5,6], although this is the subject of some debate[7].
Drug is undetectable (< 0.2microgram/ml) in plasma by 48 hours post administration in the majority of patients (important for protocols with marrow reinfusion)[8].

2) Oral
- Gelatin capsules 100mg and 50mg

- Injection can be used orally 20mg/ml (5ml amp)

Administration

- Short elimination $t_{½}$ suggests twice daily dose
- Prolonged administration is possible but careful monitoring of haematological toxicity is necessary

Interactions (IV and Oral preparations)

- No major interactions with the possible exception of warfarin, where etoposide may displace protein bound warfarin or alter it's metabolism, leading to increased prothrombin times[1,9]
- Cisplatin may reduce the clearance of etoposide[10].
- For the related drug tenopiside and possibly for etoposide as well, co-administration of anticonvulsants (Phenytoin or Phenobarbitone) can also result in increase clearance[9].

Overdosage (IV and Oral preparations)

- Full supportive measures, including the use of growth factors should be considered.
- Dialysis and haemofiltration are not effective as etoposide is highly plasma protein bound[11].

SECTION THREE

Dilution specification & stability

Intravenous

- Manufacturers recommend diluting to 0.25mg/ml, however a dilution of 0.4mg/ml is stable at room temperature for 96 hours (may precipitate if refrigerated).
- Only licensed in UK for administration in Normal Saline.
- Poor water solubility therefore formulated in polyethylene glycol solubilising agent which dissolves plastics.
- Use nylon filters + PVC bags or glass bottles

Oral

- IV preparation is stable for 28 days in glass vials.

Pharmacokinetics:

Intravenous

- Medium (range) clearance 26 (14-54) ml/min/m^2, $t_{1/2B}$ 132 (87-673) mins [8,12,13].
- AUC - 3.9 (1.8 - 7.3)mg/ml.min/100mg/m^2.
- Children tend to have shorter elimination half life than adults, possibly because of better renal function and hence higher renal clearance. Clearance can be increased in hypoalbuminaemic patients leading to a reduced AUC. However, since this is associated with a reduction in protein binding, haematologic toxicity may be greater in such patients[14].
- Clearance is independent of age[8,15] and dose[8] (90 - 25Omg/m^2) but is more variable at doses > 100mg/m^2 and therapeutic drug monitoring may be indicated.

Oral

- Absorption of capsules differs from IV preparation taken orally[9].
- Approximate equivalence ratios:

**IV: Oral capsules**	1:2
**IV: Oral injection**	1:1.4

Oral capsules: Oral injections 1:0.7

- Larger oral doses over 200mg/m^2 are less effectively absorbed than lower doses.
- Medium bioavailability is 48% at dose > 300mg, but 86% at a dose of 100mg. This suggests a saturable step in oral absorption[16].
- Oral bioavailability varies, with big inter and intra patient variability[17,18].
- Bioavailability is not affected by food.

Pharmacodynamics:

Correlation between AUC and/or- steady state levels and haematological toxicity has been seen in adult patients[19]. There is a closer pharmacodynamic relationship with unbound rather than with total concentrations of etoposide in plasma[20,21].

REFERENCES

1. Cancer Chemotherapy Handbook 2nd Edition Eds RT Dorr & DD Van Hoff Publ Appleton and Lange 1994
2. Vepesid pharmaceuticals ref KF/KD/0005K Jan 1993
Bristol Myers Squibb Technical Services
3. Data Sheet Compendium 1995-96 ABPI Data Pharm Publications Ltd
4. British Journal of Cancer 1989 60:458 The stability of the intravenous preparation of etoposide in isotonic fluids. S Joel et al
5. J Clin Oncol 1989 7:1333-1340 A randomized trial to evaluate the effect of schedule on the activity of etoposide in small-cell lung cancer. M Slevin et al
6. J Clin Oncol 1994 12:1427-1435 A randomized trial of two etoposide schedules in small-cell lung cancer: the influence of pharmacokinetics on efficacy and toxicity. P Clark et al.
7. Europ J Cancer 1996 32A:2291-2297 Etoposide for the treatment of paediatric tumours: What is the best way to give it? SP Lowis_SP and DR Newell.
8. Cancer Res 1993 53:4881-4889 Etoposide pharmacokinetics in children: The development and prospective validation of a dosing equation. SP Lowis et al.
9. Clinical Pharmacokin 1987 12:223-252 The clinical pharmacology of etoposide and teniposide. PJ Clarke and ML Slevin.
10. Clin Pharmacol Ther 1994 56:503-511 Etoposide pharmacokinetics and pharmacodynamics after acute and chronic exposure to cisplatin. M Relling M et al.
11. Br J Cancer 1996 73: 776-780 Pharmacokinetically guided dosing of carboplatin and etoposide during peritoneal dialysis and haemodialysis. MW English et al.
12. Cancer Chemother Pharmacol 1982 7:147-150 Pharmacokinetics of teniposide (VM26) and etoposide (VP16-213) in children with cancer. WE Evans et al.
13. Cancer Res 1984 44:3109-3133 Pharmacokinetics of etoposide (VP16) in children and adolescents with refractory solid tumors. JA Sinkule et al.
14. J Natl Cancer Inst 1991 83: 1560-1564 Modeling interpatient pharmacodynamic variability of etoposide. R Mick and MJ Ratain.
15. J Clin Oncol 1995 13: 2954-2960 Steady-state levels and bone marrow toxicity of etoposide in children and infants: does etoposide require age-dependent dose calculation ? J Boos et al.
16. Cancer Chemother Pharmacol 1989 24: 329-331 The effect of dose on the bioavailability of oral etoposide: confirmation of a clinically relevant observation. ML Slevin et al.
17. Cancer Treatment Reports 1985 69: 269-273 Bioavailability, pharmacokinetics and clincial effects of an oral preparation of etoposide. DJ Stewart et al.
18. J Clin Oncol 1993 11: 374-377 Bioavailability of low-dose oral etoposide. KR Hande et al.
19. J Natl Cancer Inst 1991 83: 1560-1564 Modeling interpatient pharmacodynamic variability of etoposide. R Mick and MJ Ratain.

20 Clin Pharmacol Ther 1991 50: 385-393 Relation of systemic exposure to unbound etoposide and hematologic toxicity. CF Stewart et al.
21 J Clin Oncol 1996 14: 257-267 Predicting etoposide toxicity: telationship to organ function and protein binding. SP Joel et al.

23.2.8 IFOSFAMIDE

SECTION ONE
Alternative names

- Mitoxana (Proprietary Name)

Mechanism of action

Ifosfamide is a pro-drug which is activated by microsomal enzymes, mainly in the liver, producing a series of metabolites. The primary anti-tumour metabolite, ifosphosphoramide mustard is thought to exert its cytotoxic effect by producing interstrand and interstrand DNA crosslinks

Considerations prior to administration
- Low serum albumin associated with increased risk of encephalopathy[19]
- Presence of large pelvic tumour (ureteric obstruction)
- Satisfactory Full Blood Count.
- Impaired renal function[20,21,22]
- Impaired liver function
- Prior cisplatin treatment may enhance renal toxicity[20,21,22]
- Urothelial damage following radiation or previous chemotherapy

Adverse Effects
Common
- Renal toxicity, particularly tubular dysfunction
- Myelosuppression
- Emesis
- Alopecia

Occasional
- Liver dysfunction
- Haemorrhagic cystitis if inadequate mesna prophylaxis

Rare
- Encephalopathy

SECTION TWO

Recommended route

Intravenous infusion over 1 hour.

Dose/schedule

Courses given approximately 3 weeks apart to allow haematological recovery.
In order to prevent urothelial toxicity, hydration and prophylactic mesna are essential with this drug.

Hydration and mesna

Ifosfamide by 1 hour infusion Intravenous hydration (with glucose/saline + potassium chloride 20 mmol/l.)*, containing **mesna** at **120%** (mg/mg) of the prescribed **daily ifosfamide dose.** Infuse this solution at a rate of 3l / m^2 / 24 hours, commencing 3 hours before the first ifosfamide dose and continuing for a **minimum** of 12 hours after completion of the last ifosfamide infusion. Recommendations are of 24 hours post hydration.

Interactions

The concomitant use of ifosfamide with anticoagulants, especially warfarin, may result in an increased anti-coagulant effect.

Overdosage

The most serious consequences are haemorrhagic cystitis, and myelosuppression. If the overdose is recognised early, intravenous hydration and diuresis, together with mesna may be beneficial in ameliorating damage to the urinary tract. Methylene blue [23] and diazepam [24] have shown some activity in reversing ifosfamide encephalopathy

SECTION THREE

Dilution's specification [26]

Ifosfamide is supplied in vials containing 1 g or 2 g.
Reconstitute in Water for Injections BP as follows:

1. 1 g vial add 12.5 ml Water for Injections BP
2. 2 g vial add 25 ml Water for Injections BP

Resultant solution of 8% (80 mg / ml) ifosfamide should not be injected directly into a peripheral vein. The solution may be:-

i) diluted to < 4% w/v solution and infused directly into the vein
ii) infused in 5% glucose, glucose / saline, or saline
iii) infused directly into a fast running infusion.

Stability

A large body of information is available on stability of ifosfamide and mesna in solution.[27,28]
Ifosfamide is stable in glucose/saline solution with added potassium* and is physically and chemically compatible with mesna (1mg/ml) in these solutions at concentrations of 1 mg/ml for 24 hours at room temperature, and 72 hours at 4°C.
* **(Glucose 2.5%, sodium chloride 0.45% with potassium 20 mmol/l)**

Pharmacokinetics

Like cyclophosphamide the pharmacokinetics of ifosfamide are complex and its pharmacologic activity resides with its metabolites. Thus, only limited information concerning the likely effect of the drug can be determined from measuring plasma concentrations of parent drug. Ifosfamide is usually given intravenously, often by continuous infusion. Oral administration is associated with an increased risk of side-effects (see below)[1], although bioavailability is reported to be high [2]. The major route of elimination is metabolism, with urine recovery of unchanged drug of up to 30% [3, 4, 5]. A high degree of both inter- and intra-subject variation in pharmacokinetics and metabolism has been reported[5, 6, 7].

The clearance of ifosfamide depends on the schedule of administration as it induces its own metabolism following continuous exposure. This usually occurs within 12 hours of the start of administration, but induction of metabolism is usually complete by 48 hours, with no further induction with longer infusions [8]. This increase in metabolism is associated with an increase in both activating (4-hydroxylation) and inactivating (dechloroethylation) routes of metabolism [6]. There is no difference in metabolism or autoinduction comparing continuous infusion or repeated bolus administration on consecutive days [9]

The following values for clearance, half-life and volume of distribution should be interpreted with regard to the schedule of administration and prior ifosfamide treatment [4, 9, 10, 11].

Half-life	1 to 7 hours
Clearance	3.0 to 8.2 $l/h/m^2$
Volume of distribution	310 to 1100 l/kg

CSF penetration of ifosfamide is almost complete [12].
As with cyclophosphamide, co-administration of carbamazepine [8], phenytoin [13, 14] and prolonged steroid treatment [15, 16] increase the clearance and metabolism of ifosfamide, but the clinical significance of this is unknown. Drugs which may inhibit ifosfamide metabolism include azole antifungal agents and nifedipine [7].

Pharmacodynamics

At present, little is known about the relationship between plasma concentrations of ifosfamide, or its metabolites, and anti-tumour or toxic effects. Encephalopathy has been associated with high levels of the chloroacetaldehyde by-product of the dechloroethylation pathway of metabolism[17]. This side-effect has also been linked with elevated dechloroethylation of the S-enantiomer of ifosfamide [18]. Particular risk factors have been identified as increasing the risk of encephalopathy and/or nephrotoxicity [19]. These include impaired renal function, prior platinum treatment [20], nephrectomy or pelvic disease, although the absence of these does not preclude the occurrence of toxicity [21, 22]. Recently, methylene blue has been suggested as an antidote to encephalopathy [23], but this treatment is still under evaluation and diazepam has also been reported to be effective [24]. Recommendations have been made for treatment termination or delay due to encephalopathy in paediatric patients [25].

References.

1. British Journal of Cancer 1989 60:258-261

A simple outpatient treatment with oral ifosfamide and oral etoposide for patients with small cell lung cancer (SCLC).
T Cerny, M Lind, N Thatcher, R Swindell and R Stout
2. Arzneimietel Forschung 1986 36:878-880
Pharmacokinetics and bioavailability of oral ifosfamide.
T Wagner and P Drings
3. Cancer Chemotherapy and Pharmacology 1991 28:455-460
Urinary excretion of the enantiomers of ifosfamide and its inactive metabolites in children.
J Boos, U Welslau, J Ritter, G Blaschke and G Schellong
4. Cancer Research 1993 53:3758-3764
Pharmacokinetics and metabolism in children of ifosfamide administered as a continuous infusion.
AV Boddy, SM Yule, R Wyllie, L Price, ADJ Pearson and JR Idle
5. British Journal of Cancer 1994 69:931-936
Metabolism of ifosfamide during a 3 day infusion.
JM Hartley, L Hansen, SJ Harland, PW Nicholson, F Pasini and RL Souhami
6. Cancer Chemotherapy and Pharmacology 1993 33:36-42
Comparative pharmacokinetics of ifosfamide, 4-hydroxyifosfamide, chloroacetaldehyde and 2- and 3-dechloroethylifosfamide in patients on fractionated intravenous ifosfamide therapy.
V Kurowski and T Wagner
7. Cancer Chemotherapy and Pharmacology 1996 In press.:Intrasubject variation in children of ifosfamide pharmacokinetics and metabolism during repeated administration.
AV Boddy, SM Yule, R Wyllie, L Price, ADJ Pearson and JR Idle
8. Cancer Chemotherapy and Pharmacology 1995 36:53-60
The kinetics of the auto-induction of ifosfamide metabolism during continuous infusion.
AV Boddy, M Cole, ADJ Pearson and JR Idle
9. European Journal of Cancer 1995 31A:985-790
Comparison of continuous infusion and bolus administration of ifosfamide in children.
AV Boddy, SM Yule, R Wyllie, L Price, ADJ Pearson and JR Idle
10. Clinical Pharmacokinetics 1994 26:439-456
Ifosfamide clinical pharmacokinetics.
T Wagner
11. Anticancer Research 1994 14:517-532
Ifosfamide metabolism and pharmacokinetics (Review).
GP Kaijser, JH Beijnen, A Bult and WJM Underberg
12. Cancer Research 1988 48:2113-2115
Cerebrospinal fluid penetration of active metabolites of cyclophosphamide and ifosfamide in rhesus monkeys.
CAS Arndt, FM Balis, CL McCully, M Colvin and DG Poplack
13. Cancer Research 1995 55:810-816
Nonlinear pharmacokinetics of cyclophosphamide in patients with metastatic breast cancer receiving high-dose chemotherapy followed by autologous bone marrow transplantation.
T-L Chen, J Passos-Coelho, D Noe, M Kennedy, K Black, O Colvin and L Grochow
14. Journal of Pharmaceutical Sciences 1995 84:292-294
Phenytoin causes a rapid increase in 6-beta-hydroxycortisol urinary excretion in humans - a putative measure of CYP3A induction.
JC Fleishaker, LK Pearson and GR Peters
15. Hepatology 1993 18:1254-1262
Regulation of human liver cytochromes P-450 in family 3A in primary and continuous culture of human hepatocytes.
EG Schuetz, JD Scheutz, SC Strom, MT Thompson, RA Fisher, DT Molowa, D Li and PS Guzelian

16. Cancer Research 1995 55:803-809
Cyclophosphamide metabolism in children.
SM Yule, AV Boddy, M Cole, L Price, R Wyllie, M Tasso, ADJ Pearson and I J.R.
17. Lancet 1986 ii:1219-1220
Dechloroethylation of ifosfamide and neurotoxicity.
MP Goren, RK Wright, CB Pratt and FE Pell
18. Lancet 1994 343:982-983
Ifosfamide stereoselective dechloroethylation and neurotoxicity.
IW Wainer, J Ducharme, CP Granvil, M Trudeau and B Leyland-Jones
19. European Journal of Cancer and Clinical Oncology 1986 22:815-819
Prediction of ifosfamide and mesna associated encephalopathy.
CA Meanwell, AE Blake, KA Kelly, L Honigsberger and G Blackledge
20. Journal of Clinical Oncology 1990 8:1399-1401
Ifosfamide neurotoxicity is related to previous cisplatin treatment for pediatric solid tumors.
CB Pratt, MP Goren, WH Meyer, B Singh and RK Dodge
21. Lancet 1987 i:987
Ifosfamide/mesna encephalopathy.
AK McCallum
22. European Journal of Cancer and Clinical Oncology 1989 25:1303-1310
Ifosfamide encephalopathy: a reappraisal.
SW Watkin, DJ Husband, JA Green and HM Warenius
23. Lancet 1994 343:763-764
Prophylaxis and reversal of ifosfamide encephalopathy with methylene-blue.
A Kupfer, C Aeschlimann, B Wermuth and T Cerny
24. Neurology 1993 43:2700-2702
Ifosfamide causes a diazepam-sensitive encephalopathy.
NA Simonian, FG Gilliam and KH Chiappa
25. Journal of Clinical Oncology 1986 4:1253-1261
Central nervous system toxicity following the treatment of paediatric patients with ifosfamide/mesna. CB Pratt, AA Green, ME Horowitz, WH Meyer, E Etcubanas, E Douglass, FA Hayes, H Thompson, J Wilimas, M Igarashi and E Kovnar
26. The Cytotoxic Handbook. II Edition. Alwood M, Wright P. 1993
27. Handbook of Injectable Drugs. IX Edition. Trissel LA. 1996
28. Ifosfamide in Paediatric Malignancy. M Phil Thesis. Ian M Sharkey. 1992

23.2.9 MELPHALAN (HIGH DOSE INTRAVENOUS)

SECTION ONE

Alternative names

- [bis(chloro-2-ethyl)amino]-L-phenylalanine
- L-phenylalanine mustard
- L-PAM
- Alkeran-proprietary name

Mechanisms of action
Alkylates DNA through the formation of reactive intermediates which attack nucleophilic sites and thus interferes with DNA replication and transcription

Considerations prior to administration

- Ensure adequate renal function (GFR>30 mls/min/1/73m^2)[1,2,3]

- Cyclophosphamide "prime" 300mg/m^2 5-9 days prior to chemotherapy [4] (only if using single agent melphalan)
- Adequate "rescue" available (marrow or stem cells) [5]
- Adequate hydration and urine output at time of administration [5,6]

Adverse effects

Common

- Nausea and vomiting (severe and immediate) [7,8]
- Profound myelosuppression

- Mucositis
- Alopecia
- Sterility (in boys)

Occasional

- Haemorrhagic diarrhoea
- Amenorrhoea
- Encephalopathy [4]
- Hypersensitivity

Rare

- Veno-occlusive disease
- Pulmonary fibrosis/pneumonitis
- Secondary leukaemia
- Dermatitis

SECTION TWO

Recommended routes

Intravenous - into a large vein.[1]

CAUTION

- Bone marrow reconstitution depends upon adequate autologous, allogenic marrow or stem cells.
- GI toxicity may be extreme.

Administration

Either:

- Inject within 30 minutes of preparation into a fast running drip

or

- Further dilute to 0.4mg/ml in N.saline and infuse over (up to) 1.5 hours

Dose/schedule

- 110mg/m^2 - 200mg/m^2 is in use
- Only used as part of an intensive high dose consolidation therapy (alone or in combination with other drugs, mIBG or TBI).
- Hydration (3L/m^2/day) must be established with good urine output prior to administration of melphalan and continued until at least 12 hours afterwards[10] (ie. 4ml/kg/min urine output pre-melphalan and for at least 2 hours post administration). Use N.saline (initially improves drug stability).
- Frusemide may be required to maintain good urine output.
- Marrow or PBSC must not be reinfused until at least 12 hours [2,5] after the end of melphalan infusion.

Interactions

- Nalidixic acid has been reported to result in severe GI toxicity in children.[7]

Overdose

- Full supportive measures.
- Melphalan is dialysable but rapidly and spontaneously breaks down into non toxic products.[11]

SECTION THREE

Dilution specifications and stability

- Store at room temperature away from direct sunlight.
- Reconstitute the 50mg freeze-dried vial using 10ml of the Solvent-Diluent provided. Shake vigorously to dissolve. The resulting solution contains 5mg in 1ml anhydrous melphalan.
- Compatible with plastic containers, administration seta and in line filters.
- Spontaneous hydrolysis is slower if diluted and given in N.saline

Pharmacokinetics

- Melphalan is cleared primarily by chemical degradation.
- Urinary excretion of intact melphalan is <20%,[11] although higher values have been reported in adults.[12]
- There is a weak relationship between GFR and melphalan clearance in adults, but this cannot be used to adjust doses.[13]

Median and Range of Mean Pharmacokinetic Parameters reported: [11,6,9,2]

Clearance	331(306-443)	ml/min/m^2
t½b	62(38-180)	min
AUC	0.30(0.22-0.33)	mg/ml.min per 100mg/m^2

In the majority of patients, plasma melphalan concentrations are below 0.1mg/ml 12h after administration at which time haematopoietic cells can be safely transplanted.

Pharmacodynamics

- In a small unconfirmed study, children with slow melphalan clearance had a better outcome than those with rapid clearance.[10]
- Test dosing and adaptive control have been successfully used to overcome inter-patient pharmacokinetic variability.[14]

REFERENCES

1. Alberts DS, Chen H-SG, Benz D & Mason NL. Effect of renal dysfunction in dogs on the disposition and marrow toxicity of melphalan. Br. J. Cancer, **43**: 330-334, 1981
2. Ardiet C, Tranchand B, Biron P, Rebattu P & Philip T. Pharmacokinetics of high-dose intravenous melphalan in children and adults with forced diuresis. Cancer Chemother. Pharmacol. **16**: 300-305, 1986
3. Taha IAK, Ahmad RA, Rogers DW et al. Pharmacokinetics of melphalan in children following high-dose intravenous injection. Cancer Chemother. Pharmacol **10**: 212-216. 1983
4. Selby P, Lopes N, Mundy J, Crofts M et al. Cyclophosphamide priming reduces intestinal damage in man following high dose melphalan chemotherapy. Cancer, **55**: 531-533, 1987
5. McElwain TJ Hedley DW, Burton G et al. Marrow autotransplantation accelerates haematological recovery in patients with malignant melanoma treated with high-dose melphalan. Br. J. Cancer, **40**: 72-80, 1979.
6. Gouyette A, Hartmann O & Pico J-L. Pharmacokinetics of high-dose melphalan in children and adults. Cancer Chemother. Pharmacol. **16**: 184-189, 1986
7. Cancer Chemotherapy Handbook, 2nd Edition. Eds: RT Dorr & DD Von Hoff, Publ: Appleton & Lange, 1994
8. Cancer Chemotherapy Principles & Practice. Eds: Chabner BA & Collins JM. Publ: JB Lippincott Co., Philadelphia. 1990
9. Kellie SJ, Kingston J, Dodge RK, Malpas JS, Pritchard J. Toxicity of high dose melphlan (HDM) with autologous bone marrow reinfusion (ABMR) in paediatric solid tumours. International Soc. of Paediatric Oncology, SIOP, XX Meeting, Trondheim, Norway. Abstract p 205, 1988
10. Cytotoxics Handbook, 2nd Edition. Eds: M Allwood & P. Wright, Publ: Radcliffe Medical Press, 1993
11. Cancer Chemother Pharmacol **15**: 263-267, 1985
    High dose melphalan in children with advanced malignant disease J Ninane et al
12. Cancer Chemother Pharmacol **22**: 3488-352, 1988
    Renal clearance and protein binding of melphalan in patients with cancer
    PA Reece et al
13. Anticancer Res **14**: 2379-2382, 1994
    Pharmacokinetics of high-dose melphalan in adults: Influence of renal function MF Kergueris et al
14. Cancer Chemother Pharmacol **23**: 95-100, 1989
    High-dose melphalan adjustment: Possibility of using a test dose. B Tranchand et al

23.2.10 PROCARBAZINE

SECTION 1

Alternative names

Natulan®
Ibenzmethyzin

N-Methylhydrazine

Mechanism of action

Suppresses mitosis by prolonging interphase[1]
Cell cycle phase specific- activity in S and G_2 phase

Considerations prior to administration
- FBC
- Renal function - decrease dose in renal impairment (serum creatinine>176micromol/l in adults) [1,2]
- LFTs - decrease dose if bilirubin>17micromol/l [2]
- Drug Interactions (see later)

Adverse effects

Common
- Myelosuppression [1,2]
- Mild nausea and vomiting (in first few days of treatment) [1]
- Diarrhoea [1]
- CNS toxicities - paraesthesia, ataxia, dizziness, headaches [1,2]
- Nightmares, depression, insomnia, nervousness, hallucinations [1]

Occasional
- Tremors, coma, convulsions [1,2]
- Influenza like syndrome [1]

Rare
- Pulmonary toxicity (probably hypersensitivity reaction) [1,2]
- Nystagmus diplopia, papilloedema, photophobia [1,2]
- Dermatologic reactions ie alopecia, pruritis, rash [1]

SECTION 2

Recommended routes
Oral
Capsules 50mg

CAUTION
Concurrent contraindicated drugs - see drug interactions

Administration
- Orally, in single or divided doses [1,3]
- Administration with food may reduce the incidence of nausea and vomiting.

Dose/ schedule

100mg/m^2 daily in divided doses for 10-14 days. (Range 50-200mg)

Interactions

Caution should be exercised with the following drugs:

CNS depressants	Procarbazine may augment the effect leading to respiratory depression [1,2]
Antidepressants	Hypertensive crisis, tremors, agitation reported Sympathomimetics - may potentially interact due to Procarbazine having weak MAOI activity [2]
Food	Procarbazine is a weak MAOI and restriction of tryamine containing foods (ie cheese, bananas, marmite, dark beer)has been recommended though the potential for hypertensive crises appears to be low [1,2,4]
Alcohol	- a disulfiram reaction may occur with alcohol and Procarbazine and CNS depression may be accentuated. [1,3]

Overdosage
The main effects are protracted myelosuppression and CNS complications.
Treat with supportive measures [2]

SECTION 3

Dilution specification and stability

Max storage temp 25^{0}C in blister packs [2]

Pharmacokinetics

Rapidly and completely absorbed from the GI tract with peak plasma levels at 0.5 to 1hour. Peak CSF levels at 0.5 to 1.5hours. Extensively metabolised in liver to several active(cytotoxic) metabolites. Over 75% of the dose is excreted in the urine. [1,2]
Plasma $t_{1/2}$ 7 mins (following iv injection) [1]

Pharmacodynamics

There are no good studies examining dose-response effects. There is no clear evidence of any correlation between the plasma concentration and its cytotoxic effects. [2]

REFERENCES

1. Cancer Chemotherapy Handbook 2nd Edition RT Dorr and DD Von Hoff Appleton and Lange
2. Therapeutic Drugs Volume 2 Edited by Sir Colin Dollery Churchill Livingstone
3. BNF No 32 Sep 1996
4. Drug Interactions 3rd Edition. Ivan Stockley Blackwell Science

23.2.11 VINBLASTINE

SECTION ONE

Alternative names:
- Velbe

Mechanisms of Action
Tubulin binding agent producing mitotic arrest.

Considerations prior toadministration

Ensure good, robust, venous access
Hepatic function.

Adverse effects

Common
- Abdominal pain
- Constipation
- Leucopenia

Occasional
- Peripheral neuropathy (mild)
- Thrombocytopenia and Anaemia

Rare
- Nausea and vomiting
- Alopecia
- Paralytic ileus

SECTION TWO

Recommended routes

By bolus injection or into the tubing of a fast running intravenous infusion.
Hydration not required.

CAUTION

Vinblastine is a highly vesicant drug, and great care must be taken to avoid extravasation

Dose/schedule

Concentration for administration 1 mg/ml.
Recommended dose: 6mg/m^2 no more frequently than every 7 days.
Maximum dose: 10mg.

Interactions
Nil known

SECTION THREE
Pharmacokinetics:

There are no published data on the clinical pharmacokinetics of vinblastine in children. In adults, vinblastine plasma clearance is greater than that of vincristine, possibly explaining the need to administer higher doses of vinblastine[1] . In adults, vinblastine pharmacokinetics are variable and elimination is due to metabolism, urinary and faecal excretion[1] . Daily administration in adults can lead to vinblastine accumulation[2] . Vinblastine pharmacokinetics may be non-linear and dependent

upon hepatic function[3] . A dose reduction has been recommended for patients with a raised serum bilirubin [4].

Pharmacodynamics

Unconfirmed studies in adults suggest that patients treated with continuous infusion vinblastine are more likely to develop leucopenia if plasma levels exceed 1ng/ml,[5] and that reduced clearance may also be associated with an improved response rate in breast cancer [6].

References

1 Anticancer Res 1992 12:1699-1716
Pharmacology, bio-analysis and pharmacokinetics of the vinca alkaloids and semi-synthetic derivatives (Review)
O van Tellingen et al

2 Anticancer Research 1992 12:655-660
Increasing peak levels of vinblastine given in repeated divided doses
SJ-P van Belle et al

3 Clin Pharmacol Ther 1987 41:61-67
Interpatient and intrapatient variability in vinblastine pharmacokinetics
MJ Ratain et al

4 Cancer Chemotherapy: Principles and Practice, Chapter 10 pp253-275, 1990, JB Lippincott, Philadelphia , RA Bender et al (BA Chabner and JM Collins, eds.)

5 Cancer Res 1986 46:4827-4830
Phase I and pharmacological study of vinblastine by prolonged continuous infusion
MJ Ratain and NJ Vogelzang

6 Cancer Res 1983 43:1405-1408
Clinical pharmacokinetics of vinblastine by continuous intravenous infusion
K Lu et al

23.2.12 VINCRISTINE

SECTION ONE

Alternative names
- Oncovin

Mechanisms of action

Tubulin binding agent producing mitotic arrest.

Considerations prior to administration

- Well established, robust, venous access.
- Hepatic function.
- Neurotoxicity
- Avoid administration at the same time as intrathecal methotrexate.

Adverse effects

Common
- Alopecia
- Abdominal pain - cramps

- Pain in jaw, bones and joints
- Constipation

Occasional
- Peripheral neuropathy (loss of deep tendon reflexes)
- Autonomic neuropathy (paralytic ileus, urinary retention)

Rare
- Leucopenia, Thrombocytopenia, Anaemia
- Nausea and vomiting
- Raised LFTs (mild and transient)
- Convulsions
- Diplopia and Photophobia

Toxicity related to individual and cumulative dose of Vincristine

SECTION TWO

Recommended routes

By bolus injection or into the tubing of a fast-running intravenous infusion.
Hydration not required.

CAUTION
Vincristine is a highly vesicant drug, and great care must be taken to avoid extravasation.

DO NOT GIVE INTRATHECALLY

Dose /schedule
- Variable
- Dose reduction may be necessary if toxicity unacceptable
- The need to limit the total vincristine dose per administration to 2mg is not supported by clinical experience in adults.[1]

Interactions

Vincristine plasma clearance can be reduced by nifedipine[2], cimetidine[3] or ranitidine[3], and increased by phenobarbitone[3]. The clinical relevance of these interactions in not clear.
Overdose

Plasmapheresis and phenobarbitone have been reported to be of value in cases of systemic vincristine overdose[4,5].

SECTION THREE

Dilution specification

- Dextrose 5%, Sodium Chloride 0.9%
- Undiluted at 1 mg/ml but at this concentration there would be increased toxicity with extravasation, therefore can be administered at lower concentrations, e.g. 0.2 mg/ml.

Stability

- Solution 1 mg/ml - 2 years in vial at 2 to 8 ^{o}C
- Lyophilised powder - 3 years at 2 to 8 ^{o}C. Chemically stable for 30 days after reconstitution when stored at 2 - 8 ^{o}C.

Pharmacokinetics

Vincristine is eliminated by hepatic metabolism and biliary excretion[6]. Clearance is variable and may be age dependent.[3,7]

Mean (±SD) pharmacokinetic parameters:[3,7]

Clearance	431±238, 482±342	ml/min/m^2
t½b	1122±1128, 823±390	min
AUC	3.2±1.8, 2.9±2.1	mg/ml.min *per* 1.4mg/m^2

In adults, but not children, vincristine neurotoxicity has been related to AUC[3,8]. Also in adults, impaired liver function has been related to reduced clearance and predisposition to neurotoxicity[9]. A dose reduction has been recommended for patients with a raised serum bilirubin[10].

References

1 Cancer Treat Rep 1987 71:229-233
Reappraisal of some dosage adjustment guidelines.
A Sulkes and JM Collins

2 Cancer 1989 64:1805-1811
Pharmacokinetics of vincristine in cancer patients treated with nifedipine
L Fedeli et al

3 J Pediatr 1994 125:642-649
Pharmacokinetics of vincristine in children and adolescents with acute lymphocytic leukaemia
WR Crom et al

4 Lancet 1992 340:185
Favorable outcome after plasmapheresis for vincristine overdose
JY Pierga et al

5 Pediatr Hematol Oncol 1991 8:171-178
Vincristine overdose: Experience with 3 patients
Kosmidis et al

6 Anticancer Res 1992 12:1699-1716
Pharmacology, bio-analysis and pharmacokinetics of the vinca alkaloids and semi-synthetic derivatives (Review)
O van Tellingen et al

7 Med Pediatric Oncol 1995 24:235-240

Vincristine disposition in children with acute lymphoblastic leukaemia
SSN de Graff et al
8 Cancer Chemother Pharmacol 1982 8:211-214
Can severe vincristine neurotoxicity be prevented?
ZR Desai et al
9 Cancer Chemother Pharmacol 1982 8:215-219
The pharmacokinetics of vincristine in man
HW van den Berg et al
10 Cancer Chemotherapy: Principles and Practice, Chapter 10 pp253-275, 1990, JB Lippincott, Philadelphia
RA Bender et al (BA Chabner and JM Collins, eds.)

23.3 ABBREVIATIONS

Abt.	(Abteilung) Division
ABVD	Chemotherapy cycle: Adriamycin, Bleomycin, Vinblastin, Dacarbazine
ADR	Adverse Drug Reaction
AE	Adverse Event
ALAT	Alanine-amino-transferase
AMG	German Drug Law
AML	Acute Myeloid Leukemia
AP	Alkaline Phosphatase
AR	Adequate response
ARA-C	Cytosin-Arabinosid, Cytarabin
ASAT	Aspartate-amino-transferase
ASHAP	Chemotherapy cycle: Adriamycin, Methylprednisolone, Cytarabin, Cisplatinum
BCNU	Carmustin
BEAM	Chemotherapy cycle: BCNU (Carmustin), Etoposide, Ara-C (Cytarabin), Melphalan
BfArM	Bundesinstitut für Arzneimittel und Medizinprodukte
B-symptoms	Systemic symptoms: unexplained fever, weight loss, night sweats
C	Celsius
°C	Degree Celsius (temperature dimension unit)
Ca	Calcium
CCLG	Children’s Cancer & Leukaemia Group (England, Scotland, Ireland and Wales)
CCS	Children`s Cancer Study
CD	Compact Disc
CEP	Chemotherapy cycle: Lomustin, Etoposide, Prednimustin
cf.	Confer
Cm	Centimeter
CMV	Cytomegalovirus
CNS	Central Nervous System
COMP	Chemotherapy cycle: Cyclophosphamide, Vincristine, Methotrexate, Prednisone
COPDAC	Chemotherapy cycle: Cyclophosphamide, Vincristine, Prednisone/Prednisolone, Dacarbazine
COPP	Chemotherapy cycle: Cyclophosphamide, Vincristine, Prednisone/Prednisolone, Procarbazine
Cp	compare
CR	Complete remission
CRF	Case Report Form

Cru	Complete remission unconfirmed
CSRA	Central staging and response assessment
CT	Computed tomography
CTC	Common Toxicity Criteria
CTCAE	Common Terminology Criteria for Adverse Events
Cum.	cumulative
CV	Curriculum Vitae
CVB	Chemotherapy cycle:
D	Dimension
2D	Two-dimensional
3D	Three-dimensional
DAL	Deutsch-Österreichische-Leukämie/Lymphom-Liga
DAL-HD	Deutsch-Österreichische-Leukämie/Lymphom-Liga-Hodgkin`s Disease
Dexa-BEAM	Chemotherapy cycle:
DD	Differential diagnosis
DFS	Disease free survival
DHAP	Chemotherapy cycle:
DICOM	Digital imaging file format
DMC	Data Monitoring Committee
DNA	Desoxyribonucleic acid
DTIC	Dacarbazine
DVH	Dose Volume Histogram
EBMT	European Bone Marrow Transplantation Society
EBV	Epstein Barr Virus
EC	Ethics Committee
ECG	Electrocardiogramme
Echo	Echocardiogramme
Echo-CG	Echocardiogramme
ED	Einzeldosis (single dose)
EEG	Electroencephalogramme
EFS	Event free survival
e.g.	Example given
E-lesions	Extranodal lesions by contiguous involvement
ENT	Ear, Nose and Throat
EORTC	European Organization for Research and Treatment of Cancer
Et al.	Et altra (and others)
Eudra-CT	European ClinicalTrials Data Base
Euro-Net	European Network
Euro-Net-PHL-C1	European Network on Pediatric Hodgkin`s Lymphoma-Classical Hodgkin-1
FAB	French-American British (classification for acute leukemias)

FDG-Pet	Fluoro-Deoxyglucose-Positron emission tomography
FFTF	Freedom from treatment failure
FF2F	Freedom from second failure
Fig.	Figure
FSH	Follicle stimulating hormone
fT4	Free Tetra-iodineThyionine
GCP	Good Clinical Practice
GCP-V	GCP-Verordnung
G-CSF	Granulocyte-Colony stimulating factor
GGT	Gamma Glutamyl transferase
GMP	Good Manufacturing Practice
GOT	Synonyme for ASAT (Aspartate-amino-transferase)
GPOH	Gesellschaft für Pädiatrische Onkologie und Hämatologie, (German Society for Paediatric Oncology and Haematology)
GPOH-HD	GPOH-HodgkinStudiengruppe (GPOH-Hodgkin`s study group)
GPT	Synonyme for ALAT (Alanine-amino-transferase)
GvH	Graft versus host
Gy	Gray
h	Hour
HCV	Hepatitis C Virus
HDR-allo	Hodgkins Disease relapse-allogeneic transplantation trial
HIV	Humane Immunodeficiency Virus
HL	Hodgkin`s Lymphoma
HLA	Human Leukocyte Antigen
HSV	Herpes simplex virus
5-HT-3 antagonist	Serotonine-antagonist
ICH	International Conference on Harmonisation
ID	Identification code
i.e.	Id est
IEP	Chemotherapy cycle: Ifosfamide, Etoposide, Prednisone/Prednisolone
IF-RT	Involved field radiotherapy
Incl.	Including
IR	Inadequate response
IRu	Inadequate response unconfirmed
ISF	Investigator Site File
ITT	Intention-to-treat
i.v.	intravenously
KCL	Potassium chlorine
Kg	Kilogram
KKSL	Koordinierungszentrum für Klinische Studien Leipzig

LDH	Lactate dehydrogenase
LESG	Late Effect Study Group
LH	Luteinizing Hormone
LH-RH	Luteinizing Hormone Releasing Hormone
LN	Lymph node
LSRA	Local staging and response assessment
m^2	Square meter
mAS	milli Ampere seconds
mg	Milligram
MHD	French Hodgkin`s Lymphoma trial name
min	Minimum
ml	Milliliter
mm	Millimeter
MRI	Magnetic Resonance Imaging
MTX	Methotrexate
NC	No change
NCI	National cancer institute
NCR	No carbon required
ND	Not done
NHL	Non Hodgkin’s lymphoma
Non-GPOH	Not belonging to GPOH group
OEPA	Chemotherapy cycle: Vincristine, Etoposide, Prednisone, Adriamycine
OE*PA	Chemotherapy cycle: Vincristine, Etoposide (20% increased dose), Prednisone/Prednisolone, Adriamycine
OPA-COMP	Chemotherapy cycle: Vincristine, Prednisone, Adriamycine - Cyclophosphamide, Vincristine, Methotrexate, Prednisone
OPPA	Chemotherapy cycle: Vincristine, Procarbazine, Prednisone, Adriamycine
OPPA_COPP	Chemotherapy cycle: consisting of OPPA and COPP
OS	Overall survival
PCR	Polymerase Chain Reaction
PD	Progressive disease
PET	Positron emission tomography
PFS	Progression free survival
PHL-C1	Paediatric Hodgkin`s Lymphoma-Classical Hodgkin`s Lymphoma-1
p.i.	Per injectionem
PIL	Patient identification list
p.o.	Per os (by mouth)
PP	Per-protocol
PR	Partial remission
Prog.	Progression
PTV	Primary target volume

11q23	Cytogenetic abnormality; fusion chromosome
RCT	Randomized controlled trial
Resid.	Residual, residuum
Resp.	Respective(ly)
RG	Response group
RNA	Ribonucleic acid
SAE	Serious Adverse Event
SAR	Serious Adverse Reaction
SCT	Stem cell transplantation
SD	Standard deviation
SE	Standard error
SIAD	Syndrome of inadequate
SmPC	Summary of medicinal Product Characteristics
Sono	Sonography, ultrasound
SOP	Standard Operating Procedures
SRA	Staging and response assessment
SST	Secondary solid tumour
STAR	strategy for treatment adapted to response
ST-HD	Salvagetherapy-Hodgkin`s disease
T2-STIR	Fat saturated T2-weighted spin echo sequence (MRI)
SUSAR	Suspected Unexpected Serious Adverse Drug Reaction
T1-Flash	T1-weighted gradient echo sequence (MRI)
TG	Therapy group
T2-TIRM	Fat saturated T2-weighted spin echo sequence (MRI)
TMF	Trial Master File
TOX	Toxicity
T1-SE	T1-weighted spin echo sequence (MRI)
TSH	Thyroid stimulating hormone
TTT	Therapy titration study
UICC	Unité Internationale contre le Cancre
UK	United Kingdom
VBVP	Chemotherapy cycle: Vinblastin, Bleomycine, Prednisone, Etoposide
VCR	Vincristine
VEEP	Chemotherapy cycle: Vincristine, Etoposide, Epirubicine, Prednisone
VP-16	Etoposide
Vs.	versus
WB-MRI	Whole body MRI
WHO	World Health Organisation
VZV	Varicella zoster Virus
X-ray	Two-dimensional radiogramme

23.4 GPOH-HD HODGKIN'S LYMPHOMA WORKING GROUP

Members of the GPOH-HD Committee (status quo December 2011):

Dr. Bergsträsser (paediatrician, Zürich, Switzerland)	eva.bergstraesser@kispi.unizh.ch
Prof. Dr. A. Engert (medical haematologist, Cologne)	a.engert@uni-koeln.de
Dr. Brosteanu (biometrician, Leipzig)	oana.brosteanu@kksl.uni-leipzig.de
Dr. T. Bernig (paediatrician, Halle)	toralf.bernig@uk-halle.de
Prof. Dr. Burdach (paediatrician, Munich)	stefan.burdach@lrz.tu-muenchen.de
PD Dr. Claviez (paediatrician, Kiel)	a.claviez@pediatrics.uni-kiel.de
Prof. Dr. Karin Dieckmann (radiotherapist, Wien)	Karin.dieckmann@meduniwien.ac.at
Dr. Dörffel (paediatrician, Berlin)	wdoerffel@berlin.helios-kliniken.de
Dr. Hasenclever (biometrician, Leipzig)	hasenclever@imise.uni-leipzig.de
Prof. Dr. Spielmann (radiology, Halle)	rolf-peter.spielmann@medizin.uni-halle.de
Dr. Gabriele Escherich (paediatrician, Hamburg)	escheric@uke.uni-hamburg.de
Prof. Dr. Jürgens (paediatrician, Münster)	jurgh@uni-muenster.de
Prof. Dr. Vordermark (radiotherapist, Halle)	dirk.vordermark@medizin.uni-halle.de
Prof. Dr. Körholz (paediatrician, Halle)	dieter.koerholz@medizin.uni-halle.de
Prof. Dr. Kluge (nuclear physician, Leipzig)	regine.kluge@medizin.uni-leipzig.de
Prof. Dr. Lehrnbecher (paediatrician; Frankfurt)	thomas_lehrnbecher@yahoo.com
Dr. Heike Lüders (physicist, Berlin)	hlueders@berlin.helios-kliniken.de
Dr. Georg Mann (paediatrician, Vienna, Austria)	georg.mann@stanna.at
Dr. Marciniak (radiologist, Berlin)	hmarciniak@berlin.helios-kliniken.de
Prof. Dr. Mauz-Körholz (paediatrician, Halle)	christine.mauz-koerholz@medizin.uni-halle.de
Prof. Dr. Pötter (radiotherapist; Vienna, Austria)	richard.poetter@univie.ac.at
Dr. Ursula Rühl (radiotherapist, Berlin)	ruehl_berlin@web.de
Prof. Dr. Sabri (nuclear physician, Leipzig)	sabri@medizin.uni-leipzig.de
Dr. Scheel-Walter (paediatrician, Tübingen)	hgscheel@med.uni-tuebingen.de
Prof. Dr. G. Schellong (paediatrician, Münster)	schellon@uni-muenster.de
Prof. Dr. Schlegel (paediatrician, Würzburg)	schlegel@mail.uni-wuerzburg.de
Prof. Dr. Sykora (paediatrician, Hannover)	sykora.karl-walter@mh-hannover.de
Prof. Dr. Feller (pathologist, Lübeck)	ac.feller@uksh.de
Dr. Lutz Wickmann (paediatrician, Berlin)	lwickmann@berlin.helios-kliniken.de
Prof. Dr. Willich (radiotherapist, Münster)	willich@uni-muenster.de

23.5 NCRI HODGKIN'S LYMPHOMA WORKING GROUP

(status quo December 2006):

W. Hamish Wallace	Edinburgh	Oncology	Chairman
Martin Hewitt	Nottingham	Oncology	
Ananth Shankar	Royal London/Middlesex	Oncology	
Laurence Abernethy	Liverpool	Radiology	
Mike Williams	Cambridge	Radiotherapy	
Helen Lucraft	Newcastle	Radiotherapy	
Eve Gallop-Evans	Cardiff	Radiotherapy	
Keith McCarthy	Cheltenham	Pathology	
Ken MacLennan	Leeds	Pathology	
Stephen Daw	Middlesex	Oncology	
Georgina Hall	Oxford	Haematology	
Malcolm Taylor	Manchester	Biology	
Elizabeth Chalmers	Glasgow	Haematology	
Jo Begent	Great Ormond Street	Oncologist/Trainee	
Caroline Bateman	Royal Marsden	Trainee	
Janis Hayward	CRCTU	Trial Coordinator	

23.6 SFCE HODGKIN'S LYMPHOMA WORKING GROUP

Alain Robert	robert.a@chu-toulouse.fr
Andre Baruchel	andre.baruchel@sls.ap-hop-paris.fr
Carrie Christian	carrie@lyon.fnclcc.fr
Claudine Schmitt	c.schmitt@chu-nancy.fr
Christine Edan	christine.edan@wanadoo.fr
Francoise Montravers	francoise.montravers@tnn.aphp.fr
Gérard Michel	gmichel@ap-hm.fr
Guy Leverger	guy.leverger@trs.ap-hop-paris.fr
Helene Pacquement	helene.pacquement@curie.net
JL Habrand	habrand@igr.fr
Anne Lambilliotte	a-lambilliotte@chru-lille.fr
Nathalie Aladjidi	n aladjidi@hotmail.com
Odile Oberlin	oberlin@igr.fr
Schell Mathias	schell@lyon.fnclcc.fr
Sylvie.Helfre	sylvie.helfre@curie.net
Thierry Leblanc	thierry.leblanc@sls.ap-hop-paris.fr
Yves Perel	yves.perel@chu-bordeaux.fr

23.7 PPLLSG HODGKIN'S LYMPHOMA WORKING GROUP

Walentyna Balwierz	balwierz@mp.pl
Jerzy Kowalczyk	jkowalcz@dsk.lublin.pl
Alicja Chybicka	klin@pedhemat.am.wroc.pl
Jacek. Wachowiak	jacek.wachowiak@plusnet.pl
Danuta. Sonta-Jakimczyk	edziubek@sk1.zabrze.pl
Anna Balcerska	hemonkp@amg.gda.pl
Michal. Matysiak	mmatysiak@litewska.edu.pl
Mariusz Wysocki	hematonko@by.home.pl
Maryna Krawczuk-Rybak	rybak@amb.edu.pl
Tomasz Urasinski	urasin@sci.pam.szczecin.pl
Jerzy Bodalski	jot@toya.net.pl
Grazyna Sobol	onkohematologia@wp.pl
Grazyna Karolczyk	karolczykg@chok.kielce.pl
Maria Wieczorek	mariawiecz@poczta.onet.pl
Anna Skowronska Gardas	agardas@coi.waw.pl
Krzysztof Paprota	lekkp@gazeta.pl
Jadwiga Maldyk	mkanold@neostrada.pl
Angelina Moryl-Bujakowska	amb6000@op.pl

23.8 SCIENTIFIC ADVISORY BOARD

Current members of the Scientific Advisory Board are listed below (status quo November 2005). Further members can be appointed by the clinical board of the EuroNet-PHL-group.

Chairpersons: Prof. Dr. S. Burdach, Munich
Prof. Dr. W. H. Wallace
Prof. Dr. D. Körholz

Members of the Scientific Advisory Board

Prof. Dr. Stein, Berlin
Dr. Hasenclever, Leipzig
PD Dr. Berger (Switzerland)
Dr. Malcolm Taylor, Manchester, UK
Dr. Paul Murray Birmingham, UK
Prof. D. Machin, UK
Prof. Judith Landman-Parker, F
Prof. Dr. Walentyna Balwierz
